# Access to 2-Fluorinated Aziridine-2-phosphonates from *α*,*α*-Halofluorinated *β*-Iminophosphonates—Spectroscopic and Theoretical Studies †

**DOI:** 10.3390/molecules28145579

**Published:** 2023-07-22

**Authors:** Mateusz Klarek, Tomasz Siodła, Tahar Ayad, David Virieux, Magdalena Rapp

**Affiliations:** 1 Faculty of Chemistry, Adam Mickiewicz University, Uniwersytetu Poznańskiego 8, 61-614 Poznań, Poland; mateusz.klarek@amu.edu.pl (M.K.); tomasz.siodla@amu.edu.pl (T.S.); 2 Institut Charles Gerhardt, CNRS, Ecole Nationale Supérieure de Chimie de Montpellier, 8 Rue de l’Ecole Normale, 34296 Montpellier, France; tahar.ayad@enscm.fr (T.A.); david.virieux@enscm.fr (D.V.)

**Keywords:** imines, phosphonates, aminophosphonates, halofluorination, aziridine, DFT calculations

## Abstract

The efficient one-pot halofluorination of a *β*-enaminophosphonate/*β*-iminophosphonate tautomeric mixture resulting in *α*,*α*-halofluorinated *β*-iminophosphonates is reported. Subsequent imine reduction gave the corresponding *β*-aminophosphonates as a racemic mixture or with high diastereoselectivity. The proposed protocol is the first example of a synthesis of *N*-inactivated aziridines substituted by a fluorine and phosphonate moiety on the same carbon atom. Based on spectroscopic and theoretical studies, we determined the *cis*/*trans* geometry of the resulting fluorinated aziridine-2-phosphonate. Our procedure, involving the reduction of *cis/trans*-fluoroaziridine mixture **24**, allows us to isolate chiral *trans-*aziridines **24** as well as *cis-*aziridines **27** that do not contain a fluorine atom. We also investigated the influence of the fluorine atom on the reactivity of aziridine through an acid-catalyzed regioselective ring-opening reaction. The results of DFT calculations, at the PCM/ωB97x-D/def2-TZVPD level of theory, are in good agreement with the experiments. The transition states of the S_N_2 intramolecular cyclization of vicinal haloamines have been modeled.

## 1. Introduction

The aziridine motif frequently appears in biologically active compounds [1,2,3,4,5,6,7]. Therefore, this heterocyclic three-membered ring motif also serves as an attractive building block for organic transformations owing to the strained ring system and its ability to undergo highly regio- and stereospecific ring-opening reactions [8,9,10,11,12,13,14,15,16,17,18].

**Figure 1:**
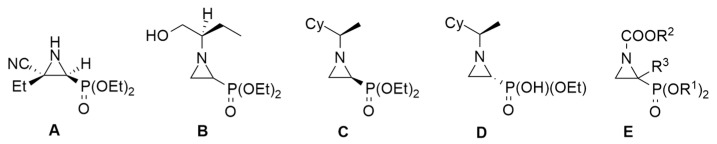
Biologically active aziridinyl phosphonates.

Among the commonly encountered substituents in aziridine systems, heteroatoms or heteroatom-based groups, such as fluorine or phosphonates are of special interest [8,14,19,20,21,22,23,24,25]. Incorporating electronegative fluorine atoms in organic molecules often dramatically influences the physical and biological properties of the parent compounds [26,27,28,29,30,31,32,33]. Furthermore, the presence of fluorine has been reported to have profound effects on the reactivity of aziridines and the regioselectivity of ring-opening reactions. For instance, the reaction of *N*-substituted 2,2-difluorinated aziridines with aqueous HCl or MeONa in methanol led to *α*-chloroacetamides [34,35] or *α*-methoxyacetamide [34], respectively. The nucleophilic attack occurs preferentially from the less-hindered side. It is important to note that exposure of 2,2-difluorinated aziridines to moist air results in the formation of *α*-fluoroacetamides [35]. In contrast, when 2-monofluoro aziridines are subjected to gaseous HCl or sodium methoxide, they provides access to 2-chloro-2-fluoroamines or 2,2-dimethoxyethylamine [34]. This reaction occurs from the ring opening at the more hindered position of aziridine ring. As a comparison, Konev et al. reported the transformation of 2-fluoro aziridine to fluorinated propargyl amines through *α*-formation of a fluoro imine intermediate [36]. These findings suggest that fluorinated aziridines, owing to the ring strain and the presence of fluorine atom, can be regarded as valuable reagents for the synthesis of nitrogen-containing compounds. On the other hand, the presence of a phosphonate function on aziridine also induces interesting behaviors. 2-Phosphonoaziridine derivatives have been recognized for their biological properties (Figure 1). The cyanoaziridinylphosphonate **A** has exhibited antiproliferative activity (in vitro) against human cancer cell lines derived from human lung adenocarcinoma (A549 strain, IC_50_ = 1.5 ± 0.84 μM) [37]. Additionally, *N*-functionalized 2-phosphonoaziridines **B** and **C** displayed moderate activity against the bacteria *E. coli*, and *Kocuria* spp. (Fs24) [38], as well as moderate antifungal activity against *C. albicans* ATCC 10,231 (MIC 12.5 μg/mL), respectively [39]. In comparison, the aziridynyl 2-phosphonic acid monoester **D** is less active than the diester counterparts, and exhibited moderate or low antibacterial activity against *E. coli, K. pneumoniae, A. baumanii,* and *P. aeruginosa* [40]. Moreover, racemic series of 1-alkoxycarbonyl-2-phosphonoaziridine **E** have also been found to possess antibacterial activity [41].

Furthermore, the presence of a phosphonate group attached to the aziridine ring offers a promising pathway for the synthesis of aminophosphonic acids and their derivatives [22,23,24,42,43,44,45]. Aminophosphonates structurally and functionally mimic the amino acids. The tetrahedral phosphonic acid is an effective surrogate of the planar carboxylic group, making them attractive targets for the development of biologically active compounds [32,33,46,47,48,49,50,51,52].

**Figure 2:**
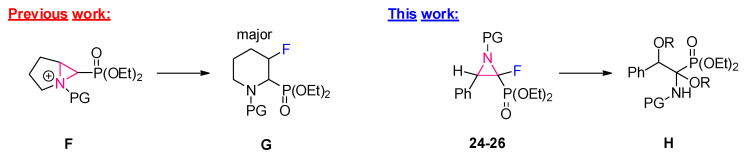
*α-*Aminophosphonates formed through aziridine ring-opening reaction.

In a recent study, we successfully achieved the diastereoselective synthesis of fluorinated piperidine phosphonates from *N*-substituted hydroxyphosphonates derived from proline [53]. The fluorination occurred through an aziridinium intermediate **F**, and subsequent ring opening, resulting in ring expansion **G** (Figure 2). The diastereoselectivity of the deoxyfluorination process appeared to depend on the combination of neighboring group and bulky phosphonate influences, among other factors. Motivated by these findings, we decided to apply our protocol [54] to the synthesis of a series of *N*-substituted *α*-halofluorinated aminophosphonates **16**–**21**. Both achiral and optically active aziridinylphosphonates **24**–**26** could be potentially obtained. Additionally, we will form optically enriched aminophosphonates **H** through the selective aziridine ring opening (Figure 2).

## 2. Results and Discussion

### 2.1. Synthesis of α,α-Halofluoro-β-aminophosphonates via Reduction of α,α-Halofluoro-β-iminophosphonates

We initiated the sequence by the condensation of diethyl *ß*-ketophosphonate **1** with a series of primary amines, respectively, (*S*)- and (*R*)-*α*-methylbenzylamine (MBn-NH_2_), *p*-methoxybenzylamine (PMB-NH_2_), and *p*-methoxyphenylamine (PMP-NH_2_). These reactions resulted in a tautomeric mixture of major *β*-enaminophosphonates (**2**–**5**) and minor *β*-iminophosphonates (**6**–**9**) [54,55]. When *p*-methoxyphenylamine was used, the ratio of enamine (*Z*)/imine (*E*:*Z*) **5a**/**9a,b** was 1/0.53 (1:0.1), as determined by ^31^P NMR. The *Z* geometry of *β*-enamines was confirmed by the chemical shifts and coupling constants *J*_CP_ values of C(*β*) and C(*α*) signals (δ: 161.6, ^2^*J*_CP_ 6 Hz and δ: 82.2, ^1^*J*_CP_ 188 Hz, respectively) in ^13^C NMR spectra. The signals of *C*=N and an *ipso* carbon atom (attached to the double bond) appeared at δ: 160.1, ^2^*J*_CP_ equals to 8 Hz and δ: 144.0, ^3^*J*_CP_ 2 Hz, respectively, matching the main *E* isomer of the imine **9a** (Table 1). In a second step, a mild and efficient one-pot *gem*-bromo- or *gem*-chlorofluorination of **2**–**9** led to the formation of imines (**10**–**11**,**14a,b** and rac-**12**–**13**,**15**) possessing chiral (*S*)- or (*R*)-MBn or non-optically active PMB or PMP protecting groups (PG).

The one-pot *gem*-bromofluorination or *gem*-chlorofluorination of crude *E/Z* enamine/imine (**2**–**5**/**6**–**9**) mixture was accomplished using Selectfluor and *N*-bromosuccinimide (NBS) or *N*-chlorosuccinimide (NCS) yielding *α,α*-bromofluoro or *α,α*-chlorofluoro-*β*-iminophosphonates (**10**–**11,14a,b** and rac-**12**–**13,15**). The reactions, monitored by ^31^P NMR, proceeded in yields ranging from 87% for rac-**13** to 95% for **11a**,**b** (*dr* 1:1). The formation of *β*-iminophosphonates was established based on NMR experiments and IR spectra. Analysis of the ^19^F NMR spectra of **10a** and **10b** (*dr* 1:1) presented two doublets corresponding to the diastereomers at δ: −129.1 and −128.7 (^2^*J*_FP_ 82 Hz). The ^31^P NMR spectra also showed two doublets at δ: 7.4 and 7.9 with the same coupling constants. Similar signals for the chlorofluorinated derivative (**14a**,**b** *dr* 1:1) were observed at δ: −125.6 and −125.4 (d, ^2^*J*_FP_ 86/87 Hz) in the ^19^F NMR and at δ: 8.0 and 7.5 in the ^31^P NMR, respectively. This trend is in good agreement with differences in chemical shifts observed in brominated or chlorinated fluoro-organic compounds. [56] Moreover, the *C*=N bond gave a distinctive doublet of doublets in the ^13^C NMR spectra of **10a**,**b** at δ: 164.4/164.6 as a (^2^*J*_CF_ 27/28 Hz, ^2^*J*_CP_ 6 Hz). The stretching band at 1648 cm^−1^ in the IR spectra also confirmed the presence of the C=N bond. [57] The halogenated *β*-iminophosphonates (**10**–**11,14a,b** and rac-**12**–**13,15**) were formed as a single isomer. Due to steric hindrance between the protecting group (PG), phosphonate moiety, and bromine/chlorine atom, the resulting geometry of the imine bond was attributed to the *E*-isomer and was confirmed by HSQC, HMBC, and 2D NOESY experiments. For instance, the 2D ^1^H-^1^H NOESY experiment of **14a** indicated significant correlations between the proton -C*H*(CH_3_)Ph from the (*S*)-MBn group and the *ortho* proton from the phenyl ring located at imine carbon -NC(*Ph*)CFP (see the Supporting Information for details). On the other hand, for *α,α*-difluorinated *β*-iminophosphonate analogs, the through-space interactions of PG (benzylic) protons and one of the fluorine atoms indicated the major formation of the *Z*-stereoisomer [54]. Long-range F-H intramolecular nonbonding interactions have also been observed in the case of (*Z*)-*N*-tetrafluoropropenyl-uracil/thymine derivatives and *α*-fluorinated imines [35,58]. Moreover, it is worth noting that less than 10% of difluoro, dibromo, or dichloro derivatives were also formed during the reaction. They can be removed by column chromatography. Only in the case of **5a**/**9a,b** tautomers was it necessary to purify the crude reaction mixture due to the formation of impurities in the subsequent step.

**Scheme 1:**
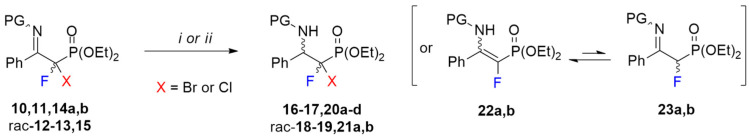
Reduction of *α,α-*halofluorinated *ß*-iminophosphonates (**10,11,14a,b,** rac-**12**–**13,15**). Reaction conditions: *i.* imine (0.5 mmol), NaBH_3_CN (3 mmol), glacial CH_3_COOH (3 mmol), MeOH, rt, 40 min. *ii.* **10a**,**b** (0.3 mmol), LiAlH_4_ (0.45 mmol), THF, 0 °C 🡪 rt, 60 min.

The obtained *α,α*-halofluorinated *ß*-iminophosphonates **10**–**15** were subsequently used as precursors for the synthesis of *ß*-aminophosphonates **16**–**21** (Scheme 1). Thus, the imine bond reduction using NaBH_3_CN in glacial acetic acid resulted in the formation of amines (**16**–**17, 20a**–**d** and rac-**18**–**19, 21a,b**) as a mixture of diastereomers in yields ranging from 91 to 97%. Furthermore, the reduction of **10a**,**b** using LiAlH_4_ (LAH) led to the formation of a monofluorinated tautomeric mixture of enamine **22a,b**/imine **23a,b** [59] in 83% yield (*E-***22a**/*Z-***22b** ratio 1:0.3; **22a,b**/**23a,b** ratio 1:0.05). This was attributed to the substitution of the bromine atom in the imine **10a**,**b** by the hydrogen and subsequent tautomerization to form the enamine **22a,b**/imine **23a,b** tautomers (Scheme 1). In contrast, De Kimpe et al. reported that the application of LAH in the reduction of halogenated imines led to reductive cyclization and the formation of an aziridine ring [60,61].

**Figure 3:**
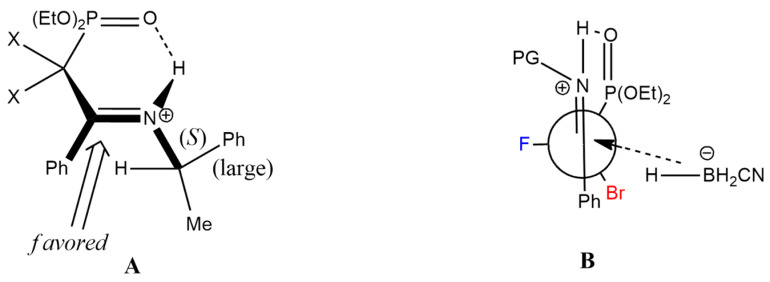
Diastereoselective approach for imine reduction based on: (**A**)—rigid Houk model; (**B**)—Felkin-Anh model for major isomer; PG: PMB or PMP.

We observed that the reduction of **10**–**11**,**14a,b** substituted by the chiral (*S*) or (*R*)-*α*-methylbenzyl groups resulted in asymmetric induction, yielding aminophosphonates **16**–**17**,**20a**–**d** with high diastereoselectivity. The configuration of the new stereogenic C-2-carbon center strongly depends on a chiral protecting group (C-1′). The favorable hydride anion attack occurs from the least hindered face of the imine rigidified by an intramolecular hydrogen bond, which according to the Houk model (Figure 3A), yields **16a**–**d** as a diastereomeric mixture (*dr* 1:1:0.1:0.07) predominantly containing two major (presumably 1′*S*, 1*R*, 2*R* and 1′*S*, 1*S*, 2*R*) and two minor (1′*S*, 1*S*, 2*S*, and 1′*S*, 1*R*, 2*S*) isomers. The diastereoselection only depends on steric factors between C-H moiety, which eclipses the double bond of imine, and the methyl group and phenyl ring derived from the (*S*)-MBn protecting group [62]. Transformations of imines containing this particular protecting group frequently led to asymmetric induction in different types of nucleophilic addition to the imine C=N bond [54,63,64,65]. In comparison, the reduction of rac-**12**–**13**,**15** which have achiral protecting groups (PMB or PMP) leads to aminophosphonates rac-**18**–**19**,**21a,b** as the mixture of two diastereomers, with a slight preference for one isomer and a *dr* ratio ranging from 1:0.7 for rac-**18**,**21a,b** to 1:0.88 for rac-**19a,b**. For the major diastereomer, the hydride addition to C=N occurs from the most unhindered side with the most electronegative group (F) perpendicular to the imine bond and the system stiffened by an intramolecular H-bond (Figure 3B) [66,67]. Analogous diastereoselectivity has also been observed during the synthesis of fluorinated epoxy alkylphosphonate from *α*-fluoro-*β*-keto alkylphosphonates [53]. Based on these data, we temporarily established the stereochemistry of major **18**–**19,21a** as rac 1*R*, 2*R* and the minor isomers **18**–**19,21b** as rac 1*R*,2*S* (e.g., 1*R*,2*S/*1*S*,2*R*). The stereochemical hydride addition to the imine C=N bond, based on the proposed Houk model (Figure 3A) for **10a,b** and the Felkin-Anh model (Figure 3B) for rac-**12*,*13** leading to major diastereomers is presented on Figure 3.

To optimize the reduction of imines **10**–**11,14a,b** and rac-**12**–**13,15**, various activations were tested, including organic acid (glacial acetic acid), Lewis acid (anhydrous CeCl_3_), and chiral organic acid [(1*S*)-(+)-camphorsulfonic acid (CSA)]. The yields and diastereomer ratios of amines **16**–**17**,**20a**–**d**, rac-**18**–**19**,**21a,b**, based on ^19^F and ^31^P NMR analysis of the crude reaction mixture are summarized in Table 2. Among the tested conditions, the combination of sodium cyanoborohydride and acetic acid (entry 1, Table 2) for the imines **10a**,**b** reduction gave the highest yield (97%) of **16a-d**, (*dr* 1:1:0.1:0.07). On the contrary, the use of BH_3_^.^THF furnished the highest diastereoselectivity with a *dr* of 1:0.25:ca.0:ca.0 and a high yield of 91% (entry 4, Table 2). However, changing borane to BH_3_ × S(CH_3_)_2_ resulted in a drastic decrease in yields to 47% with a *dr* of 1:0.20:ca.0:ca.0 (entry 5, Table 2). When rac-**12** was reacted with NaBH_3_CN/HOAc, rac-**18a**,**b** was obtained with a predominance of one diastereomer (*dr* 1:0.7) and a yield of 97% (entry 8, Table 2). Chiral organic acid (1*S*)-(+)-CSA activation gave the amine 83% yield and a *dr* of 1:0.74 (entry 10, Table 2). Furthermore, the reaction with Lewis acid CeCl_3_ and NaBH_3_CN (entry 9, Table 2) led to higher diastereoselectivity, yielding rac-**18a**,**b** with a *dr* of 1:0.5 (85% yield). As previously mentioned, the diastereoselectivity of the *E*-imine reduction strongly depended on the nature of the nitrogen-substituent. The hydride addition to rac-**13** (PG = 4-methoxyphenyl) gave a poor selectivity of rac-**19a,b** with a *dr* of 1:0.88 (entry 11, Table 2), while higher diastereoselectivity was observed with the 4-methoxybenzyl (PMB) group, leading to rac-**18a,b** with a *dr* of 1:0.70) (entry 8, Table 2). Moreover, replacing the bromine atom by a chlorine atom (**14a,b,** rac-**15**) did not significantly affect the yields nor diastereomeric ratios (entry 12–13, Table 2).

**Figure 4:**
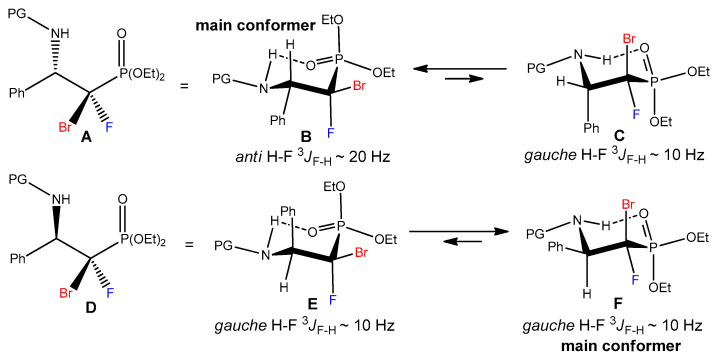
Chair-like conformers (**B**,**C** and **E**,**F**) representation of amine minor (**A**) and major (**D**) diastereomers. PG = PMB, PMP.

The diastereoselectivity of reduction was assessed by spectral analysis of products. The diastereomers of amines **16**–**17**,**20a**–**d,** and not chiral (±)-like and (±)-unlike stereoisomers rac-**18**–**19**,**21a,b** were distinguished based on their respective vicinal ^3^*J*_FH_ coupling constants, which depend on the Karplus equation [56,68]. The *β*-aminophosphonates and their acids can exist in CDCl_3_ in major “frozen” chair-like conformation, due to an intramolecular hydrogen bonding between the amino group and the phosphoryl group (Figure 4) [69]. The (±)-*unlike* isomer (Figure 4A) presented two conformations where the more stable one presented an *anti*-periplanar H-F arrangement (^3^*J*_FH_ ~20 Hz, Figure 4B), while the minor one is a *gauche*-conformation (^3^*J*_FH_ ~10 Hz, Figure 4C) [54,56]. By contrast, the two conformations of the (±)-*like* isomer (Figure 4D) only presented *gauche*-orientations (Figure 4E,F). Furthermore, due to the bulkiness of the phenyl and phosphonate groups (both likely in *anti-*position), we attributed the configuration of **16a** as 1′*S*, 1*R*, 2*R* and for **16b** as 1′*S*, 1*S*, 2*R,* while the major or minor isomers of rac-**18-19**, **21** were assigned as rac-1*R*, 2*R* or rac*-*1*R*, 2*S*, respectively.

On the other hand, apart from the vicinal H-F coupling constant values between fluorine and hydrogen nuclei and the [N–H…^.^O=P(OEt)_2_] hydrogen bond, it was necessary to take into account the electrostatic interactions between C-F and N-H in relation to the structure of each conformer.

### 2.2. Conformational Analysis of α,α-Halofluorinated ß-Aminophosphonates by DFT Calculations

**Figure 5:**
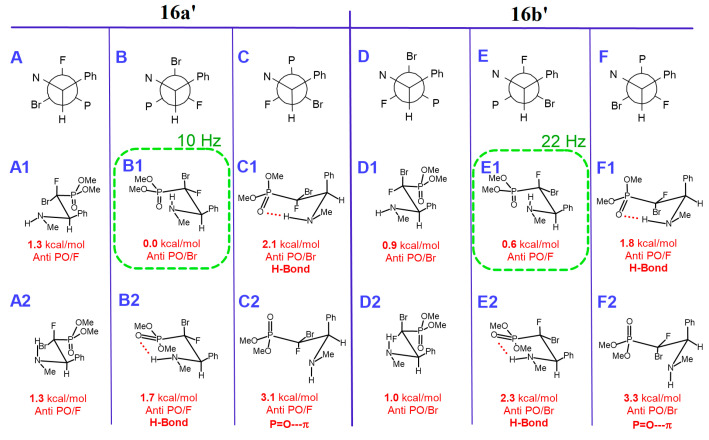
Potential conformers of *β*-aminophosphonates **16a’** (**A**,**B** and **C**) and **16b’** (**D**,**E** and **F**) and their relative potential energies [kcal/mol].

To explain the differences in H-F coupling constants, and to confirm the relative configuration of stereogenic centers for both *β*-aminophosphonate diastereomers **16a** and **16b**, a conformational analysis was conducted using the PCM/ωB97x-D/def2-TZVPD level of theory. To simplify the calculations, the ethoxy groups were substituted with methoxy groups, and the PG group was replaced with a methyl group (Figure 5 **A**–**F 1**–**2**). The potential conformations for both diastereomers (**16a’** or **16b’**) were clustered into three groups corresponding to three distinct arrangements of the C(*β*)H**–**C(*α*)F single bond: **A**, **B**, and **C** for **16a’**, or **D**, **E**, and **F** for **16b’**. (Figure 5) All structures were drawn in the chair-like representation to show the conformations of all relevant bonds. The most thermodynamically stable conformations within each group were labeled with the number **1** (**A1–F1**), while geometries labeled with the number **2** (**A2–F2**) were selected for comparative purposes. All energies presented were calculated in reference to the most stable conformer **B1**, which possessed the lowest energy.

**Figure 6:**
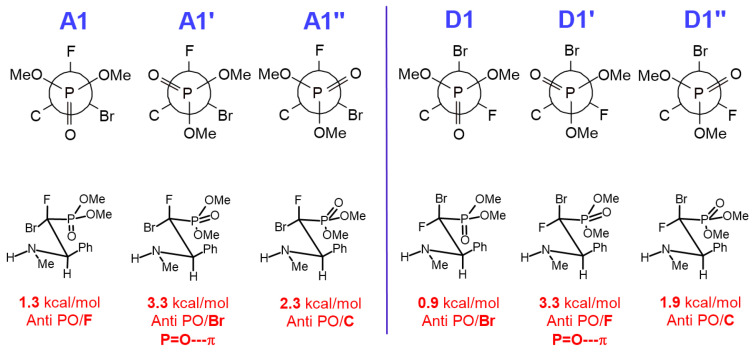
Selected conformers and their relative potential energies [kcal/mol] arise from the rotation of the phosphonate moiety for **16a’** (**A**) and **16b’** (**D**).

The **B1** geometry with the H-F atoms of the C(*β*)H**–**C(*α*)F bond in the *gauche* position was determined to be the most favorable conformation for **16a’**. Moreover, the most stable conformation for **16b’** was the **E1** conformation (+ 0.6 kcal/mol), where the vicinal H-F atoms were *anti*. These results are in good agreement with the experimental NMR coupling constants: *^3^J*_HF_ *~*10 Hz and *^3^J*_H-F_ ~22 Hz for **16a’** and **16b’**, respectively. Analyzing the relative energies of the conformers depicted in Figure 5, it becomes evident that the electrostatic interaction between N-H and C-Br or C-F (present in the **A1**, **A2**, **B1**, **C2**, **D1**, **D2**, **E1**, **F2**) likely plays a more significant role than the stabilization via the P=O…H-N hydrogen bonding (present in the **B2**, **C1**, **E2**, **F1**). However, in the case of **C** and **F** conformations, the preference is reversed: the geometries with the P=O…H-N hydrogen bond (**C1** and **F1**) are more stable. This phenomenon is likely due to the phosphonate−aromatic (P=O…π) repulsive interaction [70], which destabilizes conformers **C2** and **F2**. The same interaction explains why the most stable geometries of conformers **A (A1** and **A2)** are the *anti*-arrangement of P=O/C-F, while the most stable geometries for conformers **D (D1** and **D2)** have the *anti*-arrangement of P=O/C-Br (Figure 5). As shown in Figure 6, the *anti*-arrangement of P=O/C-Br (conformer **A1′**) leads to a phosphonate−aromatic (P=O…π) repulsive interaction, resulting in an energy increase up to +3.3 kcal/mol. A similar effect can be observed for **D** conformations, which have the *anti*-arrangement of P=O/C-F (**D1′**). Conformations **A1′’** and **D1′’**, both having the *anti*-arrangement of P=O/C-C, are not preferred due to the unfavorable dipole-dipole interactions between P=O and C-F/C-Br occupying the gauche position (Figure 6).

Furthermore, comparing the energy of **A1** and **A2** (or **D1** and **D2**) reveals an equivalence in the electrostatic interaction between N-H and C-F vs. N-H and C-Br. This conclusion is also supported by comparison of geometries that differ only in the position of the -F and -Br substituents. These pairs of geometries include **A1** and **D1**; **A1′** and **D1′**; **A1′’** and **D1′’**; **A2** and **D2**; **B1** and **E1**; **B2** and **E2**; **C1** and **F1**; and **C2** and **F2** (Figure 5 and Figure 6). The small differences in stability between these structures (maximum of 0.6 kcal/mol) indicate similar effects caused by C-Br and C-F bonds on the overall energy of the molecule.

In summary, the conformational analysis described above allows us to conclude that the stability of P=O--H-N hydrogen bonding can be outweighed by the electrostatic interaction between C-F (C-Br) and N-H, unless it leads to phosphonate−aromatic (P=O…π) repulsive interactions.

### 2.3. Aziridine Synthesis by Ring Closure Reaction

**Scheme 2:**
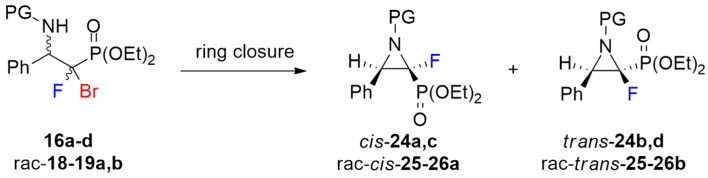
Ring closure of *α,α*-bromofluoro-*β*-aminophosphonate (**16a**–**d,** rac-**18**–**19a,b**) to 2-fluoroaziridinyl-2-phosphonate (**24a**–**d,** rac-**25**–**26a,b**). PG = (*S*) or (*R*)-MBn, PMB, PMP. Reaction conditions: amine **16a**–**d,** rac-**18**–**19a,b** (0.5 mmol), TEA (0.6 mmol), DMF, 70 °C, 4 h, Schlenk flask, inert gas. Note*:* for simplicity, in the case of **24a**–**d**, only the stereochemistry of the major diastereomers (**24a,b**) was shown.

Our approach to aziridine synthesis involved the intramolecular S_N_2 nucleophilic substitution of a halogen atom by a vicinal amine leading to the formation 2-fluoro-aziridinyl-2-phosphonates **24**–**26**. We observed that bromofluorinated aminophosphonates **16a**–**d,** rac-**18**–**19a,b**, when left at room temperature, undergo spontaneous transformation into aziridines, with 50% of conversion over the course of one month. This process depends on the nature of the diastereomers. For amine **16a**–**d**, only a pair of isomers **16b,d** cyclized to form the aziridines *trans*-**24b,d**. To expedite this reaction, treatment of the *ß*-aminophosphonates **16a**–**d,** rac-**18**–**19a,b** with TEA in DMF for 4 h gave a mixture of diastereomers of fluoro-aziridinylphosphonates **24a**–**d,** rac-**25**–**26a,b** in yields ranging from 48% to 82% (Scheme 2).

To enhance the rate of the cyclization reaction, an optimization was performed by testing various solvents, adjusting the base addition, and changing reaction conditions such as time and temperature (from room temperature to 70 °C). The outcomes of this screening are summarized in Table 3.

The optimization results showed that cyclization initiated by triethylamine (TEA) in *N*,*N*-dimethylformamide (entry 1, Table 3), as well in acetonitrile (entry 2, Table 3), were the most favorable for obtaining **24a**–**d** in yields of 82% and 70%, respectively. Toluene, dichloromethane, and tetrahydrofuran (entry 3–5, Table 3) were considered as the least favorable solvents. A better pale, but still poor yield was observed, while the reaction was conducted in dimethyl carbonate (DMC) (entry 6, Table 3). When we monitored the same reaction for chlorofluorinated derivatives (**20a**–**d**) (entry 9, Table 3), only signals of substrates were detected (^19^F, ^31^P NMR). Moreover, it should be pointed out that no racemization occurred during the aziridine formation.

Additionally, we observed that while two out of four amine diastereomers **16b,d** and rac-**18**–**19b** (*unlike*-diastereomers) were readily transformed into *trans*-aziridine **24b,d** and rac-**25**–**26b**, the conversion was not complete and the *like*-ones did not cyclize into *cis*-aziridines **24a,c** and rac-**25**–**26a**. According to our observations, they underwent degradation leading to several unidentified products, which could explain the different diastereomeric ratios after reaction, with the *trans*-isomer being dominant. A shorter reaction time resulted in incomplete substrate conversion, while a longer reaction time influenced the formation of by-products. In addition, we noticed a particularly low stability of the *cis*-isomer for rac-**26a** (entry 8, Table 3), which can be explained by steric factors.

Moreover, we also studied the influence of a base on aziridine ratio and reaction yield (Table 4). The reaction of **16a**–**d** (*dr* 1:1:0.1:0.07), with TEA as a base in DMF at 70 °C, gave **24a**–**d** with a *cis/trans* ratio of 0.78:1 (entry 1, Table 4). Without a base at 70 °C in DMF (entry 7, Table 4), the result was a low 38% yield of **24a**–**d**, due to partial decomposition of starting material. Under the same conditions using sodium hydride, only traces of **24a**–**d** were detected by ^19^F NMR in the crude mixture (<10% yield) (entry 6, Table 4). Using quinine (entry 2, Table 4) gave the best yield of 84% for **24a**–**d**. By contrast, DBU mainly led to the formation of monofluorinated enamine (*E-***22a**/*Z-***22b** ratio 1:0.3) and imine (**23a,b**) (**22a,b**/**23a,b** ratio 1:0.05) in 21% yield (entry 4, Table 4), while DIPEA furnished **24a**–**d** in 70% yield and a *cis*/*trans* ratio of 0.67:1 (entry 5, Table 4). Surprisingly, the cyclization of **16a**–**d** performed with *L*-proline resulted in a 77% yield of **24a**–**d** with a *cis*/*trans* ratio of 0.69:1 (entry 3, Table 4). Selected bases (TEA, quinine, and proline) were also tested for the amine rac-**18a,b** (entries 8–10, Table 4), and no significant differences were observed compared to **16a**–**d**.

We have noticed that the aziridines **24a**–**d** and rac-**25**–**26a,b** are air-stable and can be stored for several months without any sign of degradation, as confirmed by ^31^P NMR analysis. Unlike the difluoroaziridines reported by De Kimpe et al., no spontaneous fluorine migration was observed [34,35]. During the purification of the crude using silica gel column chromatography, we found that prior deactivation with 1% of triethylamine was necessary. This allowed yields to increase from ~20–30% to ~50–60%, respectively. Similar observations have been reported in the literature for fluorinated aziridines [35].

### 2.4. Spectroscopic Studies Concerning Cis- and Trans-Aziridines

The structure determination of *cis*- and *trans*-aziridines was based on the NMR experiments performed on rac-**25a**,**b**. In the ^19^F NMR spectrum, the major *trans*-isomer rac-**25b** showed a signal at δ −171, appearing as a doublet of quartets with a ^2^*J*_FP_ equal to 112 Hz. The aziridine proton (^3^*J*_FH_ 4 Hz, -C*H*(Ph)CF), and long-range coupling with two benzyl protons (^4^*J*_FH_ 5 Hz). The analysis of ^31^P{^1^H} NMR spectra showed a signal a doublet at δ: 10.9, while the signal derived from the *cis*-isomer was observed at δ: 9.97 (d, ^2^*J*_PF_ 117 Hz). A distinct signal difference was also observed in the ^19^F NMR spectrum of rac-**25a**, where the signal was located at δ: −180 (dd, ^2^*J*_FP_ 117 Hz, ^3^*J*_FH_ 8 Hz). Additionally, there was no coupling between the fluorine and the benzyl proton for the *cis*-isomer. The ^1^H NMR spectrum indicated the non-equivalence of benzyl protons [71], which resulted in the splitting of the signal into two separate doublets or a doublet of doublets (rac-**25a**: br d at δ: 4.0 and 4.1, ^2^*J*_HH_ 14 Hz; rac-**25b**: dd at δ: 4.1, ^2^*J*_HH_ 14, ^4^*J*_HF_ 5 Hz, and 4.5, ^2^*J*_HH_ 14, ^4^*J*_HF_ 3 Hz). In the ^1^H NMR spectrum, the key signal from the aziridine proton was observed at δ: 3.45 (t, ^3^*J*_HF_ and ^3^*J*_HP_ 4 Hz) for the *trans*-isomer (rac-**25b**), and at δ: 3.26 (d, ^3^*J*_HF_ 8 Hz) for the *cis-*isomer rac-**25a**. Performing ^1^H{/^19^F} NMR simplified both signals to a broad doublet at δ: 3.45 (^3^*J*_HP_ 3.6 Hz) and a broad singlet at δ: 3.25, respectively. However, the decoupling of the ^1^H{/^31^P} NMR spectrum simplified only the signal from rac-**25b** (d, ^3^*J*_HF_ 4 Hz). These data clearly indicated the aziridine protons from both isomers interacted with fluorine, while only the proton from *trans*-aziridine rac-**25b** was coupled with the phosphorus atom. These NMR results, along with the literature data, enabled the determination of the presumed geometry of aziridines. Furthermore, the electron-withdrawing substituents (P and F) decreased the values of vicinal couplings constants between hydrogen nuclei and heteroatoms [72]. In practice, the range of coupling constants for the substituted fluorinated aziridines can be estimated depending on the vicinal H-F relationship H-F*_trans_*: 2–5 Hz, H-F*_cis_*: 7–9 Hz [56,73,74]. Accordingly, we assigned a higher value of ^3^*J*_HF_ 8.4 Hz for H-F in the *cis*-relationship of rac-**25a**, and a lower ^3^*J*_HF_ 4.3 Hz, for the *trans* H-F in rac-**25b**, which is in good agreement with the literature. In the case of the *trans* orientation of the H-P bond, the assumed dihedral angle CHCP is approximately 180°, resulting in a vicinal coupling constant of ^3^*J*_HP_ around 0–1 Hz. This observation was also noticed in our case for rac-**25a** with a ^3^*J*_HP_ value of 0 Hz. Similar observations were reported for trifluoromethylcyclopropylphosphonates (^3^*J*_HP_ ca. 1 Hz) [75]. The above-described data were further confirmed by characteristic signals in the ^13^C NMR spectra, with a typical couplings constant for *α*-substituted aziridinylphosphonates, localized C-2 at δ 83.9 (dd, ^2^*J*_CF/P_ 260, 232 Hz) or 86.7 (dd, ^2^*J*_CF/P_ 274, 272 Hz), for the *trans*- and *cis*-aziridine, respectively. These significant results for rac-**25a,b**, along with analogous relationships, were also observed in the case of **24a**–**d** and rac-**26a,b** (see the Supporting Information for details). The summarized NMR data for rac-**25a,b** can be found in Table 5.

**Figure 7:**
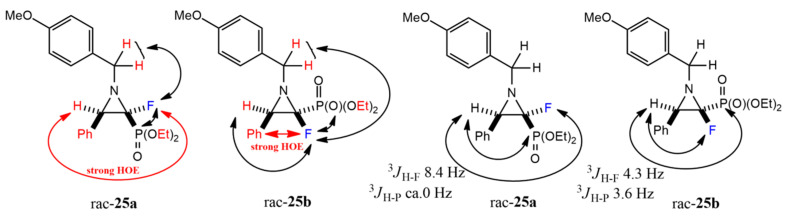
F-H correlations and H-F, H-P coupling constant values for *rac-***25a,b** based on *^1^*H*-^19^*F HOE and ^1^H, ^1^H{/^19^F}, ^1^H{/^31^P} NMR.

To support the stereochemical assessments, 1D ^1^H-^1^H nuclear Overhauser effect (NOE) experiments as well as 1D ^19^F-^1^H heteronuclear NOE (HOE) NMR experiments were performed for both isomers of rac-**25a,b**. In the 1D NOE spectrum, we observed correlations between the aziridine proton NC*H*CFP and diastereotopic benzyl protons for both isomers. Furthermore, we observed weak correlations between the aziridine proton NC*H*CFP (t, δ: 3.45 ppm) and the protons of the phosphonate group -P(O)(OC*H*_2_C*H*_3_)_2_, specifically in the *trans*-isomer (rac-**25b**), indicating that these substituents are on the same side of the ring. In addition, the HOE spectrum for the *trans*-isomer rac-**25b** revealed strong correlations between the fluorine atom and the closely located protons of the phenyl ring NCH(*Ph*)CFP. A similar correlation was observed for rac-**25a**, albeit with a noticeably weaker HOE effect. Based on these observations, there is a *cis*-relationship between the fluorine atom and the phenyl ring for rac-**25b**. In contrast, the most intensive enhancement in the HOE experiment was detected for correlation between the fluorine atom and the aziridine proton in rac-**25a** (Figure 7).

### 2.5. Study on Aziridine Ring Closure by DFT Calculations

**Figure 8:**
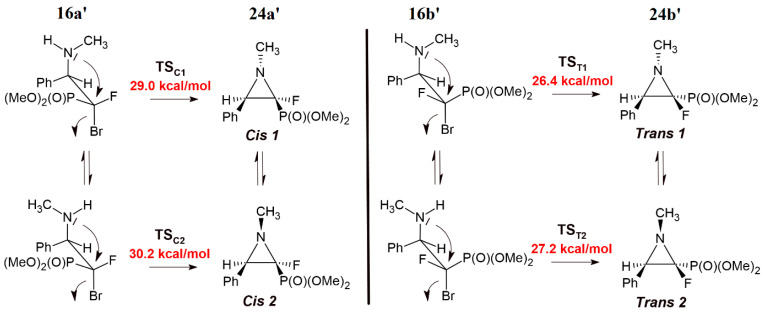
The proposed mechanism of aziridine **24a**,**b’** formation from **16a,b’** and relative potential energy barrier values.

**Figure 9:**
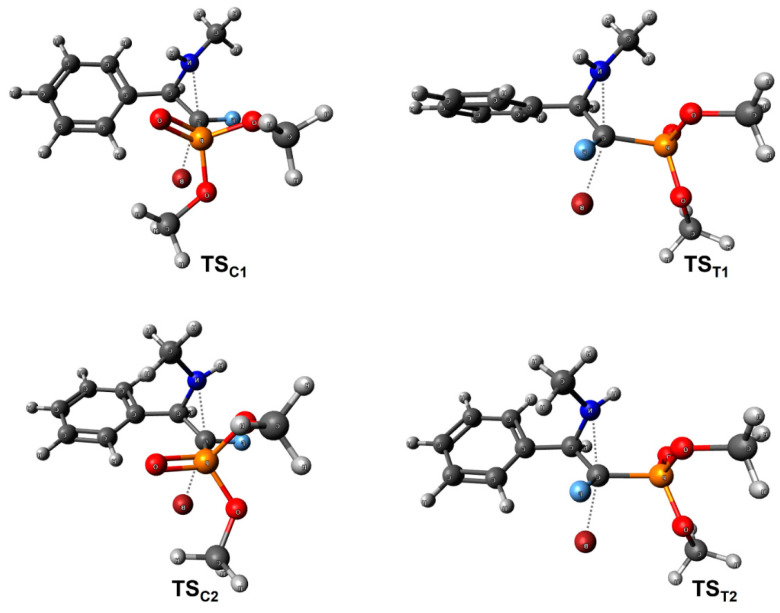
Optimized structures of transition states leading to the formation of aziridines presented in Figure 8.

To decipher the differences in the cyclization tendencies, DFT calculations were used to determine the potential energy barriers associated to the reaction pathways for all stereoisomers. Substrates **16a’** and **16b’** were utilized, resulting in products **24a’** and **24b’** (Figure 8). The calculated energies revealed that the transition states **TS_C1_** and **TS_C2_** leading to the formation of *cis*-aziridine **24a’** exhibited higher energy barriers (3 kcal/mol) compared to the transition states leading to the *trans*-aziridine (**TS_T1_**, **TS_T2_**), which is attributed to the difference of steric interactions between the phenyl and phosphonate groups (Figure 8). The optimized structures of the transition states leading to the formation of both *cis-* and *trans*-aziridines are presented in Figure 9. These results are in good agreement with the experimental findings where **16b** was found to undergo cyclization at a faster rate (approximately 50% over a month). Furthermore, the most stable conformer of **16b’** is **E1**, which exhibits an *anti*-arrangement of C-N and C-Br groups which promote the cyclization. Therefore, the *cis*-aziridine is kinetically favored.

### 2.6. Isolation of Chiral Trans-Aziridine ***24b***,***d*** and Non-Fluorinated Cis-Aziridine ***27***

**Scheme 3:**
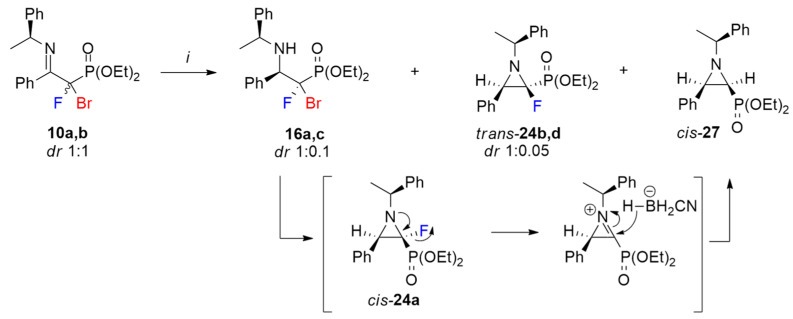
Reduction of **10a**,**b** at the higher temperature. Reaction conditions: *i*. **10a,b** (0.5 mmol), NaBH_3_CN (4 mmol), CH_3_COOH (1.5 mmol), MeOH, 70 °C, 3–7 h. Note: For simplicity, only the stereochemistry of the major diastereomers (**16a,b** and **24a,b**) was shown.

*Trans*-aziridine *trans*-**24b,d** can be obtained with high diastereoselectivity by the sequence involving an imine reduction and a subsequent ring closure reaction. When the reaction was carried out from **10a**,**b** with sodium cyanoborohydride in glacial acetic acid at 70 °C for 3 h (Scheme 3), we observed a diastereoselective reduction. Two of the four amine diastereomers (**16b,d**) underwent a direct conversion into *trans*-aziridines *trans*-**24b,d** with a *dr* of 1:0.05. In parallel, small amounts of non-fluorinated aziridine *cis*-**27** were also isolated. Extended reaction time favored the conversion of **16a,c** to *cis*-**27** (after 7h: **16a,c**/*trans-***24b,d**/*cis*-**27** 0.3:1:0.7), while prolonged reaction time led to the formation of some non-identified side-products. Based on these results, we hypothesized that diastereomers **16a,c** slowly cyclized to form *cis*-aziridines **24a,c** (Scheme 3). Then, the formation of an azirine intermediate resulting from the departure of the fluorine atom takes place, followed by the stereoselective addition of a hydride nucleophile at the opposite side of the aryl substituent, resulting in the stereospecific formation of aziridine *cis*-**27**. The stereochemistry of *cis*-**27** was deduced based on the observed vicinal coupling constants ^3^*J*_HH_ 7 Hz, which are in good agreement with the literature [76,77,78,79,80]. Similar conclusions were drawn by De Kimpe et al. in their study on the *N*-substituted *cis*-2-aryl-3-alkylaziridines [77].

**Scheme 4:**
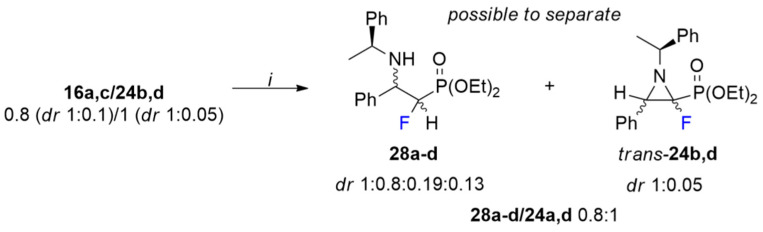
Synthetic approach to separate *trans*-aziridine **24b,d** from the mixture of **16a,c/***trans*-**24b,d**. Reaction conditions: *i*. **16a,c/***trans*-**24b,d** (0.3 mmol)*, NaBH_4_ (0.6 mmol), Pd/C (10 mol%), MeOH, 20 min, rt. * calculated for **16a,c**.

The aziridine *cis*-**27** is more polar than the fluorinated analogues and was isolated in 32% yield. To isolate the *trans-*aziridine **24b,d**, we treated a mixture of **16a,c**/*trans*-**24b,d** with sodium borohydride and a catalytic amount of Pd/C (10 mol%). Unfortunately, after 20 min at room temperature, we only observed the conversion of **16a,c** to amine products **28a**–**d** (Scheme 4). Interestingly, the presence of palladium catalyst promoted the diastereomerization of **16a,c**, resulting in the formation of the mixture of four diastereomers of the amine (**28a**–**d**) (*dr* 1:0.8:0.19:0.13) from **16a,c** (*dr* 1:0.1) as easily observed by ^19^F NMR. Finally, we were able to isolate of the *trans*-aziridine (**24b,d**) in 65% yield and very high diastereoselectivity (*dr* 1:0.06).

### 2.7. Aziridine Ring Transformations

**Scheme 5:**
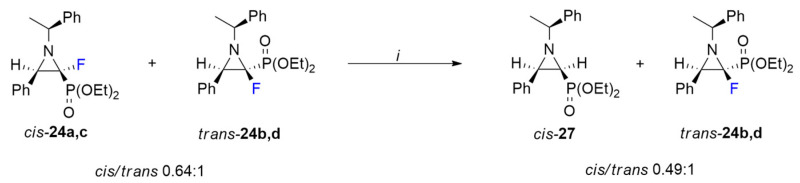
Aziridine transformation with NaBH_4_. Reaction conditions: *i*. **24a-d** (0.3 mmol), NaBH_4_ (0.6 mmol.), Pd/C (10 mol%), MeOH, 70 °C, 3 h. Note: For simplicity, only the stereochemistry of the major diastereomers was shown.

Finally, as previously mentioned, treatment of aziridine **24a-d** *cis/trans* 0.64(*dr* 1:0.07)/1(*dr* 1:0.09) with sodium borohydride afforded aziridine *cis-***27** in 39% yield via the formation of the azirine intermediate (Scheme 5).

As expected, the reaction selectively led to the conversion of one pair of diastereomers (*cis*-aziridine), while the other one (*trans*-aziridine) remained unchanged in the reaction mixture. By this sequence, both fluorinated and non-fluorinated aziridines can be isolated.

The reactivity of fluorinoaziridinyl-2-phosphonates was evaluated in a regioselective ring opening of **24a**–**d** (*cis/trans* 1(*dr* 1:0.45)/0.65(*dr* 1:0.04)). In such reactions, *N*-activation is required as the (*S*)-MBn is a poor leaving group [81]. Activation by Yb(OTf)_3_ and CeCl_3_ Lewis acids turned out to be ineffective. A similar observation was reported by Beksultanova et al. in their study related to the ring opening of aziridine-2-phosphonate catalyzed by BF_3_ × OEt_2_ [82]. This lack of reactivity may be due to the presence of a fluorine atom, which decrease the basicity of the nitrogen atom [83].

**Scheme 6:**
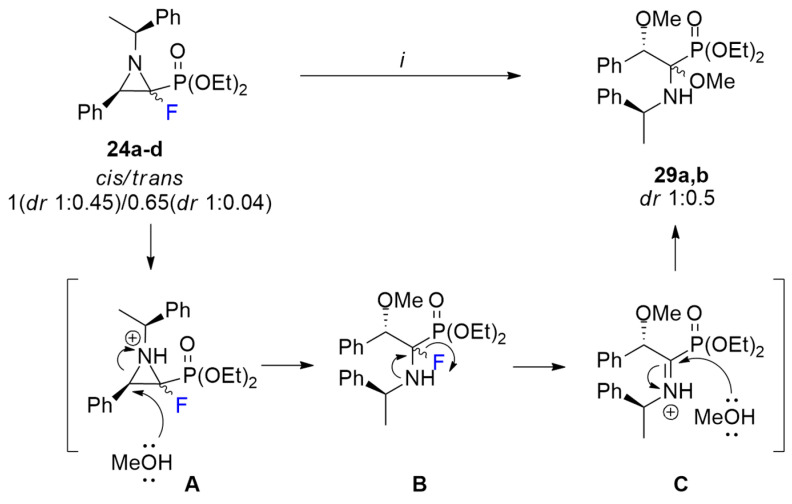
Acid-catalyzed ring-opening reaction of **24a**–**d** through intermediates **A**–**C**. Reaction conditions: *i*. **24a**–**d** (0.5 mmol), H_2_SO_4_ 98% (~0.5 mmol) MeOH, 70 °C, 2 h. Note: For simplicity, only the stereochemistry of the major diastereomers was shown.

For comparison, we tested the reaction on aziridines **24a**–**d** with methanol as a nucleophilic agent and sulfuric acid as an activating reagent. This reaction resulted in the formation of *β*-methoxy-*α*-hemiaminal phosphonates **29a,b** in 86% yield (*dr* 1:0.5) according to the mechanism depicted in Scheme 6.

First, MeOH attacked the acid-activated aziridine at the less hindered carbon atom, resulting in the formation of *α*-fluoro-*α*-aminophosphonate with inversion of configuration at the C-2-carbon atom. Then, an elimination led to the iminium **C**, which readily reacted with methanol to form **29a,b** with a *dr* of 1:0.5. Based on the Houk model discussed at the Figure 3, the relative stereochemistry of **29a,b** was assumed to be 1’*S*, 1*R*, 2*S* and 1’*S*, 1*S*, 2*S*, respectively. We also examined the reaction of **24a**–**d** in glacial acetic acid according to Wróblewski’s protocol [80] and we did not observe substrate conversion.

In the ^31^P NMR spectra of **29a,b**, two singlets were observed at δ: 18.2 and 17.3, corresponding to the mixture diastereomers (*dr* 1:0.5). Additionally, in the ^13^C NMR spectrum, the doublet of C-α was located at δ 104.5 and 103.9 with large coupling constants of ^1^*J*_CP_ 196 Hz, which is characteristic of *α*-substituted *α*-aminophosphonates [84]. In comparison, the C-*β* signal was observed at δ: 63.4 (d, ^2^*J*_CP_ 15 Hz) and 63.1 (d, ^2^*J*_C-P_ 7 Hz) (see the Supporting Information for details).

## 3. Experimental Section

### 3.1. General Methods

All NMR experiments were performed with a Varian Mercury 300 MHz, Varian VNMR-S 400 MHz, Bruker Ascend™ 400 MHz NANOBA and Bruker Avance 600 MHz spectrometers. Full assignment of all NMR signals and determination of the stereochemistry were possible with the use of various NMR techniques, including ^1^H, ^1^H{/^19^F}, ^1^H{/^31^P}, ^1^H-^1^H COSY, ^1^H-^1^H NOE, ^1^H-^19^F HOE, ^1^H-^13^C HSQC, ^1^H-^13^C HMBC, ^13^C, ^19^F, and ^31^P{/^1^H} experiments. The NMR shifts were determined in relation to the residual solvent proton signal (for CDCl_3_: 7.26 ppm–^1^H NMR, 77.16 ppm–^13^C NMR) and are expressed in parts per million (ppm) in CDCl_3_. Coupling constants (*J*) were reported in hertz (Hz). The following abbreviations were used to express the multiplicities: s–singlet, d–doublet, t–triplet, q–quartet, quint–quintet, dd–doublet of doublets, dt–doublet of triplets, dq–doublet of quartets, td–triplet of doublets, ddd–doublet of doublet of doublets, m–multiplet, br d–broad doublet, br s–broad singlet. ^19^F NMR spectra were measured with trichlorofluoromethane (CFCl_3_) as the internal standard, while for ^31^P NMR spectroscopy, 85% H_3_PO_4_ was used as the external standard. High-resolution mass spectra (HRMS) for the final compounds were performed on an Agilent 6210 ESI using electrospray ionization. Electron ionization mass spectroscopy (EI-MS; low-resolution, direct injection) was performed on a Bruker 320MS/420GC spectrometer.

The obtained compounds were purified by column chromatography using silica gel Merck Kieselgel 60 (230–400 mesh) as the stationary phase, and ethyl acetate/hexane or ethyl acetate/petroleum ether as developing systems. Thin Layer Chromatography (TLC) was performed on commercially available Merck Kieselgel 60-F_254_ with ethyl acetate/hexane as the mobile phase. Visualization of the TLC plates was done using UV light and/or permanganate solution.

Solvents were dried by commonly used methods: toluene was freshly distilled over sodium hydride (NaH_2_) and acetonitrile was distilled over calcium hydride (CaH_2_) prior to use. Anhydrous MeOH and DMF were stored over 4Å molecular sieves. All of the reagents were purchased from Fluorochem^®^, Acros^®^, Alfa Aesar^®^ or Sigma-Aldrich^®^, and used as received.

### 3.2. Theoretical Calculations

Gaussian 16 [85] was used to fully optimize and calculate the frequencies for all the structures at the ωB97x-D/def2-TZVPD level of theory [86,87,88]. The vibrational frequencies were calculated at the same level of theory, and then their positivity was applied to confirm that each of the calculated structures corresponds to a minimum on the potential energy surface. The polarizable continuum model (PCM) [89] was used to simulate solvents: DMF (reaction pathways modelling) and chloroform (conformational analysis of NMR solution). Transition structures were located using the Berny algorithm with the NoEigenTest request. Various combinations of conformations for both invertomers (nitrogen atom) were examined to determine minimum energy pathways for all cyclization reactions.

### 3.3. General Procedure for Synthesis of β-Enaminophosphonates (***2***–***5a,b***)/β-Iminophosphonates (***6***–***9a,b***)

Compounds **2**–**5** and **6**–**9** were synthesized according to a previously reported methodology from diethyl 2-oxo-2-phenylethylphosphonate **1** (151 µL, 0.5 mmol) and primary amine (0.5 mmol) [54]. The reaction mixture was refluxed using a Dean–Stark apparatus. The obtained NMR data based on ^1^H and ^31^P{/^1^H} NMR for **2**–**4a,b** and **6**–**8a,b** are identical with those reported in the literature [54].

### 3.4. The Synthesis of β-Enaminophosphonates/β-Iminophosphonates (***5a***/***9a***,***b***)

Compounds **5a/9a,b** are prepared as described in the general procedure.

The ratio of enamine **5a** and imines **9a,b** was determined with the use of ^1^H and ^31^P{/^1^H} NMR from the crude mixture, and was 1:0.53; the ratio of enamine (Z) **5a** equaled 1, and imines (*E*/*Z*) **9a,b** equaled 1:0.1, respectively. For tautomeric mixture **5a/9a,b** after column chromatography (silica gel, AcOEt/hexane: 5% → 60%), only the NMR spectra of the main products was given (***Z*-5a/*E*-9a** 1:0.53). Yellow oil, 157 mg, yield 87%.

**(*Z*)-Diethyl (2-((4-methoxyphenyl)amino)-2-phenylvinyl)phosphonate (*Z*-5a).**

^1^H NMR (401 MHz): δ = 9.12 (br s, 1H, N*H*), 7.34–7.30 (m, 2H, *H*_ar_), 7.29–7.23 (m, 3H, *H*_ar_), 6.88–6.87 (m, 2H, *H*_ar_), 6.57–6.56 (m, 2H, *H*_ar_), 4.23 (d, *J* = 12.4 Hz, 1H, C*H*P), 4.13–4.05 (m, 4H, 2x OC*H*_2_CH_3_), 3.64 (s, 3H, Ph(4-OC*H*_3_)), 1.32 (td, *J* = 7.1, 0.5 Hz, 6H, OCH_2_C*H*_3_). ^13^C NMR (101 MHz): δ = 161.63 (d, *J* = 6.0 Hz, *C*=CHP), 155.36 (s, *C_ar_*(OCH_3_)), 137.48 (d*, J* = 19.7 Hz, *C_ipso_*), 134.68 (s, *C_ipso_*), 130.63, 129.30, 128.30, 123.69 (4x s, *C*H_ar_), 113.92 (s, *C*H*_ar_*C*_ar_*(OCH_3_)), 82.17 (d, *J* = 188.1 *C*HP), 61.45 (d, *J* = 6.0 Hz, O*C*H_2_CH_3_), 55.39 (s, Ph(4-O*C*H_3_)), 16.20 (d, *J* = 6.7 Hz, OCH_2_*C*H_3_). ^31^P{/^1^H} NMR (122 MHz): δ = 24.73 (s, 1P). MS (EI) m/z = 361.2 [M]^+^.

**(*E*)-Diethyl (2-((4-methoxyphenyl)imino)-2-phenylethyl)phosphonate (*E*-9a).**

^1^H NMR (401 MHz): δ = 7.43–7.39 (m, 3H, *H*_ar_), 7.25–7.19 (m, 2H, *H*_ar_), 6.88–6.87 (m, 2H, *H*_ar_), 6.57–6.56 (m, 2H, *H*_ar_), 4.05–4.00 (m, 2H, OC*H*_2_CH_3_), 3.92–3.84 (m, 2H, OC*H*_2_CH_3_), 3.78 (s, 3H, Ph(4-OC*H*_3_)), 3.39 (d, *J* = 23.3 Hz, 2H, C*H*_2_P), 1.10 (td, *J* = 7.1, 0.5 Hz, 6H, OCH_2_C*H*_3_). ^13^C NMR (101 MHz): δ = 160.07 (d, *J* = 7.8 Hz, *C*=N), 156.39 (s, *C_ar_*(OCH_3_)), 143.97 (d*, J* = 2.3 Hz, *C_ipso_*), 138.76 (s, *C_ipso_*), 128.32, 128.29, 128.06, 120.83 (4x s, *C*H_ar_), 114.40 (s, *C*H*_ar_*C*_ar_*(OCH_3_)), 62.22 (d, *J* = 6.6 Hz, O*C*H_2_CH_3_), 55.55 (s, Ph(4-O*C*H_3_)), 29.51 (d, *J* = 134.3 Hz, *C*H_2_P), 16.46 (d, *J* = 6.3 Hz, OCH_2_*C*H_3_). ^31^P{/^1^H} NMR (122 MHz): δ = 22.12 (s, 1P).

**(*Z*)-Diethyl (2-((4-methoxyphenyl)imino)-2-phenylethyl)phosphonate (*Z*-9b).**

Diagnostic signals: ^31^P{/^1^H} NMR (122 MHz): δ = 24.00 (s).

### 3.5. General Procedure for Synthesis of α,α-Bromofluorinated ß-Iminophosphonates (***10***–***11a***,***b***, rac-***12***–***13***)

The solution of Selectfluor (319 mg, 0.9 mmol) in dry, freshly distilled acetonitrile (10 mL) was gently heated to 50 °C and vigorously stirred until the compound was completely dissolved. After, the solution was cooled to ambient temperature and added together with NBS (89 mg, 0.5 mmol) to the mixture of the appropriate *β*-enaminophosphonate/*β*-iminophosphonate **2**–**5**/**6**–**9** (0.5 mmol). After 15 min of stirring at room temperature, the solvent was removed under reduced pressure. Next, the residue was dissolved in CHCl_3_ (2 mL), water (10 mL) was added, and it was extracted (3 × 10 mL CHCl_3_). The organic layers were combined, dried over Na_2_SO_4_, and evaporated to give products as a pale-yellow oil. The crude products were purified by column chromatography (AcOEt/petroleum ether: 5% → 50%).

**(*E*)-Diethyl ((1*R*)-1-bromo-1-fluoro-2-phenyl-2-(((*S*)-1-phenylethyl)imino)ethyl) phosphonate (10a) and (*E*)-diethyl ((1*S*)-1-bromo-1-fluoro-2-phenyl-2-(((*S*)-1-phenylethyl)imino)ethyl) phosphonate (10b).**

Pale-yellow oil, 210 mg, yield 92%. Isolated as a mixture of diastereomers **10a,b** (*dr* 1:0.54) (153 mg) and single diastereomer **10b** (57mg).

**10a**: ^1^H NMR (400 MHz): δ = 7.44–7.40 (m, 1H, *H*_ar_), 7.39–7.36 (m, 2H, *H*_ar_), 7.33–7.30 (m, 2H, *H*_ar_), 7.29–7.26 (m, 3H, *H*_ar_), 7.25–7.20 (m, 2H, *H*_ar_), 4.47–4.35 (m, 3H, OC*H*_2_CH_3_, C*H*CH_3_), 4.34–4.23 (m, 2H, OC*H*_2_CH_3_), 1.38 (d, *J* = 6.6 Hz, 3H, CHC*H*_3_), 1.35 (td, *J* = 7.0, 0.8 Hz, 3H, OCH_2_C*H*_3_), 1.32 (td, *J* = 7.1, 0.9 Hz, 3H, OCH_2_C*H*_3_). ^13^C NMR (101 MHz): δ = 164.44 (dd, *J* = 27.0, 6.0 Hz, *C*=N), 144.57 (s, *C_ipso_*), 131.52 (d, *J* = 5.0 Hz, *C_ipso_*), 128.60, 128.58, 127.87, 126.97, 126.62, 126.47 (6x s, *C*H_ar_), 100.05 (dd, *J* = 269.8, 187.5 Hz, *C*BrF), 65.52 (d, *J* = 6.2 Hz, O*C*H_2_CH_3_), 65.20 (d, *J* = 6.7 Hz, O*C*H_2_CH_3_), 61.19 (s, *C*HCH_3_), 24.54 (s, CH*C*H_3_), 16.64 (d, *J* = 6.1 Hz, OCH_2_*C*H_3_), 16.61 (d, *J* = 6.1 Hz, OCH_2_*C*H_3_). ^19^F NMR (283 MHz): δ = −129.08 (d, *J* = 82.2 Hz, 1F). ^31^P{/^1^H} NMR (122 MHz): δ = 7.37 (d, *J* = 82.1 Hz, 1P). IR (neat): 1648, 1261, 1012, 972, 649 [cm^−1^]. MS (EI) m/z = 457.3 [M+H]^+^.

**10b:** ^1^H NMR (400 MHz): δ = 7.42–7.37 (m, 3H, *H*_ar_), 7.31–7.28 (m, 2H, *H*_ar_), 7.30–7.24 (m, 4H, *H*_ar_), 7.20–7.16 (m, 1H, *H*_ar_), 4.42 (q, *J* = 6.5 Hz, 1H, C*H*CH_3_), 4.39–4.26 (m, 2H, OC*H*_2_CH_3_), 4.25–4.05 (m, 2H, OC*H*_2_CH_3_), 1.40 (d, *J* = 6.5 Hz, 3H, CHC*H*_3_), 1.30 (td, *J* = 7.1, 0.9 Hz, 3H, OCH_2_C*H*_3_), 1.22 (td, *J* = 7.1, 0.8 Hz, 3H, OCH_2_C*H*_3_). ^13^C NMR (101 MHz): δ = 164.64 (dd, *J* = 28.3, 5.9 Hz, *C*=N), 144.15 (s, *C_ipso_*), 131.70 (d, *J* = 5.1 Hz, *C_ipso_*), 128.50, 128.43, 128.41, 128.33, 126.99, 126.72 (6x s, *C*H_ar_), 99.78 (dd, *J* = 269.3, 187.2 Hz, *C*BrF), 65.51 (d, *J* = 6.3 Hz, O*C*H_2_CH_3_), 65.05 (d, *J* = 6.6 Hz, O*C*H_2_CH_3_), 61.40 (s, *C*HCH_3_), 23.97 (s, CH*C*H_3_), 16.56 (d, *J* = 6.1 Hz, OCH_2_*C*H_3_), 16.44 (d, *J* = 6.1 Hz, OCH_2_*C*H_3_). ^19^F NMR (283 MHz): δ = −128.65 (d, *J* = 82.3 Hz, 1F). ^31^P{/^1^H} NMR (122 MHz): δ = 7.93 (d, *J* = 82.3 Hz, 1P).

**(*E*)-Diethyl ((1*S*)-1-bromo-1-fluoro-2-phenyl-2-(((*R*)-1-phenylethyl)imino)ethyl) phosphonate (11a) and (*E*)-Diethyl ((1*R*)-1-bromo-1-fluoro-2-phenyl-2-(((*R*)-1-phenylethyl)imino)ethyl)phosphonate (11b).**

Pale-yellow oil, 217 mg, yield 95%. Isolated as a mixture of diastereomers **11a,b** (*dr* 1:0.93), which could not be separated by the chromatography techniques employed in this study.

**11a**: ^1^H NMR (400 MHz): δ = 7.43–7.37 (m, 5H, *H*_ar_), 7.26–7.21 (m, 5H, *H*_ar_), 4.42–4.35 (m, 3H, OC*H*_2_CH_3_, C*H*CH_3_), 4.34–4.25 (m, 2H, OC*H*_2_CH_3_), 1.39 (d, *J* = 6.5 Hz, 3H, CHC*H*_3_), 1.36 (td, *J* = 7.1, 0.8 Hz, 3H, OCH_2_C*H*_3_), 1.33 (td, *J* = 7.1, 0.9 Hz, 3H, OCH_2_C*H*_3_). ^13^C NMR (101 MHz): δ = 164.45 (dd, *J* = 27.1, 6.1 Hz, *C*=N), 144.53 (s, *C_ipso_*), 131.52 (d, *J* = 5.1 Hz, *C_ipso_*), 129.52, 128.60, 128.45, 126.96, 126.69, 126.43 (6x s, *C*H_ar_), 100.99 (dd, *J* = 269.7, 187.4 Hz, *C*BrF), 65.45 (d, *J* = 6.1 Hz, O*C*H_2_CH_3_), 65.14 (d, *J* = 6.7 Hz, O*C*H_2_CH_3_), 61.17 (s, *C*HCH_3_), 24.49 (s, CH*C*H_3_), 16.56 (d, *J* = 6.0 Hz, OCH_2_*C*H_3_), 16.45 (d, *J* = 6.1 Hz, OCH_2_*C*H_3_). ^19^F NMR (283 MHz): δ = −128.52 (d, *J* = 82.1 Hz, 1F). ^31^P{/^1^H} NMR (122 MHz): δ = 7.38 (d, *J* = 82.1 Hz, 1P). IR (neat): 1644, 1262, 1017, 970, 647 [cm^−1^]. MS (EI) m/z = 457.3 [M+H]^+^.

**11b**: ^1^H NMR (400 MHz): δ = 7.32–7.27 (m, 5H, *H*_ar_), 7.23–7.16 (m, 5H, *H*_ar_), 4.43 (q, *J* = 6.5 Hz, 1H, C*H*CH_3_), 4.27–4.17 (m, 2H, OC*H*_2_CH_3_), 4.16–4.08 (m, 2H, OC*H*_2_CH_3_), 1.41 (d, *J* = 6.5 Hz, 3H, CHC*H*_3_), 1.31 (td, *J* = 7.1, 0.8 Hz, 3H, OCH_2_C*H*_3_), 1.23 (td, *J* = 7.1 0.8 Hz, 3H, OCH_2_C*H*_3_). ^13^C NMR (101 MHz): δ = 164.63 (dd, *J* = 28.4, 5.9 Hz, *C*=N), 144.12 (s, *C_ipso_*), 131.69 (d, *J* = 5.1 Hz, *C_ipso_*), 128.55, 128.41, 128.36, 128.29, 127.01, 126.93 (6x s, *C*H_ar_), 99.14 (dd, *J* = 269.1, 187.0 Hz, *C*BrF), 65.42 (d, *J* = 6.2 Hz, O*C*H_2_CH_3_), 65.00 (d, *J* = 6.6 Hz, O*C*H_2_CH_3_), 61.37 (s, *C*HCH_3_), 23.92 (s, CH*C*H_3_), 16.53 (d, *J* = 6.1 Hz, OCH_2_*C*H_3_), 16.41 (d, *J* = 6.0 Hz, OCH_2_*C*H_3_). ^19^F NMR (283 MHz): δ = −128.13 (d, *J* = 82.3 Hz, 1F). ^31^P{/^1^H} NMR (122 MHz): δ = 7.42 (d, *J* = 82.4 Hz, 1P).

**rac-(*E*)-Diethyl ((1*R*/1*S*)-1-bromo-1-fluoro-2-((4-methoxybenzyl)imino)-2-phenylethyl)phosphonate (rac-12).**

Pale-yellow oil, 222 mg, yield 94%.

^1^H NMR (400 MHz): δ = 7.45–7.42 (m, 3H, *H*_ar_), 7.34–7.30 (m, 2H, *H*_ar_), 7.22–7.15 (m, 2H, *H*_ar_), 6.85–6.80 (m, 2H, *H*_ar_), 4.46 (br s, C*H*_2_N, 2H), 4.33–4.24 (m, 2H, OC*H*_2_CH_3_), 4.23–4.10 (m, 2H, OC*H*_2_CH_3_), 3.76 (s, 3H, Ph(4-OC*H*_3_)), 1.26 (td, *J* = 7.1, 0.9 Hz, 3H, OCH_2_C*H*_3_), 1.25 (td, *J* = 7.1, 0.8 Hz, 3H, OCH_2_C*H*_3_). ^13^C NMR (101 MHz): δ = 166.67 (dd, *J* = 27.7, 6.1 Hz, *C*=N), 158.67 (s, *C_ar_*(OCH_3_)), 131.44 (d, *J* = 5.2 Hz, *C_ipso_*), 130.90 (s, *C_ipso_*), 129.70, 128.93 (2x s, *C*H_ar_), 128.60 (d, *J* = 1.5 Hz, *C*H_ar_), 128.50 (s, *C*H_ar_), 113.93 (s, *C*H*_ar_*C*_ar_*(OCH_3_)), 99.97 (dd, *J* = 269.7, 187.6 Hz, *C*BrF), 65.54 (d, *J* = 6.3 Hz, O*C*H_2_CH_3_), 65.15 (d, *J* = 6.7 Hz, O*C*H_2_CH_3_), 56.42 (br s, *C*H_2_N), 55.39 (s, Ph(4-O*C*H_3_)), 16.49 (d, *J* = 6.1 Hz, OCH_2_*C*H_3_), 16.48 (d, *J* = 6.0 Hz, OCH_2_*C*H_3_). ^19^F NMR (283 MHz): δ = −129.07 (d, *J* = 81.2 Hz, 1F). ^31^P{/^1^H} NMR (122 MHz): δ = 7.98 (d, *J* = 81.4 Hz, 1P). IR (neat): 1645, 1261, 1247, 1013, 970, 647 [cm^−1^]. MS (EI) m/z = 392.2 [M-Br]^+^.

**rac-(*E*)-Diethyl ((1*R*/1*S*)-1-bromo-1-fluoro-2-((4-methoxyphenyl)imino)-2-phenylethyl)phosphonate (rac-13).**

Brown oil, 200 mg, yield 87%.

^1^H NMR (400 MHz): δ = 7.31–7.26 (m, 5H, *H*_ar_), 6.68–6.64 (m, 4H, *H*_ar_), 4.41–4.30 (m, 4H, 2x OC*H*_2_CH_3_), 3.68 (s, 3H, Ph(4-OC*H*_3_)), 1.36 (td, *J* = 7.1, 0.9 Hz, 3H, OCH_2_C*H*_3_), 1.35 (td, *J* = 7.1, 0.9 Hz, 3H, OCH_2_C*H*_3_). ^13^C NMR (101 MHz): δ = 164.12 (dd, *J* = 28.4, 5.9 Hz, *C*=N), 157.44 (s, *C_ar_*(OCH_3_)), 140.26 (s, *C_ipso_*), 131.94 (d, *J* = 5.0 Hz *C_ipso_*), 129.56, 129.43, 123.19, 118.90 (4x s, *C*H_ar_), 113.90 (s, *C*H*_ar_*C*_ar_*(OCH_3_)), 100.17 (dd, *J* = 270.1, 187.2 Hz, *C*BrF), 65.58 (d, *J* = 6.4 Hz, O*C*H_2_CH_3_), 65.29 (d, *J* = 6.6 Hz, O*C*H_2_CH_3_), 55.35 (s, Ph(4-O*C*H_3_)), 16.56 (d, *J* = 5.9 Hz, OCH_2_*C*H_3_), 16.55 (d, *J* = 6.0 Hz, OCH_2_*C*H_3_). ^19^F NMR (283 MHz): δ = −128.49 (d, *J* = 81.7 Hz, 1F). ^31^P{/^1^H} NMR (122 MHz): δ = 7.30 (d, *J* = 81.5 Hz, 1P). IR (neat): 1645, 1262, 1246, 1012, 972, 645 [cm^−1^]. MS (EI) m/z = 459.3 [M+H]^+^.

### 3.6. General Procedure for Synthesis of α,α-Chlorofluorinated ß-Iminophosphonates (***14a***,***b***, rac-***15***)

Compounds **14a,b** and rac-**15** were obtained according to the above-described procedure for bromofluorinated iminophosphonates (**10**–**11a,b**, rac-**12**–**13**). Selectfluor and *N*-chlorosuccinimide (NCS) were used as halogenation reagents with appropriate molar equivalents equaled 1.35 and 1.0, respectively.

**(*E*)-Diethyl ((1*R*)-1-chloro-1-fluoro-2-phenyl-2-(((*S*)-1-phenylethyl)imino)ethyl)phosphonate (14a) and (*E*)-diethyl ((1*S*)-1-chloro-1-fluoro-2-phenyl-2-(((*S*)-1-phenylethyl)imino)ethyl)phosphonate (14b)**

Pale-yellow oil, 128 mg, yield 62%. Isolated as a mixture of diastereomers **14a,b** (*dr* 0.2:1). The rest of diastereomer **14a** (65 mg) was contaminated with difluoro- and dichloroiminophosphonate derivatives.

**14a**: ^1^H NMR (400 MHz): δ = 7.45–7.41 (m, 3H, *H*_ar_), 7.32–7.27 (m, 4H, *H*_ar_), 7.25–7.21 (m, 3H, *H*_ar_), 4.49 (q, *J* = 6.5 Hz, 1H, C*H*CH_3_), 4.38–4.32 (m, 2H, OC*H*_2_CH_3_), 4.29–4.25 (m, 2H, OC*H*_2_CH_3_), 1.42 (d, *J* = 6.5 Hz, 3H, CHC*H*_3_), 1.34 (td, *J* = 7.1, 0.8 Hz, 3H, OCH_2_C*H*_3_), 1.32 (td, *J* = 7.1, 0.8 Hz, 3H, OCH_2_C*H*_3_). ^13^C NMR (101 MHz): δ = 163.55 (dd, *J* = 28.1, 6.5 Hz, *C*=N), 144.46 (s, *C_ipso_*), 131.51 (d, *J* = 4.7 Hz, *C_ipso_*), 128.58, 128.55, 128.43, 128.02, 127.08, 126.58 (6x s, *C*H_ar_), 105.89 (dd, *J* = 260.7, 189.9 Hz, *C*ClF), 65.11 (d, *J* = 6.5 Hz, O*C*H_2_CH_3_), 64.78 (d, *J* = 6.5 Hz, O*C*H_2_CH_3_), 61.33 (s, *C*HCH_3_), 24.51 (s, CH*C*H_3_), 16.52 (d, *J* = 6.1 Hz, OCH_2_*C*H_3_), 16.48 (d, *J* = 6.0 Hz, OCH_2_*C*H_3_). ^19^F NMR (283 MHz): δ = −125.59 (d, *J* = 86.2 Hz, 1F). ^31^P{/^1^H} NMR (122 MHz): δ = 8.02 (d, *J* = 86.0 Hz, 1P). IR (neat)**:** 1657, 1262, 1011, 969, 666 [cm^−1^]. MS (EI) m/z = 376.2 [M-Cl]^+^.

**14b**: ^1^H NMR (400 MHz): δ = 7.43–7.37 (m, 3H, *H*_ar_), 7.31–7.26 (m, 4H, *H*_ar_), 7.25–7.22 (m, 3H, *H*_ar_), 4.46 (q, *J* = 6.5 Hz, 1H, C*H*CH_3_), 4.39–4.23 (m, 3H, OC*H*_2_CH_3_, OC*H*HCH_3_), 4.22–4.12 (m, 1H, OCH*H*CH_3_), 1.44 (d, *J* = 6.5 Hz, 3H, CHC*H*_3_), 1.31 (td, *J* = 7.1, 0.9 Hz, 3H, OCH_2_C*H*_3_), 1.24 (td, *J* = 7.1, 0.8 Hz, 3H, OCH_2_C*H*_3_). ^13^C NMR (101 MHz): δ = 163.75 (dd, *J* = 28.6, 6.8 Hz, *C*=N), 144.16 (s, *C_ipso_*), 131.64 (d, *J* = 4.8 Hz, *C_ipso_*), 129.56, 129.53, 128.50, 128.39, 126.99, 126.68 (6x s, *C*H_ar_), 105.77 (dd, *J* = 259.9, 193.0 Hz, *C*ClF), 65.35 (d, *J* = 6.5 Hz, O*C*H_2_CH_3_), 64.96 (d, *J* = 6.5 Hz, O*C*H_2_CH_3_), 61.45 (br s, *C*HCH_3_), 24.16 (s, CH*C*H_3_), 16.53 (d, *J* = 6.0 Hz, OCH_2_*C*H_3_), 16.42 (d, *J* = 5.9 Hz, OCH_2_*C*H_3_). ^19^F NMR (283 MHz): δ = −125.39 (d, *J* = 87.0 Hz, 1F). ^31^P{/^1^H} NMR (122 MHz): δ = 7.54 (d, *J* = 87.0 Hz, 1P).

**rac- (E)-Diethyl (1R/1S)-1-chloro-1-fluoro-2-((4-methoxybenzyl)imino)-2-phenylethyl)phosphonate (rac-15).**

Pale-yellow oil, 195 mg, yield 91%.

^1^H NMR (400 MHz): δ = 7.43–7.39 (m, 3H, *H*_ar_), 7.33–7.27 (m, 2H, *H*_ar_), 7.19–7.14 (m, 2H, *H*_ar_), 6.84–6.78 (m, 2H, *H*_ar_), 4.46 (br s, 2H, C*H*_2_N), 4.32–4.23 (m, 2H, OC*H*_2_CH_3_), 4.21–4.15 (m, 2H, OC*H*_2_CH_3_), 3.75 (s, 3H, Ph(4-OC*H*_3_)), 1.26 (td, *J* = 7.1, 0.9 Hz, 3H, OCH_2_C*H*_3_), 1.24 (td, *J* = 7.1, 0.8 Hz, 3H, OCH_2_C*H*_3_). ^13^C NMR (101 MHz): δ = 165.78 (dd, *J* = 27.9, 6.7 Hz, *C*=N), 158.68 (s, *C_ar_*(OCH_3_)), 131.35 (d, *J* = 4.9 Hz *C_ipso_*), 129.18, 129.15, 128.93, 128.51 (4x s, *C*H_ar_), 128.43 (d, *J* = 1.3 Hz, *C*H_ar_), 128.25 (s, *C*H_ar_), 113.91 (s, *C*H*_ar_*C*_ar_*(OCH_3_)), 105.94 (dd, *J* = 260.5, 193.5 Hz, *C*ClF), 65.55 (d, *J* = 6.9 Hz, O*C*H_2_CH_3_), 65.10 (d, *J* = 6.6 Hz, O*C*H_2_CH_3_), 56.46 (br s, *C*H_2_N), 55.36 (s, Ph(4-O*C*H_3_)), 16.48 (d, *J* = 6.0 Hz, OCH_2_*C*H_3_), 16.45 (d, *J* = 5.8 Hz, OCH_2_*C*H_3_). ^19^F NMR (283 MHz): δ = −125.57 (d, *J* = 85.6 Hz, 1F). ^31^P{/^1^H} NMR (122 MHz): δ = 8.09 (d, *J* = 86.0 Hz, 1P). IR (neat): 1648, 1260, 1014, 970, 668 [cm^−1^]. MS (EI) m/z = 428.8 [M+H]^+^.

### 3.7. General Procedure for Synthesis of α,α-Halofluorinated ß-Aminophosphonates (***16***–***17***,***20a***–***d***, rac-***18***–***19***,***21a***,***b***)

To the stirred solution of *β*-iminophosphonate (**10**–**11,14a,b**, rac-**12**–**13**,**15**) (0.5 mmol) in MeOH (3 mL), NaBH_3_CN (188 mg, 3 mmol) and glacial CH_3_COOH (171 µL, 180 mg, 3 mmol) at room temperature were added. Stirring was continued for 40 min and then the solvent was evaporated. Next, the residue was dissolved in CHCl_3_ (2 mL), water (10 mL) was added, and the inorganic layer was extracted (3 × 10 mL CHCl_3_). The organic layers were combined, dried over Na_2_SO_4_, and evaporated to give the product as a pale-yellow oil. The crude products were purified by column chromatography (AcOEt/hexane: 5% → 60%).

**Diethyl ((1*S*/*R*, 2*S*/*R*)-1-bromo-1-fluoro-2-phenyl-2-(((*S*)-1-phenylethyl)amino)ethyl)phosphonate (16a-d).**

Isolated as a mixture of two major diastereomers **16a,b** (*dr* 1:0.96). Diagnostic signals for traces of diastereomers **16c,d** were determined from the crude reaction mixture. Pale-yellow oil, 222 mg, yield 97%.

**Diethyl ((1*R*, 2*R*)-1-bromo-1-fluoro-2-phenyl-2-(((*S*)-1-phenylethyl)amino)ethyl)phosphonate (16a).**

^1^H NMR (400 MHz): δ = 7.36–7.33 (m, 5H, *H*_ar_), 7.28–7.25 (m, 2H, *H*_ar_), 7.21–7.19 (m, 3H, *H*_ar_), 4.58 (dd, *J* = 10.2, 4.7 Hz, 1H, C*H*(Ph)CF), 4.40–4.29 (m, 2H, OC*H*_2_CH_3_), 4.27–4.23 (m, 2H, OC*H*_2_CH_3_), 3.75 (q, *J* = 6.4 Hz, 1H, C*H*CH_3_), 1.34 (d, *J* = 6.4 Hz, 3H, CHC*H*_3_), 1.28 (td, *J* = 7.1, 0.8 Hz, 3H, OCH_2_C*H*_3_), 1.27 (td, *J* = 7.1, 0.8 Hz, 3H, OCH_2_C*H*_3_). ^13^C NMR (101 MHz): δ = 145.46 (s, *C_ipso_*), 135.70 (d, *J* = 8.6 Hz, *C_ipso_*), 128.54, 128.50, 128.41, 128.06, 127.24, 126.91 (6x s, *C*H_ar_), 107.44 (dd, *J* = 270.1, 184.3 Hz, *C*BrF), 65.58 (d, *J* = 7.3 Hz, O*C*H_2_CH_3_), 65.16 (d, *J* = 7.3 Hz, O*C*H_2_CH_3_), 64.12 (dd, *J* = 22.0, 7.5 Hz, *C*H(Ph)CF), 54.71 (s, *C*HCH_3_), 21.89 (s, CH*C*H_3_), 16.60 (d, *J* = 6.1 Hz, OCH_2_*C*H_3_), 16.44 (d, *J* = 6.2 Hz, OCH_2_*C*H_3_). ^19^F NMR (283 MHz): δ = −125.06 (dd, *J* = 82.3, 10.2 Hz, 1F). ^31^P{/^1^H} NMR (122 MHz): δ = 9.77 (d, *J* = 82.0 Hz, 1P). IR (neat): 1264, 1024, 977, 648 [cm^−1^]. MS (EI) m/z = 459.4 [M+H]^+^.

**Diethyl ((1*S*, 2*R*)-1-bromo-1-fluoro-2-phenyl-2-(((*S*)-1-phenylethyl)amino)ethyl)phosphonate (16b).**

^1^H NMR (400 MHz): δ = 7.33–7.29 (m, 5H, *H*_ar_), 7.26–7.24 (m, 1H, *H*_ar_), 7.23–7.21 (m, 2H, *H*_ar_), 7.18–7.16 (m, 2H, *H*_ar_), 4.37 (dd, *J* = 22.0, 3.5 Hz, 1H, C*H*(Ph)CF), 4.22–4.17 (m, 2H, OC*H*_2_CH_3_), 4.17–4.07 (m, 2H, OC*H*_2_CH_3_), 3.74 (q, *J* = 6.5 Hz, 1H, C*H*CH_3_), 1.35 (d, *J* = 6.4 Hz, 3H, CHC*H*_3_), 1.26 (td, *J* = 7.1, 0.8 Hz, 3H, OCH_2_C*H*_3_), 1.25 (td, *J* = 7.1, 0.8 Hz, 3H, OCH_2_C*H*_3_). ^13^C NMR (101 MHz): δ = 145.67 (s, *C_ipso_*), 137.14 (dd, *J* = 6.0, 2.5 Hz, *C_ipso_*), 128.44, 128.40, 128.13, 127.64, 127.15, 126.84 (6x s, *C*H_ar_), 107.62 (dd, *J* = 274.2, 183.1 Hz, *C*BrF), 66.47 (dd, *J* = 18.2, 7.9 Hz, *C*H(Ph)CF), 64.88 (d, *J* = 7.2 Hz, O*C*H_2_CH_3_), 64.74 (d, *J* = 7.2 Hz, O*C*H_2_CH_3_), 55.67 (s, *C*HCH_3_), 22.10 (s, CH*C*H_3_), 16.52 (d, *J* = 6.1 Hz, OCH_2_*C*H_3_), 16.42 (d, *J* = 6.1 Hz, OCH_2_*C*H_3_). ^19^F NMR (283 MHz): δ = −135.38 (dd, *J* = 84.6, 22.1 Hz, 1F). ^31^P{/^1^H} NMR (122 MHz): δ = 9.25 (d, *J* = 84.7 Hz, 1P).

**Diethyl ((1*S*, 2*S*)-1-bromo-1-fluoro-2-phenyl-2-(((*S*)-1-phenylethyl)amino)ethyl)phosphonate (16c).**

Diagnostic signals: ^19^F NMR (283 MHz): δ = −128.26 (dd, *J* = 78.0, 10.2 Hz). ^31^P{/^1^H} NMR (122 MHz): δ = 9.06 (d, *J* = 77.9 Hz).

**Diethyl ((1*R*, 2*S*)-1-bromo-1-fluoro-2-phenyl-2-(((*S*)-1-phenylethyl)amino)ethyl)phosphonate (16d).**

Diagnostic signals: ^19^F NMR (283 MHz): δ = −135.49 (dd, *J* = 88.4, 20.9 Hz). ^31^P{/^1^H} NMR (122 MHz): signal masked by other diastereomers signals.

**Diethyl ((1*S*/1*R*, 2*S*/2*R*)-1-bromo-1-fluoro-2-phenyl-2-(((*R*)-1-phenylethyl)amino)ethyl)phosphonate (17a-d).**

Isolated as a mixture of two major diastereomers **17a,b** (*dr* 1:0.8). Diagnostic signals for traces of diastereomers **17c,d** were determined from the crude reaction mixture. Pale-yellow oil, 217 mg, yield 95%.

**Diethyl ((1*S*, 2*S*)-1-bromo-1-fluoro-2-phenyl-2-(((*R*)-1-phenylethyl)amino)ethyl)phosphonate (17a).**

^1^H NMR (400 MHz): δ = 7.35–7.33 (m, 5H, *H*_ar_), 7.28–7.26 (m, 2H, *H*_ar_), 7.22–7.19 (m, 3H, *H*_ar_), 4.58 (dd, *J* = 10.2, 4.7 Hz, 1H, C*H*(Ph)CF), 4.36–4.32 (m, 2H, OC*H*_2_CH_3_), 4.26–4.23 (m, 2H, OC*H*_2_CH_3_), 3.75 (q, *J* = 6.4 Hz, 1H, C*H*CH_3_), 1.35 (td, *J* = 7.1, 0.8 Hz, 3H, OCH_2_C*H*_3_), 1.32 (d, *J* = 6.4 Hz, 3H, CHC*H*_3_), 1.31 (td, *J* = 7.1, 0.8 Hz, 3H, OCH_2_C*H*_3_). ^13^C NMR (101 MHz): δ = 145.41 (s, *C_ipso_*), 135.63 (d, *J* = 8.6 Hz, *C_ipso_*), 128.52, 128.49, 128.42, 128.04, 127.22, 126.81 (6x s, *C*H_ar_), 107.39 (dd, *J* = 270.1, 184.4 Hz, *C*BrF), 65.56 (d, *J* = 7.0 Hz, O*C*H_2_CH_3_), 65.15 (d, *J* = 7.3 Hz, O*C*H_2_CH_3_), 64.05 (dd, *J* = 21.9, 7.5 Hz, *C*H(Ph)CF), 54.65 (s, *C*HCH_3_), 21.85 (s, CH*C*H_3_), 16.59 (d, *J* = 6.0 Hz, OCH_2_*C*H_3_), 16.43 (d, *J* = 6.1 Hz, OCH_2_*C*H_3_). ^19^F NMR (283 MHz): δ = −125.04 (dd, *J* = 82.1, 10.1 Hz, 1F). ^31^P{/^1^H} NMR (122 MHz): δ = 9.25 (d, *J* = 82.0 Hz, 1P). IR (neat): 1262, 1021, 978, 646 [cm^−1^]. MS (EI) m/z = 459.4 [M-H]^+^.

**Diethyl ((1*R*, 2*S*)-1-bromo-1-fluoro-2-phenyl-2-(((*R*)-1-phenylethyl)amino)ethyl)phosphonate (17b).**

^1^H NMR (400 MHz): δ = 7.32–7.29 (m, 5H, *H*_ar_), 7.26–7.24 (m, 2H, *H*_ar_), 7.19–7.16 (m, 3H, *H*_ar_), 4.42–4.37 (m, 2H, OC*H*_2_CH_3_), 4.34 (dd, *J* = 22.1, 3.2 Hz, 1H, C*H*(Ph)CF), 4.23–4.15 (m, 2H, OC*H*_2_CH_3_), 3.74 (q, *J* = 6.5 Hz, 1H, C*H*CH_3_), 1.33 (d, *J* = 6.4 Hz, 3H, CHC*H*_3_), 1.27 (td, *J* = 7.1, 0.8 Hz, 3H, OCH_2_C*H*_3_), 1.26 (td, *J* = 7.1, 0.8 Hz, 3H, OCH_2_C*H*_3_). ^13^C NMR (101 MHz): δ = 145.62 (s, *C_ipso_*), 137.08 (dd, *J* = 6.2, 2.7 Hz, *C_ipso_*), 128.45, 128.40, 128.31, 128.11, 127.13, 126.88 (6x s, *C*H_ar_), 107.57 (dd, *J* = 274.0, 182.8 Hz, *C*BrF), 66.42 (dd, *J* = 18.2, 8.0 Hz, *C*H(Ph)CF), 64.85 (d, *J* = 7.2 Hz, O*C*H_2_CH_3_), 64.72 (d, *J* = 7.1 Hz, O*C*H_2_CH_3_), 55.63 (s, *C*HCH_3_), 22.06 (s, CH*C*H_3_), 16.51 (d, *J* = 6.1 Hz, OCH_2_*C*H_3_), 16.42 (d, *J* = 6.1 Hz, OCH_2_*C*H_3_). ^19^F NMR (283 MHz): δ = −135.36 (dd, *J* = 84.6, 22.0 Hz, 1F). ^31^P{/^1^H} NMR (122 MHz): δ = 8.73 (d, *J* = 84.6 Hz, 1P).

**Diethyl((1*R*, 2*R*)-1-bromo-1-fluoro-2-phenyl-2-(((*R*)-1-phenylethyl)amino)ethyl)phosphonate (17c).**

Diagnostic signals ^19^F NMR (283 MHz): δ = −128.25 (dd, *J* = 77.9, 10.2 Hz). ^31^P{/^1^H} NMR (122 MHz): δ = 8.56 (d, *J* = 78.0 Hz).

**Diethyl((1*R*, 2*S*)-1-bromo-1-fluoro-2-phenyl-2-(((*R*)-1-phenylethyl)amino)ethyl)phosphonate (17d).**

^19^F NMR (283 MHz): signal masked by other diastereomers signals. Diagnostic signals ^31^P{/^1^H} NMR (122 MHz): δ = 8.40 (d, *J* = 86.9 Hz).

**rac-Diethyl ((1*R*, 2*R*)-1-bromo-1-fluoro-2-((4-methoxybenzyl)amino)-2-phenylethyl)phosphonate (rac-18a) and rac-diethyl ((1*R*, 2*S*)-1-bromo-1-fluoro-2-((4-methoxybenzyl)amino)-2-phenylethyl)phosphonate (rac-18b).**

Isolated as a mixture of two diastereomers rac-**18a,b** (*dr* 1:0.75), which could not be separated by the chromatography techniques employed in this study. Pale-yellow oil, 230 mg, yield 97%.

rac-**18a**: ^1^H NMR (400 MHz): δ = 7.46–7.44 (m, 3H, *H*_ar_), 7.38–7.36 (m, 2H, *H*_ar_), 7.14–7.10 (m, 2H, *H*_ar_), 6.83–6.81 (m, 2H, *H*_ar_), 4.37–4.26 (m, 3H, OC*H*_2_CH_3_, C*H*(Ph)CFP), 4.20–4.15 (m, 2H, OC*H*_2_CH_3_), 3.76 (s, 3H, Ph(4-OC*H*_3_)), 3.72 (br d, *J* = 12.9 Hz, 1H, C*H*HN), 3.48 (br d, *J* = 13.1 Hz, 1H, CH*H*N), 1.30 (td, *J* = 7.1, 0.9 Hz, 3H, OCH_2_C*H*_3_), 1.22 (td, *J* = 7.0, 0.8 Hz, 3H, OCH_2_C*H*_3_). ^13^C NMR (101 MHz): δ = 158.84 (s, *C_ar_*(OCH_3_)), 135.13 (d, *J* = 8.5 Hz, *C_ipso_*), 131.52 (s, *C_ipso_*), 129.85 (d, *J* = 1.5 Hz, *C*H_ar_), 129.71, 128.65, 128.12 (3x s, *C*H_ar_), 113.78 (s, *C*H*_ar_*C*_ar_*(OCH_3_)), 106.46 (dd, *J* = 270.2, 183.8 Hz, *C*BrF), 65.79 (d, *J* = 7.0 Hz, O*C*H_2_CH_3_), 65.24 (dd, *J* = 21.5, 7.9 Hz, *C*H(Ph)CF), 65.03 (d, *J* = 7.1 Hz, O*C*H_2_CH_3_), 55.32 (s, Ph(4-O*C*H_3_)), 50.02 (s, *C*H_2_N), 16.48 (d, *J* = 5.9 Hz, OCH_2_*C*H_3_), 16.33 (d, *J* = 5.9 Hz, OCH_2_*C*H_3_). ^19^F NMR (283 MHz): δ = −126.81 (dd, *J* = 79.5, 9.4 Hz, 1F). ^31^P{/^1^H} NMR (122 MHz): δ = 9.52 (d, *J* = 79.5 Hz, 1P). IR (neat): 1261, 1245, 1020, 975, 647 [cm^−1^]. MS (EI) m/z = 475.3 [M-H]^+^.

rac-**18b**: ^1^H NMR (400 MHz): δ = 7.44–7.41 (m, 3H, *H*_ar_), 7.35–7.32 (m, 2H, *H*_ar_), 7.17–7.14 (m, 2H, *H*_ar_), 6.80–6.78 (m, 2H, *H*_ar_), 4.28–4.22 (m, 3H, OC*H*_2_CH_3,_ C*H*(Ph)CFP), 4.13–4.04 (m, 2H, OC*H*_2_CH_3_), 3.76 (s, 3H, Ph(4-OC*H*_3_)), 3.65 (br d, *J* = 13.0 Hz, 1H, C*H*HN), 3.47 (br d, *J* = 13.0 Hz, 1H, C*H*HN), 1.22 (td, *J* = 7.1, 1.0 Hz, 3H, OCH_2_C*H*_3_), 1.21 (td, *J* = 7.1, 0.8 Hz, 3H, OCH_2_C*H*_3_). ^13^C NMR (101 MHz): δ = 158.80 (s, *C_ar_*(OCH_3_)), 136.37 (dd, *J* = 5.9, 2.5 Hz, *C_ipso_*), 131.54 (s, *C_ipso_*), 129.95 (d, *J* = 1.5 Hz, *C*H_ar_), 129.59, 128.55, 128.14 (3x s, *C*H_ar_), 113.77 (s, *C*H*_ar_*C*_ar_*(OCH_3_)), 106.64 (dd, *J* = 272.9, 184.4 Hz, *C*BrF), 67.72 (dd, *J* = 18.4, 8.2 Hz, *C*H(Ph)CF), 64.99 (d, *J* = 7.0 Hz, O*C*H_2_CH_3_), 64.57 (d, *J* = 7.3 Hz, O*C*H_2_CH_3_), 55.31 (s, Ph(4-O*C*H_3_)), 50.76 (s, *C*H_2_N), 16.47 (d, *J* = 5.9 Hz, OCH_2_*C*H_3_), 16.36 (d, *J* = 5.9 Hz, OCH_2_*C*H_3_). ^19^F NMR (283 MHz): δ = −135.34 (dd, *J* = 85.7, 20.6 Hz, 1F). ^31^P{/^1^H} NMR (122 MHz): δ = 8.93 (d, *J* = 85.7 Hz, 1P).

**rac-Diethyl ((1*R*, 2*R*)-1-bromo-1-fluoro-2-((4-methoxyphenyl)amino)-2-phenylethyl)phosphonate (rac-19a) and rac-diethyl ((1*R*, 2*S*)-1-bromo-1-fluoro-2-((4-methoxyphenyl)amino)-2-phenylethyl)phosphonate (rac-19b).**

Isolated as a mixture of two diastereomers rac-**19a,b** (*dr* 1:0.86), which could not be separated by the chromatography techniques employed in this study. Pale yellow oil, 209 mg, yield 91%.

rac-**19a**: ^1^H NMR (400 MHz): δ = 7.34–7.26 (m, 5H, *H*_ar_), 6.66–6.64 (m, 2H, *H*_ar_), 6.59–6.57 (m, 2H, *H*_ar_), 4.37–4.26 (m, 3H, OC*H*_2_CH_3_, C*H*(Ph)CFP), 4.17–4.10 (m, 2H, OC*H*_2_CH_3_), 3.65 (s, 3H, Ph(4-OC*H*_3_)), 1.35 (td, *J* = 7.1, 0.8 Hz, 3H, OCH_2_C*H*_3_), 1.25 (td, *J* = 7.1, 0.7 Hz, 3H, OCH_2_C*H*_3_). ^13^C NMR (101 MHz): δ = 152.95 (s, *C_ar_*(OCH_3_)), 139.48 (s, *C_ipso_*), 135.71 (d, *J* = 8.8 Hz, *C_ipso_*), 129.17 (d, *J* = 2.1 Hz, *C*H_ar_), 128.66, 128.51, 128.19 (3x s, *C*H_ar_), 114.84 (s, *C*H*_ar_*C*_ar_*(OCH_3_)), 105.61 (dd, *J* = 271.6, 183.6 Hz, *C*BrF), 65.87 (d, *J* = 6.9 Hz, O*C*H_2_CH_3_), 65.65 (d, *J* = 7.4 Hz, O*C*H_2_CH_3_), 63.30 (dd, *J* = 23.4, 6.9 Hz, *C*H(Ph)CF), 55.68 (s, Ph(4-O*C*H_3_)), 16.57 (d, *J* = 5.7 Hz, OCH_2_*C*H_3_), 16.43 (d, *J* = 5.6 Hz, OCH_2_*C*H_3_). ^19^F NMR (283 MHz): δ = −126.36 (dd, *J* = 78.1, 9.7 Hz, 1F). ^31^P{/^1^H} NMR (122 MHz): δ = 9.22 (d, *J* = 77.9 Hz, 1P). IR (neat): 1260, 1249, 1023, 974, 646 [cm^−1^]. MS (EI) m/z = 459.2 [M-H]^+^.

rac-**19b**: ^1^H NMR (400 MHz): δ = 7.51–7.43 (m, 5H, *H*_ar_), 6.70–6.67 (m, 2H, *H*_ar_), 6.57–6.53 (m, 2H, *H*_ar_), 4.24–4.17 (m, 3H, OC*H*_2_CH_3,_ C*H*(Ph)CFP), 4.10–4.00 (m, 2H, OC*H*_2_CH_3_), 3.66 (s, 3H, Ph(4-OC*H*_3_)), 1.27 (td, *J* = 7.1, 0.8 Hz, 3H, OCH_2_C*H*_3_), 1.16 (td, *J* = 7.1, 0.8 Hz, 3H, OCH_2_C*H*_3_). ^13^C NMR (101 MHz): δ = 152.82 (s, *C_ar_*(OCH_3_)), 140.04 (s, *C_ipso_*), 136.51 (d, *J* = 4.8 Hz, *C_ipso_*), 129.36 (d, *J* = 1.7 Hz, *C*H_ar_), 128.85, 128.61, 128.49 (3x s, *C*H_ar_), 115.68 (s, *C*H*_ar_*C*_ar_*(OCH_3_)), 105.37 (dd, *J* = 274.0, 181.3 Hz, *C*BrF), 66.49 (dd, *J* = 19.7, 7.6 Hz, *C*H(Ph)CF), 65.06 (d, *J* = 6.6 Hz, O*C*H_2_CH_3_), 64.79 (d, *J* = 6.7 Hz, O*C*H_2_CH_3_), 55.69 (s, Ph(4-O*C*H_3_)), 16.36 (d, *J* = 5.7 Hz, OCH_2_*C*H_3_), 16.24 (d, *J* = 5.9 Hz, OCH_2_*C*H_3_). ^19^F NMR (283 MHz): δ = −135.34 (dd, *J* = 82.1, 18.6 Hz, 1F). ^31^P{/^1^H} NMR (122 MHz): δ = 8.17 (d, *J* = 82.1 Hz, 1P).

**Diethyl ((1*S*/1*R*, 2*S*/2*R*)-1-chloro-1-fluoro-2-phenyl-2-(((*S*)-1-phenylethyl)amino)ethyl)phosphonate (20a-d).**

Isolated as a mixture of four diastereomers **20a**–**d** (*dr* 1:0.83:0.07:0.11). Pale-yellow oil, 196 mg, yield 95%.

**Diethyl ((1*R*, 2*R*)-1-chloro-1-fluoro-2-phenyl-2-(((*S*)-1-phenylethyl)amino)ethyl)phosphonate (20a).**

^1^H NMR (400 MHz): δ = 7.35–7.32 (m, 5H, *H*_ar_), 7.27–7.25 (m, 2H, *H*_ar_), 7.21–7.19 (m, 3H, *H*_ar_), 4.71 (dd, *J* = 8.9, 4.6 Hz, 1H, C*H*(Ph)CF), 4.38–4.31 (m, 2H, OC*H*_2_CH_3_), 4.29–4.26 (m, 2H, OC*H*_2_CH_3_), 3.74 (q, *J* = 6.4 Hz, C*H*CH_3_), 1.36 (td, *J* = 7.1, 0.8 Hz, 3H, OCH_2_C*H*_3_), 1.34 (d, *J* = 6.4 Hz, 3H, CHC*H*_3_), 1.33 (td, *J* = 7.1, 0.8 Hz, 3H, OCH_2_C*H*_3_). ^13^C NMR (101 MHz): δ = 145.40 (s, *C_ipso_*), 135.16 (d, *J* = 8.2 Hz, *C_ipso_*), 128.49, 128.45, 128.40, 128.09, 127.21, 126.76 (6x s, *C*H_ar_), 110.45 (dd, *J* = 260.4, 191.5 Hz, *C*ClF), 65.32 (d, *J* = 6.9 Hz, O*C*H_2_CH_3_), 64.97 (d, *J* = 7.2 Hz, O*C*H_2_CH_3_), 63.80 (dd, *J* = 23.3, 8.1 Hz, *C*H(Ph)CF), 54.76 (s, *C*HCH_3_), 21.68 (s, CH*C*H_3_), 16.54 (d, *J* = 6.0 Hz, OCH_2_*C*H_3_), 16.42 (d, *J* = 6.3 Hz, OCH_2_*C*H_3_). ^19^F NMR (283 MHz): δ = −123.06 (dd, *J* = 87.4, 8.8 Hz, 1F). ^31^P{/^1^H} NMR (122 MHz): δ = 9.61 (d, *J* = 87.4 Hz, 1P). IR (neat): 1264, 1025, 984, 673 [cm^−1^]. MS (EI) m/z = 413.2 [M]^+^.

**Diethyl ((1*S*, 2*R*)-1-chloro-1-fluoro-2-phenyl-2-(((*S*)-1-phenylethyl)amino)ethyl)phosphonate (20b).**

^1^H NMR (400 MHz): δ = 7.32–7.29 (m, 5H, *H*_ar_), 7.25–7.24 (m, 2H, *H*_ar_), 7.23–7.21 (m, 2H, *H*_ar_), 7.19–7.18 (m, 1H, *H*_ar_), 4.41 (dd, *J* = 21.3, 3.3 Hz, 1H, C*H*(Ph)CF), 4.24–4.19 (m, 2H, OC*H*_2_CH_3_), 4.18–4.04 (m, 2H, OC*H*_2_CH_3_), 3.73 (q, *J* = 6.6 Hz, 1H, C*H*CH_3_), 1.33 (d, *J* = 6.3 Hz, 3H, CHC*H*_3_), 1.29 (td, *J* = 7.1, 0.8 Hz, 3H, OCH_2_C*H*_3_), 1.26 (td, *J* = 7.1, 0.8 Hz, 3H, OCH_2_C*H*_3_). ^13^C NMR (101 MHz): δ = 145.55 (s, *C_ipso_*), 136.48 (dd, *J* = 6.1, 2.7 Hz, *C_ipso_*), 128.45, 128.35, 128.30, 128.15, 127.12, 126.84 (6x s, *C*H_ar_), 111.08 (dd, *J* = 265.2, 190.1 Hz, *C*ClF), 65.83 (dd, *J* = 18.6, 8.7 Hz, *C*H(Ph)CF), 64.80 (d, *J* = 7.2 Hz, O*C*H_2_CH_3_), 64.68 (d, *J* = 7.4 Hz, O*C*H_2_CH_3_), 55.52 (s, *C*HCH_3_), 21.96 (s, CH*C*H_3_), 16.48 (d, *J* = 6.1 Hz, OCH_2_*C*H_3_), 16.43 (d, *J* = 6.2 Hz, OCH_2_*C*H_3_). ^19^F NMR (283 MHz): δ = −134.74 (dd, *J* = 89.0, 21.5 Hz, 1F). ^31^P{/^1^H} NMR (122 MHz): δ = 9.02 (d, *J* = 89.2 Hz, 1P).

**Diethyl ((1*S*, 2*S*)-1-chloro-1-fluoro-2-phenyl-2-(((*S*)-1-phenylethyl)amino)ethyl)phosphonate (20c).**

Diagnostic signals ^19^F NMR (283 MHz): δ = −125.91 (dd, *J* = 83.2, 9.2 Hz). ^31^P{/^1^H} NMR (122 MHz): δ = 8.89 (d, *J* = 83.3 Hz).

**Diethyl ((1*R*, 2*S*)-1-chloro-1-fluoro-2-phenyl-2-(((*S*)-1-phenylethyl)amino)ethyl)phosphonate (20d).**

Diagnostic signals ^19^F NMR (283 MHz): δ = −134.88 (dd, *J* = 91.3, 21.0 Hz). ^31^P{/^1^H} NMR (122 MHz): δ = 8.66 (d, *J* = 91.5 Hz).

**rac-Diethyl ((1*R*, 2*R*)-1-chloro-1-fluoro-2-((4-methoxybenzyl)amino)-2-phenylethyl)phosphonate (rac-21a) and rac-diethyl ((1*R*, 2*S*)-1-chloro-1-fluoro-2-((4-methoxybenzyl)amino)-2-phenylethyl)phosphonate (rac-21b).**

Isolated as a mixture of two diastereomers rac-**21a,b** (*dr* 1:0.92), which could not be separated by the chromatography techniques employed in this study. Pale-yellow oil, 206 mg, yield 96%.

rac-**21a**: ^1^H NMR (400 MHz): δ = 7.45–7.42 (m, 3H, *H*_ar_), 7.39–7.36 (m, 2H, *H*_ar_), 7.14–7.11 (m, 2H, *H*_ar_), 6.83–6.81 (m, 2H, *H*_ar_), 4.45 (dd, J = 8.4, 4.4 Hz, 1H, C*H*(Ph)CFP), 4.36–4.25 (m, 2H, OC*H*_2_CH_3_), 4.16–4.08 (m, 2H, OC*H*_2_CH_3_), 3.75 (s, 3H, Ph(4-OC*H*_3_)), 3.70 (br d, *J* = 12.9 Hz, 1H, C*H*HN), 3.47 (d, *J* = 12.9 Hz, 1H, CH*H*N), 1.28 (td, *J* = 7.1, 0.8 Hz, 3H, OCH_2_C*H*_3_), 1.23 (td, *J* = 7.1, 0.8 Hz, 3H, OCH_2_C*H*_3_). ^13^C NMR (101 MHz): δ = 158.86 (s, *C_ar_*(OCH_3_)), 134.75 (d, *J* = 8.3 Hz, *C_ipso_*), 131.48 (s, *C_ipso_*), 129.83 (d, *J* = 1.3 Hz, *C*H_ar_), 129.71, 128.64, 128.18 (3x s, *C*H_ar_), 113.82 (s, *C*H*_ar_*C*_ar_*(OCH_3_)), 109.70 (dd, *J* = 260.8, 191.2 Hz, *C*ClF), 65.57 (d, *J* = 6.9 Hz, O*C*H_2_CH_3_), 65.07 (dd, *J* = 22.1, 8.6 Hz, *C*H(Ph)CF), 64.92 (d, *J* = 7.1 Hz, O*C*H_2_CH_3_), 55.31 (s, Ph(4-O*C*H_3_)), 50.18 (s, *C*H_2_N), 16.45 (d, *J* = 5.9 Hz, OCH_2_*C*H_3_), 16.32 (d, *J* = 5.9 Hz, OCH_2_*C*H_3_). ^19^F NMR (283 MHz): δ = −124.54 (dd, *J* = 84.8, 8.3 Hz, 1F). ^31^P{/^1^H} NMR (122 MHz): δ = 9.37 (d, *J* = 84.7 Hz, 1P). IR (neat): 1263, 1253, 1024, 981, 672 [cm^−1^]. MS (EI) m/z = 430.8 [M-H]^+^.

rac-**21b**: ^1^H NMR (400 MHz): δ = 7.43–7.40 (m, 3H, *H*_ar_), 7.36–7.33 (m, 2H, *H*_ar_), 7.18–7.14 (m, 2H, *H*_ar_), 6.80–6.78 (m, 2H, *H*_ar_), 4.23–4.17 (m, 3H, OC*H*_2_CH_3,_ C*H*(Ph)CFP), 4.10–3.98 (m, 2H, OC*H*_2_CH_3_), 3.76 (s, 3H, Ph(4-OC*H*_3_)), 3.65 (br d, *J* = 12.9 Hz, 1H, C*H*HN), 3.46 (d, *J* = 12.9 Hz, 1H, C*H*HN), 1.25 (td, *J* = 7.1, 0.9 Hz, 3H, OCH_2_C*H*_3_), 1.22 (td, *J* = 7.0, 0.8 Hz, 3H, OCH_2_C*H*_3_). ^13^C NMR (101 MHz): δ = 158.83 (s, *C_ar_*(OCH_3_)), 135.85 (dd, *J* = 6.0, 2.4 Hz, *C_ipso_*), 131.52 (s, *C_ipso_*), 129.90 (d, *J* = 1.3 Hz, *C*H_ar_), 129.60, 128.55, 128.21 (3x s, *C*H_ar_), 113.81 (s, *C*H*_ar_*C*_ar_*(OCH_3_)), 110.35 (dd, *J* = 264.0, 191.1 Hz, *C*ClF), 67.24 (dd, *J* = 18.8, 8.9 Hz, *C*H(Ph)CF), 64.99 (d, *J* = 7.1 Hz, O*C*H_2_CH_3_), 64.61 (d, *J* = 7.2 Hz, O*C*H_2_CH_3_), 55.30 (s, Ph(4-O*C*H_3_)), 50.70 (s, *C*H_2_N), 16.42 (d, *J* = 5.9 Hz, OCH_2_*C*H_3_), 16.35 (d, *J* = 5.9 Hz, OCH_2_*C*H_3_). ^19^F NMR (283 MHz): δ = −134.91 (dd, *J* = 90.6, 20.5 Hz, 1F). ^31^P{/^1^H} NMR (122 MHz): δ = 8.76 (d, *J* = 90.8 Hz, 1P).

### 3.8. Synthesis of α-Fluorinated ß-Enaminophosphonate/ß-Iminophosphonate (***22a***,***b***/***23a***,***b***)

To a solution of the *β*-iminophosphonate (**10a,b**) (137 mg, 0.3 mmol) in anhydrous THF (2 mL), LiAlH_4_ (17 mg, 0.45 mmol) at 0 °C was added. The reaction was warmed to room temperature, and then stirred for 40 min. After this time, solvent was evaporated and chloroform (5 mL) was added to the residue. The crude mixture was filtered through a syringe filter and purified using column chromatography (AcOEt/hexane: 10% → 50%) to give yellow oil with 83% yield (94 mg), as a mixture of enamine (**22a,b**) and imine (**23a,b**): (*E-***22a**/*Z-***22b** ratio 1:0.3; **22a,b**/**23a,b** ratio 1:0.05)

**(*E*)-Diethyl (1-fluoro-2-phenyl-2-(((*S*)-1-phenylethyl)amino)vinyl)phosphonate (*E*-22a).**

^1^H NMR (401 MHz): δ = 7.38–7.35 (m, 2H, *H*_ar_), 7.28–7.25 (m, 2H, *H*_ar_), 7.24–7.20 (m, 2H, *H*_ar_), 7.19–7.14 (m, 2H, *H*_ar_), 7.05–7.02 (m, 2H, *H*_ar_), 6.48 (dd, *J* = 10.5 Hz, 4.6 Hz, N*H*), 4.09–3.76 (m, 5H, 2x OC*H*_2_CH_3_, C*H*CH_3_), 1.41 (d, *J* = 6.9 Hz, 3H, CHC*H*_3_), 1.35 (td, *J* = 7.1, 0.7 Hz, 3H, OCH_2_C*H*_3_), 1.26 (td, *J* = 7.1, 0.6 Hz, 3H, OCH_2_C*H*_3_). ^13^C NMR (101 MHz): δ = 151.41 (dd, *J* = 30.1 Hz, 17.3 Hz, *C*(Ph) = CF), 144.55 (s, *C_ipso_*), 129.52, 129.30, 128.39, 128.33, 126.49, 125.78, (6x s, *C*H_ar_), 62.77 (d, *J* = 4.8 Hz, O*C*H_2_CH_3_), 62.56 (d, *J* = 4.6 Hz, O*C*H_2_CH_3_), 54.48 (s, *C*HCH_3_), 23.41 (s, CH*C*H_3_), 16.37 (d, *J* = 6.7 Hz, OCH_2_*C*H_3_), 16.20 (d, *J* = 6.8 Hz, OCH_2_*C*H_3_). ^19^F NMR (283 MHz): δ = −175.62 (dd, *J* = 91.7, 4.6 Hz, 1F). ^31^P{/^1^H} NMR (162 MHz): δ = 12.10 (d, *J* = 91.8 Hz, 1P). MS (EI) m/z = 377.1 [M]^+^.

**(*Z*)-Diethyl(1-fluoro-2-phenyl-2-(((*S*)-1-phenylethyl)amino)vinyl)phosphonate (*Z*-22b).**

Diagnostic signals ^19^F NMR (283 MHz): δ = −163.94 (dd, *J* = 92.6, 6.1 Hz). ^31^P{/^1^H} NMR (162 MHz): δ = 9.42 (d, *J* = 92.5 Hz).

**(*E/Z*)-Diethyl(1-fluoro-2-phenyl-2-(((*S*)-1-phenylethyl)imino)ethyl)phosphonate (23a,b).**

Diagnostic signals ^19^F NMR (283 MHz): −205.17 (dd, *J* = 78.4, 46.2 Hz), −205.73 (dd, *J* = 79.1, 46.2 Hz) ^31^P{/^1^H} NMR (162 MHz): signals masked by other tautomers signals.

### 3.9. General Procedure for Synthesis of 2-Fluorinated Aziridine-2-phosphonates (***24a***–***d***, rac-***25***–***26a***,***b***)

To a solution of the *β*-aminophosphonate (**16a**–**d**, rac-**b**) (0.5 mmol) in anhydrous DMF (3 mL), triethylamine (84 µL, 61 mg, 0.6 mmol) was added. The reaction mixture was heated at 70 °C for 4 h at inert atmosphere. After completion of the reaction (monitored by ^19^F NMR), the solvent was removed under reduced pressure. The mixture was purified by column chromatography (AcOEt/petroleum ether 5% → 50%) with previously deactivated silica gel (short pad 1 cm, 1% triethylamine in hexane, 20 mL).

**Diethyl ((2*S*/2*R*, 3*R*/3*S*)-2-fluoro-3-phenyl-1-((*S*)-1-phenylethyl)aziridin-2-yl)phosphonate (24a-d).**

Crude reaction mixture: **24a**–**d** (*dr* 0.78:1:0.13:0.08). Isolated as a mixture of four diastereomers **24a**–**d** (*dr* 0.70:1:0.17:0.04) (97mg) and single diastereomer **24a** (32mg). Diagnostic signals for traces of diastereomers **24c**–**d** were determined from the crude reaction mixture. Pale-yellow oil, 129 mg, yield 68%.

**Diethyl ((2*S*,3*R*)-2-fluoro-3-phenyl-1-((*S*)-1-phenylethyl)aziridin-2-yl)phosphonate (*cis*-24a).**

^1^H NMR (401 MHz): δ = 7.49–7.43 (m, 1H, *H*_ar_), 7.30–7.21 (m, 8H, *H*_ar_), 7.15–7.10 (m, 1H, *H*_ar_), 4.08–3.96 (m, 3H, OCH_2_C*H*_3_, OC*H*HCH_3_), 3.92–3.84 (m, 1H, OCH*H*CH_3_), 3.81 (q, *J* = 6.4 Hz, 1H, C*H*CH_3_), 3.18 (d, *J* = 8.7 Hz, 1H, C*H*(Ph)CPF), 1.61 (d, *J* = 6.5 Hz, 3H, CHC*H*_3_), 1.19 (t, *J* = 7.3 Hz, 3H, OCH_2_C*H*_3_), 1.17 (t, *J* = 7.2 Hz, 3H, OCH_2_C*H*_3_). ^1^H{/^19^F} NMR (401 MHz): δ = 7.50–7.44 (m, 1H, *H*_ar_), 7.31–7.21 (m, 8H, *H*_ar_), 7.15–7.10 (m, 1H, *H*_ar_), 4.08–3.97 (m, 3H, OC*H*_2_CH_3_, OC*H*HCH_3_), 3.92–3.83 (m, 1H, OCH*H*CH_3_), 3.82 (q, *J* = 6.6 Hz, 1H, C*H*CH_3_), 3.18 (br s, 1H, C*H*(Ph)CPF), 1.61 (d, *J* = 6.5 Hz, 3H, CHC*H*_3_), 1.20 (t, *J* = 7.3 Hz, 3H, OCH_2_C*H*_3_), 1.17 (t, *J* = 7.3 Hz, 3H, OCH_2_C*H*_3_). ^1^H{/^31^P} NMR (401 MHz): δ = 7.49–7.42 (m, 1H, *H*_ar_), 7.30–7.21 (m, 8H, *H*_ar_), 7.14–7.10 (m, 1H, *H*_ar_), 4.08–3.95 (m, 3H, OC*H*_2_CH_3_, OC*H*HCH_3_), 3.91–3.84 (m, 1H, OCH*H*CH_3_), 3.81 (q, *J* = 6.4 Hz, 1H, C*H*CH_3_), 3.18 (d, *J* = 8.7 Hz, 1H, C*H*(Ph)CPF), 1.61 (d, *J* = 6.5 Hz, 3H, CHC*H*_3_), 1.20 (t, *J* = 7.3 Hz, 3H, OCH_2_C*H*_3_), 1.17 (t, *J* = 7.2 Hz, 3H, OCH_2_C*H*_3_). ^13^C NMR (101 MHz): δ = 143.12 (s, *C_ipso_*), 133.87 (s, *C_ipso_*), 128.53, 128.38, 128.09, 127.74, 127.60, 127.43 (6x s, *C*H_ar_), 87.05 (dd, *J* = 274.2, 272.1 Hz, *C*FP), 63.35 (d, *J* = 6.2 Hz, O*C*H_2_CH_3_), 63.35 (d, *J* = 6.2 Hz O*C*H_2_CH_3_), 60.58 (d, *J* = 13.3 Hz, *C*HCH_3_), 48.96 (dd, *J* = 19.1, 1.6 Hz, *C*H(Ph)CFP), 23.30 (s, CH*C*H_3_), 16.31 (d, *J* = 6.3 Hz, OCH_2_*C*H_3_), 16.22 (d, *J* = 6.2 Hz, OCH_2_*C*H_3_). ^19^F NMR (283 MHz): δ = −182.45 (dd, *J* = 118.5, 8.7 Hz, 1F) ^31^P{/^1^H} NMR (122 MHz): δ = 9.64 (d, *J* = 118.8 Hz, 1P).

**Diethyl ((2*R*,3*R*)-2-fluoro-3-phenyl-1-((*S*)-1-phenylethyl)aziridin-2-yl)phosphonate (*trans*-24b).**

^1^H NMR (401 MHz): δ = 7.38–7.34 (m, 2H, *H*_ar_), 7.29–7.24 (m, 4H, *H*_ar_), 7.23–7.19 (m, 3H, *H*_ar_), 7.17–7.13 (m, 1H, *H*_ar_), 4.35 (quint, *J* = 7.2 Hz, 2H, OC*H*_2_CH_3_), 4.31–4.24 (m, 1H, OC*H*HCH_3_), 4.23–4.13 (m, 2H, OCH*H*CH_3_, C*H*CH_3_), 3.26 (t, *J* = 4.2 Hz, 1H, C*H*(Ph)CFP), 1.64 (d, *J* = 6.5 Hz, 3H, CHC*H*_3_), 1.44 (td, *J* = 7.1, 0.7 Hz, 3H, OCH_2_C*H*_3_), 1.33 (td, *J* = 7.1, 0.6 Hz, 3H, OCH_2_C*H*_3_). ^1^H{/^19^F} NMR (401 MHz): δ = 7.37–7.33 (m, 2H, *H*_ar_), 7.28–7.24 (m, 4H, *H*_ar_), 7.23–7.19 (m, 3H, *H*_ar_), 7.18–7.13 (m, 1H, *H*_ar_), 4.35 (quint, *J* = 7.2 Hz, 2H, OC*H*_2_CH_3_), 4.32–4.24 (m, 1H, OC*H*HCH_3_), 4.23–4.12 (m, 2H, OCH*H*CH_3_, C*H*CH_3_), 3.26 (br d, *J* = 4.3 Hz, 1H, C*H*(Ph)CFP), 1.64 (d, *J* = 6.5 Hz, 3H, CHC*H*_3_), 1.44 (td, *J* = 7.0, 0.7 Hz, 3H, OCH_2_C*H*_3_), 1.33 (td, *J* = 7.1, 0.6 Hz, 3H, OCH_2_C*H*_3_). ^1^H{/^31^P} NMR (401 MHz): δ = 7.37–7.32 (m, 2H, *H*_ar_), 7.29–7.23 (m, 4H, *H*_ar_), 7.22–7.18 (m, 3H, *H*_ar_), 7.17–7.14 (m, 1H, *H*_ar_), 4.35 (q, *J* = 7.1 Hz, 2H, OC*H*_2_CH_3_), 4.32–4.24 (m, 1H, OC*H*HCH_3_), 4.22–4.14 (m, 2H, OCH*H*CH_3_, C*H*CH_3_), 3.26 (d, *J* = 4.5 Hz, 1H, C*H*(Ph)CFP), 1.64 (d, *J* = 6.5 Hz, 3H, CHC*H*_3_), 1.44 (t, *J* = 7.1 Hz, 3H, OCH_2_C*H*_3_), 1.33 (t, *J* = 7.1 Hz, 3H, OCH_2_C*H*_3_). ^13^C NMR (101 MHz): δ = 143.14 (s, *C_ipso_*), 133.62 (dd, *J* = 5.2, 1.5 Hz, *C_ipso_*), 128.39, 128.10, 127.73 (3x s, *C*H_ar_), 127.61 (t, *J* = 0.9 Hz, *C*H_ar_), 127.30, 127.02 (2x s, *C*H_ar_), 84.27 (dd, *J* = 258.7, 233.2 Hz, *C*FP), 64.15 (dd, *J* = 7.8, 1.1 Hz, O*C*H_2_CH_3_), 63.54 (dd, *J* = 6.0, 0.5 Hz, O*C*H_2_CH_3_), 61.18 (dd, *J* = 5.0, 3.1 Hz, *C*HCH_3_), 47.46 (dd, *J* = 12.7, 5.8 Hz, *C*H(Ph)CFP), 23.64 (s, CH*C*H_3_), 16.39 (d, *J* = 6.4 Hz, OCH_2_*C*H_3_), 16.38 (d, *J* = 6.5 Hz, OCH_2_*C*H_3_). ^19^F NMR (283 MHz): δ = −168.76 (dt, *J* = 113.8, 4.8 Hz, 1F). ^31^P{/^1^H} NMR (122 MHz): δ = 10.83 (d, *J* = 114.0 Hz, 1P). IR (neat): 1259, 1162, 1019, 957, 759 [cm^−1^]. HRMS (ESI): *m*/*z* calcd for C_20_H_26_FNO_3_P, [M + H]^+^: 378.1634 found: 378.1628.

**Diethyl ((2*R*,3*S*)-2-fluoro-3-phenyl-1-((*S*)-1-phenylethyl)aziridin-2-yl)phosphonate (*cis*-24c).**

Diagnostic signals ^19^F NMR (283 MHz): δ = −178.36 (dd, *J* = 114.2, 8.5 Hz). ^31^P{/^1^H} NMR (122 MHz): δ = 8.94 (d, *J* = 114.1 Hz).

**Diethyl ((2*S*,3*S*)-2-fluoro-3-phenyl-1-((*S*)-1-phenylethyl)aziridin-2-yl)phosphonate (*trans*-24d).**

Diagnostic signals ^19^F NMR (283 MHz): δ = −169.11 (dt, *J* = 110.8, 4.7 Hz). ^31^P{/^1^H} NMR (122 MHz): δ = 10.22 (d, *J* = 110.6 Hz).

**rac-Diethyl ((2*S*,3*R*)-2-fluoro-1-(4-methoxybenzyl)-3-phenylaziridin-2-yl)phosphonate (rac-*cis-*25a) and rac-diethyl ((2*R*,3*R*)-2-fluoro-1-(4-methoxybenzyl)-3-phenylaziridin-2-yl)phosphonate (rac-*trans*-25b).**

Crude reaction mixture: **25a,b** (*dr* 0.68:1). Isolated as a mixture of diastereomers **25a,b** (*dr* 0.52:1) (94mg) and single diastereomer **25b** (28mg). Pale yellow oil, 122 mg, yield 62%.

rac-*cis-***25a**: ^1^H NMR (401 MHz): δ = 7.45–7.40 (m, 3H, *H*_ar_), 7.36–7.29 (m, 2H, *H*_ar_), 7.23–7.19 (m, 2H, *H*_ar_), 6.92–6.88 (m, 2H, *H*_ar_), 4.15 (br d, *J* = 13.6, 1H, C*H*HN), 3.99–3.85 (m, 4H, 2x OC*H*_2_CH_3_), 4.01 (br d, *J* = 13.6, 1H, CH*H*N), 3.82 (s, 3H, Ph(4-OC*H*_3_)), 3.26 (d, *J* = 8.5 Hz, 1H, C*H*(Ph)CFP), 1.11 (td, *J* = 7.1, 0.8 Hz, 3H, OCH_2_C*H*_3_), 1.09 (td, *J* = 7.1, 0.7 Hz, 3H, OCH_2_C*H*_3_). ^1^H{/^19^F} NMR (401 MHz): δ = 7.45–7.41 (m, 2H, *H*_ar_), 7.36–7.30 (m, 2H, *H*_ar_), 7.24–7.19 (m, 2H, *H*_ar_), 6.91–6.86 (m, 2H, *H*_ar_), 4.15 (br d, *J* = 13.5, 1H, C*H*HN), 3.97–3.85 (m, 4H, 2x OC*H*_2_CH_3_), 4.02 (br d, *J* = 13.6, 1H, CH*H*N), 3.81 (s, 3H, Ph(4-OC*H*_3_)), 3.25 (br s, 1H, C*H*(Ph)CFP), 1.11 (td, *J* = 7.1, 0.8 Hz, 3H, OCH_2_C*H*_3_), 1.10 (td, *J* = 7.1, 0.6 Hz, 3H, OCH_2_C*H*_3_). ^1^H{/^31^P} NMR (401 MHz): δ = 7.45–7.41 (m, 2H, *H*_ar_), 7.36–7.30 (m, 2H, *H*_ar_), 7.24–7.19 (m, 2H, *H*_ar_), 6.91–6.86 (m, 2H, *H*_ar_), 4.15 (d, *J* = 12.8 Hz, 1H, C*H*HN), 3.98–3.84 (m, 4H, 2x OC*H*_2_CH_3_), 4.02 (d, *J* = 13.0 Hz, 1H, CH*H*N), 3.83 (s, 3H, Ph(4-OC*H*_3_)), 3.27 (d, *J* = 8.4 Hz, 1H, C*H*(Ph)CFP), 1.11 (t, *J* = 7.0 Hz, 3H, OCH_2_C*H*_3_), 1.09 (t, *J* = 7.1 Hz, 3H, OCH_2_C*H*_3_). ^13^C NMR (101 MHz): δ = 159.00 (s, *C_ar_*(OCH_3_)), 133.44–133.38 (m, *C_ipso_*), 131.86 (s, *C_ipso_*), 129.21, 128.17, 127.86, 127.56 (4x s, *C*H_ar_), 113.80 (s, *C*H*_ar_*C*_ar_*(OCH_3_)), 86.67 (dd, *J* = 274.0, 272.5 Hz, *C*FP), 63.15 (d, *J* = 6.1 Hz, O*C*H_2_CH_3_), 63.03 (d, *J* = 6.2 Hz, O*C*H_2_CH_3_), 55.23 (s, Ph(4-O*C*H_3_)), 53.30 (d, *J* = 15.1 Hz, *C*H_2_N), 49.08 (dd, *J* = 19.2, 1.3 Hz, *C*H(Ph)CFP), 16.12 (d, *J* = 6.0 Hz, OCH_2_*C*H_3_), 16.11 (d, *J* = 6.1 Hz, OCH_2_*C*H_3_). ^19^F NMR (283 MHz): δ = −180.27 (dd, *J* = 116.7, 8.4 Hz, 1F). ^31^P{/^1^H} NMR (122 MHz): δ = 9.97 (d, *J* = 116.9 Hz, 1P).

rac-*trans*-**25b**: ^1^H NMR (401 MHz): δ = 7.39 (d, *J* = 7.7 Hz, 4H, *H*_ar_), 7.36–7.30 (m, 3H, *H*_ar_), 6.87 (d, *J* = 8.6 Hz, 2H, *H*_ar_), 4.46 (dd, *J* = 13.6, 2.8 Hz, 1H, C*H*HN), 4.34–4.27 (m, 1H, OCH*H*CH_3_), 4.25–4.18 (m, 3H, OC*H*HCH_3_, OC*H*_2_CH_3_), 4.08 (dd, *J* = 13.6, 5.1 Hz, 1H, CH*H*N), 3.80 (s, 3H, Ph(4-OC*H*_3_)), 3.45 (t, *J* = 4.1 Hz, 1H, C*H*(Ph)CFP), 1.37 (td, *J* = 7.1, 0.6 Hz, 3H, OCH_2_C*H*_3_), 1.34 (td, *J* = 7.1, 0.7 Hz, 3H, OCH_2_C*H*_3_). ^1^H{/^19^F} NMR (401 MHz): δ = 7.40–7.35 (m, 4H, *H*_ar_), 7.37–7.32 (m, 3H, *H*_ar_), 6.88 (d, *J* = 8.8 Hz, 2H, *H*_ar_), 4.47 (d, *J* = 13.6, 1H, C*H*HN), 4.32–4.26 (m, 1H, OCH*H*CH_3_), 4.25–4.17 (m, 3H, OC*H*HCH_3_, OC*H*_2_CH_3_), 4.08 (d, *J* = 13.5, 1H, CH*H*N), 3.81 (s, 3H, Ph(4-OC*H*_3_)), 3.45 (d, *J* = 4.1 Hz, 1H, C*H*(Ph)CFP), 1.38 (td, *J* = 7.1, 0.6 Hz, 3H, OCH_2_C*H*_3_), 1.35 (td, *J* = 7.1, 0.7 Hz, 3H, OCH_2_C*H*_3_). ^1^H{/^31^P} NMR (401 MHz): δ = 7.40–7.36 (m, 4H, *H*_ar_), 7.37–7.32 (m, 3H, *H*_ar_), 6.88 (d, *J* = 8.7 Hz, 2H, *H*_ar_), 4.47 (dd, *J* = 13.6, 2.8 Hz, 1H, C*H*HN), 4.33–4.26 (m, 1H, OCH*H*CH_3_), 4.25–4.18 (m, 3H, OC*H*HCH_3_, OC*H*_2_CH_3_), 4.08 (dd, *J* = 13.6, 5.1 Hz, 1H, CH*H*N), 3.81 (s, 3H, Ph(4-OC*H*_3_)), 3.45 (d, *J* = 4.3 Hz, 1H, C*H*(Ph)CFP), 1.38 (t, *J* = 7.1, 3H, OCH_2_C*H*_3_), 1.35 (t, *J* = 7.1, 3H, OCH_2_C*H*_3_). ^13^C NMR (101 MHz): δ = 158.79 (s, *C_ar_*(OCH_3_)), 133.58 (br d, *J* = 5.2 Hz, *C_ipso_*), 131.93 (s, *C_ipso_*), 129.99, 128.15, 127.84, 127.67 (4x s, *C*H_ar_), 113.71 (s, *C*H*_ar_*C*_ar_*(OCH_3_)), 83.95 (dd, *J* = 259.6, 231.6 Hz, *C*FP), 63.71 (d, *J* = 7.0 Hz, O*C*H_2_CH_3_), 63.50 (d, *J* = 6.0 Hz, O*C*H_2_CH_3_), 55.53 (dd, *J* = 5.4, 3.5 Hz, *C*H_2_N), 55.19 (s, Ph(4-O*C*H_3_)), 48.43 (dd, *J* = 12.9, 6.0 Hz, *C*H(Ph)CFP), 16.27 (d, *J* = 5.9 Hz, OCH_2_*C*H_3_), 16.25 (d, *J* = 6.0 Hz, OCH_2_*C*H_3_). ^19^F NMR (283 MHz): δ = −170.98 (dq, *J* = 111.7, 4.3 Hz, 1F). ^31^P{/^1^H} NMR (122 MHz): δ = 10.95 (d, *J* = 111.6 Hz, 1P). IR (neat): 1247, 1164, 1097, 1018, 978, 765 [cm^−1^]. HRMS (ESI): *m*/*z* calcd for C_20_H_26_FNO_4_P, [M + H]^+^: 394.1583 found: 394.1587.

**rac-Diethyl ((2*S*,3*R*)-2-fluoro-1-(4-methoxyphenyl)-3-phenylaziridin-2-yl)phosphonate (rac-*cis-*26a) and rac diethyl ((2*R*,3*R*)-2-fluoro-1-(4-methoxyphenyl)-3-phenylaziridin-2-yl)phosphonate (rac-*trans-*26b).**

Crude reaction mixture: **26a,b** (*dr* 0.09:1). Isolated as single diastereomer **26b**. Diagnostic signals for traces of diastereomers **26a** were determined from the crude reaction mixture. Pale-yellow oil, 85 mg, yield 45%.

rac-*cis-***26a**: Diagnostic signals ^19^F NMR (283 MHz): δ = −171.22 (dd, *J* = 117.5, 7.9 Hz). ^31^P{/^1^H} NMR (122 MHz): δ = 8.63 (d, *J* = 117.4 Hz).

rac-*trans-***26b**: ^1^H NMR (401 MHz): δ = 7.51–7.48 (m, 2H, *H*_ar_), 7.40–7.34 (m, 3H, *H*_ar_), 7.13–7.06 (m, 2H, *H*_ar_), 6.86–6.82 (m, 2H, *H*_ar_), 4.19–3.98 (m, 4H, 2x OC*H*_2_CH_3_), 3.84 (t, *J* = 4.1 Hz, 1H, C*H*(Ph)CFP), 3.77 (s, 3H, Ph(4-OC*H*_3_)), 1.31 (td, *J* = 7.1, 0.6 Hz, 3H, OCH_2_C*H*_3_), 1.17 (td, *J* = 7.1, 0.6 Hz, 3H, OCH_2_C*H*_3_). ^1^H{/^19^F} NMR (401 MHz): δ = 7.52–7.47 (m, 2H, *H*_ar_), 7.42–7.35 (m, 3H, *H*_ar_), 7.11–7.06 (m, 2H, *H*_ar_), 6.85–6.80 (m, 2H, *H*_ar_), 4.19–3.96 (m, 4H, 2x OC*H*_2_CH_3_), 3.84 („d”, *J* = 4.2 Hz, 1H, C*H*(Ph)CFP), 3.76 (s, 3H, Ph(4-OC*H*_3_)), 1.31 (td, *J* = 7.1, 0.6 Hz, 3H, OCH_2_C*H*_3_), 1.17 (td, *J* = 7.1, 0.6 Hz, 3H, OCH_2_C*H*_3_). ^1^H{/^31^P} NMR (401 MHz): δ = 7.50–7.46 (m, 2H, *H*_ar_), 7.41–7.34 (m, 3H, *H*_ar_), 7.11–7.05 (m, 2H, *H*_ar_), 6.85–6.82 (m, 2H, *H*_ar_), 4.20–3.99 (m, 4H, 2x OC*H*_2_CH_3_), 3.83 (d, *J* = 4.5 Hz, 1H, C*H*(Ph)CFP), 3.77 (s, 3H, Ph(4-OC*H*_3_)), 1.30 (t, *J* = 7.1 Hz, 3H, OCH_2_C*H*_3_), 1.16 (t, *J* = 7.1 Hz, 3H, OCH_2_C*H*_3_). ^13^C NMR (101 MHz): δ = 156.13 (s, *C_ar_*(OCH_3_)), 132.83 (dd*, J* = 5.3, 1.2 Hz, *C_ipso_*), 134.22 (s, *C_ipso_*), 129.30 (d, *J* = 3.2 Hz, *C*H_ar_), 128.57, 128.38, 127.79 (3x s, *C*H_ar_), 114.24 (s, *C*H*_ar_*C*_ar_*(OCH_3_)), 83.91 (dd, *J* = 260.8, 238.3 Hz, *C*FP), 63.63 (d, *J* = 6.9 Hz, O*C*H_2_CH_3_), 63.21 (d, *J* = 6.0 Hz, O*C*H_2_CH_3_), 55.49 (s, Ph(4-O*C*H_3_)), 46.12 (dd, *J* = 13.1 Hz, 5.4 Hz, *C*H(Ph)CFP), 16.29 (d, *J* = 6.1 Hz, OCH_2_*C*H_3_), 16.17 (d, *J* = 5.6 Hz, OCH_2_*C*H_3_). ^19^F NMR (283 MHz): δ = −169.58 (dd, *J* = 121.4, 4.3 Hz, 1F). ^31^P{/^1^H} NMR (122 MHz): δ = 8.82 (d, *J* = 121.5 Hz, 1P). HRMS (ESI): *m*/*z* calcd for C_19_H_24_FNO_4_P, [M + H]^+^: 380.1427 found: 380.1421.

### 3.10. Separation Method for Chiral Aziridines and Synthesis of Non-Fluorinated Aziridine-2-phosphonate: Diethyl ((2S,3R)-3-Phenyl-1-((S)-1-phenylethyl)aziridin-2-yl)phosphonate (cis-***27***)

*Method A*. Imine **10a-d** (*dr* 1:1, 228 mg, 0.5 mmol) was dissolved in anhydrous methanol (5 mL) and NaBH_3_CN (250 mg, 4 mmol), and glacial CH_3_COOH (88 µL, 92 mg, 1.5 mmol) was added. The reaction mixture was refluxed for 7h and next the solvent was evaporated. Then, the residue was dissolved in CH_2_Cl_2_ (3 mL) and extracted with a saturated solution of NaHCO_3_ and brine. The organic layers were combined, dried over Na_2_SO_4_, and evaporated to give a mixture of products **16a,c**, *trans*-**24b,d**, and *cis*-**27**. The product *cis-***27** was purified by column chromatography (AcOEt/hexane: 5% → 60%) as a pale-yellow oil (58 mg, yield 32%). Amine **16a,c** (*dr* 1:0.1) and aziridine *trans*-**24b,d** (*dr* 1:0.05) were isolated as a mixture (0.3:1, 84 mg).

*Method B*. To the mixture of *cis*- and *trans*-aziridines (**24a-d;** 0.64(*dr* 1:0.07)/1(*dr* 1:0.09), 113 mg, 0.3 mmol) dissolved in methanol (3 mL), Pd/C (10 mol%, 2 mg) and NaBH_4_ (23 mg, 0.6 mmol) were added. The reaction was stirred at 70 °C for 3h. Then, the solvent was evaporated, the residue was dissolved in CHCl_3_ (3 mL), water (10 mL) was added, and it was extracted (3 × 10 mL CHCl_3_). The organic layers were dried over anhydrous Na_2_SO_4_ and evaporated to give crude products *trans*-**24b,d**/*cis-***27** (1:0.49) separated using column chromatography with previously deactivated silica gel (short pad 1 cm, 1% triethylamine in hexane, 20 mL). *Cis-***27** was isolated as a single diastereomer (42 mg, yield 39%) and *trans-***24b,d** was isolated as a mixture of diastereomers (*dr* 1:0.07, 49 mg).

*cis*-**27**: ^1^H NMR (401 MHz): δ = 7.44–7.40 (m, 2H, *H*_ar_), 7.31–7.26 (m, 4H, *H*_ar_), 7.23–7.18 (m, 2H, *H*_ar_), 7.17–7.10 (m, 2H, *H*_ar_), 3.99– 3.90 (m, 2H, OC*H*_2_CH_3_), 3.85–3.78 (m, 1H, OCH*H*CH_3_), 3.64–3.54 (m, 1H, OC*H*HCH_3_), 2.95 (t, *J* = 6.7 Hz, 1H, C*H*(Ph)CHP), 2.74 (q, *J* = 6.5 Hz, 1H, C*H*CH_3_), 1.99 (dd, *J* = 17.8, 6.8 Hz, 1H, C*H*P), 1.56 (d, *J* = 6.5 Hz, 3H, CHC*H*_3_), 1.17 (td, *J* = 7.1, 0.6 Hz, 3H, OCH_2_C*H*_3_), 1.07 (td, *J* = 7.1, 0.6 Hz, 3H, OCH_2_C*H*_3_). ^1^H{/^31^P} NMR (401 MHz): δ = 7.46–7.40 (m, 2H, *H*_ar_), 7.32–7.25 (m, 4H, *H*_ar_), 7.24–7.18 (m, 2H, *H*_ar_), 7.17–7.11 (m, 2H, *H*_ar_), 4.00–3.92 (m, 2H, OC*H*_2_CH_3_), 3.86–3.78 (m, 1H, OCH*H*CH_3_), 3.65–3.56 (m, 1H, OC*H*HCH_3_), 2.96 (d, *J* = 6.7 Hz, 1H, C*H*(Ph)CHP), 2.75 (q, *J* = 6.5 Hz, 1H, C*H*CH_3_), 2.00 (d, *J* = 6.8 Hz, 1H, C*H*P), 1.57 (d, *J* = 6.6 Hz, 3H, CHC*H*_3_), 1.18 (t, *J* = 7.1 Hz, 3H, OCH_2_C*H*_3_), 1.08 (t, *J* = 7.1 Hz, 3H, OCH_2_C*H*_3_). ^13^C NMR (101 MHz): δ = 143.56 (s, *C_ipso_*), 135.94 (d, *J* = 2.0 Hz, *C_ipso_*), 128.54, 128.06, 127.69, 127.51, 127.18, 127.09, (6 x s, *C*H_ar_), 71.85 (d, *J* = 6.1 Hz, *C*HCH_3_), 62.10 (d, *J* = 6.4 Hz, O*C*H_2_CH_3_), 61.81 (d, *J* = 6.4 Hz, O*C*H_2_CH_3_), 46.27 (d, *J* = 5.7 Hz, *C*H(Ph)CHP), 39.86 (s, *C*HP), 23.12 (s, CH*C*H_3_), 16.39 (d, *J* = 6.5 Hz, OCH_2_*C*H_3_), 16.36 (d, *J* = 6.0 Hz, OCH_2_*C*H_3_). ^31^P{/^1^H} NMR (162 MHz): δ = 21.44 (s, 1P). HRMS (ESI): *m*/*z* calcd for C_20_H_27_NO_3_P, [M + H]^+^: 360.1729 found: 360.1723.

### 3.11. Isolation of Trans-Aziridine ***24***. Synthesis of α-Fluorinated ß-Aminophosphonate: Diethyl ((1S/R, 2S/R)-1-Fluoro-2-phenyl-2-(((S)-1-phenylethyl)amino)ethyl)phosphonate (***28a***–***d***)

To the mixture of amine (**16a,c**) and aziridine (**24b,d**) (0.8 (*dr* 1:0.1): 1(*dr* 1:0.05), 186 mg) dissolved in methanol (2 mL), NaBH_4_ (15 mg, 0.6 mmol) and Pd/C (10 mol%, 2 mg) were added. The reaction was stirred at room temperature for 20 min. Then, the crude mixture was filtrated through Celite with MeOH as a mobile phase and concentrated under vacuum. The residue was dissolved in CH_2_Cl_2_ (2 mL) and extracted with brine. The organic layers were dried over anhydrous Na_2_SO_4_ and evaporated to give a mixture of monofluorinated amine **28a**–**d** and aziridine **24b,d** (0.8:1, respectively). The crude products **24b,d**/**28a**–**d** (**28a**–**d**: *dr* 1:0.8:0.19:0.13) were separated using column chromatography with previously deactivated silica gel (1% triethylamine in hexane, 20 mL). Amine was isolated as a mixture of diastereomers **28a**–**d** (*dr* 1:0.75:0.09:0.05, 70 mg, pale-yellow oil) and aziridine was separated as a mixture of diastereomers **24b,d** (*dr* 1:0.05, 75 mg).

**28a**: ^1^H NMR (401 MHz): δ = 7.43–7.34 (m, 5H, *H*_ar_), 7.33–7.26 (m, 5H, *H*_ar_), 4.85 (ddd, *J* = 45.2, 5.1, 3.9 Hz, 1H, C*H*FP), 4.38 (ddd, *J* = 23.5, 6.2, 5.1 Hz, 1H, C*H*CFP), 4.02–3.76 (m, 4H, 2x OC*H*_2_CH_3_), 3.67 (q, *J* = 6.4 Hz, 1H, C*H*CH_3_), 1.36 (d, *J* = 6.5 Hz, 3H, CHC*H*_3_), 1.27 (td, *J* = 7.1, 0.6 Hz, 3H, OCH_2_C*H*_3_), 1.26 (td, *J* = 7.1, 0.5 Hz, 3H, OCH_2_C*H*_3_). ^13^C NMR (101 MHz): δ = 145.61 (s, *C_ipso_*) 138.58 (d, *J* = 6.6, 1.3 Hz, *C_ipso_*), 128.37, 128.34, 128.25, 127.85, 126.91, 126.58 (6x s, *C*H_ar_), 92.58 (dd, *J* = 188.3, 167.2 Hz, *C*FP), 63.01 (d, *J* = 6.9 Hz, O*C*H_2_CH_3_), 62.10 (d, *J* = 6.9 Hz, O*C*H_2_CH_3_), 59.76 (dd, *J* = 17.9, 3.5 Hz, *C*HCFP), 54.55 (s, *C*HCH_3_), 22.08 (s, CH*C*H_3_), 16.33 (d, *J* = 5.9 Hz, OCH_2_*C*H_3_), 16.15 (d, *J* = 6.0 Hz, OCH_2_*C*H_3_). ^19^F NMR (283 MHz): δ = −213.48 (ddd, *J* = 72.0, 45.6, 21.2 Hz, 1F). ^31^P{/^1^H} NMR (122 MHz): δ = 16.01 (d, *J* = 72.2 Hz, 1P). IR (neat): 1253, 1019, 969 [cm^−1^]. MS (EI) m/z = 379.4 [M]^+^.

**28b**: ^1^H NMR (401 MHz): δ = 7.50–7.43 (m, 5H, *H*_ar_), 7.35–7.32 (m, 5H, *H*_ar_), 4.99 (ddd, *J* = 45.8, 5.8, 3.7 Hz, 1H, C*H*FP), 4.28–4.06 (m, 5H, 2x OC*H*_2_CH_3_, C*H*CFP), 3.71 (q, *J* = 6.4 Hz, 1H, C*H*CH_3_), 1.34 (d, *J* = 6.5 Hz, 3H, CHC*H*_3_), 1.18 (dd, *J* = 7.1, 0.5 Hz, 3H, OCH_2_C*H*_3_), 1.15 (dd, *J* = 7.1, 0.6 Hz, 3H, OCH_2_C*H*_3_). ^13^C NMR (101 MHz): δ = 145.45 (s, *C_ipso_*) 138.43 (dd, *J* = 8.6, 3.6 Hz, *C_ipso_*), 128.32, 128.29, 128.20, 127.76, 126.82, 126.57 (6x s, *C*H_ar_), 90.89 (dd, *J* = 187.8, 165.4 Hz, *C*FP), 63.38 (d, *J* = 6.4 Hz, O*C*H_2_CH_3_), 62.55 (d, *J* = 6.7 Hz, O*C*H_2_CH_3_), 59.66 (dd, *J* = 19.9, 4.6 Hz, *C*HCFP), 54.45 (s, *C*HCH_3_), 21.50 (s, CH*C*H_3_), 16.24 (d, *J* = 5.9 Hz, OCH_2_*C*H_3_), 16.21 (d, *J* = 6.0 Hz, OCH_2_*C*H_3_). ^19^F NMR (283 MHz): δ = −215.99 (ddd, *J* = 76.4, 45.3, 23.5 Hz, 1F). ^31^P{/^1^H} NMR (122 MHz): δ = 16.16 (d, *J* = 76.4 Hz, 1P). MS (EI) m/z = 379.4 [M]^+^.

**28c**: Diagnostic signals ^19^F NMR (283 MHz): δ = −212.07 (ddd, *J* = 70.3, 45.3, 16.4 Hz). ^31^P{/^1^H} NMR (122 MHz): δ = 15.99 (d, *J* = 70.1 Hz).

**28d**: Diagnostic signals ^19^F NMR (283 MHz): δ = −216.77 (ddd, *J* = 79.8, 45.2, 24.3 Hz). ^31^P{/^1^H} NMR (122 MHz): δ = 15.88 (d, *J* = 79.6 Hz).

### 3.12. Ring Opening of Fluorinated Aziridine-2-phosphonate: Synthesis of Diethyl ((1R, 2S)-1,2-Dimethoxy-2-phenyl-1-(((S)-1-phenylethyl)amino)ethyl)phosphonate (***29a***) and Diethyl ((1S, 2S)-1,2-Dimethoxy-2-phenyl-1-(((S)-1-phenylethyl)amino)ethyl)phosphonate (***29b***)

To the mixture of aziridines **24a**–**d** (*cis/trans* 1(*dr* 1:0.45):0.65(*dr* 1:0.04), 211 mg, 0.5 mmol) dissolved in MeOH (2 mL), H_2_SO_4_ (98%; 27 µL, 49 mg, 0.5 mmol) was added dropwise. The reaction was stirred at 70 °C for 2h. Next, the solution was concentrated, and the crude mixture was neutralized with aqueous NaHCO_3_, extracted with CH_2_Cl_2_ (3 × 6 mL), and washed with brine (6 mL). The organic layers were combined and dried over anhydrous Na_2_SO_4_. After evaporation of the solvent, the crude product was purified by column chromatography to give a mixture of diastereomers **29a,b** (*dr* 1:0.5) as a pale-yellow oil, 149 mg, yield 63%.

**29a**: ^1^H NMR (401 MHz): δ = 7.42–7.39 (m, 5H, *H*_ar_), 7.35–7.31 (m, 5H, *H*_ar_), 4.33 (d, *J* = 11.9 Hz, 1H, C*H*OCH_3_), 4.02–3.89 (m, 4H, 2x OC*H*_2_CH_3_), 3.50 (q, *J* = 6.7 Hz, 1H, C*H*CH_3_), 3.48 (d, *J* = 0.7 Hz, 3H, C(P)OC*H*_3_), 3.28 (s, 3H, CHOC*H*_3_), 1.25 (d, *J* = 6.4 Hz, 3H, CHC*H*_3_), 1.18 (t, *J* = 7.1 Hz, 3H, OCH_2_C*H*_3_), 1.11 (t, *J* = 7.0 Hz, 3H, OCH_2_C*H*_3_). ^1^H{/^31^P} NMR (401 MHz): δ = 7.43–7.39 (m, 5H, *H*_ar_), 7.37–7.32 (m, 5H, *H*_ar_), 4.32 (s, 1H, C*H*OCH_3_), 4.00–3.89 (m, 4H, 2x OC*H*_2_CH_3_), 3.51 (q, *J* = 6.7 Hz, 1H, C*H*CH_3_), 3.49 (s, 3H, C(P)OC*H*_3_), 3.29 (s, 3H, CHOC*H*_3_), 1.25 (d, *J* = 6.4 Hz, 3H, CHC*H*_3_), 1.19 (t, *J* = 7.0 Hz, 3H, OCH_2_C*H*_3_), 1.11 (t, *J* = 7.1 Hz, 3H, OCH_2_C*H*_3_). ^13^C NMR (101 MHz): δ = 144.55 (s, *C_ipso_*), 138.47 (d, *J* = 5.5 Hz, *C_ipso_*), 104.47 (d, *J* = 196.3 Hz, *C*P), 63.38 (d, *J* = 14.7 Hz, *C*HOCH_3_), 63.02 (d, *J* = 6.7 Hz, O*C*H_2_CH_3_), 62.70 (d, *J* = 6.9 Hz, O*C*H_2_CH_3_), 53.73 (s, *C*HCH_3_), 52.79 (d, *J* = 4.0 Hz, CHO*C*H_3_), 50.49 (d, *J* = 8.9 Hz, C(P)O*C*H_3_), 20.91 (s, CH*C*H_3_), 16.48 (d, *J* = 5.9 Hz, OCH_2_*C*H_3_), 16.31 (d, *J* = 6.1 Hz, OCH_2_*C*H_3_). ^31^P{/^1^H} NMR (122 MHz): δ = 18.20 (s, 1P). HRMS (ESI): *m*/*z* calcd for C_22_H_33_NO_5_P, [M + H]^+^: 422.2096 found: 422.2092.

**29b**: ^1^H NMR (401 MHz): δ = 7.28–7.24 (m, 5H, *H*_ar_), 7.22–7.17 (m, 5H, *H*_ar_), 4.13–4.02 (m, 5H, 2x OC*H*_2_CH_3_, C*H*OCH_3_), 3.42 (q, *J* = 6.5 Hz, 1H, C*H*CH_3_), 3.34 (br s, 3H, C(P)OC*H*_3_), 3.11 (br s, 3H, CHOC*H*_3_), 1.24 (d, *J* = 6.6 Hz, 3H, CHC*H*_3_), 1.17 (t, *J* = 7.1 Hz, 3H, OCH_2_C*H*_3_), 1.04 (t, *J* = 7.1 Hz, 3H, OCH_2_C*H*_3_). ^13^C NMR (101 MHz): δ = 145.03 (s, *C_ipso_*), 138.21 (d, *J* = 3.1 Hz, *C_ipso_*), 103.89 (d, *J* = 196.3 Hz, *C*P), 63.09 (d, *J* = 6.9 Hz, O*C*H_2_CH_3_), 62.58 (d, *J* = 6.8 Hz, O*C*H_2_CH_3_), 62.24 (d, *J* = 7.2 Hz, *C*HOCH_3_), 54.16 (s, *C*HCH_3_), 52.27 (d, *J* = 3.0 Hz, CHO*C*H_3_), 49.65 (d, *J* = 10.4 Hz, C(P)O*C*H_3_), 21.85 (s, CH*C*H_3_), 16.25 (d, *J* = 6.1 Hz, OCH_2_*C*H_3_), 16.15 (d, *J* = 6.2 Hz, OCH_2_*C*H_3_). ^31^P{/^1^H} NMR (122 MHz): δ = 17.32 (s, 1P). IR (neat): 1494, 1452, 1276, 1259, 1233, 1024, 969 [cm^−1^].

## 4. Conclusions

In conclusion, we have successfully developed the first synthesis of *N*-inactivated aziridines **24**–**26** bearing both a fluorine and phosphonate group on the same carbon atom. Our synthetic methodology involved the one-pot halofluorination of an enamine-imine tautomeric mixture, resulting in *α*,*α*-halofluorinated *β*-iminophosphonates **10**–**15**, which were subsequently reduced to yield the corresponding *β*-aminophosphonates **16**–**21**. When starting from (*R*)- or (*S*)-*α*-methylbenzyl imine derivatives **10**–**11,14**, the reduction occurred with high diastereoselectivity (*dr* 1:1:0.1:0.07). We have also investigated the influence of the solvent and the base on the aziridine ratio and reaction yield. Based on the spectroscopic and theoretical studies, we have determined the *cis*/*trans* geometry of aziridines obtained as a racemic mixture or prepared in a diastereoselective manner, through intramolecular cyclization. Our procedure involving the reduction of *cis/trans*-aziridine mixture **24** allows us to isolate chiral *trans-*aziridines **24** as well as fluorine*-*free *cis* aziridines **27**. Moreover, the *cis/trans* fluoroaziridines **24** can react with sulfuric acid and methanol to give the non-fluorinated *α*, *β*-dimethoxy-*α*-aminophosphonate **29** in high yield.

The conformational analysis of both diastereomers of *α,α*-bromofluoro *β*-aminophosphonates conducted through DFT calculations (PCM/ωB97x-D/def2-TZVPD level of theory) allowed us to conclude that the stability of P=O…H-N hydrogen bonding can be influenced by the electrostatic interaction between C-F (C-Br) and N-H, except when it leads to a phosphonate−aromatic (P=O…π) repulsive interaction. This analysis has also confirmed the configuration at stereogenic centers. To explain the observed differences in the cyclization tendencies of pairs of *β*-aminophosphonates, the proper transition states of the aziridine ring-closure reaction have been modeled.

## Figures and Tables

**Table 1:** Synthesis of *gem*-halofluorinated *ß*-iminophosphonates (**10**–**11,14a,b** and rac-**12**–**13,15**).

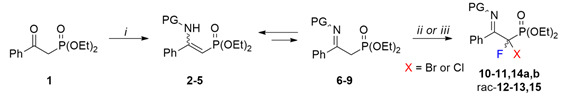						
Entry	PG	Enamine/Imine	Yield [%]	Imine	X	Yield [%]
1	(*S*)-MBn	**2a,b/6a,b**	93 ^a^	**10a,b**	Br	92 ^c^
2	(*R*)-MBn	**3a,b/7a,b**	91 ^a^	**11a,b**	Br	95 ^c^
3	PMB	**4a,b/8a,b**	85 ^a^	rac-**12**	Br	94
4	PMP	**5a/9a,b**	93 ^b^	rac-**13**	Br	87
5	(*S*)-MBn	**2a,b/6a,b**	93 ^a^	**14a,b**	Cl	94 ^c^
6	PMB	**4a,b/8a,b**	85 ^a^	rac-**15**	Cl	91

Reaction conditions: *i*. **1** (0.5 mmol), PG-NH_2_ (0.5 mmol), MgSO_4_, toluene, reflux, 24 h, Dean-Stark; *ii*. NBS (0.5 mmol), Selectfluor (0.9 mmol), CH_3_CN, rt, 15 min or *iii*. NCS (0.5 mmol), Selectfluor (0.67 mmol), CH_3_CN, rt, 15 min. ^a^ see Ref. [54], ^b^ enamine (*Z*)/imine (*E*:*Z*) 1/0.53 (1:0.1) ^c^ *dr* 1:1.

**Table 2:** Reduction of imines (**10,11,14a,b** and rac-**12**–**13,15**).

Entry	X	PG	Imine	Reductor	Yield [%]	Amine	*dr*
1	Br	(*S*)-MBn	**10a,b** ^a^	NaBH_3_CN/HOAc	97	**16a**–**d**	1:1:0.1:0.07
2	Br	(*S*)-MBn	**10a,b** ^a^	NaBH_3_CN/CeCl_3_	89	**16a**–**d**	1:0.98:0.1:0.06
3	Br	(*S*)-MBn	**10a,b** ^a^	NaBH_3_CN/(1*S*)-(+)CSA	86	**16a**–**d**	1:1.06:0.1:0.06
4	Br	(*S*)-MBn	**10a,b** ^a^	BH_3_^.^THF	91	**16a**–**d**	1:0.25:0:0
5	Br	(*S*)-MBn	**10a,b** ^a^	BH_3_^.^S(CH_3_)_2_	47	**16a**–**d**	1:0.20:0:0
6	Br	(*S*)-MBn	**10a,b** ^a^	NaBH_3_CN	0	**16a**–**d**	-
7	Br	(*R*)-MBn	**11a,b** ^a^	NaBH_3_CN/HOAc	95	**17a**–**d**	1:1:0.1:0.07
8	Br	PMB	**12**	NaBH_3_CN/HOAc	97	rac–**18a,b**	1:0.70
9	Br	PMB	**12**	NaBH_3_CN/CeCl_3_	85	rac–**18a,b**	1:0.50
10	Br	PMB	**12**	NaBH_3_CN/(1*S*)-(+)CSA	83	rac–**18a,b**	1:0.74
11	Br	PMP	**13**	NaBH_3_CN/HOAc	91	rac–**19a,b**	1:0.88
12	Cl	(*S*)-MBn	**14a,b** ^a^	NaBH_3_CN/HOAc	95	**20a**–**d**	1:1:0.1:0.07
13	Cl	PMB	**15**	NaBH_3_CN/HOAc	96	rac–**21a,b**	1:0.70

^a^ *dr* 1:1.

**Table 3:** Optimization of the intramolecular cyclization–solvent effect.

Entry	Substrate	Solvent	Time [h]	Aziridine	Yield [%] ^a^	*cis*/*trans* ^a^
1	**16a**–**d** ^b^	DMF	4	**24a**–**d**	82	0.78 ^f^: 1 ^g^
2	**16a**–**d** ^b^	CH_3_CN	12	**24a**–**d**	70	0.68 ^f^: 1 ^g^
3	**16a**–**d** ^b^	PhCH_3_	24	**24a**–**d**	*Traces*	-
4	**16a**–**d** ^b^	CH_2_Cl_2_	24	**24a**–**d**	*Traces*	-
5	**16a**–**d** ^b^	THF	24	**24a**–**d**	15	ca. 0: 1
6	**16a**–**d** ^b^	DMC	8	**24a**–**d**	32	ca. 0: 1
7	rac-**18a**,**b** ^c^	DMF	4	rac-**25a**,**b**	79	0.68: 1
8	rac-**19a,b** ^d^	DMF	4	rac-**26a**,**b**	48	0.09: 1
9	**20a**–**d** ^e^	DMF	18	**24a**,**b**	*Traces*	-

Reaction conditions: amine (0.2 mmol), TEA (0.3 mmol), solvent, 70 °C, 4–24 h, Schlenk flask, inert gas. ^a^ determined by ^19^F and/or ^31^P NMR spectra from crude mixture; ^b^ *dr* 1:1:0.1:0.07; ^c^ *dr* 1:0.72; ^d^ *dr* 1:0.88; ^e^ *dr* 1:1:0.1:0.07; ^f^ *dr* 1:0.13; ^g^ *dr* 1:0.08.

**Table 4:** Optimization of the intramolecular cyclization–base effect.

Entry	Substrate	Base	Yield [%] ^a^	Aziridine	*cis*/*trans* ^a^
1	**16a**–**d** ^b^	TEA	82	**24a**–**d**	0.78 ^e^: 1 ^f^
2	**16a**–**d** ^b^	Quinine	84	**24a**–**d**	0.86 ^e^: 1 ^f^
3	**16a**–**d** ^b^	L-Proline	77	**24a**–**d**	0.69 ^e^: 1 ^f^
4	**16a**–**d** ^b^	DBU	21 ^d^	**24a**–**d**	0.18 ^e^: 1 ^f^
5	**16a**–**d** ^b^	DIPEA	70	**24a**–**d**	0.67 ^e^: 1 ^f^
6	**16a**–**d** ^b^	NaH	<10	**24a**–**d**	-
7	**16a**–**d** ^b^	w/o base	38	**24a**–**d**	0.38 ^g^: 1 ^h^
8	rac-**18a,b** ^c^	TEA	79	rac-**25a,b**	0.68: 1
9	rac-**18a,b** ^c^	Quinine	87	rac-**25a,b**	0.92: 1
10	rac-**18a,b** ^c^	L-Proline	69	rac-**25a,b**	0.61: 1

Reaction conditions: amine (0.2 mmol), base (0.3 mmol), DMF, 70 °C, 4 h, Schlenk flask, inert gas ^a^ determined by ^19^F and/or ^31^P NMR spectra from crude mixture; ^b^ *dr* 1:1:0.1:0.07; ^c^ *dr* 1:0.72; ^d^ **22**–**23a,b** were mainly formed (*E-***22a**/*Z-***22b** ratio 1:0.3; **22a,b**/**23a,b** ratio 1:0.05); ^e^ *dr* 1:0.13; ^f^ *dr* 1:0.08;^g^ *dr* 1:0.06; ^h^ *dr* 1:0.03.

**Table 5:** Characteristic NMR values for rac-**25a,b**.

	rac-*cis-***25a**	rac-*trans-***25b**
^19^F NMR	−180.27 (dd, ^2^*J*_FP_ 116.7, ^3^*J*_FH_ 8.4 Hz)	−170.98 (dq, ^2^*J*_FP_ 111.7, ^3^*J*_FH_ 4.3 Hz)
^31^P{/^1^H} NMR	9.97 (d, ^2^*J*_PF_ 116.9 Hz)	10.95 (d, ^2^*J*_PF_ 111.6 Hz)
NC*H*CFP	3.26 (d, ^3^*J*_HF_ 8.5 Hz)	3.45 (t, ^3^*J*_HF/P_ 4.1 Hz)
*C*FP	86.67 (dd, ^1^*J*_CF/P_ 274.0, 272.5 Hz)	83.95 (dd, ^1^*J*_CF/P_ 259.6, 231.6 Hz)
*C*H(Ph)CFP	49.08 (dd, ^2^*J*_CF/P_ 19.2, 1.3 Hz)	48.43 (dd, ^2^*J*_CF/P_ 12.9, 6.0 Hz)
*C_ipso_*	133.86 (s)	133.58 (br d, ^3^*J*_CF_ 5.2 Hz)

## Data Availability

All data are provided in the manuscript.

## Supplementary Materials

The Supplementary Materials can be downloaded at: https://www.mdpi.com/article/10.3390/molecules28145579/s1.

## Sample Availability

Samples of the compounds are available from the authors.
