# Supplementary material for: Access to 2-Fluorinated Aziridine-2-phosphonates from α,α-Halofluorinated β-Iminophosphonates—Spectroscopic and Theoretical Studies
Source: Molecules. 2023 Jul 22;28(14):5579. doi: 10.3390/molecules28145579 (PMC10385471; doi:10.3390/molecules28145579)
Supplement: Supplementary file 1 [file molecules-28-05579-s001.zip › molecules-2512014-supplementary.pdf]

Supporting Informational

Access to 2-fluorinated aziridine-2-phosphonates from *α,α*-halofluorinated *β*-iminophosphonates – spectroscopic and theoretical studies

Mateusz Klarek[a], Tomasz Siodła[a], Tahar Ayad[b], David Virieux[b], Magdalena Rapp[a]

[a] *Faculty of Chemistry, Adam Mickiewicz University in Poznan, Poland*
[b] *ICGM Univ. Montpellier, CNRS, ENSCM, Montpellier, France*

Table of contents

I. Spectra of compounds **3**

β-Enaminophosphonates/ β-iminophosphonates ***5a/9a,b*** 3

(*Z*)-Diethyl (2-((4-methoxyphenyl)amino)-2-phenylvinyl)phosphonate **(*Z*-5a)** 3

(*E*)-Diethyl (2-((4-methoxyphenyl)imino)-2-phenylethyl)phosphonate **(*E*-9a)** 3

α,α-Bromofluoro-β-iminophosphonates ***10-11a,b, rac-12-13*** 5

(*E*)-Diethyl ((1*R*/1*S*)-1-bromo-1-fluoro-2-phenyl-2-(((*S*)-1-phenylethyl)imino)ethyl) phosphonate **10a,b** 5

(*E*)-Diethyl ((1*S*)-1-bromo-1-fluoro-2-phenyl-2-(((*S*)-1-phenylethyl)imino)ethyl) phosphonate **10b** 7

(*E*)-Diethyl ((1*S*/1*R*)-1-bromo-1-fluoro-2-phenyl-2-(((*R*)-1-phenylethyl)imino)ethyl) phosphonate **11a,b** 9

rac-(*E*)-Diethyl ((1*R*/1*S*)-1-bromo-1-fluoro-2-((4-methoxybenzyl)imino)-2-phenylethyl)phosphonate rac-**12** 11

rac-(*E*)-Diethyl ((1*R*/1*S*)-1-bromo-1-fluoro-2-((4-methoxyphenyl)imino)-2-phenylethyl)phosphonate rac-**13** 13

α,α-Chlorofluoro-β-iminophosphonates ***14a,b,*** *rac-**15*** 15

(*E*)-Diethyl ((1*R*/1*S*)-1-chloro-1-fluoro-2-phenyl-2-(((*S*)-1-phenylethyl)imino)ethyl)phosphonate **14a,b** 15

rac-(*E*)-Diethyl (1*R*/1*S*)-1-chloro-1-fluoro-2-((4-methoxybenzyl)imino)-2-phenylethyl)phosphonate rac-**15** 19

α,α-Bromofluoro-β-aminophosphonates ***16-17a-d,*** *rac-**18-19a,b*** 21

Diethyl ((1*S*/*R*, 2*S*/*R*)-1-bromo-1-fluoro-2-phenyl-2-(((*S*)-1-phenylethyl)amino)ethyl)phosphonate **16a-d** 21

Diethyl ((1*S*/1*R*, 2*S*/2*R*)-1-bromo-1-fluoro-2-phenyl-2-(((*R*)-1-phenylethyl)amino)ethyl)phosphonate **17a-d** 23

rac-Diethyl ((1*R*/1*S*, 2*R/*2*S*)-1-bromo-1-fluoro-2-((4-methoxybenzyl)amino)-2-phenylethyl)phosphonate rac-**18a,b** 26

rac-Diethyl ((1*R*/1*S*, 2*R*/2*S*)-1-bromo-1-fluoro-2-((4-methoxyphenyl)amino)-2-phenylethyl)phosphonate rac-**19a,b** 28

*α,α-Chlorofluoro-β-iminophosphonates **20a-d**, rac-**21a,b*** 30

Diethyl((1*S*/1*R*, 2*S*/2*R*)-1-chloro-1-fluoro-2-phenyl-2-(((*S*)-1-phenylethyl)amino)ethyl)phosphonate **20a-d** 30

rac-Diethyl ((1*S*/1*R*, 2*S*/2*R*)-)-1-chloro-1-fluoro-2-((4-methoxybenzyl)amino)-2-phenylethyl)phosphonate rac-**21a,b** 32

*α-Fluorinated β-enaminophosphonate/ β-iminophosphonate **22a,b/23a,b*** 34

(*Z*)-Diethyl(1-fluoro-2-phenyl-2-(((*S*)-1-phenylethyl)amino)vinyl)phosphonate **22a** 34

*2-Fluorinated aziridine-2-phosphonates **24a-d, rac-25-26a,b*** 36

Diethyl ((2*S*/2*R*, 3*R*/3*S*)-2-fluoro-3-phenyl-1-((*S*)-1-phenylethyl)aziridin-2-yl)phosphonate **24a-d** 36

Diethyl ((2*R*,3*R*)-2-fluoro-3-phenyl-1-((*S*)-1-phenylethyl)aziridin-2-yl)phosphonate **(*trans*-24b)** 40

rac-Diethyl ((2*S*/2*R*, 3*R*/3*S*)-2-fluoro-1-(4-methoxybenzyl)-3-phenylaziridin-2-yl)phosphonate rac-**25a,b** 44

rac-Diethyl ((2*R*,3*R*)-2-fluoro-1-(4-methoxybenzyl)-3-phenylaziridin-2-yl)phosphonate rac-***trans*-25b** 48

rac-Diethyl ((2*S*/2*R*, 3*R*/3*S*)-2-fluoro-1-(4-methoxyphenyl)-3-phenylaziridin-2-yl)phosphonate rac-***trans*-26a,b** 53

*Non-fluorinated aziridine-2-phosphonate **cis-27*** 56

Diethyl ((2*S*,3*R*)-3-phenyl-1-((*S*)-1-phenylethyl)aziridin-2-yl)phosphonate ***cis*-27** 56

*α-Fluorinated β-aminophosphonate **28a-d*** 60

Diethyl ((1*S*/*R*, 2*S*/*R*)-1-fluoro-2-phenyl-2-(((*S*)-1-phenylethyl)amino)ethyl)phosphonate **28a-d** 60

*Aziridine ring opening product **29a,b*** 62

Diethyl ((1*R*/1*S*, 2*S*)-1,2-dimethoxy-2-phenyl-1-(((*S*)-1-phenylethyl)amino)ethyl)phosphonate **29a,b** 62

II. NMR table 65

III. Geometries optimization – theoretical data 66

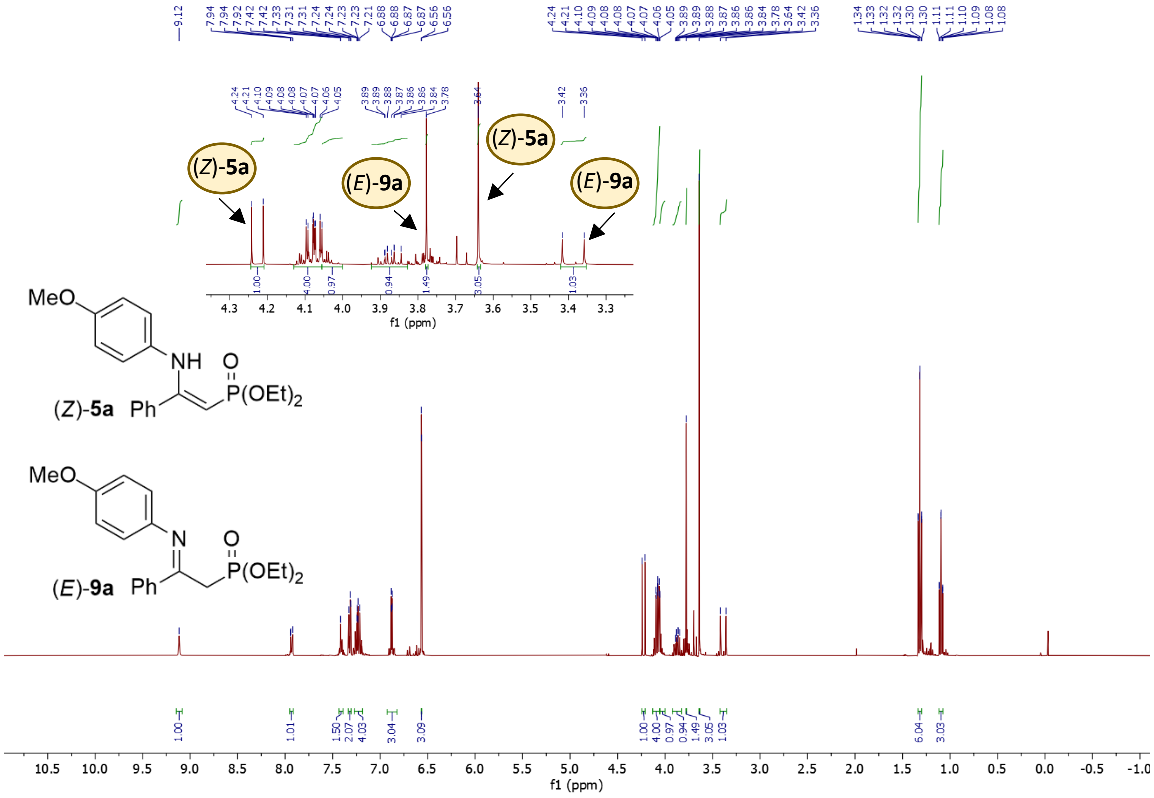

^{1}H NMR of enamine (**5a**)/imines (**9a,b**), ratio 1:0.53. (*E*)-**9a**/(*Z*)-**9b** ratio 1:0.08.

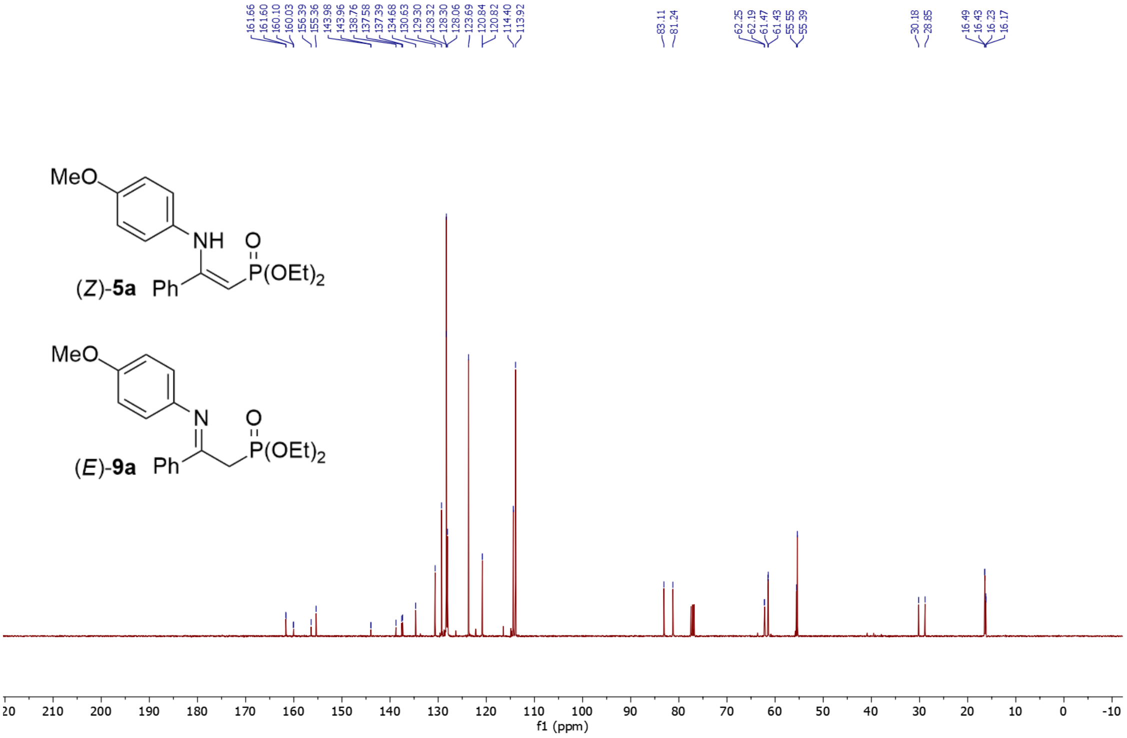

^{13}C NMR of enamine (**5a**)/imines (**9a,b**), ratio 1:0.53. (*E*)-**9a**/(*Z*)-**9b** ratio 1:0.08.

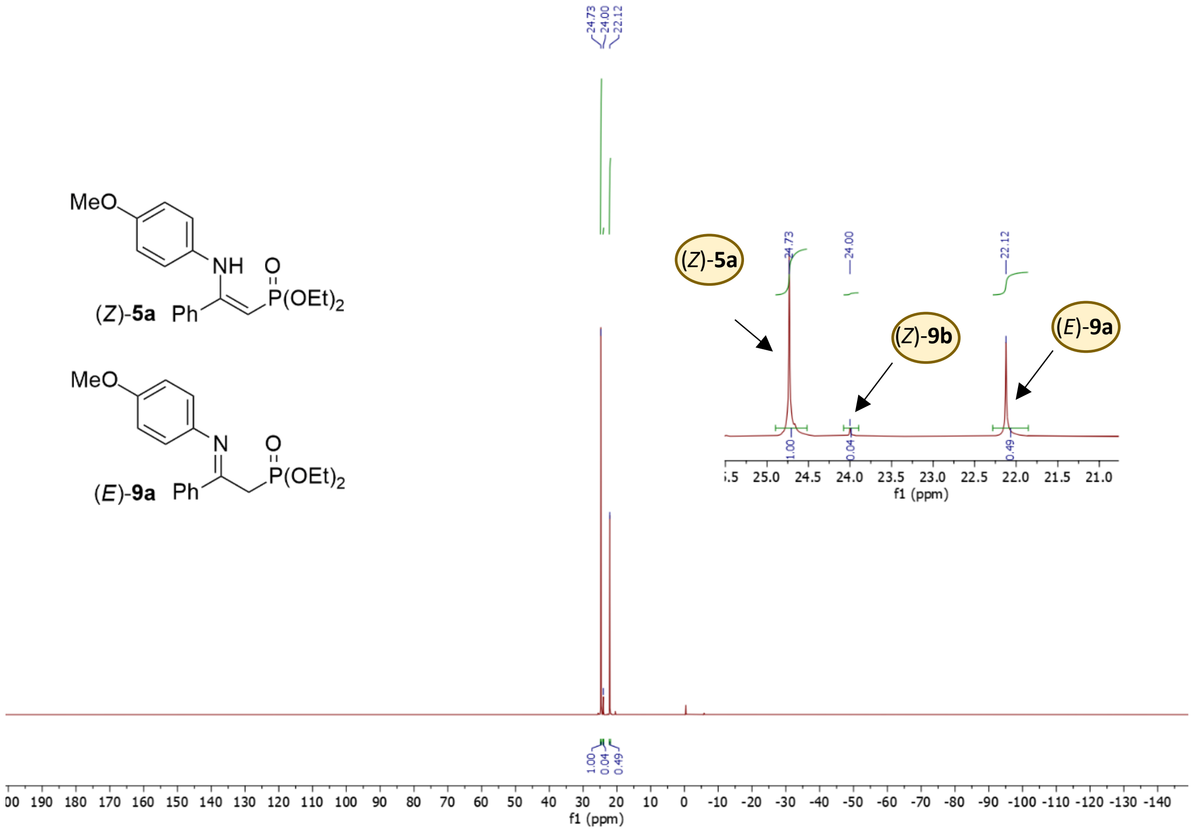

$^{31}P\{/^{1}H\}$ NMR of enamine (**5a**)/imines (**9a,b**), ratio 1:0.53. (*E*)-**9a**/(*Z*)-**9b** ratio 1:0.08.

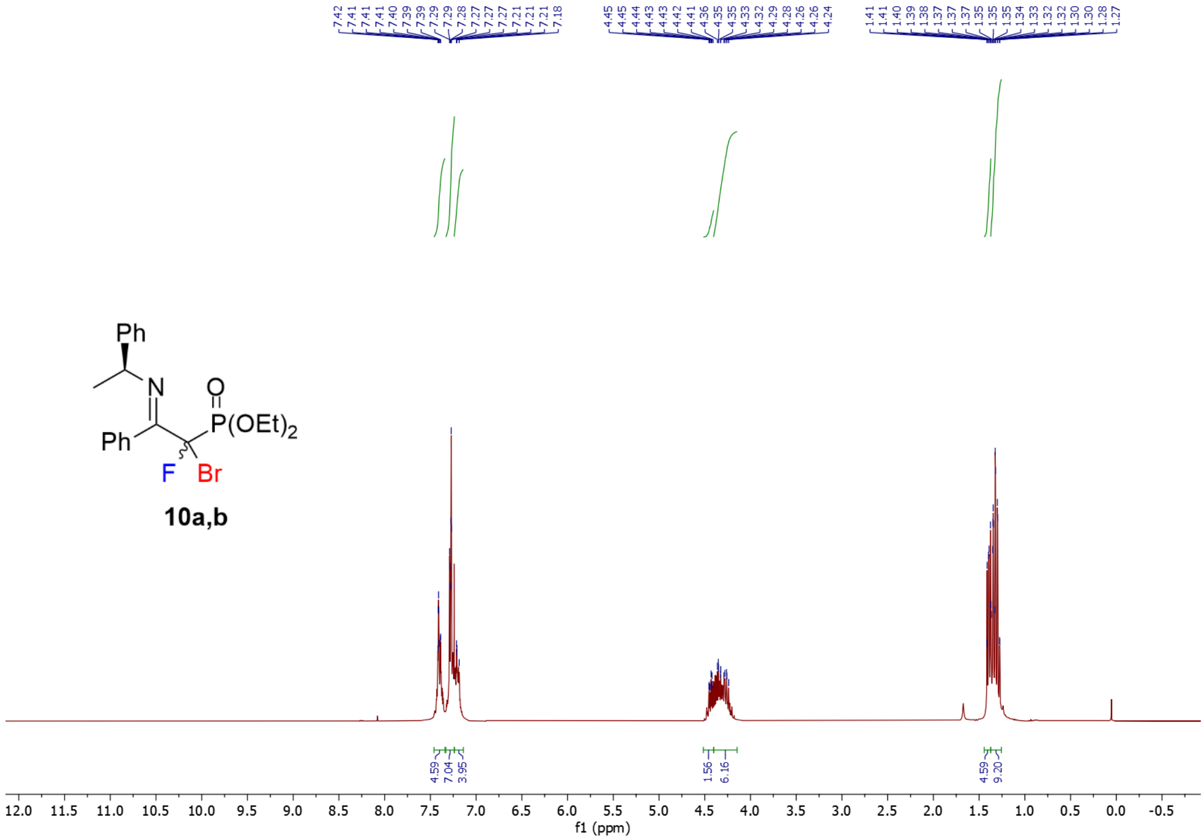

^{1}H NMR of **10a,b** *dr* = 1:0.54

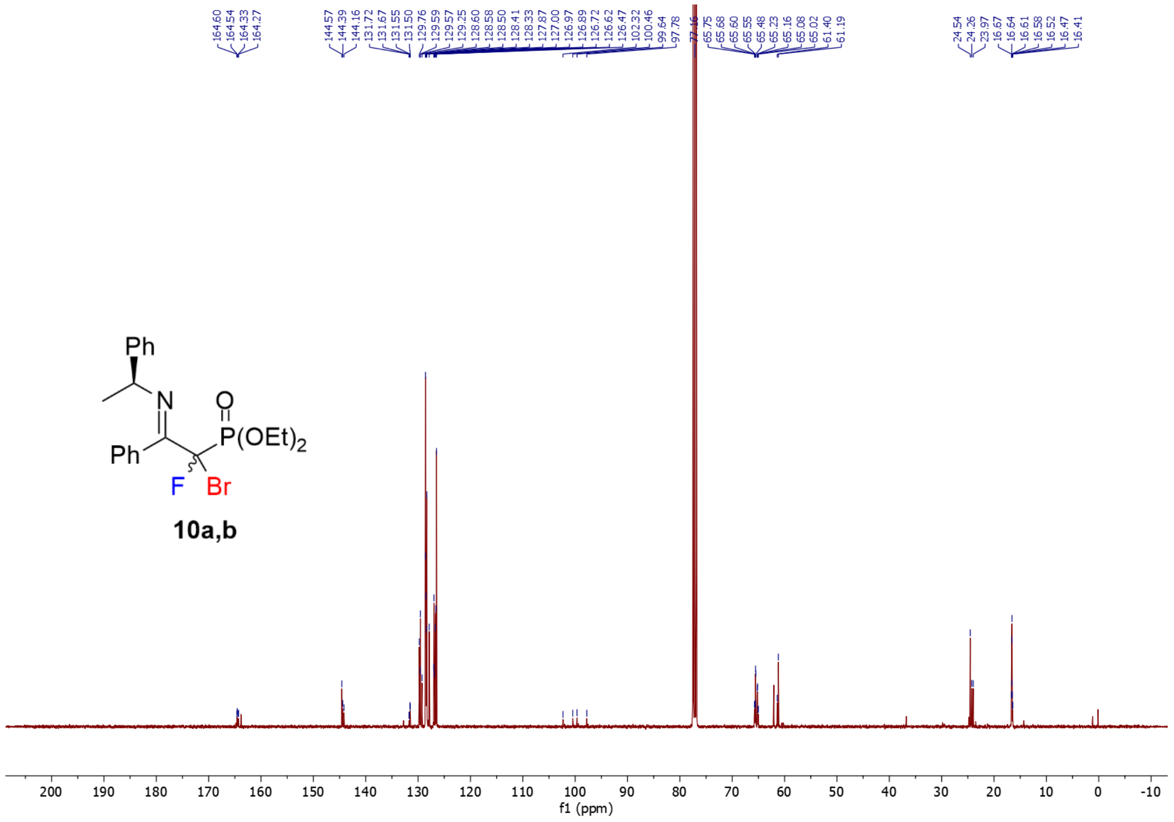

^{13}C NMR of **10a,b** *dr* = 1:0.54.

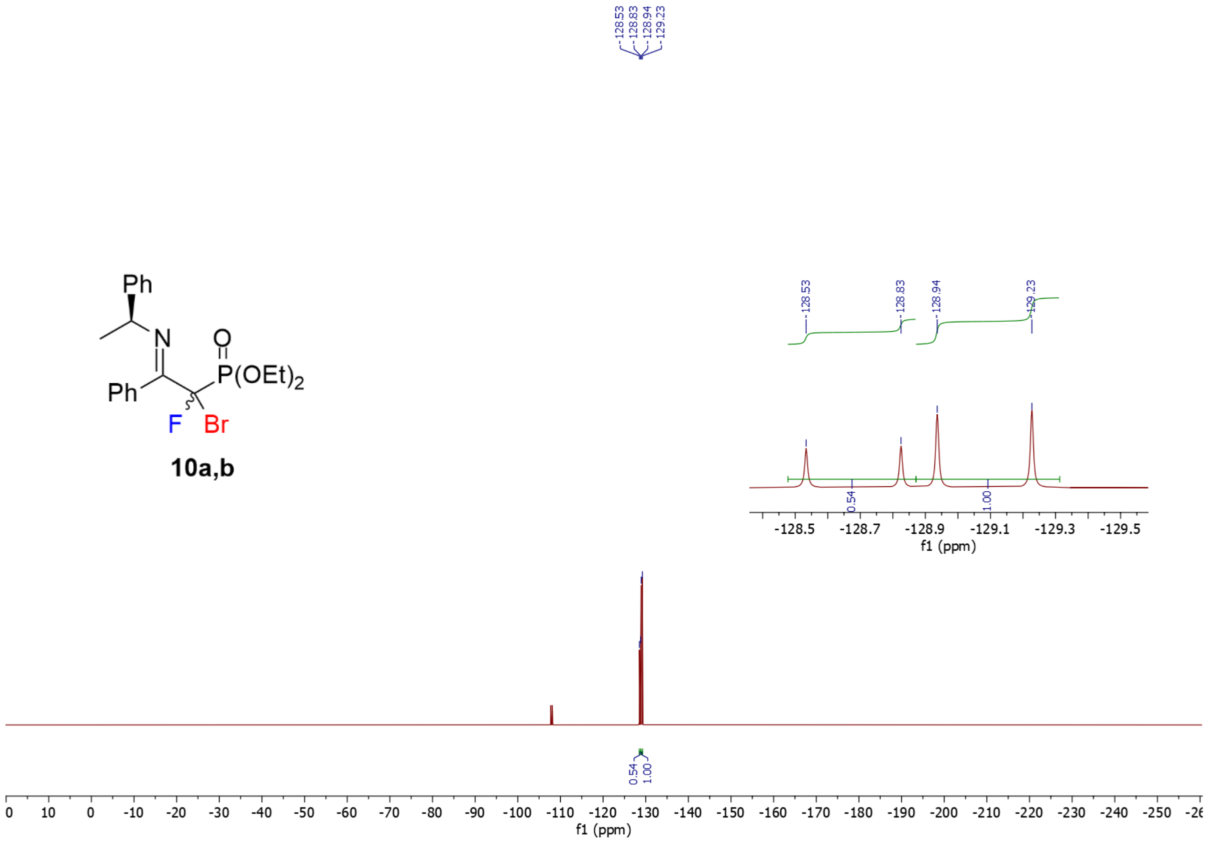

^{19}F NMR of **10a,b** *dr* = 1:0.54.

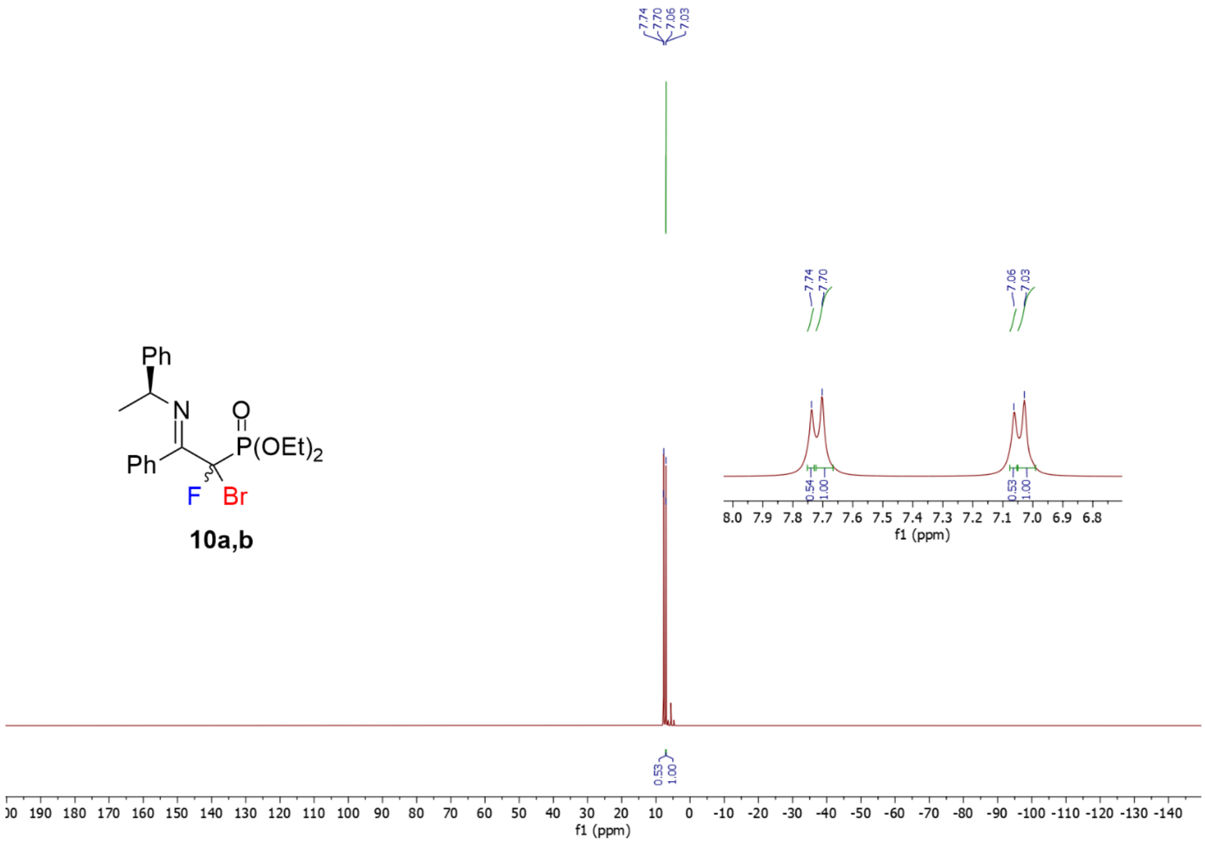

^{31}P{/^{1}H} NMR of **10a,b** *dr* = 1:0.54.

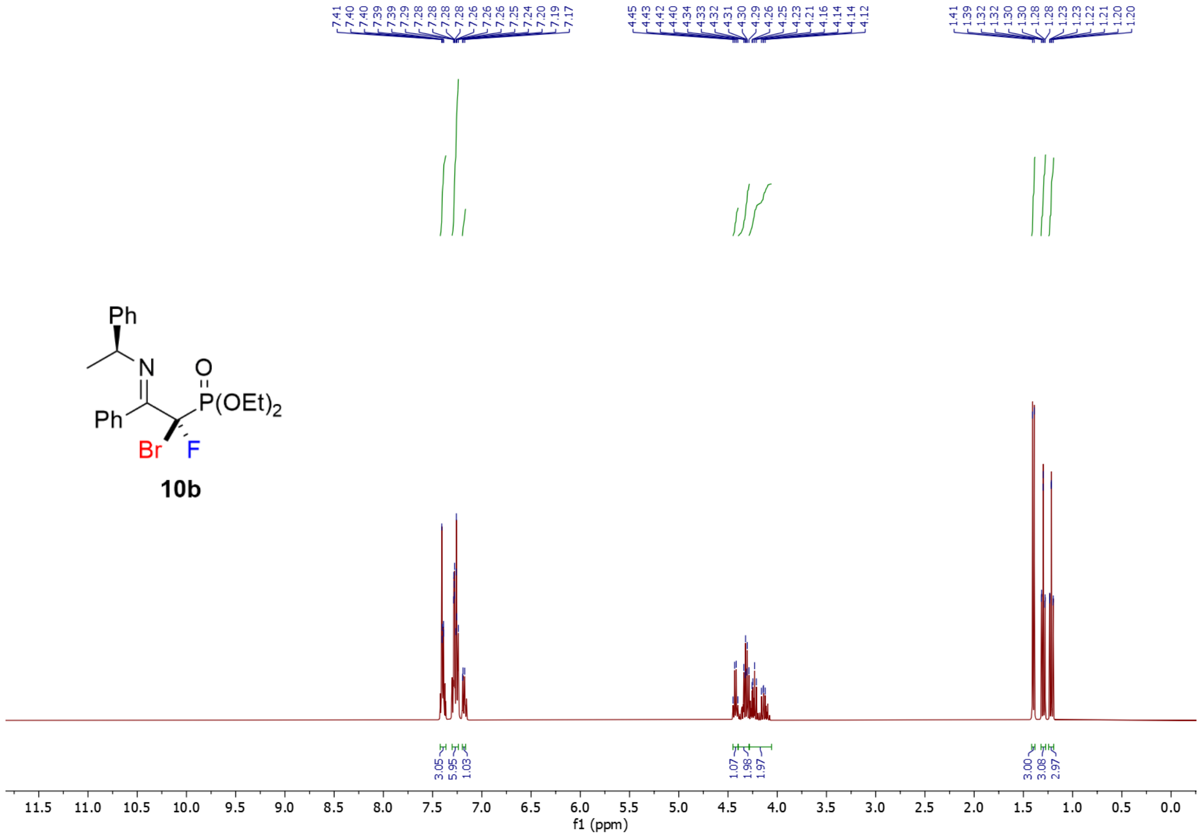

^{1}H NMR of **10b**

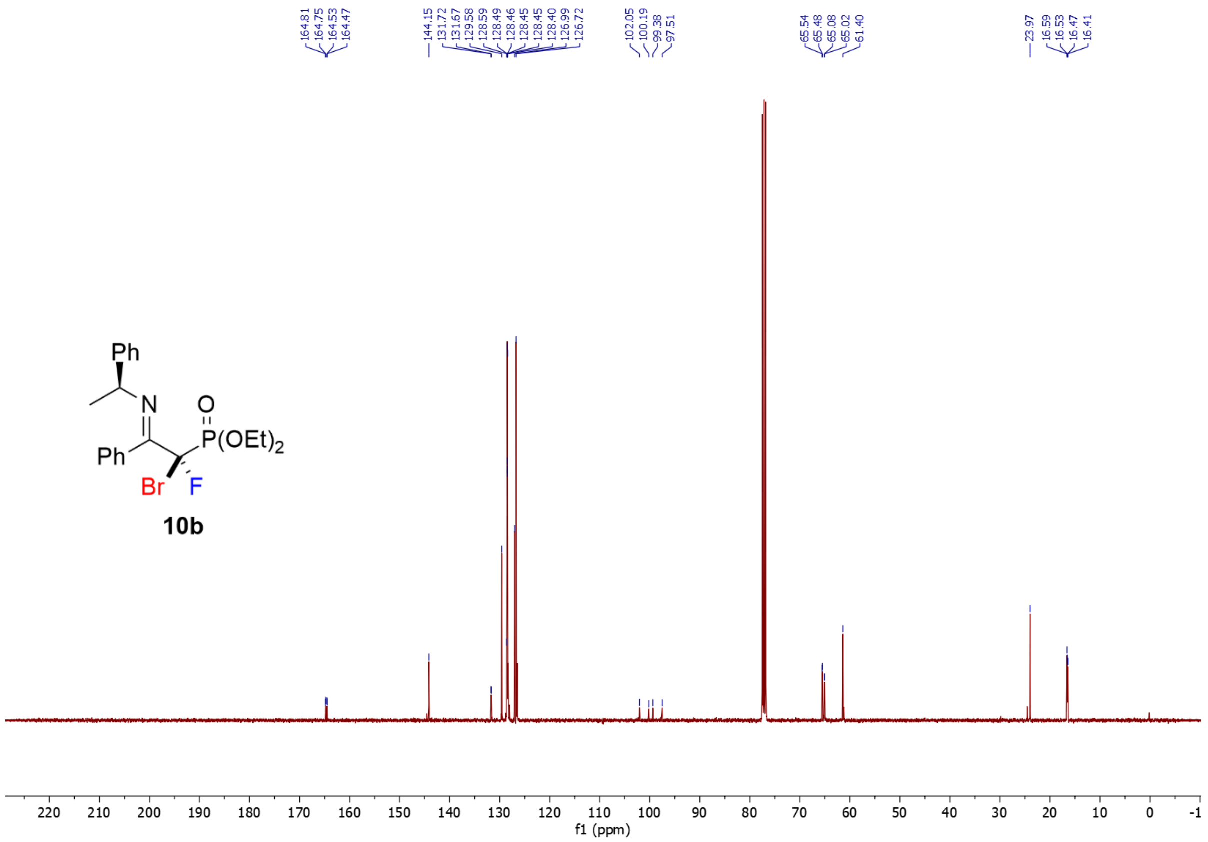

^{13}C NMR of **10b**

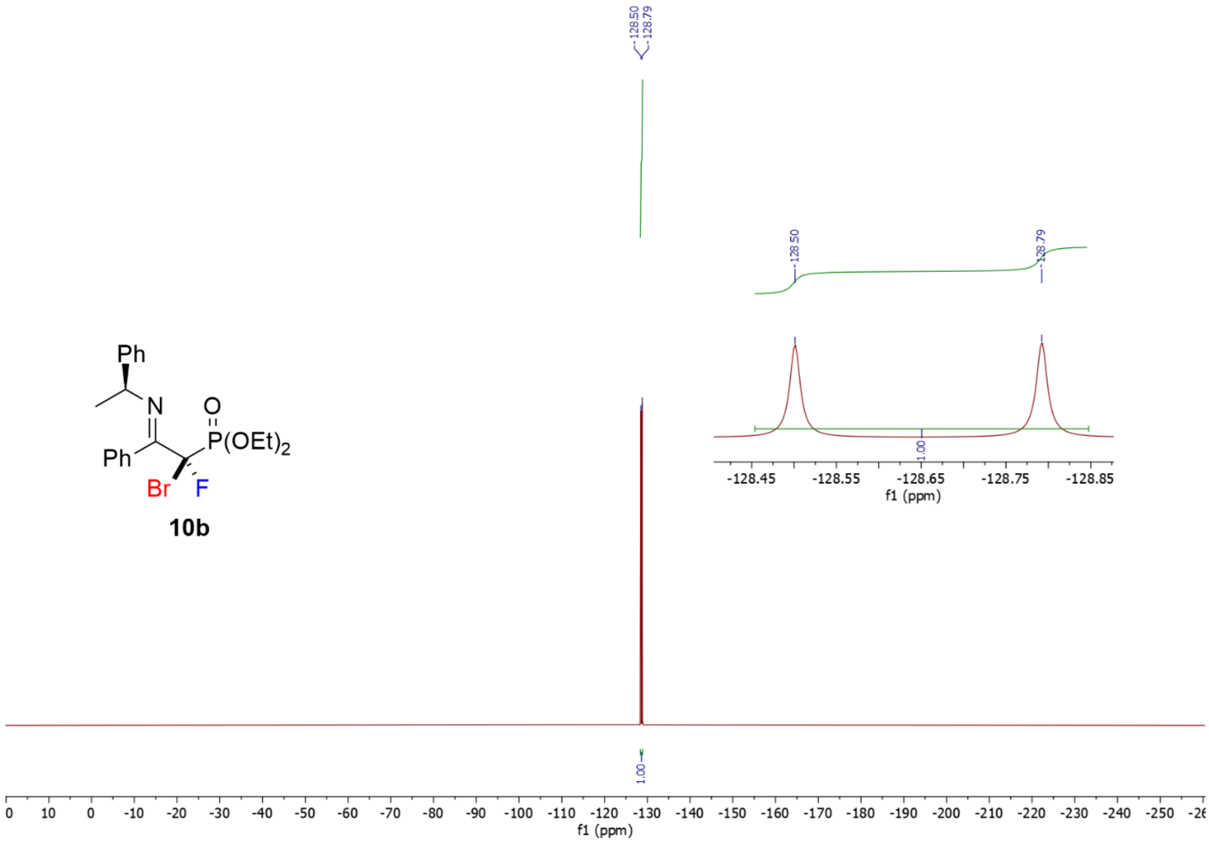

^{19}F NMR of **10b**

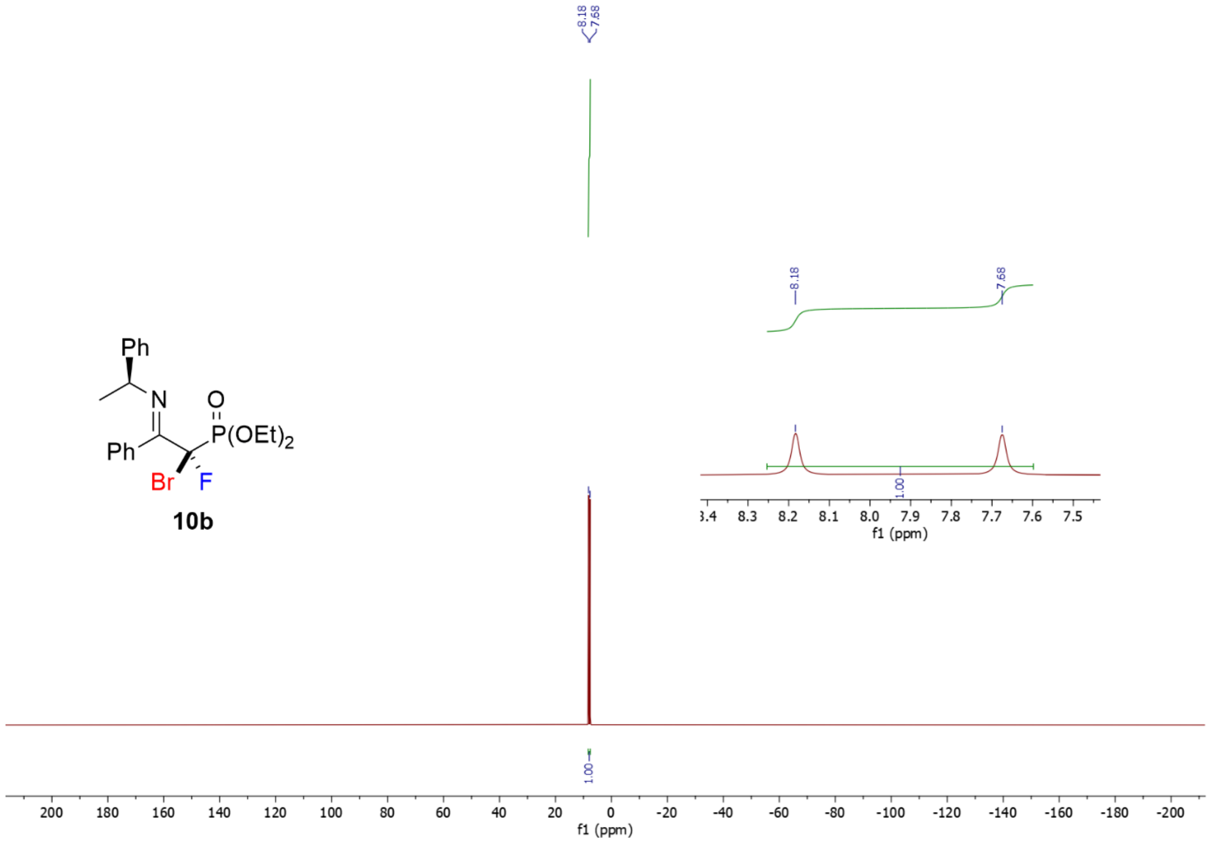

^{31}P{/^{1}H} NMR of **10b**

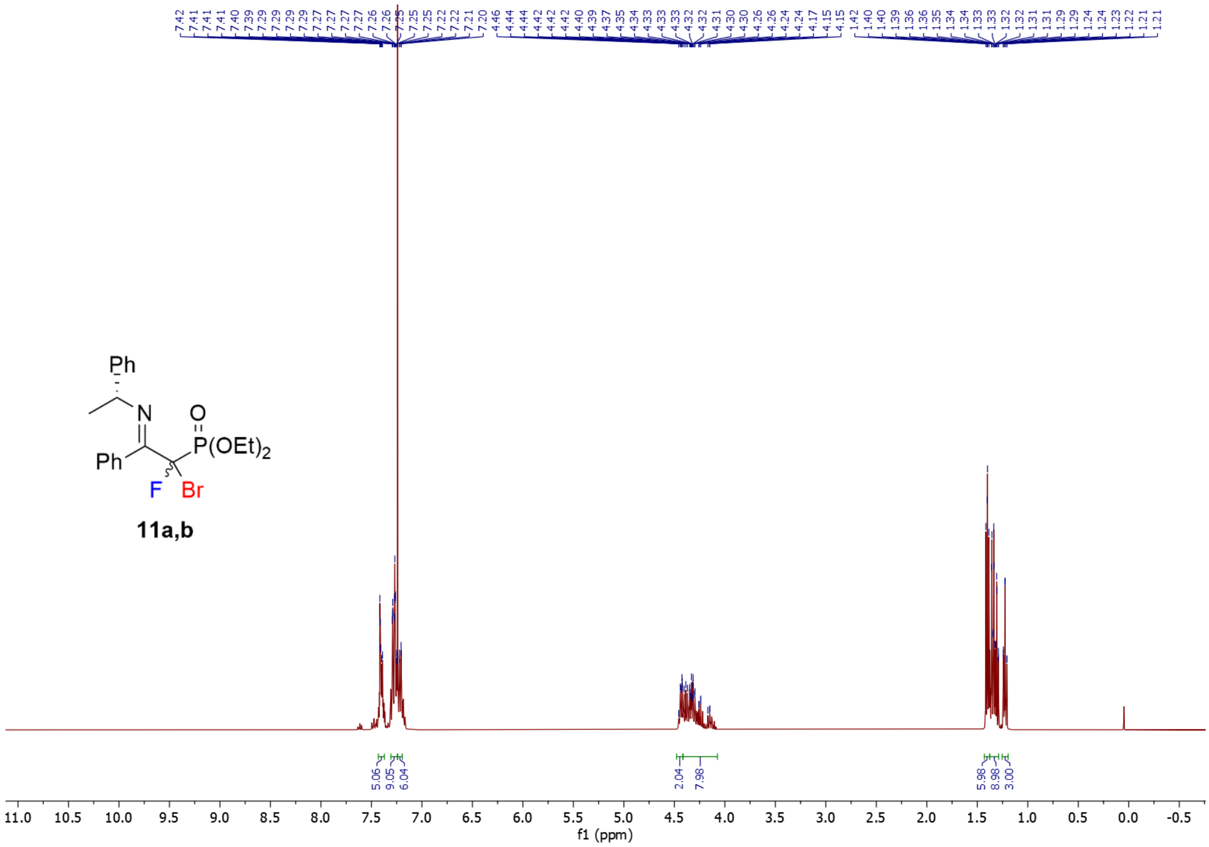

^{1}H NMR of **11a,b** *dr* = 1:0.93.

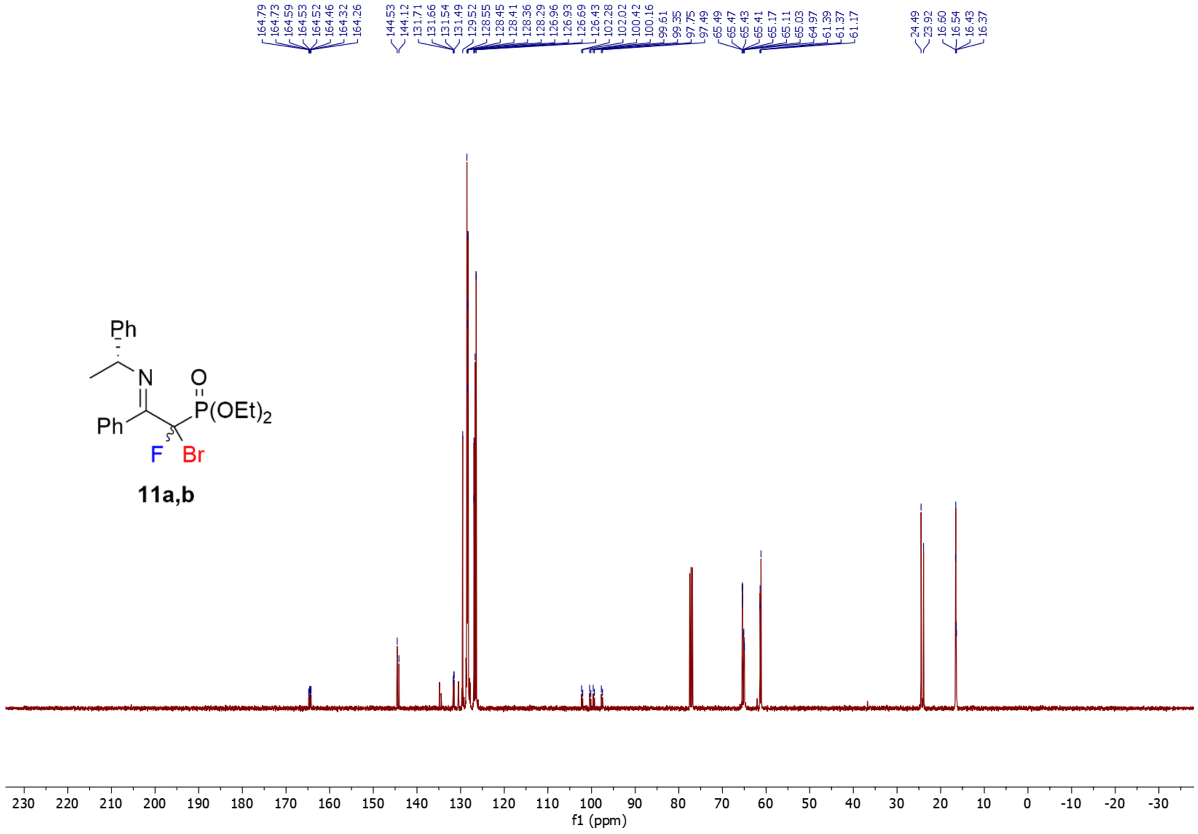

^{13}C NMR of **11a,b** *dr* = 1:0.93.

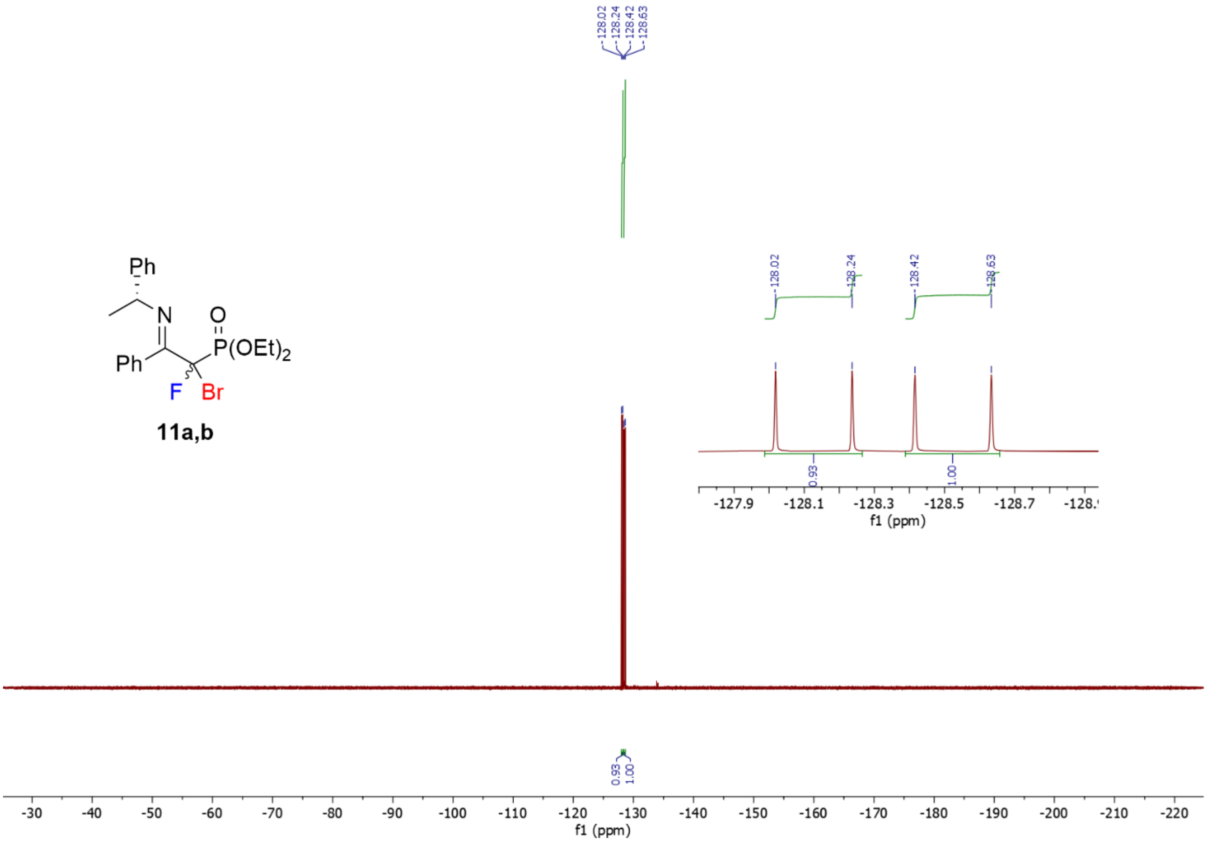

^{19}F NMR of **11a,b** *dr* = 1:0.93.

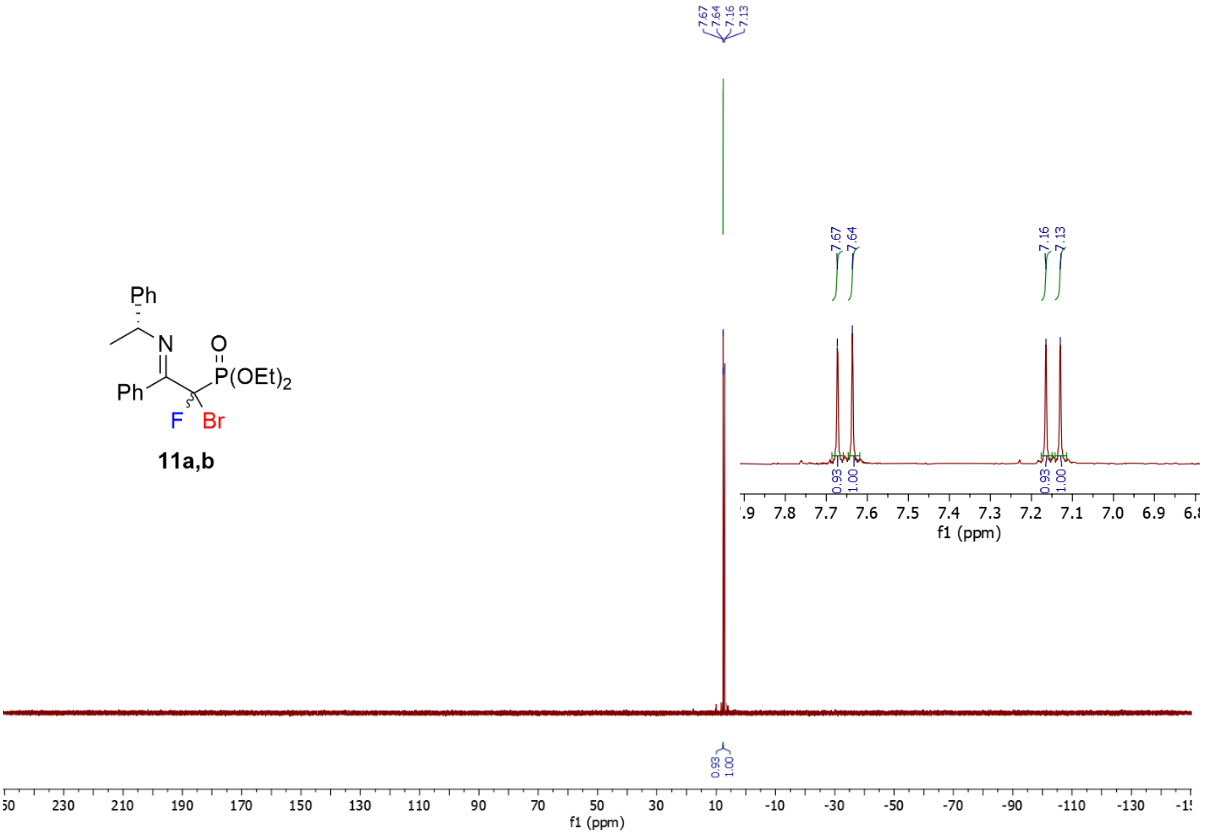

^{31}P{/^{1}H} NMR of **11a,b** *dr* = 1:0.93.

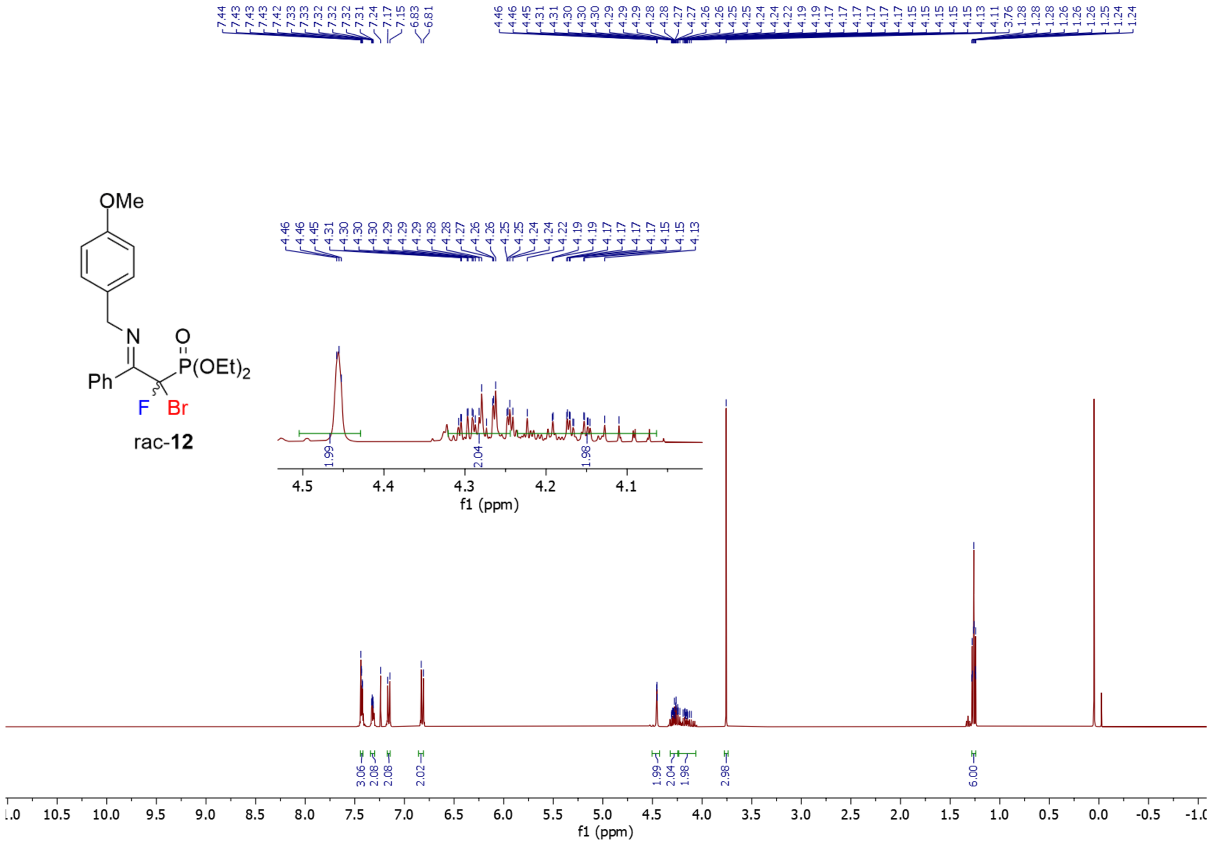

^{1}H NMR of rac-**12**

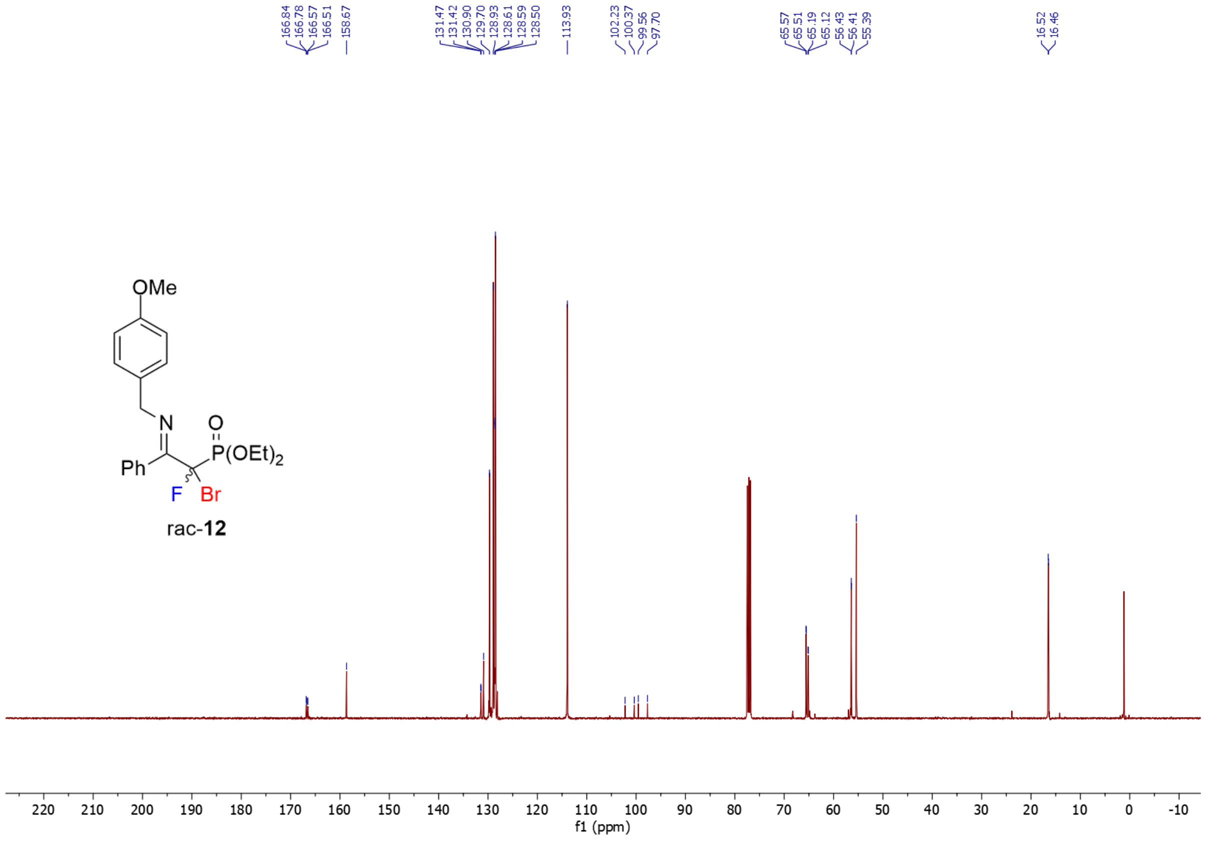

^{13}C NMR of rac-**12**

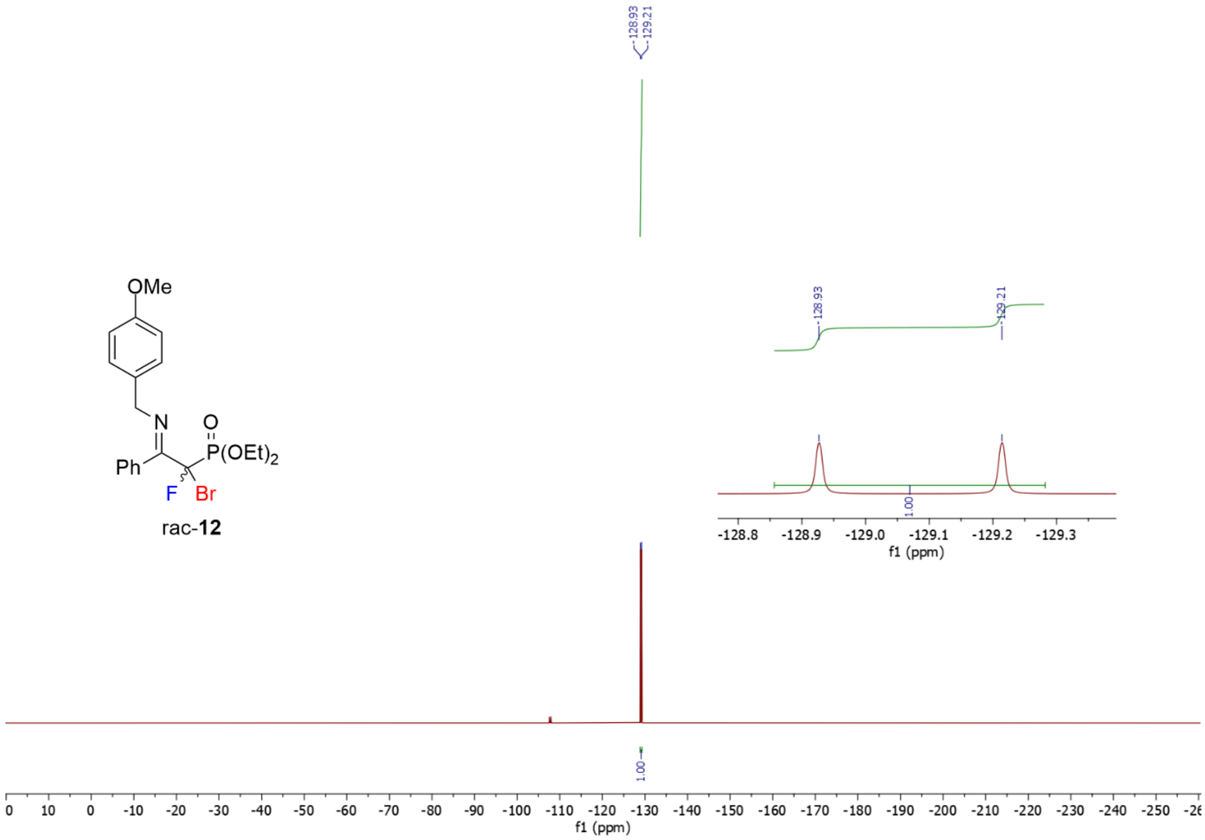

^{19}F NMR of rac-**12**

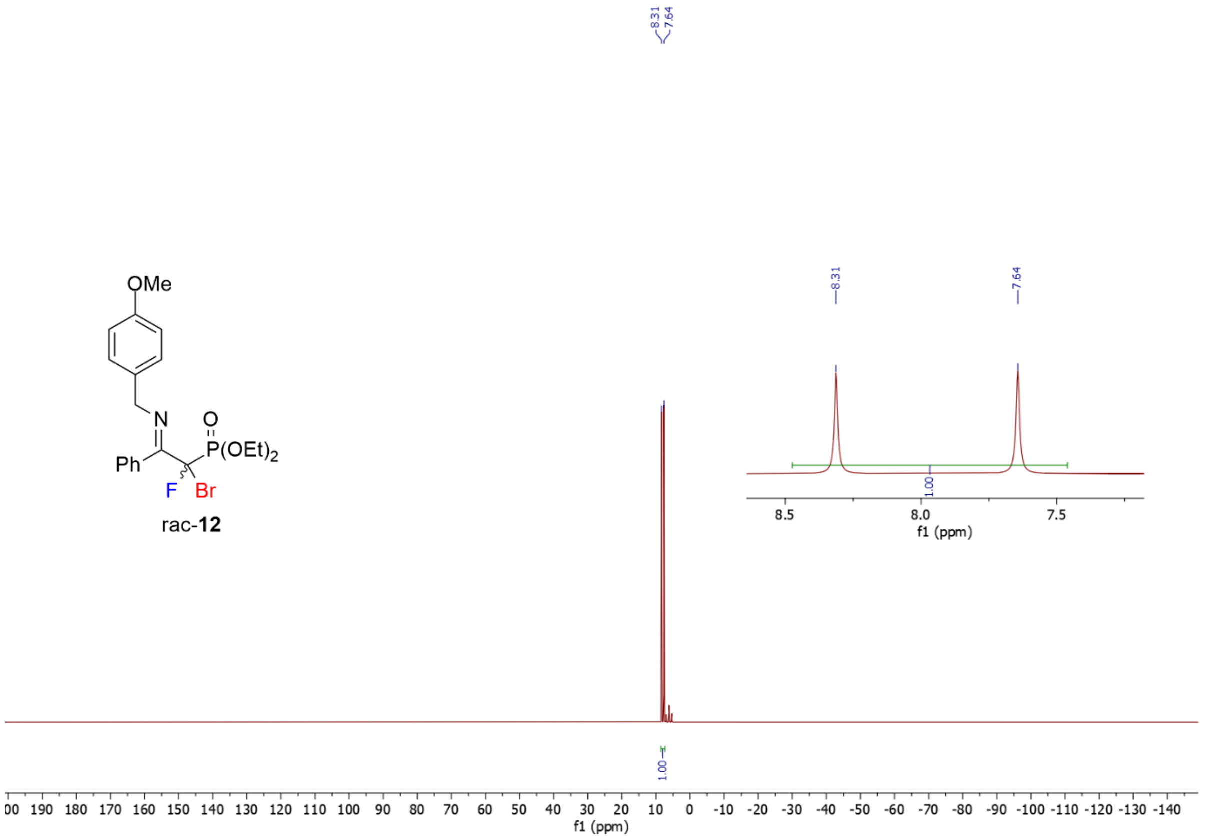

^{31}P{/^{1}H} NMR of rac-**12**

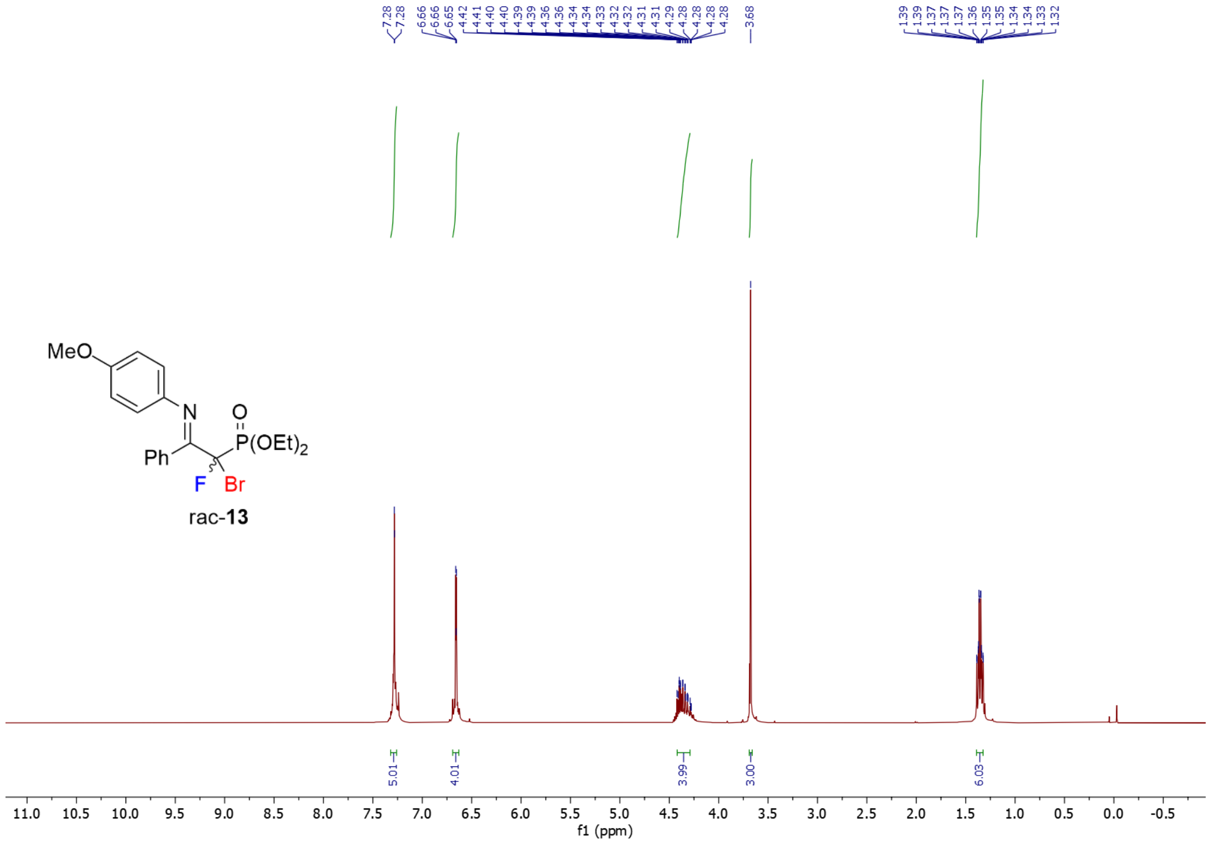

^{1}H NMR of rac-**13**

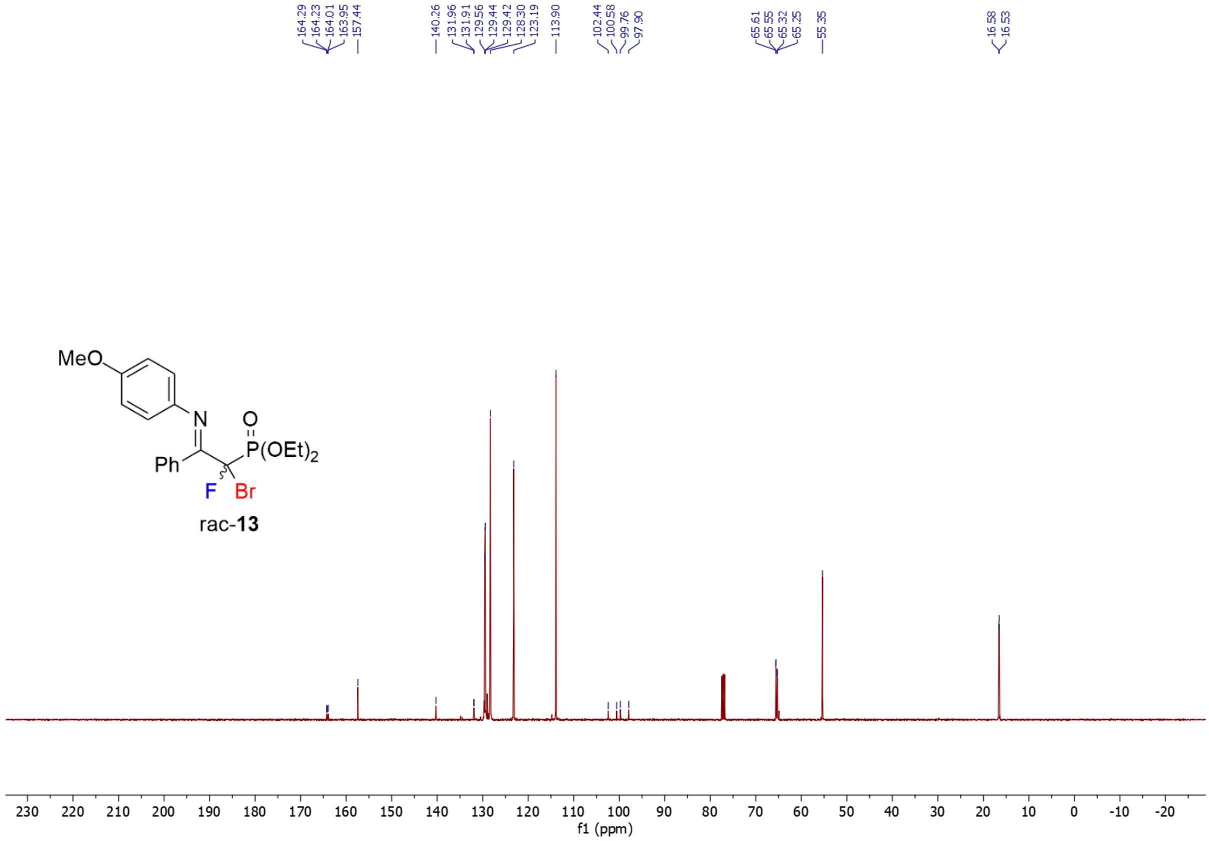

^{13}C NMR of rac-**13**

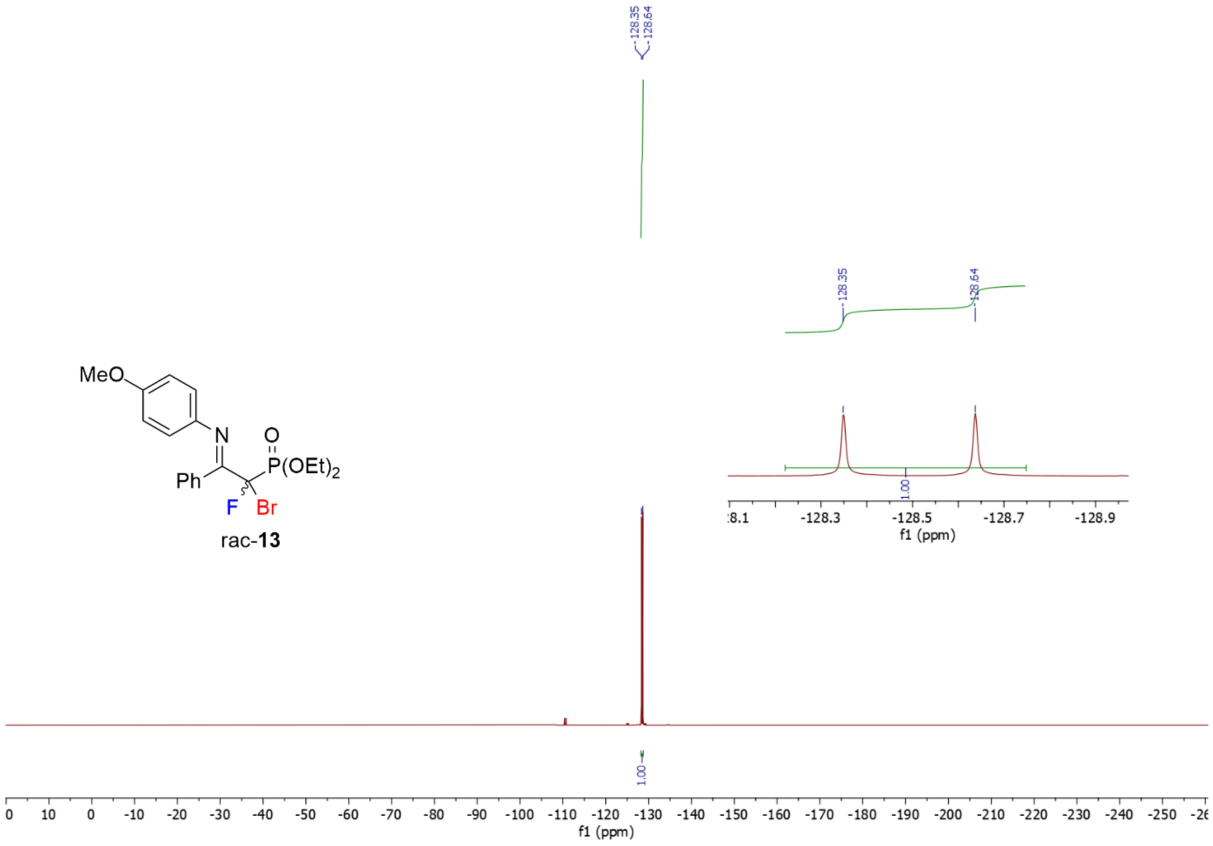

^{19}F NMR of rac-**13**

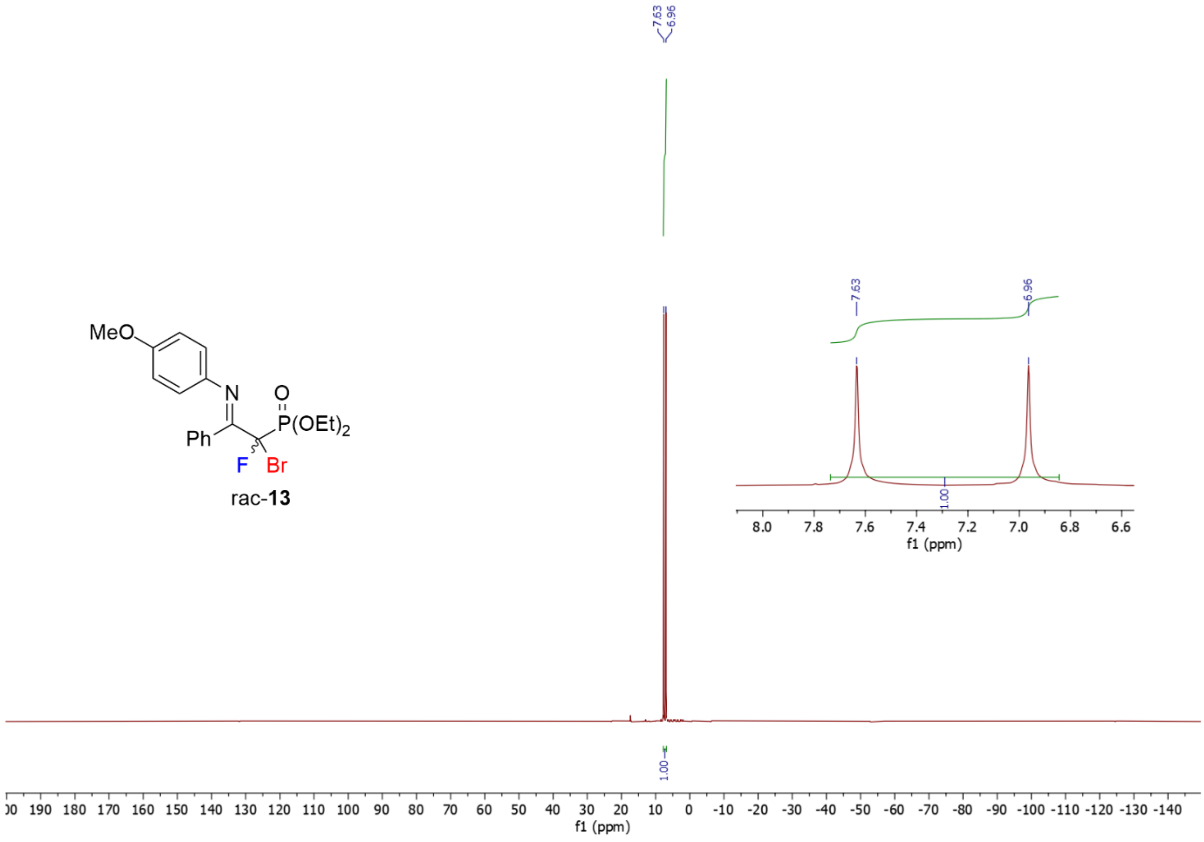

^{31}P{/^{1}H} NMR of rac-**13**

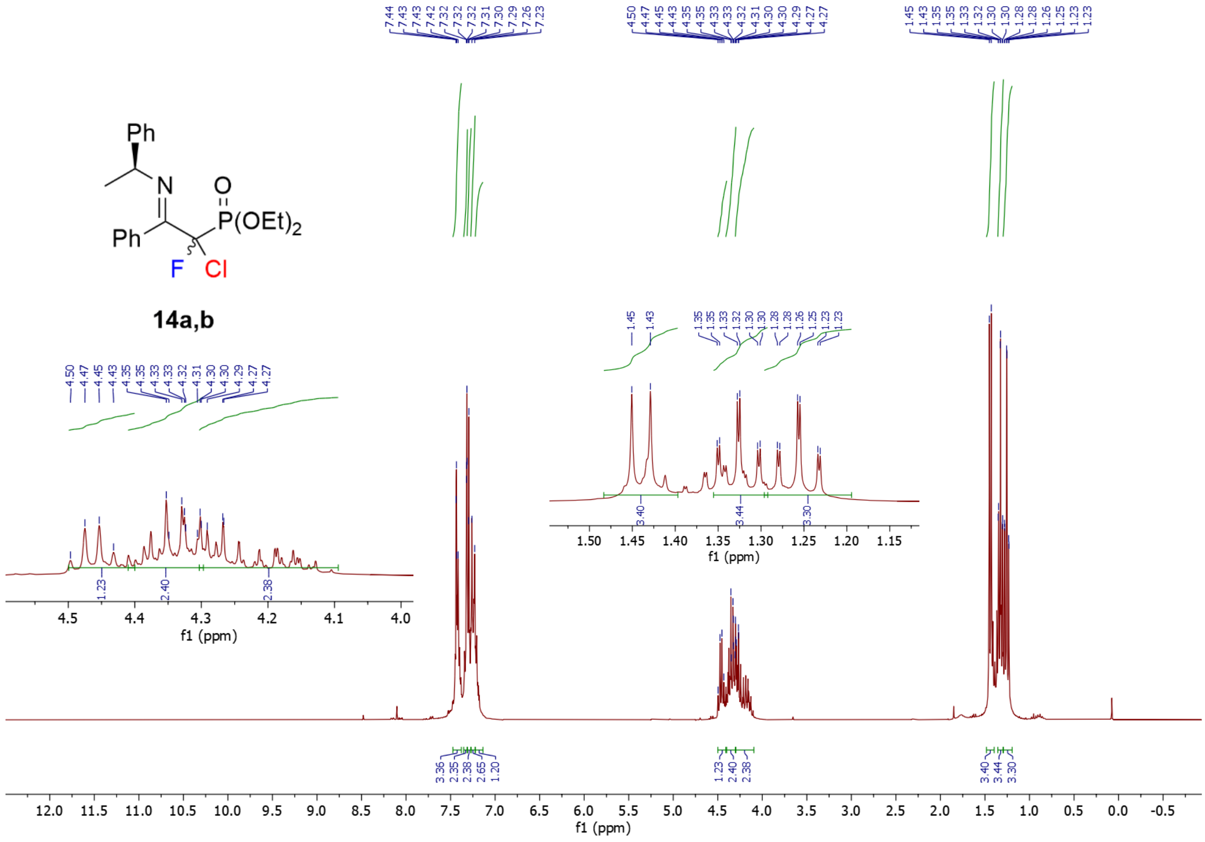

^{1}H NMR of **14a,b** *dr* = 0.2:1

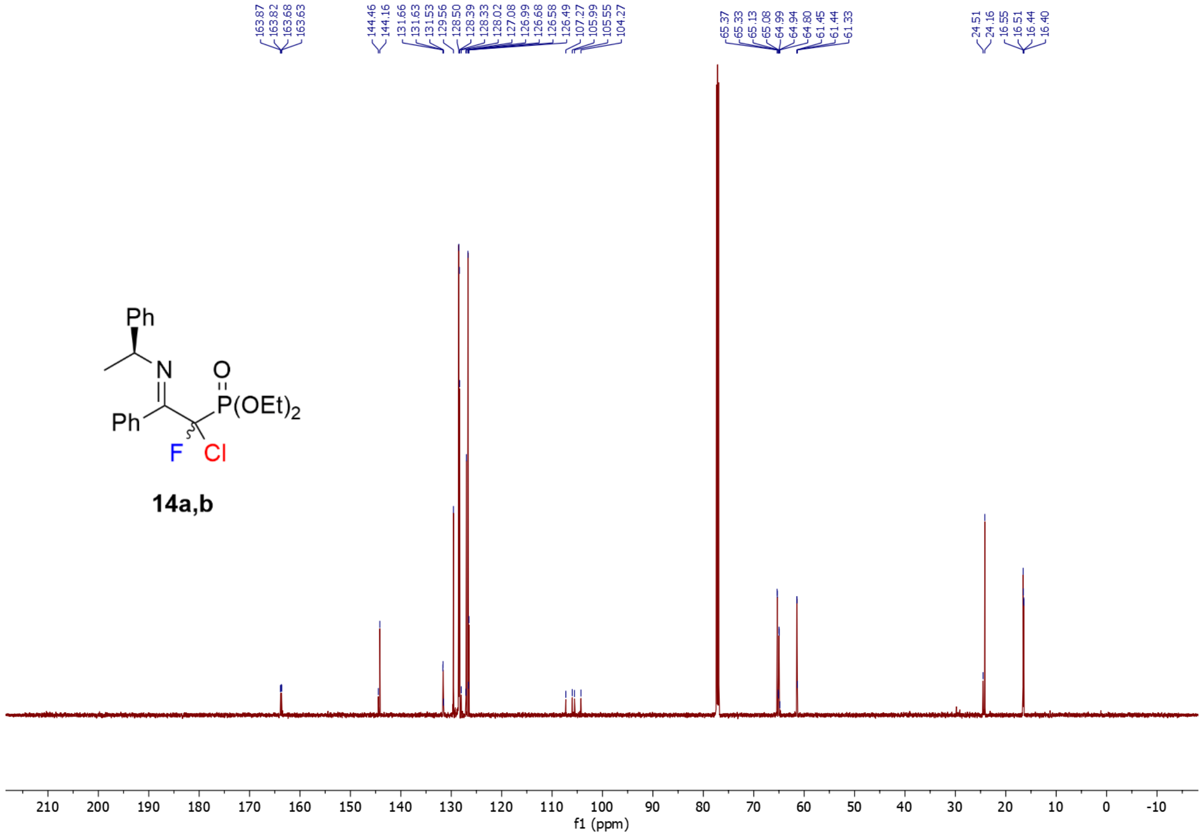

^{13}C NMR of **14a,b** *dr* = 0.2:1

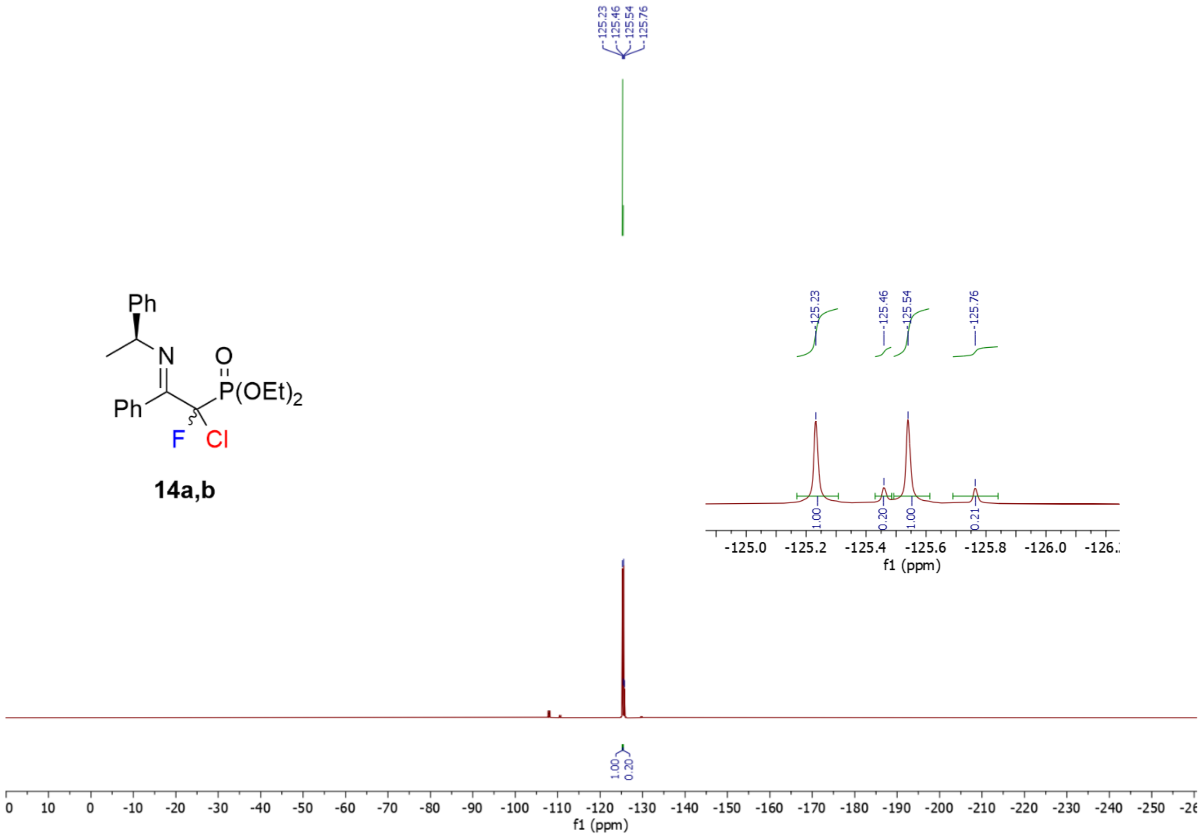

^{19}F NMR of **14a,b** *dr* = 0.2:1

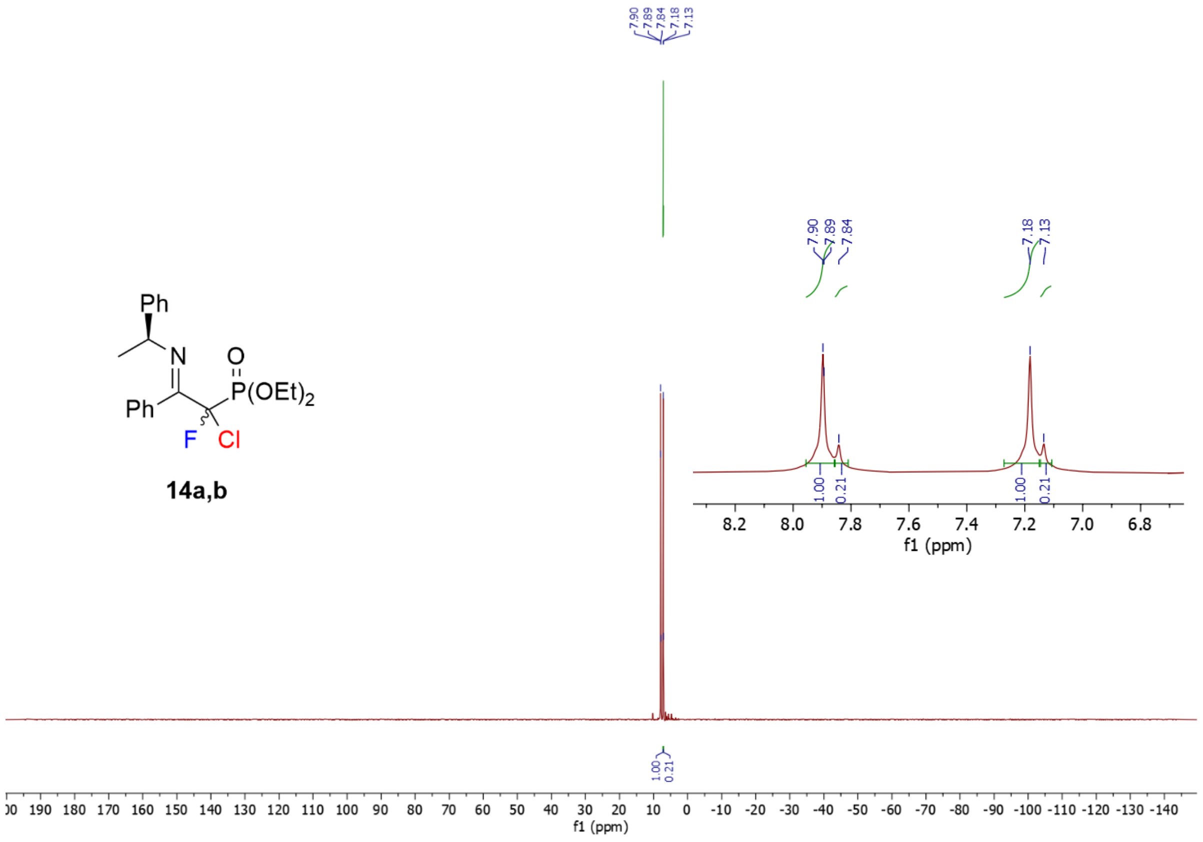

^{31}P{/^{1}H} NMR of **14a,b** *dr* = 0.2: 1

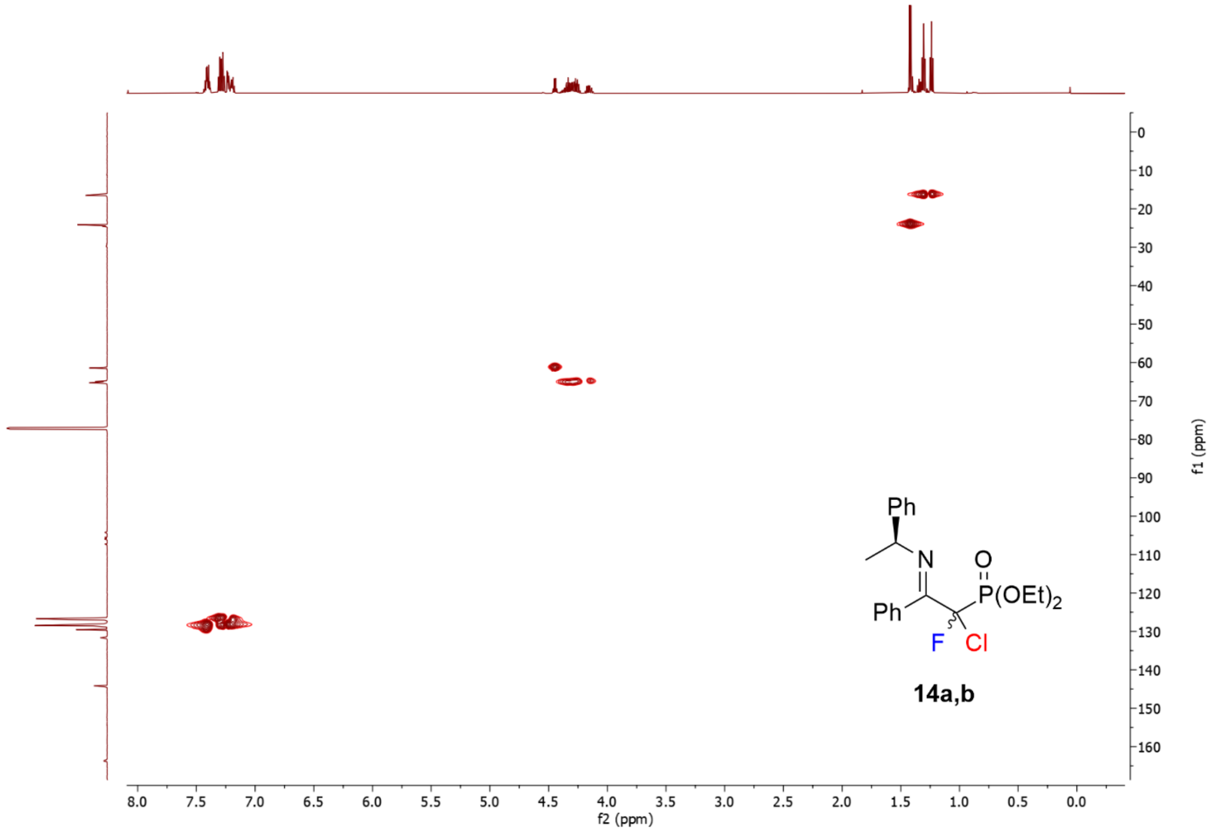

^{1}H-^{13}C HSQC NMR of **14a,b** *dr* = 0.2: 1

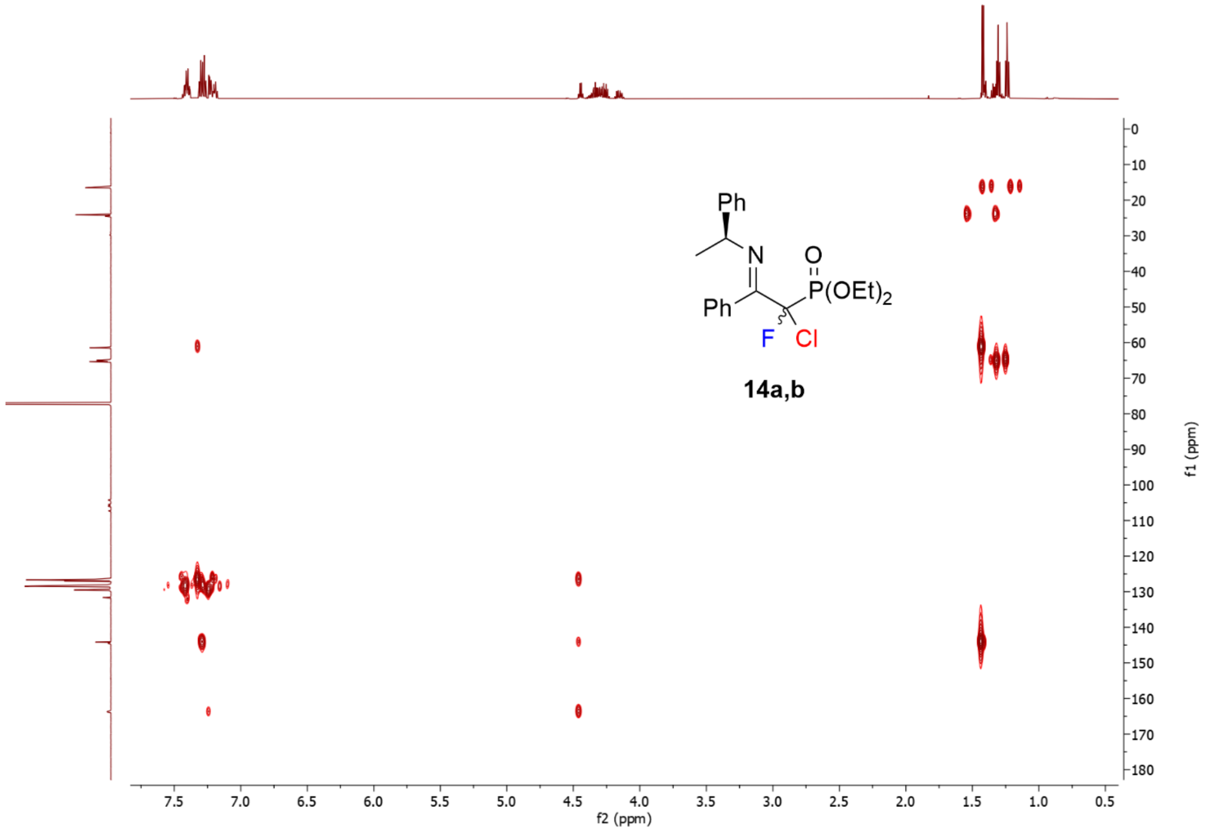

^{1}H-^{13}C HMBC NMR of **14a,b** *dr* = 0.2: 1

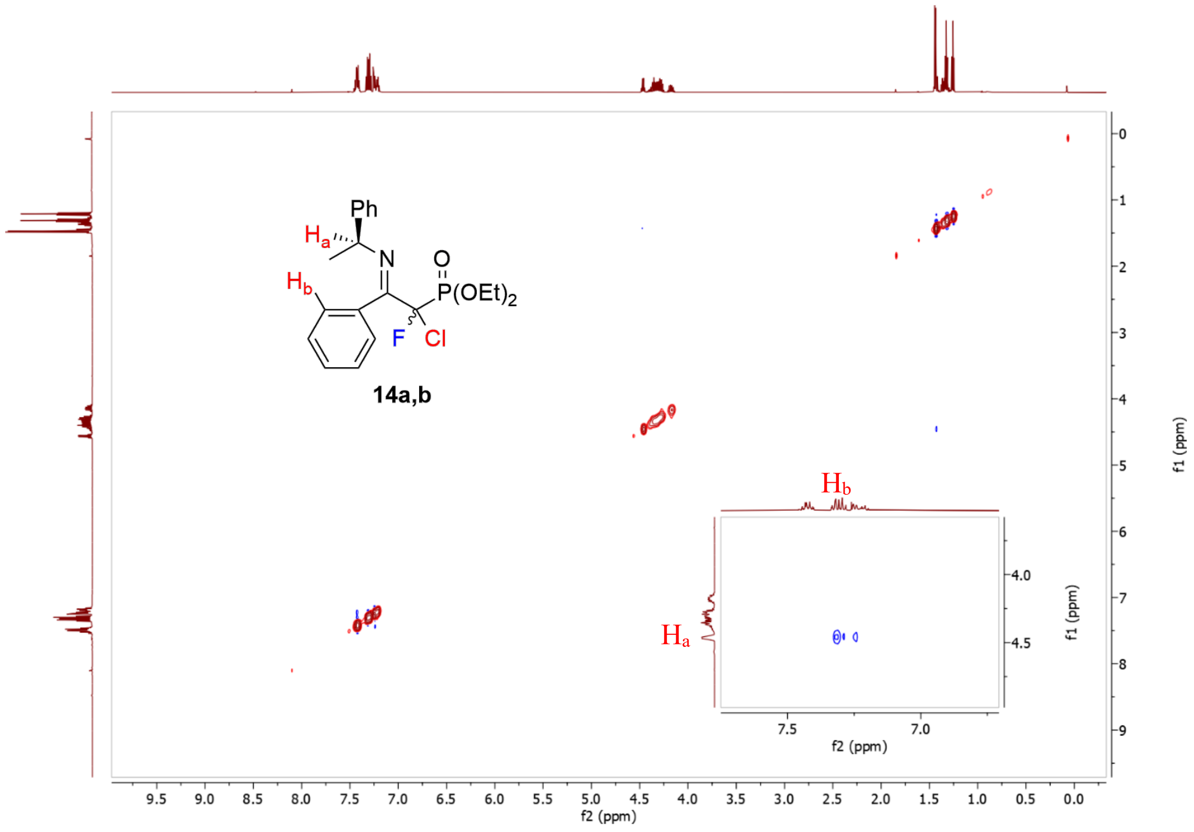

^{1}H-^{1}H NOESY NMR of **14a,b** *dr* = 0.2: 1

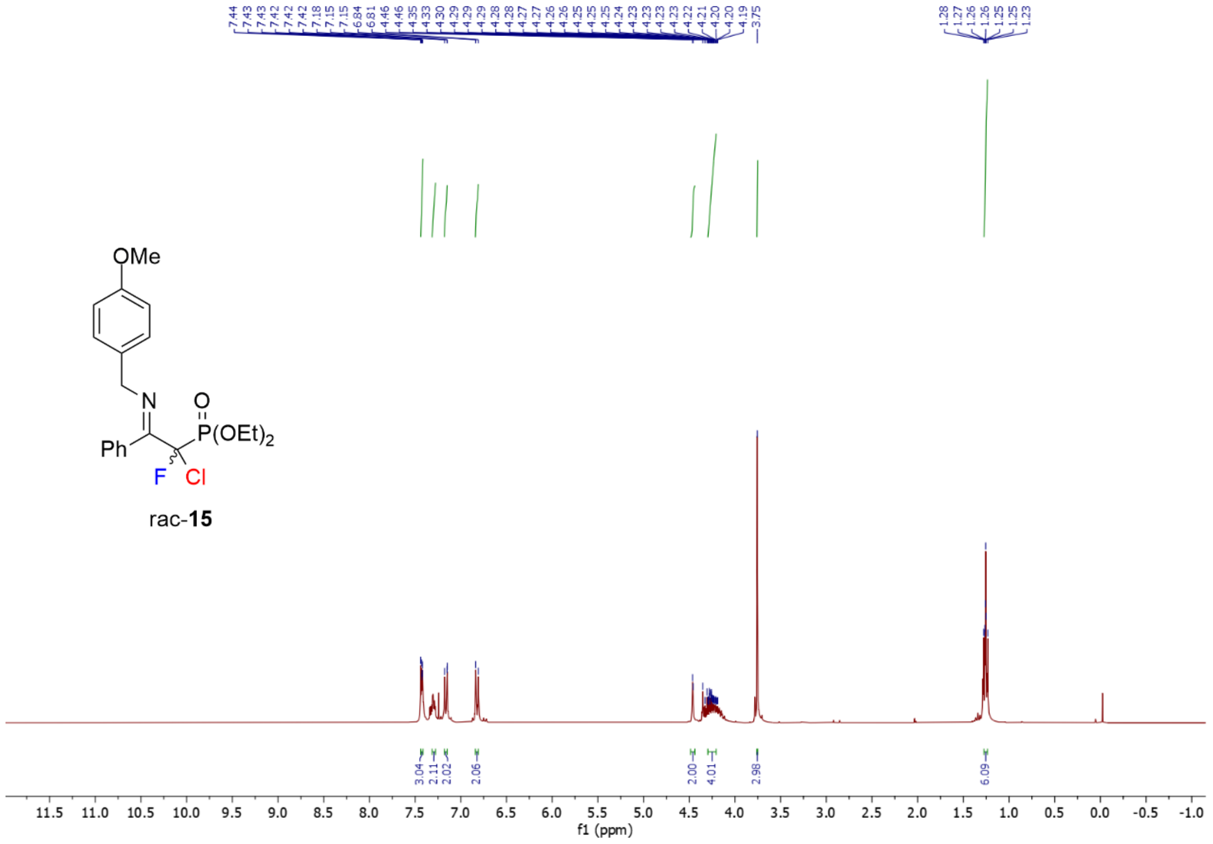

^{1}H NMR of rac-**15**

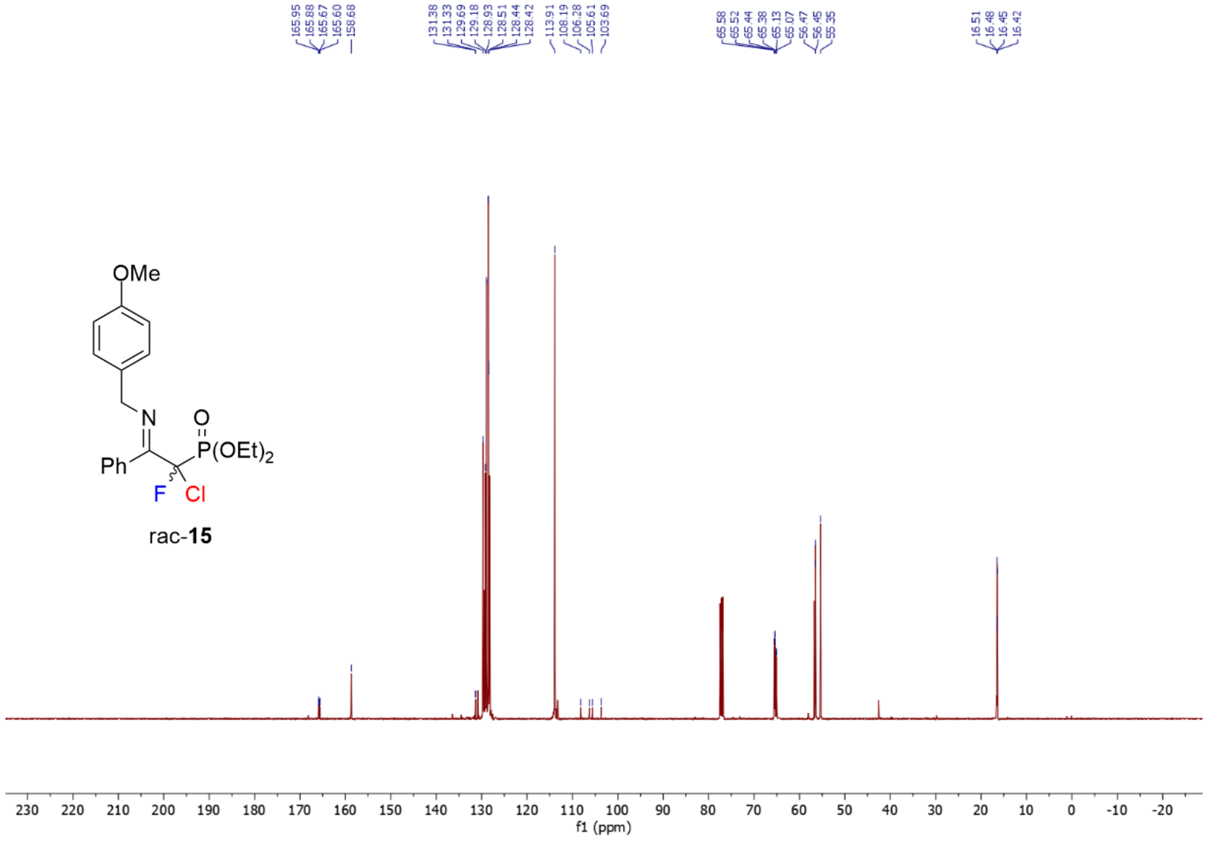

^{13}C NMR of rac-**15**

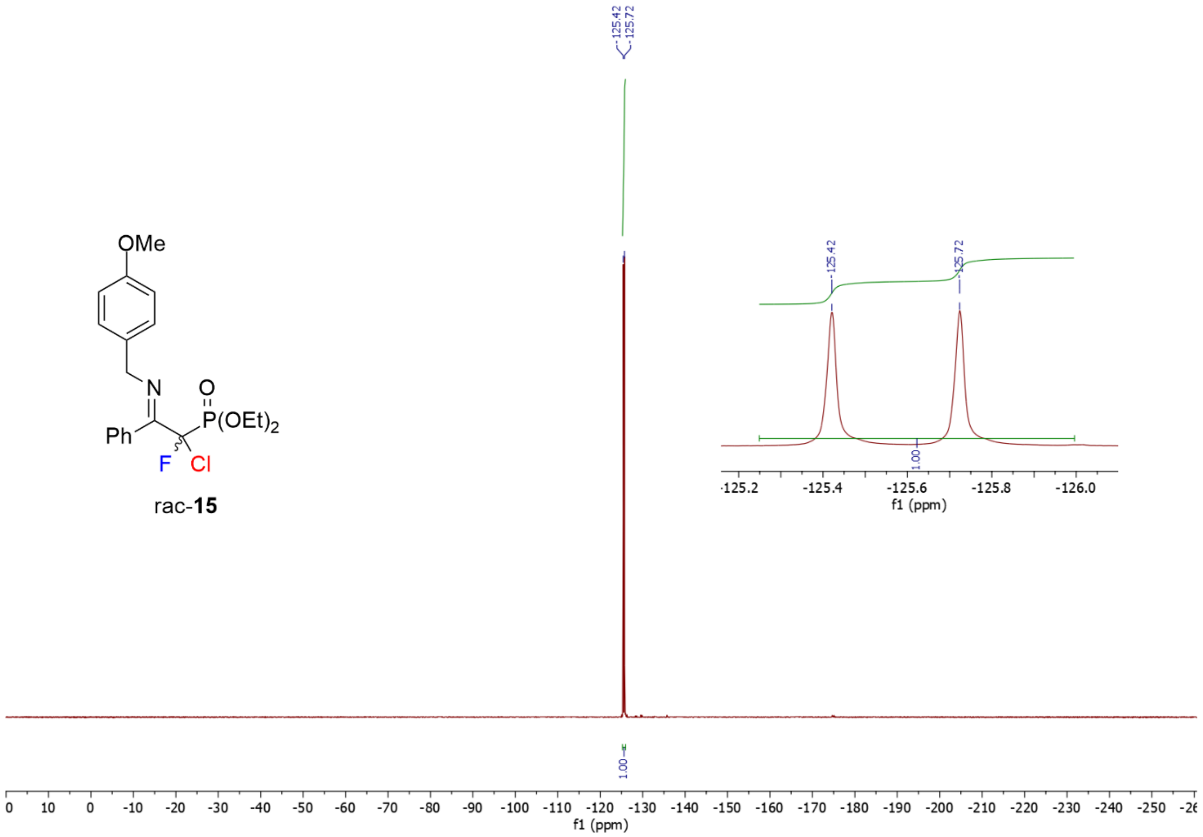

^{19}F NMR of rac-**15**

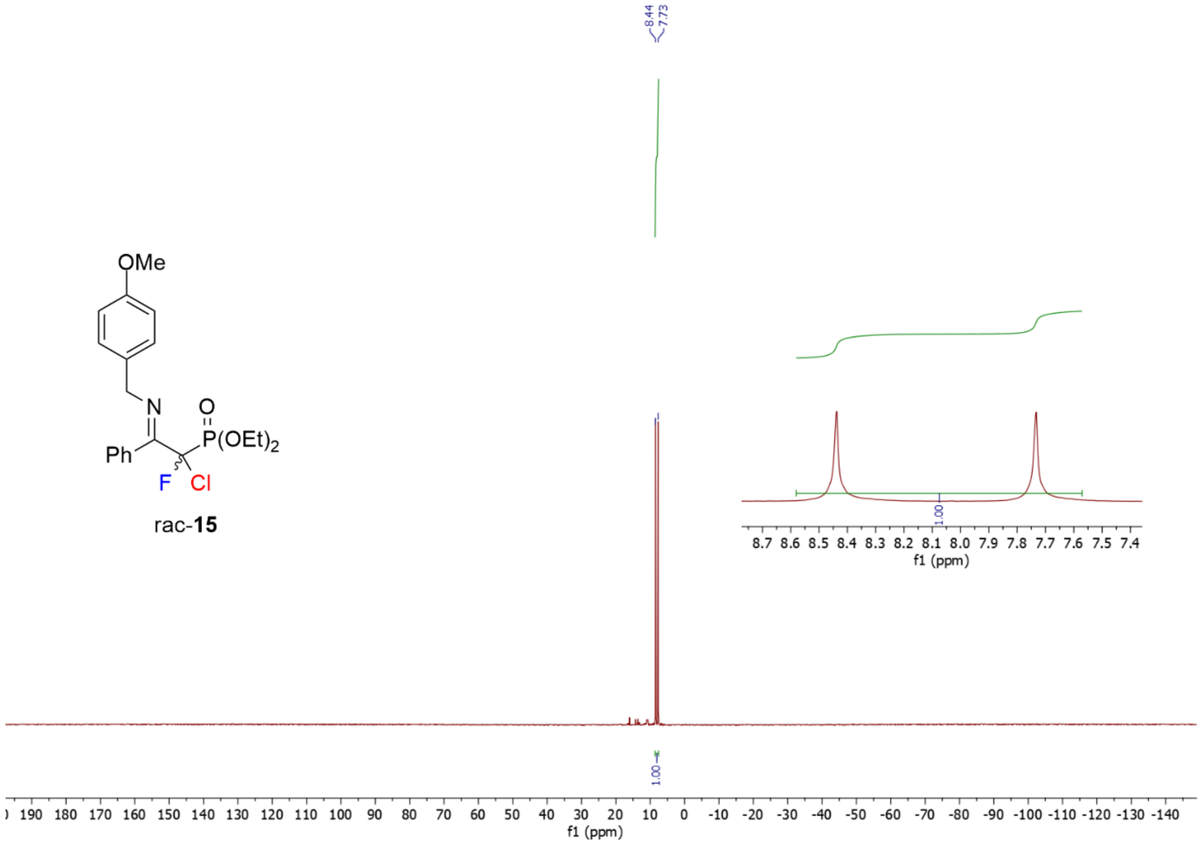

$^{31}P\{/^{1}H\}$ NMR of rac-**15**

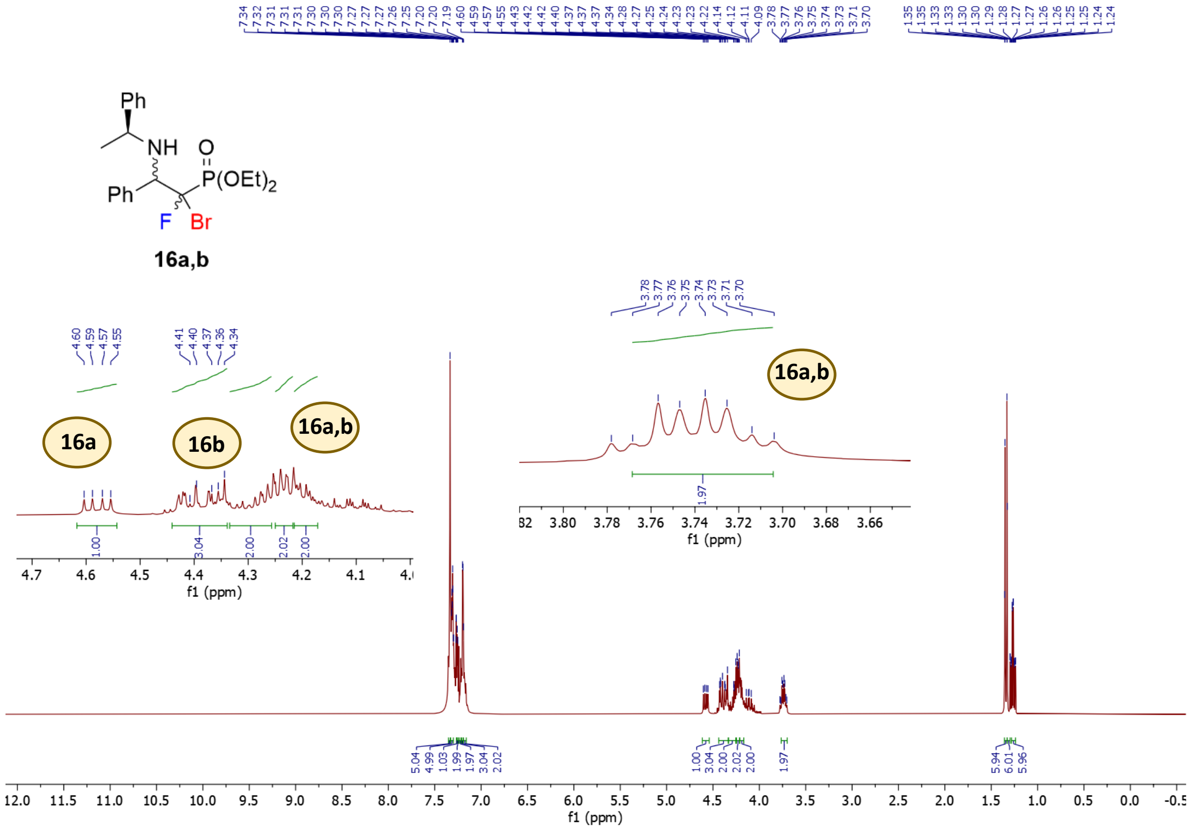

^{1}H NMR of **16a,b** *dr* = 1:0.96

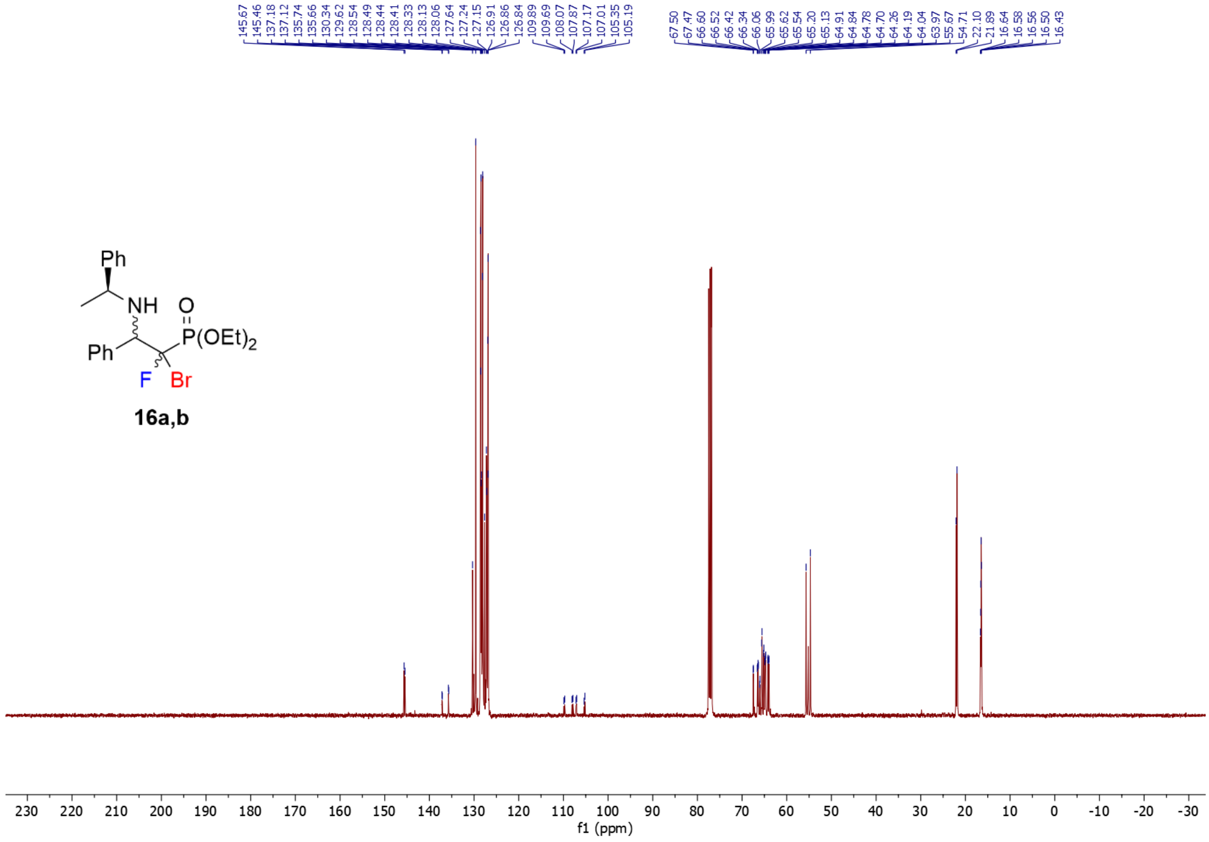

^{13}C NMR of **16a,b** *dr* = 1:0.96

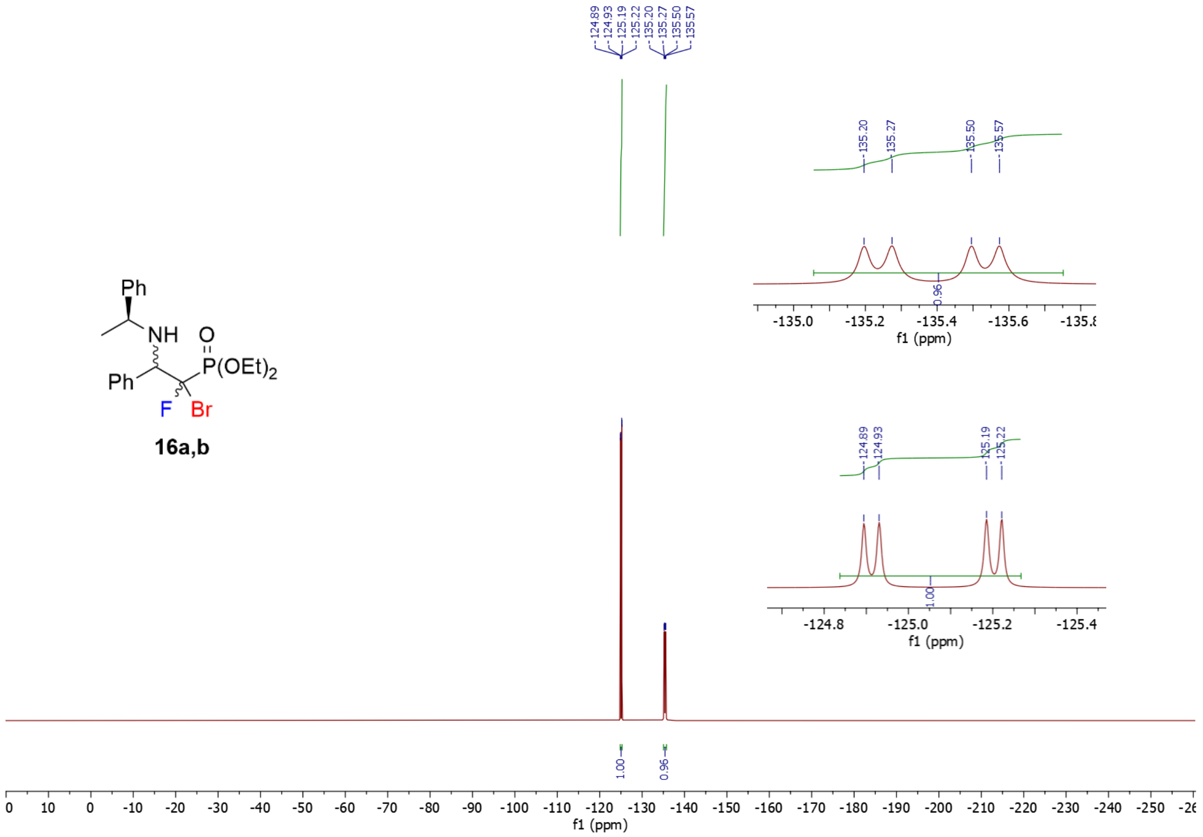

^{19}F NMR of **16a,b** *dr* = 1:0.96

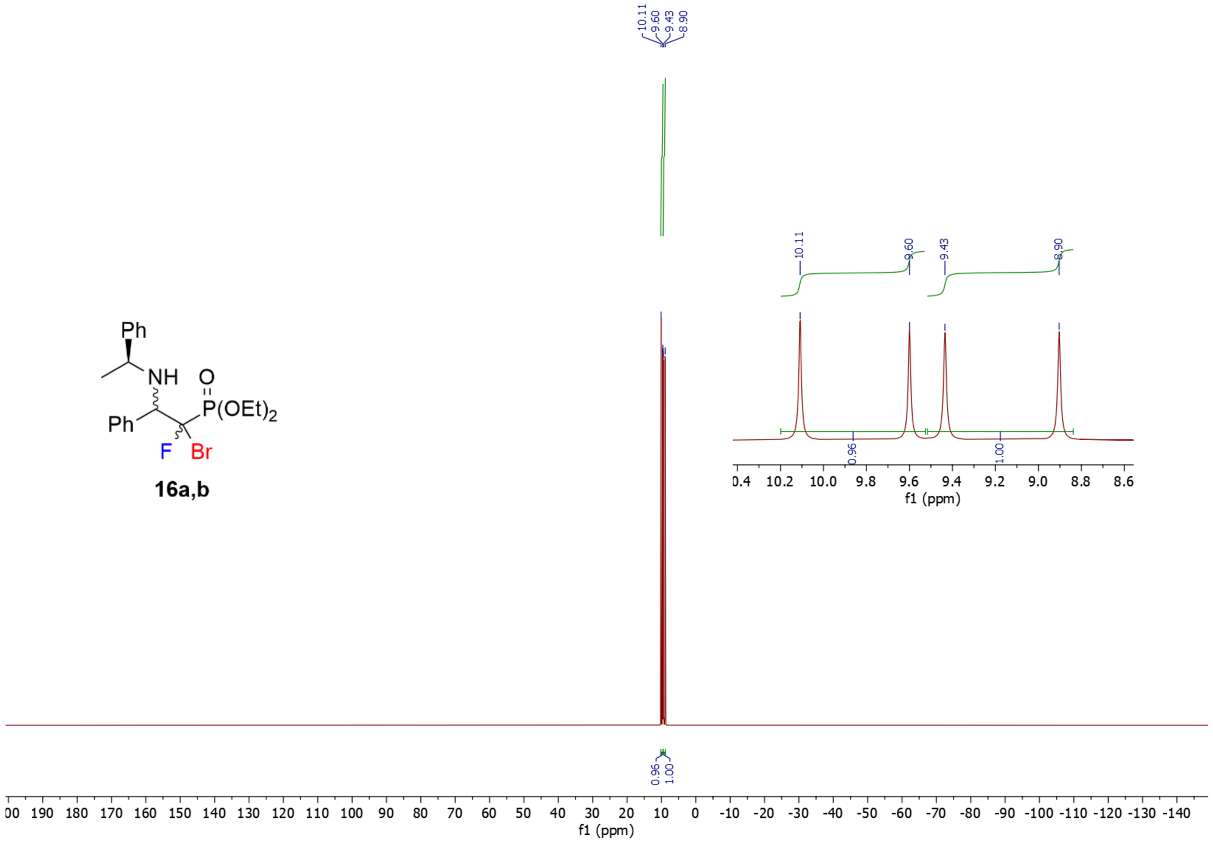

^{31}P{/^{1}H} NMR of **16a,b** *dr* = 1:0.96

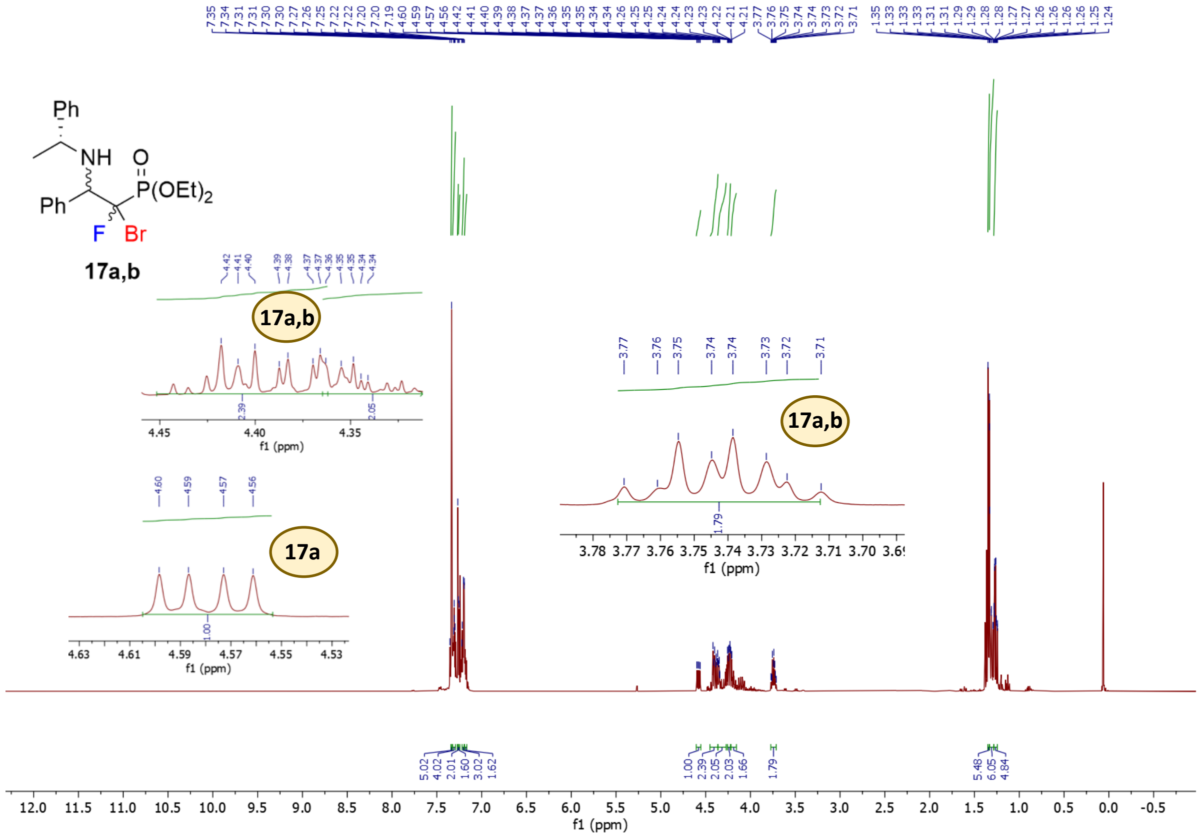

^{1}H NMR of **17a,b** *dr* = 1:0.80

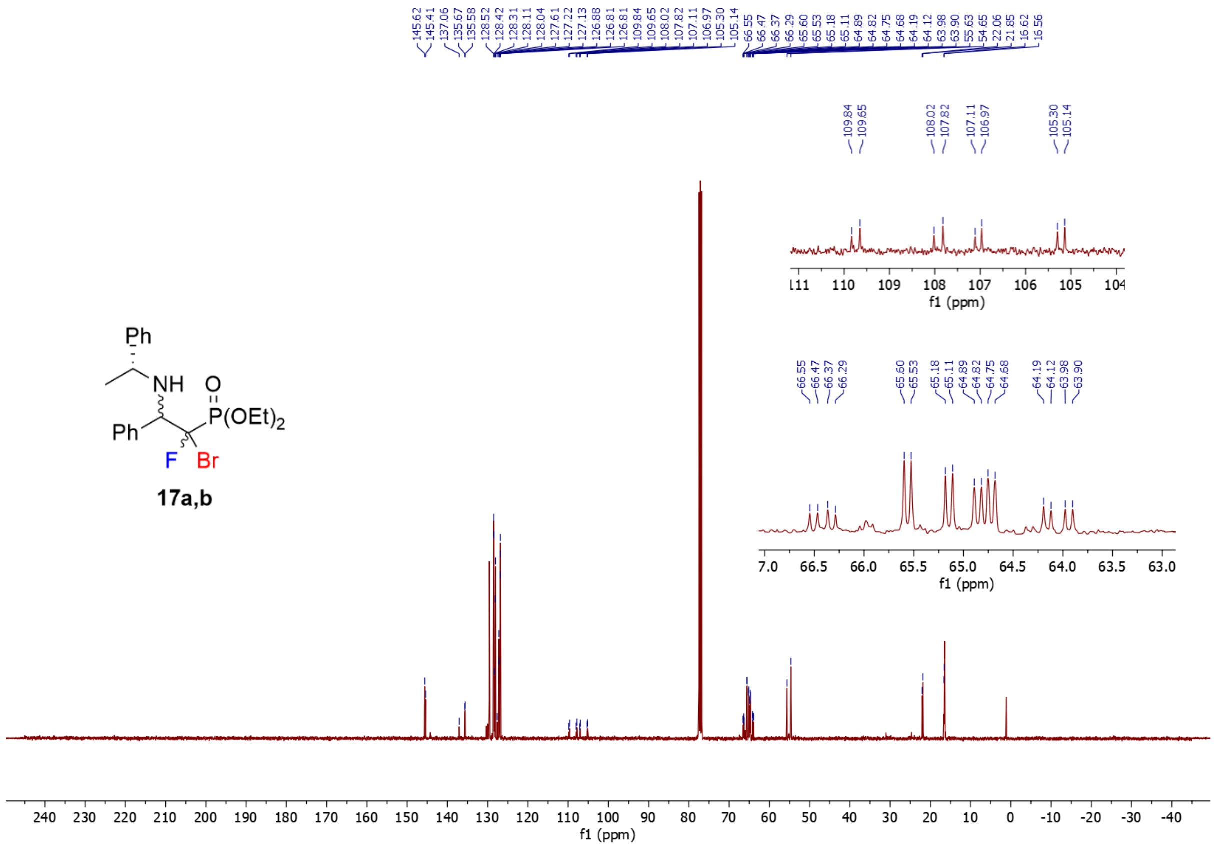

^{13}C NMR of **17a,b** *dr* = 1:0.80

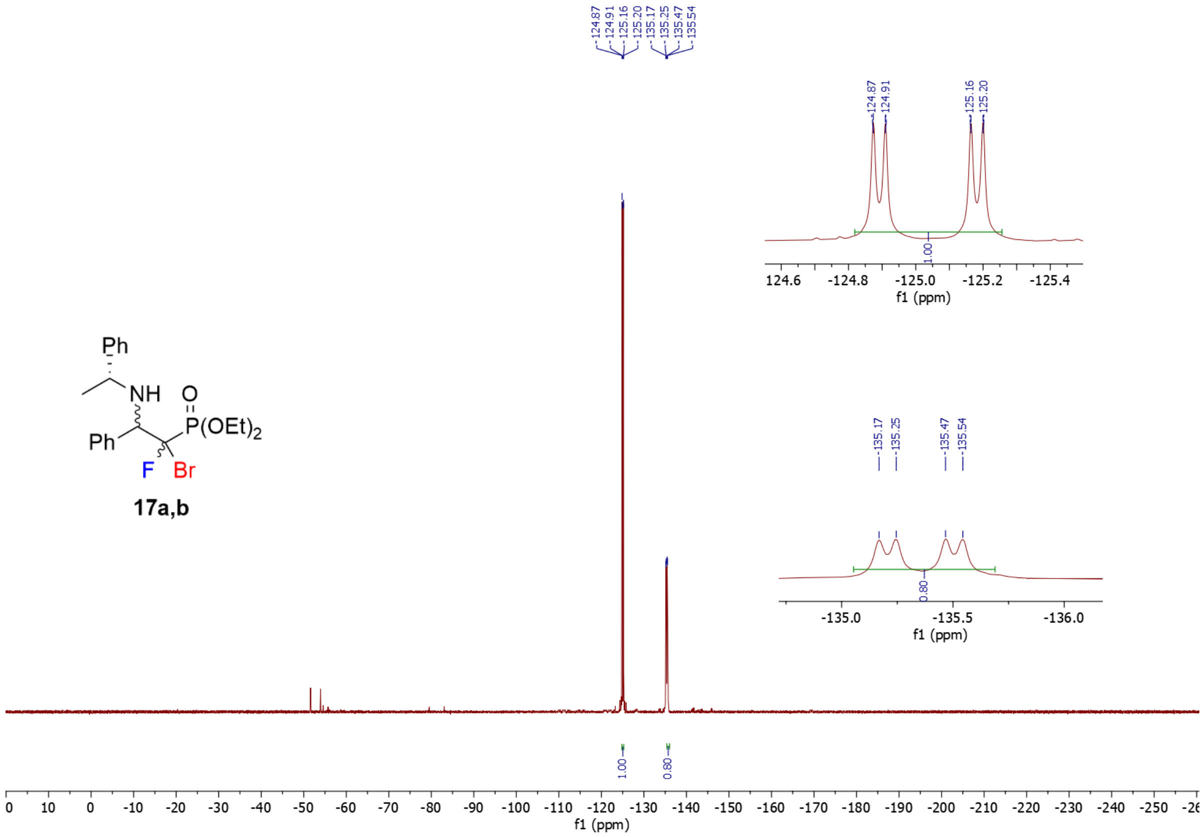

^{19}F NMR of **17a,b** *dr* = 1:0.80

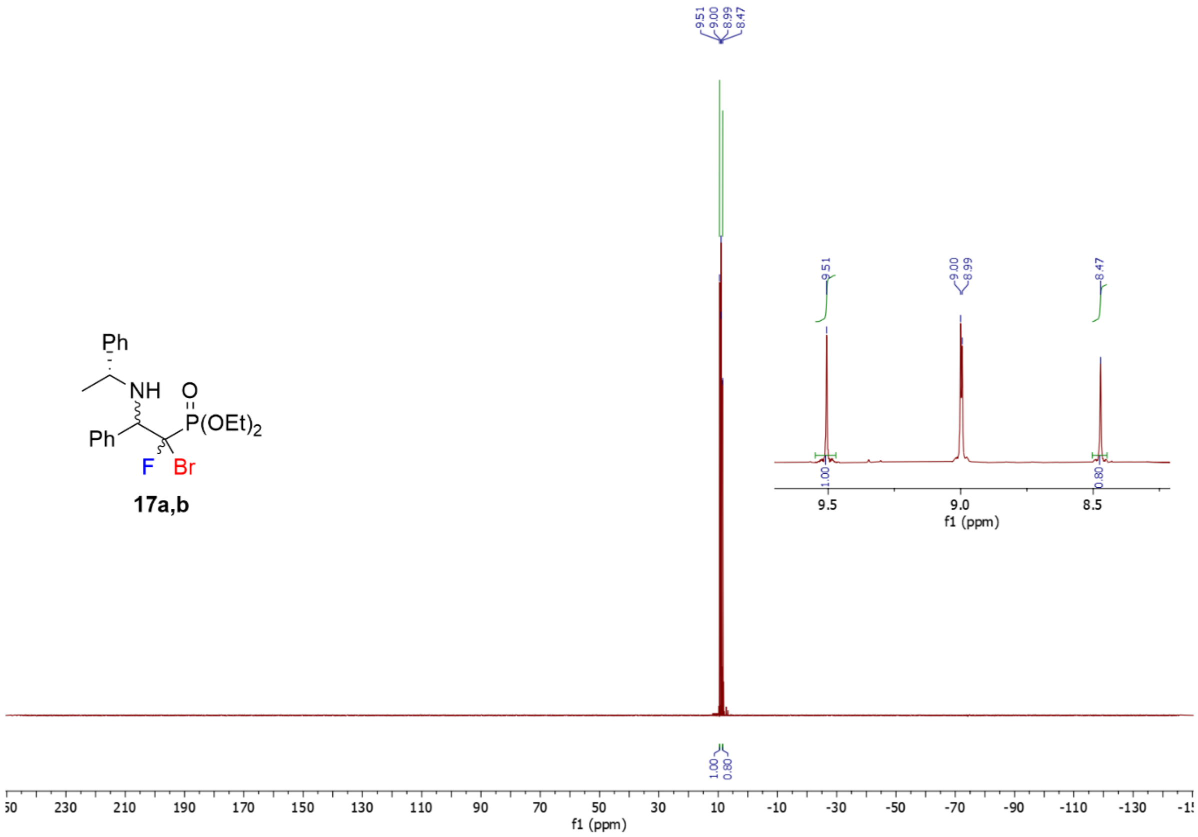

^{31}P{/^{1}H} NMR of **17a,b** *dr* = 1:0.80

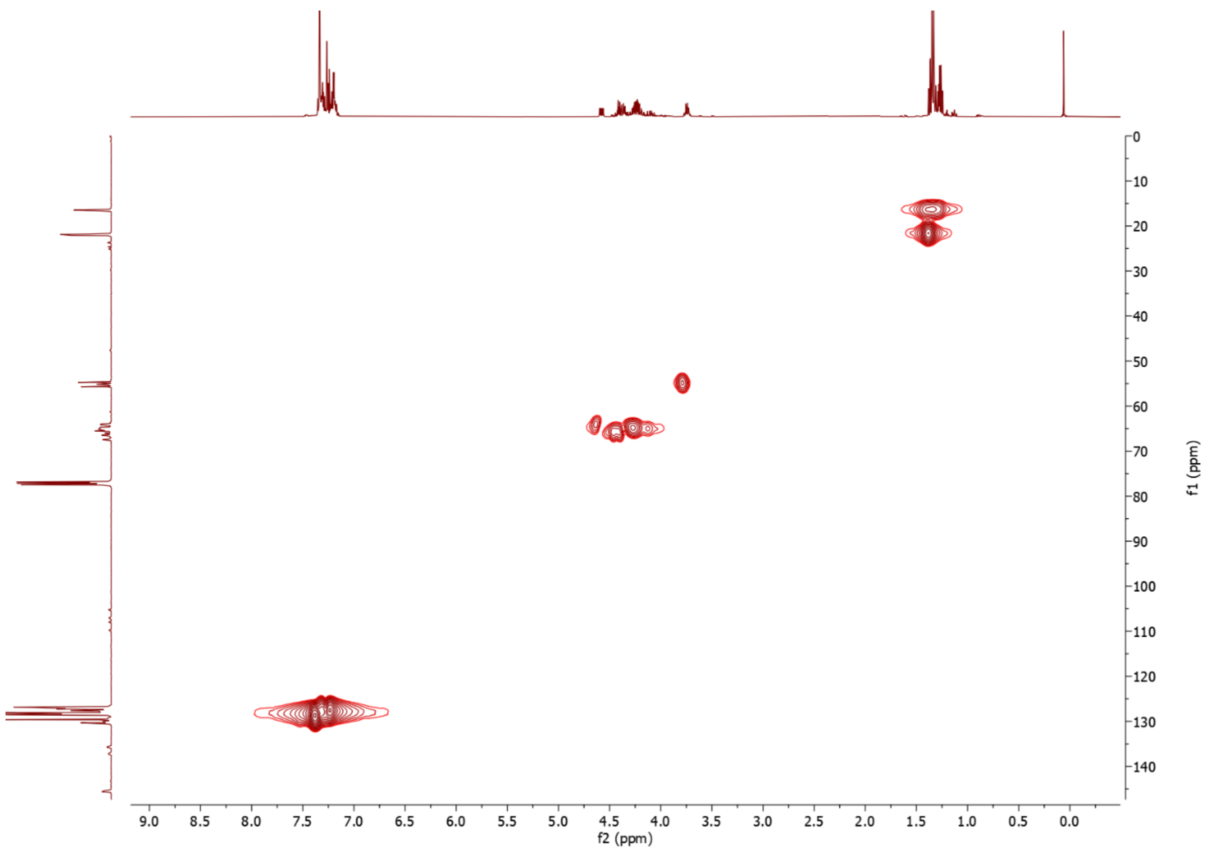

^{1}H-^{13}C HSQC NMR of **17a,b** *dr* = 1:0.80

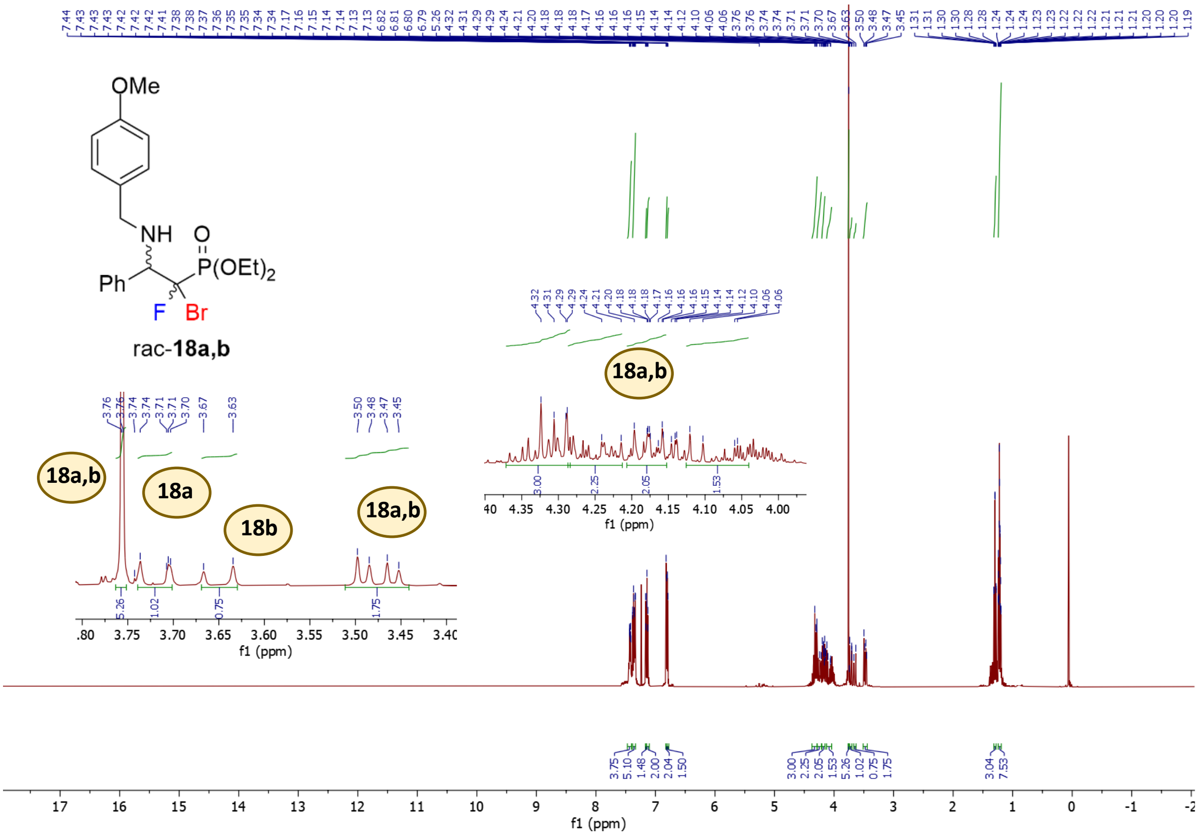

^{1}H NMR of rac-**18a,b** *dr* = 1:0.75

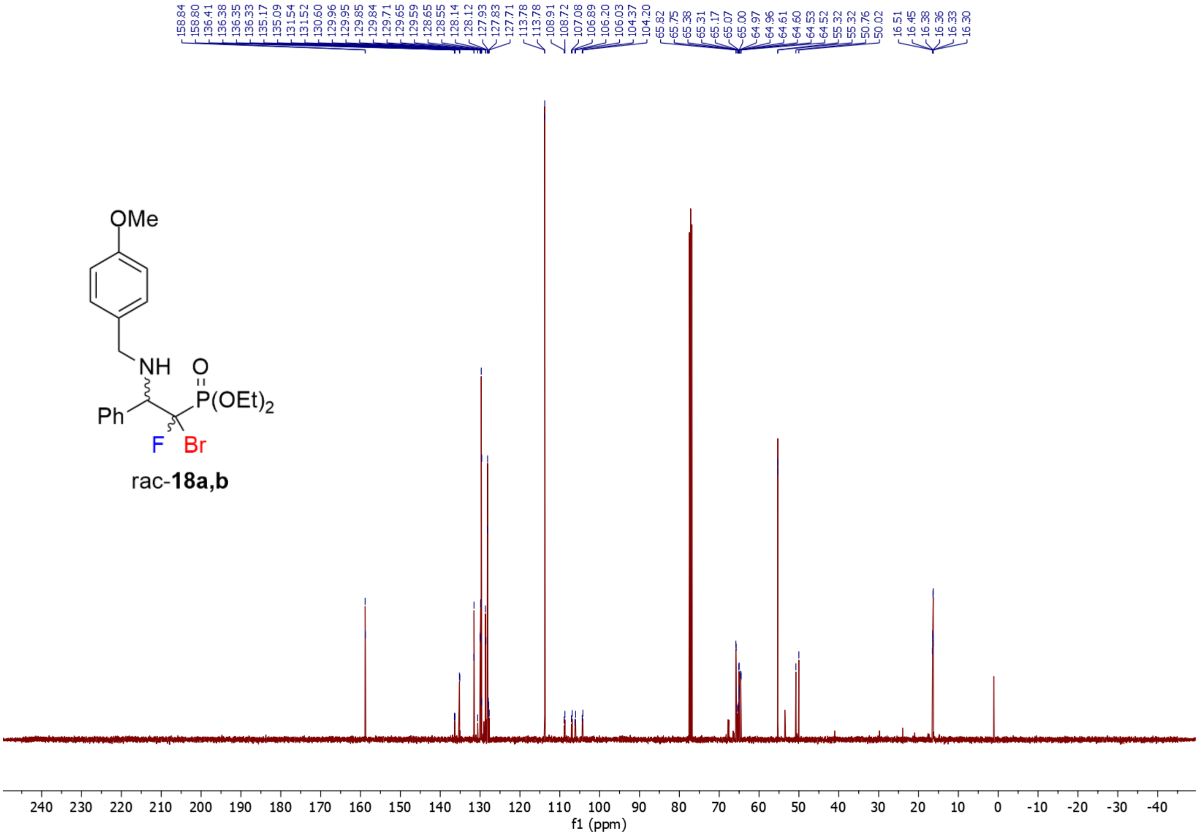

^{13}C NMR of rac-**18a,b** *dr* = 1:0.75

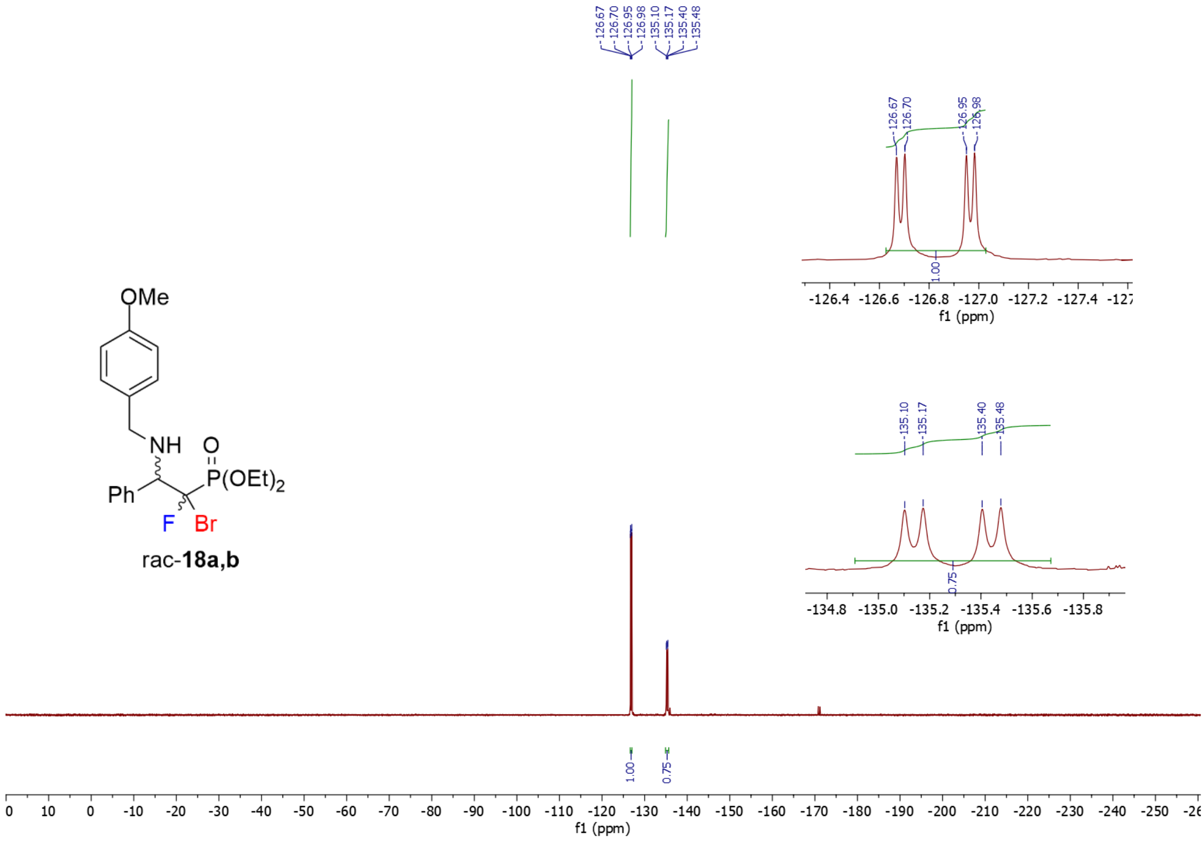

^{19}F NMR of rac-**18a,b** *dr* = 1:0.75

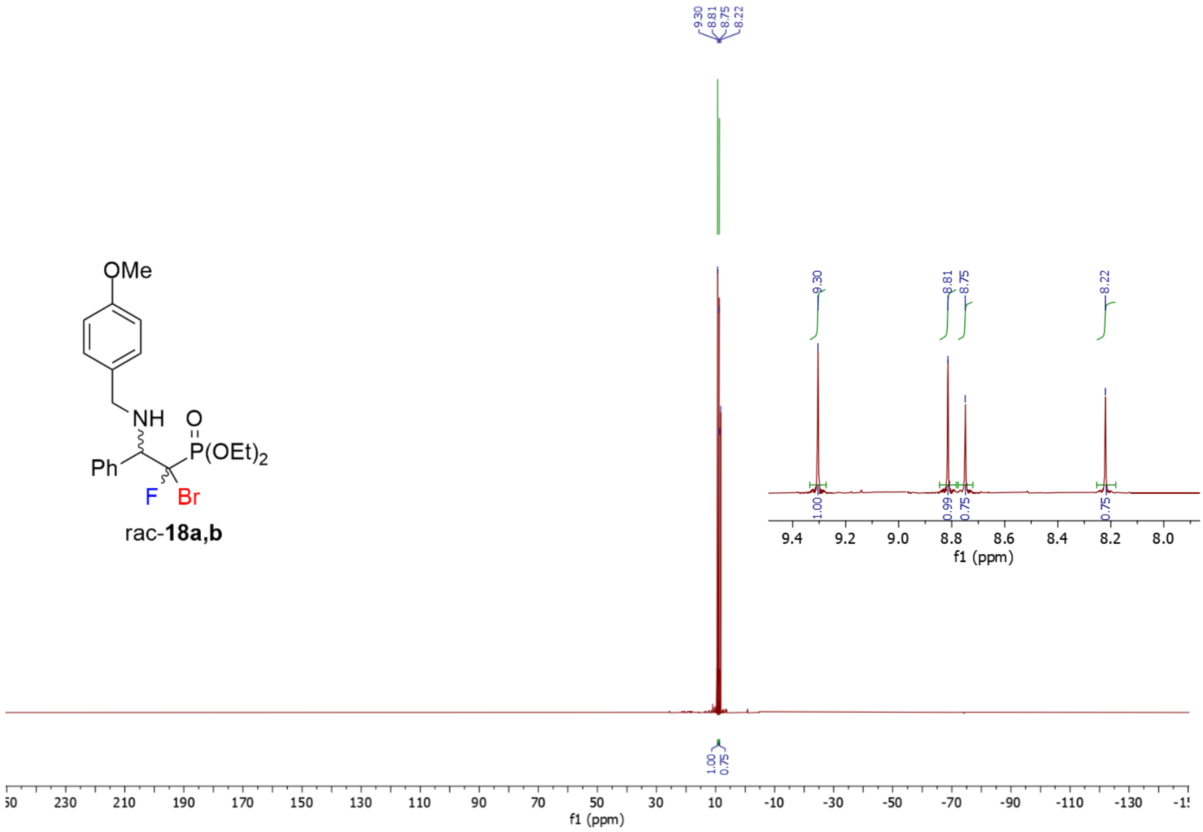

^{31}P{/^{1}H} NMR of rac-**18a,b** *dr* = 1:0.75

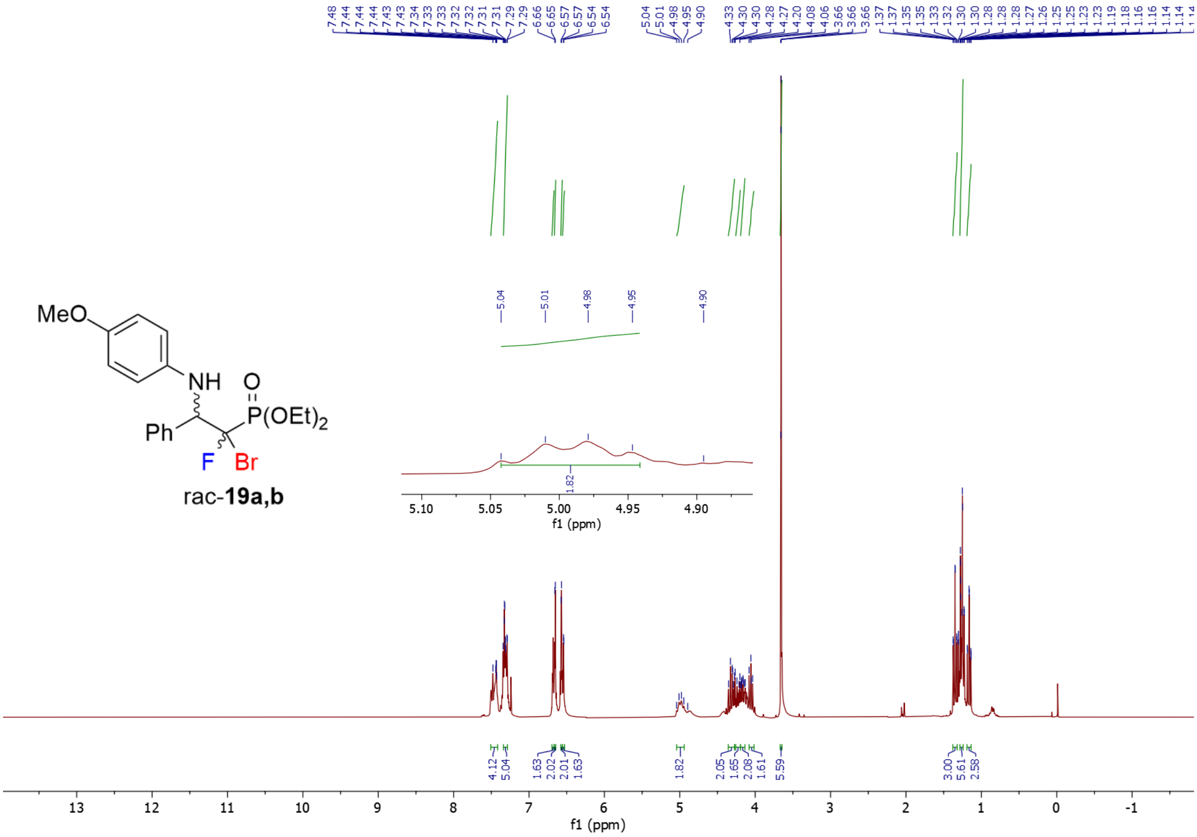

^{1}H NMR of rac-**19a,b** *dr* = 1:0.86

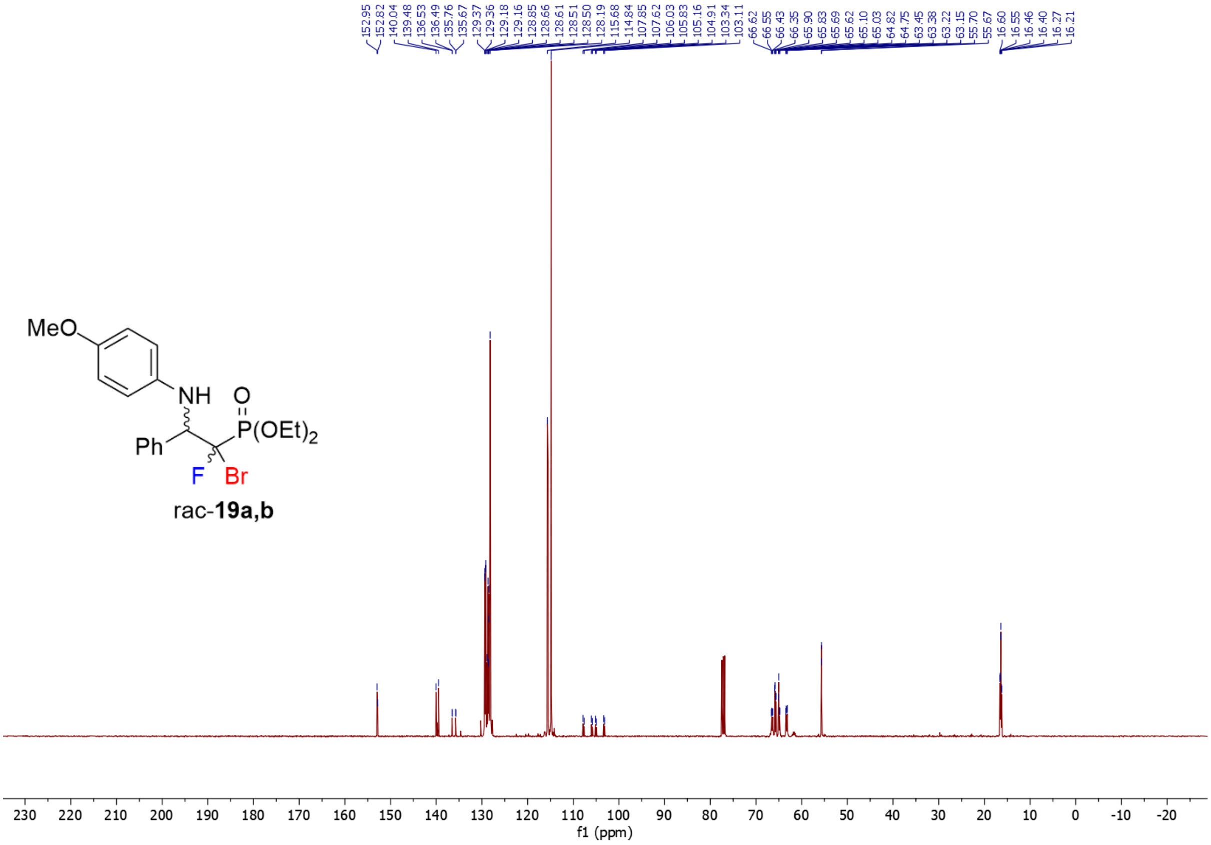

^{13}C NMR of rac-**19a,b** *dr* = 1:0.86

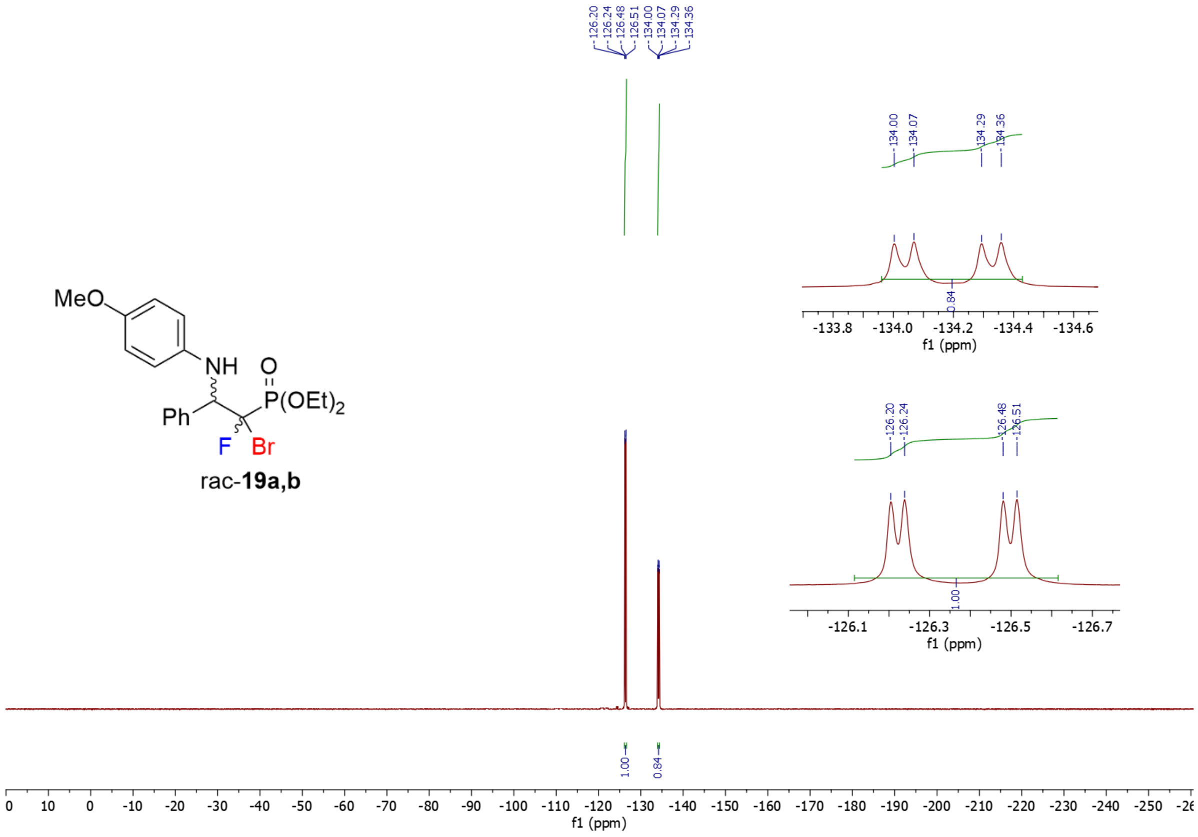

^{19}F NMR of rac-**19a,b** *dr* = 1:0.86

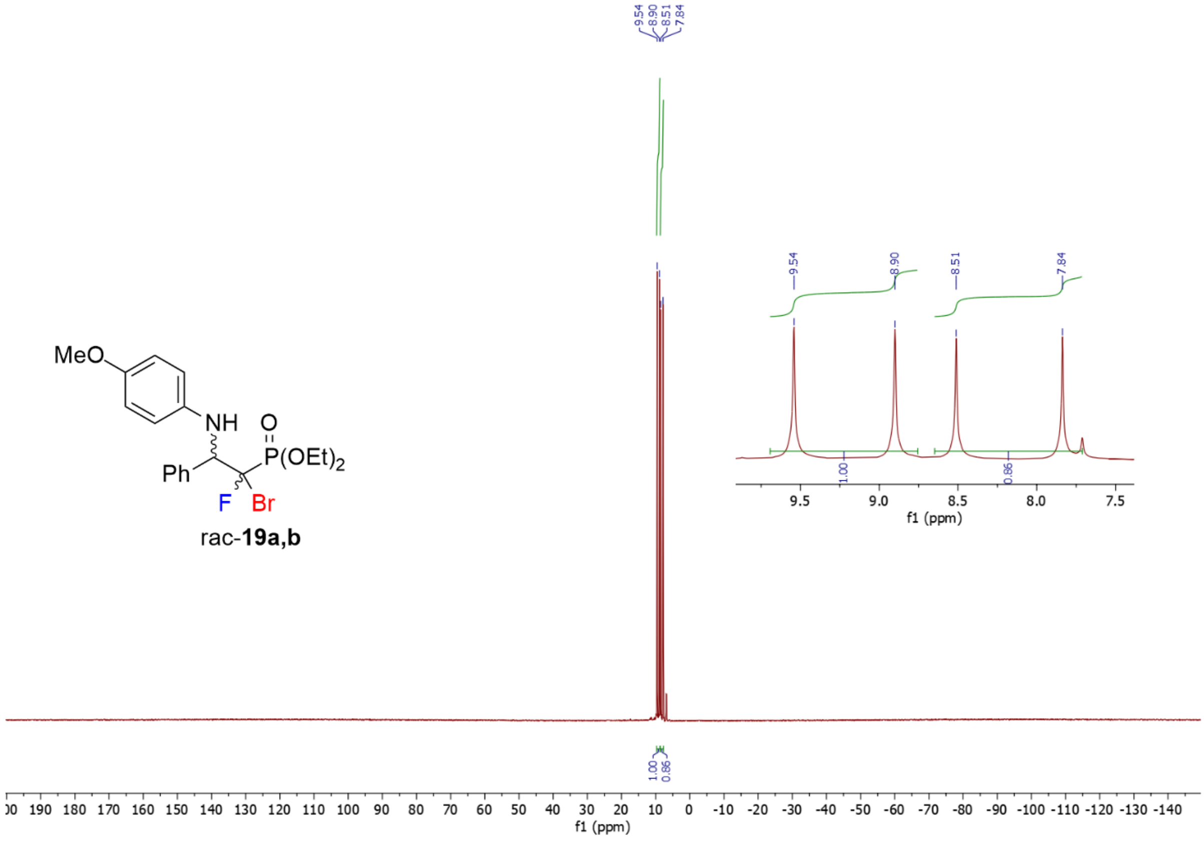

^{31}P{/^{1}H} NMR of rac-**19a,b** *dr* = 1:0.86

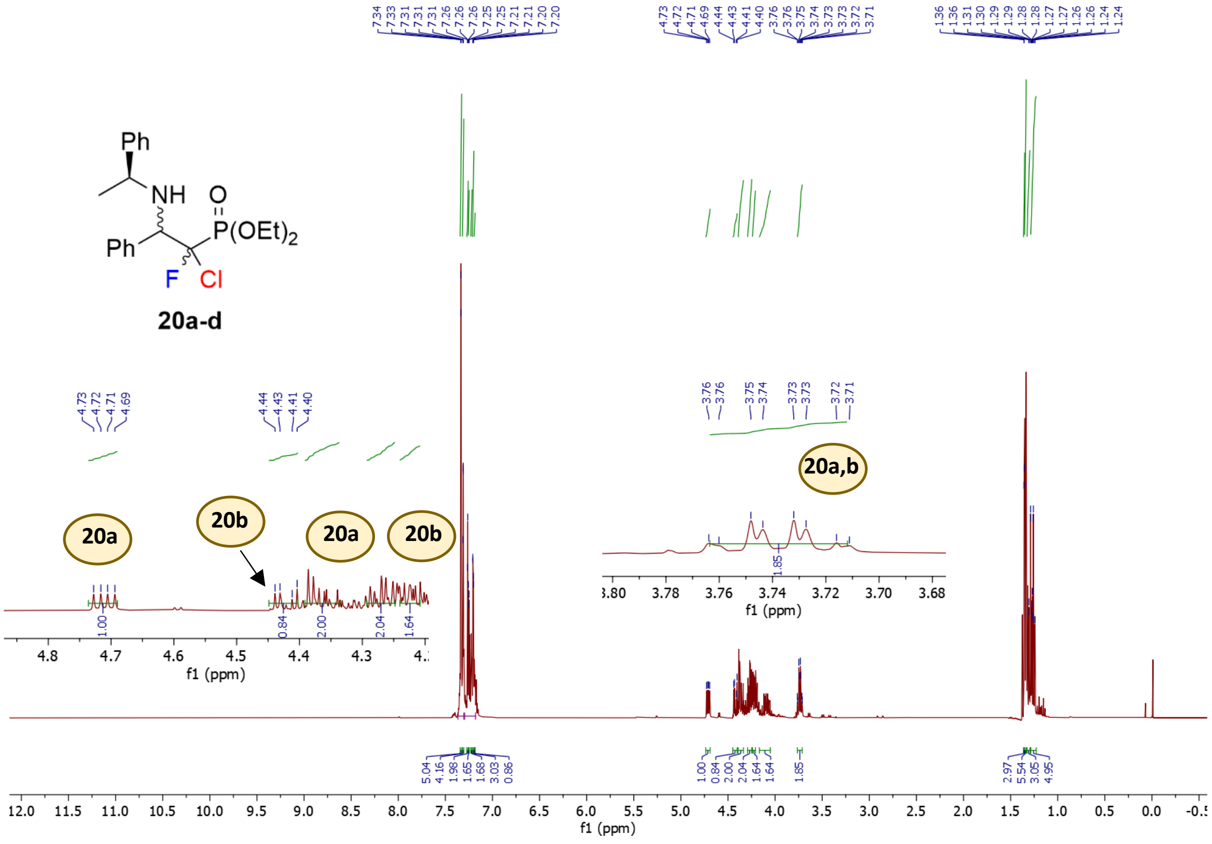

^{1}H NMR of **20a-d** *dr* = 1:0.83:0.07:0.11

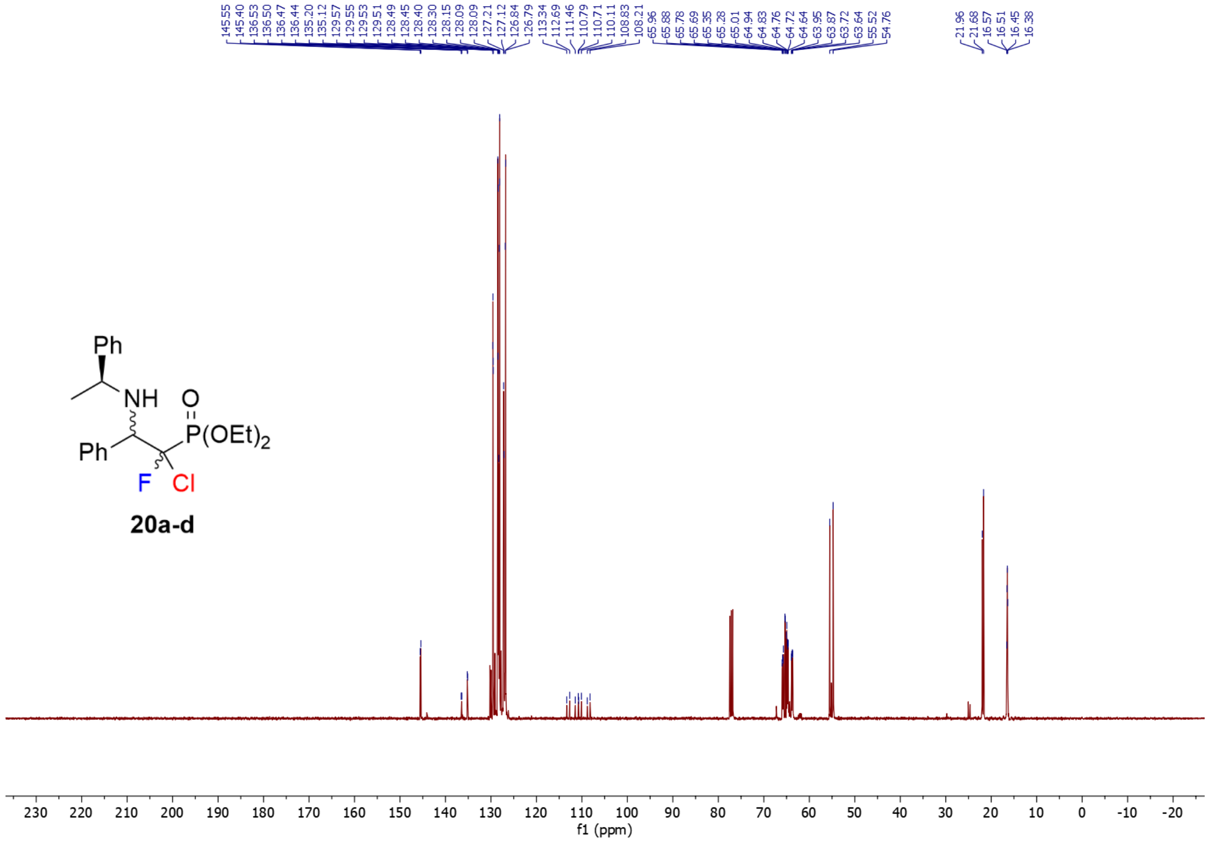

^{13}C NMR of **20a-d** *dr* = 1:0.83:0.07:0.11

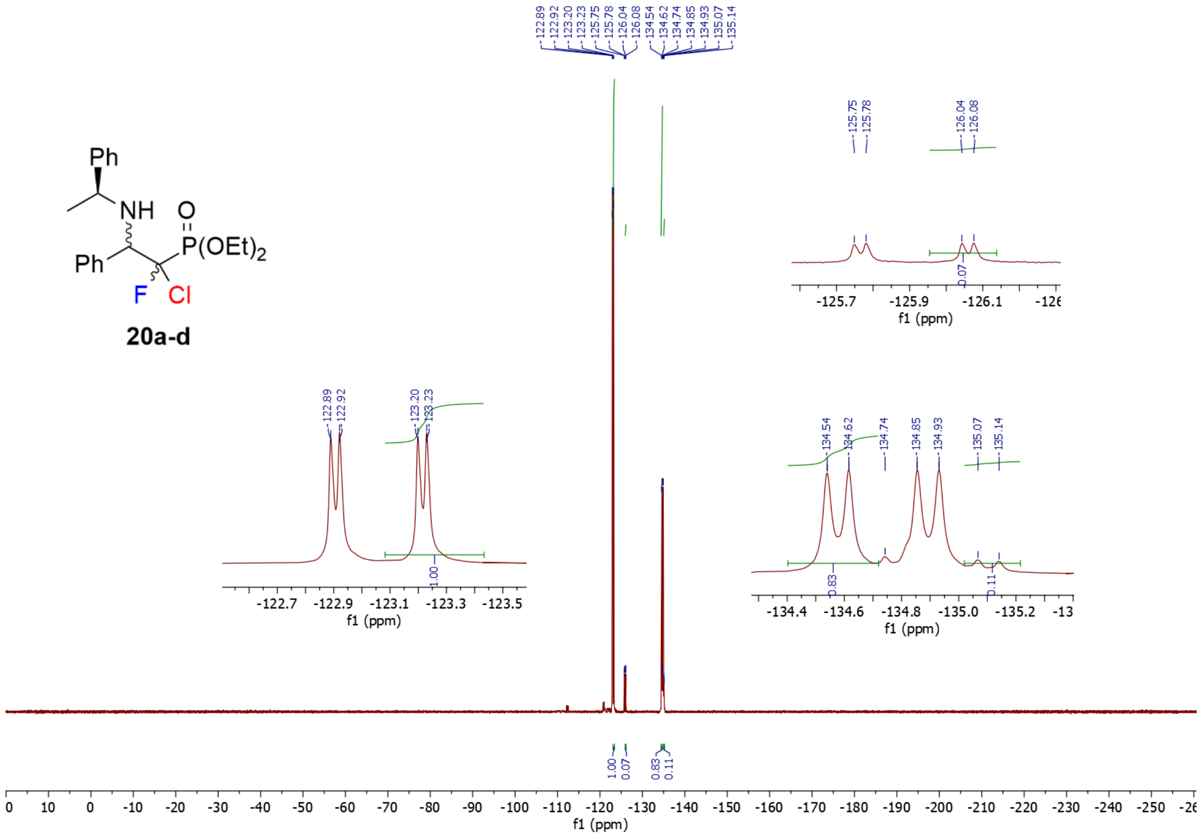

^{19}F NMR of **20a-d** *dr* = 1:0.83:0.07:0.11

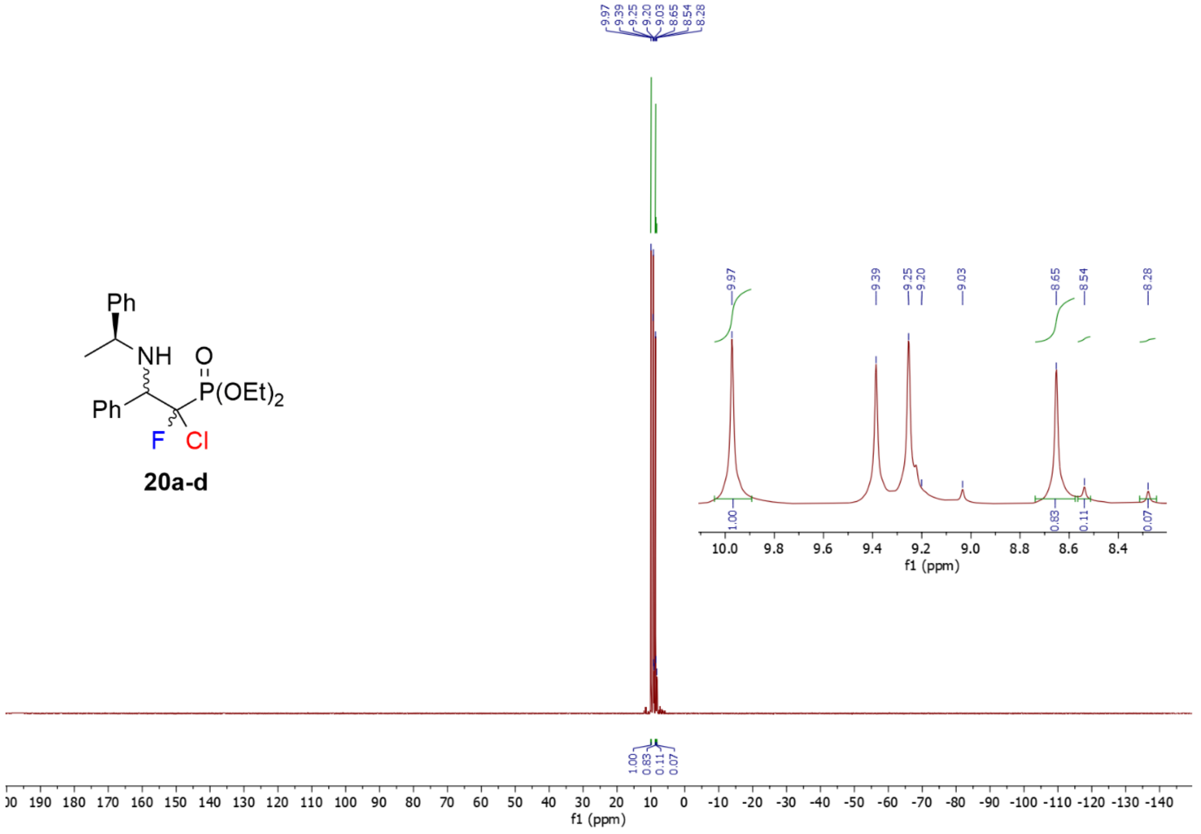

^{31}P{/^{1}H} NMR of **20a-d** *dr* = 1:0.83:0.07:0.11

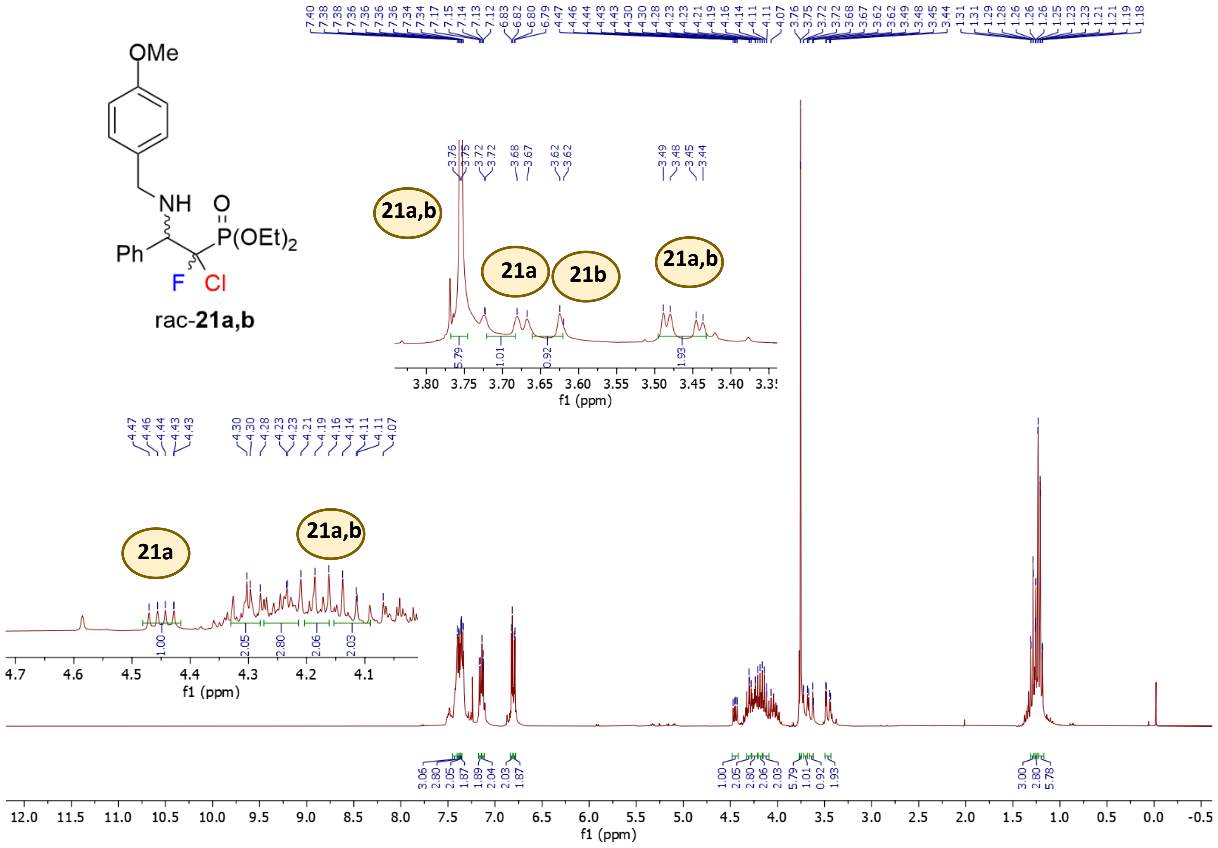

^{1}H NMR of rac-**21a,b** *dr* = 1:0.92

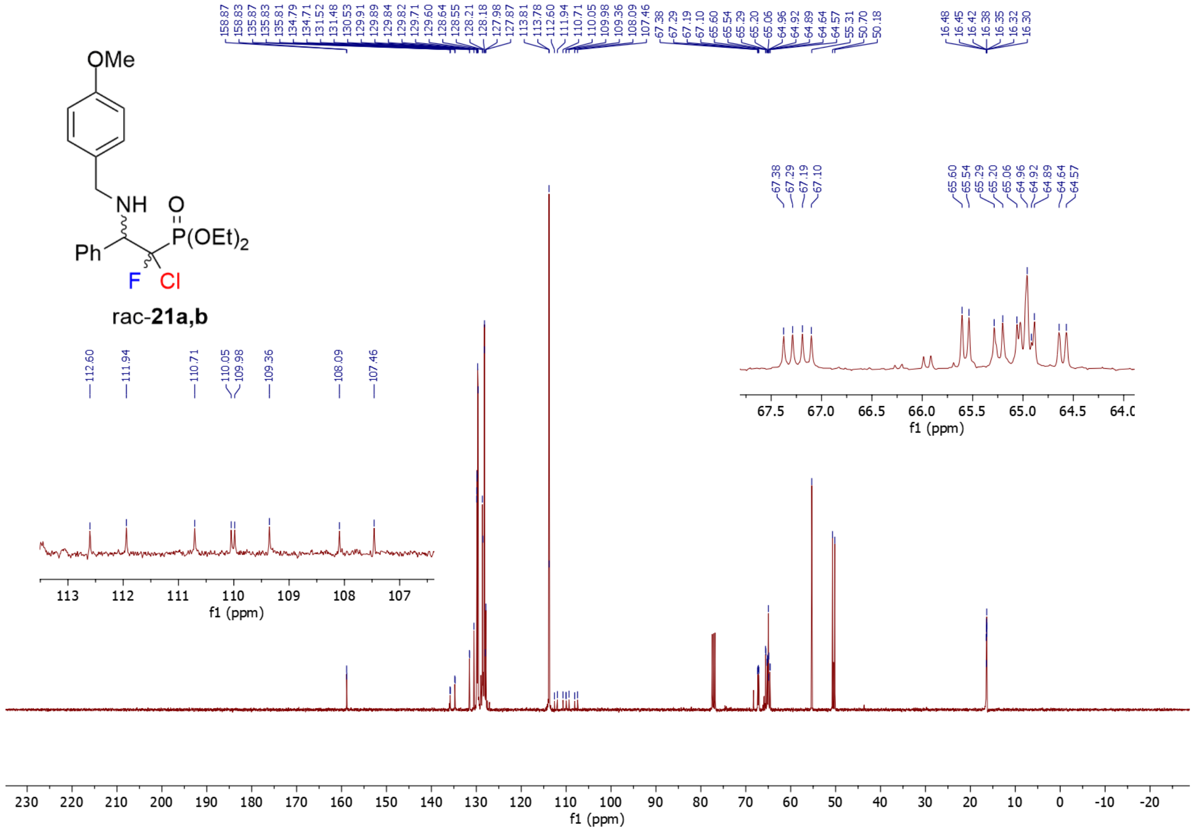

^{13}C NMR of rac-**21a,b** *dr* = 1:0.92

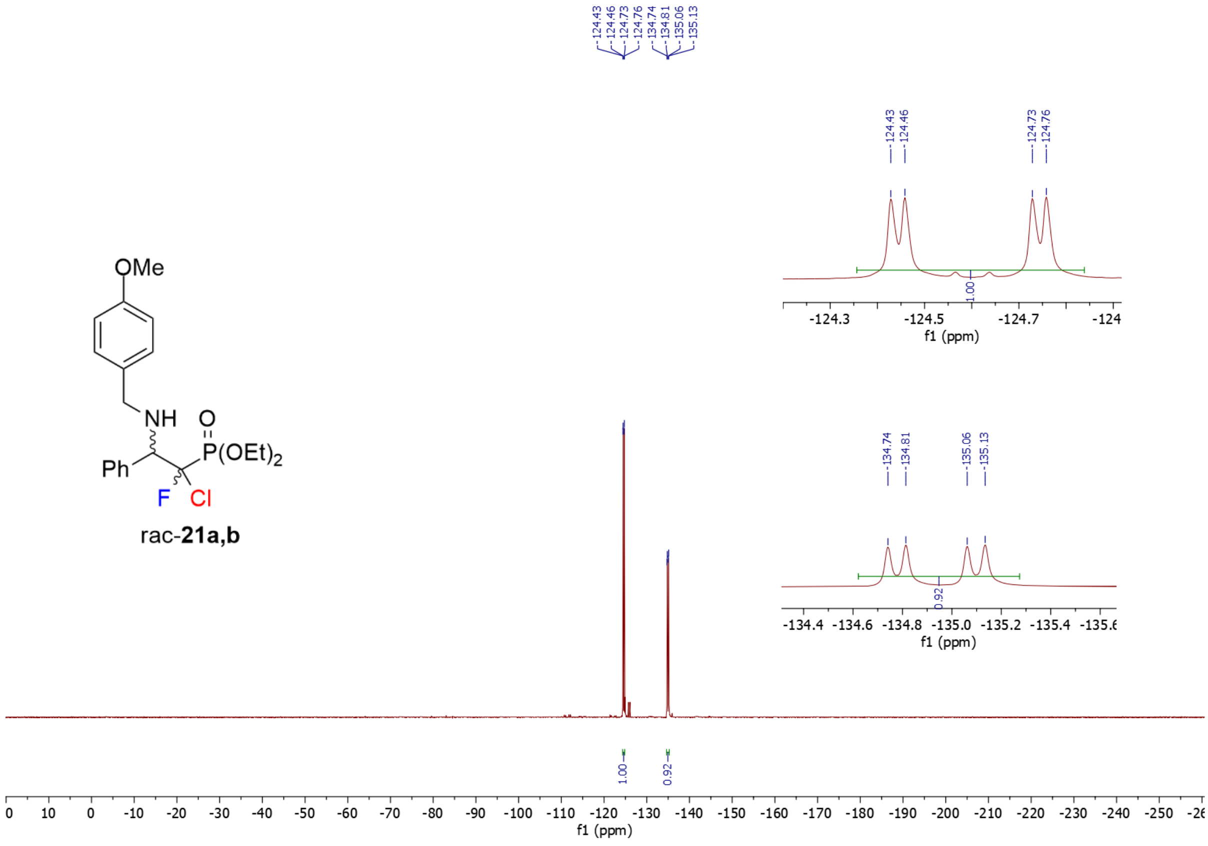

^{19}F NMR of rac-**21a,b** *dr* = 1:0.92

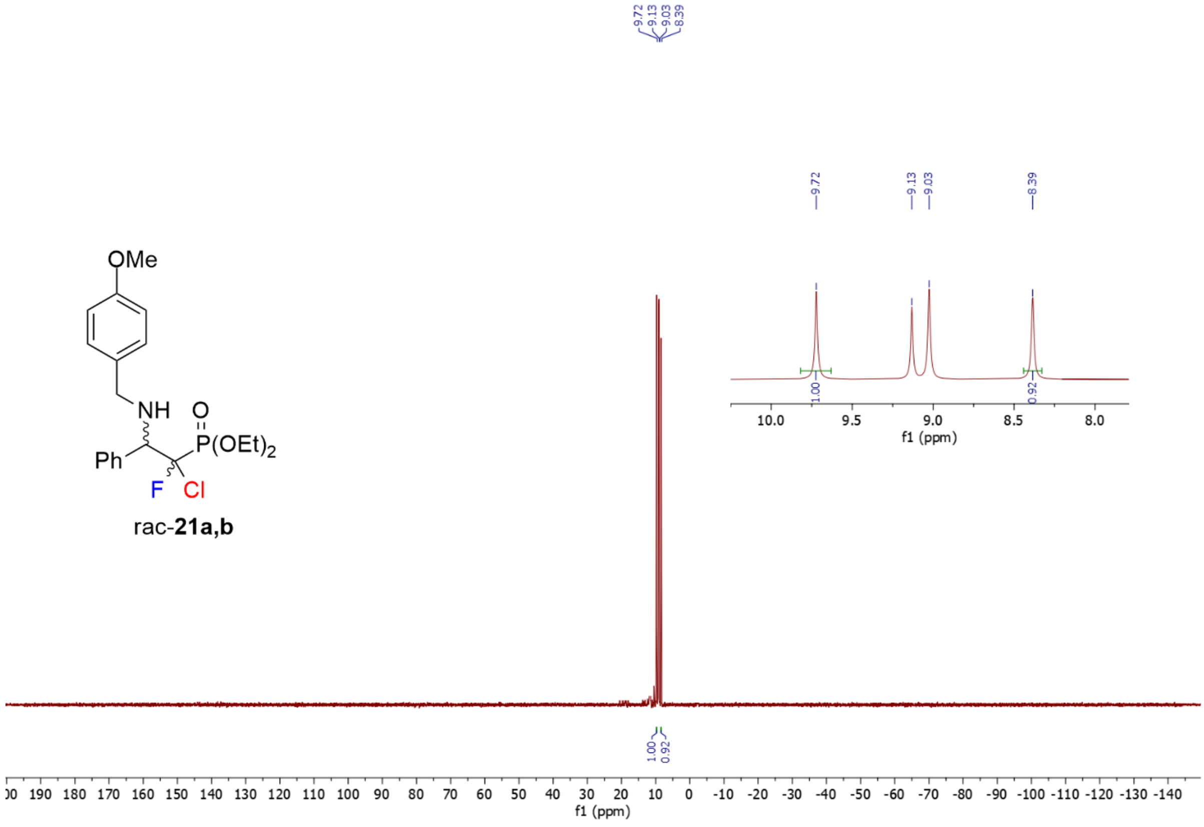

^{31}P{/^{1}H} NMR of rac-**21a,b** *dr* = 1:0.92

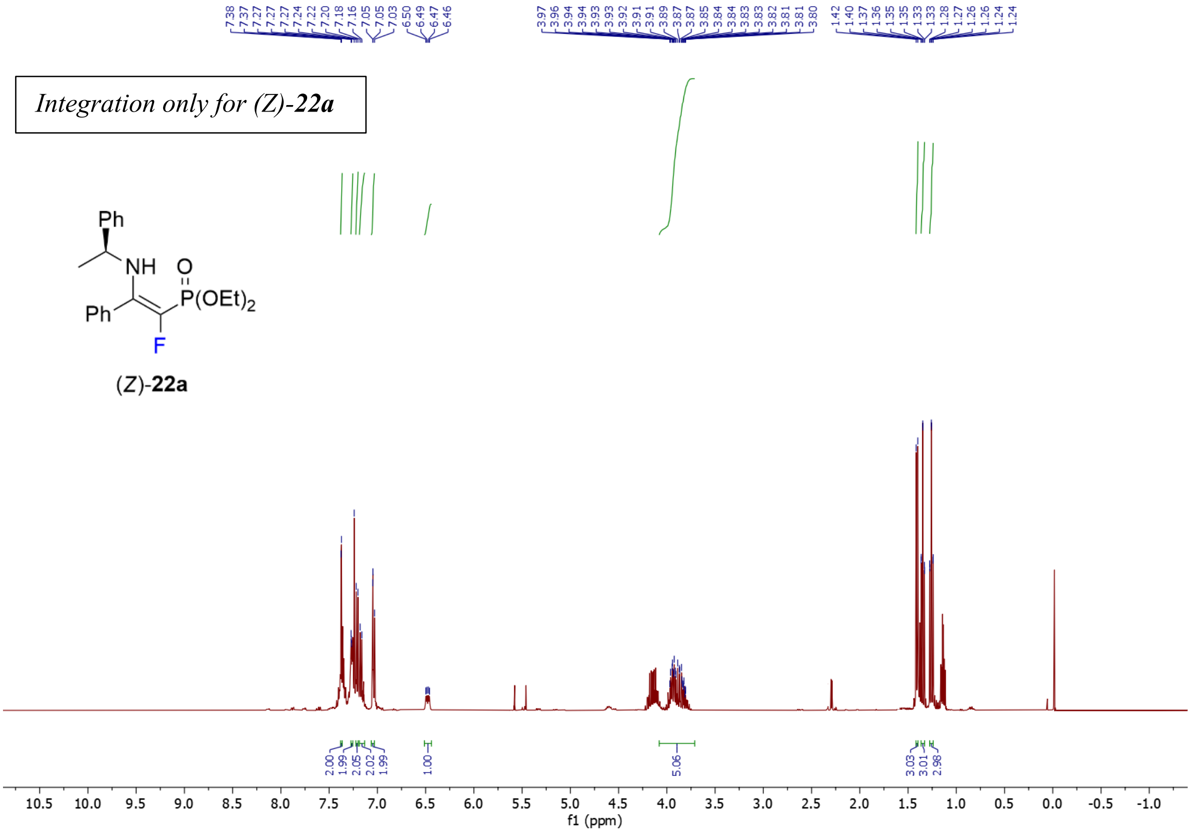

^{1}H NMR of enamine (**22a,b**)/imine (**23a,b**) ratio 1:0.05. (*Z*)-**22a**/(*E*)-**22b** ratio 1:0.31.

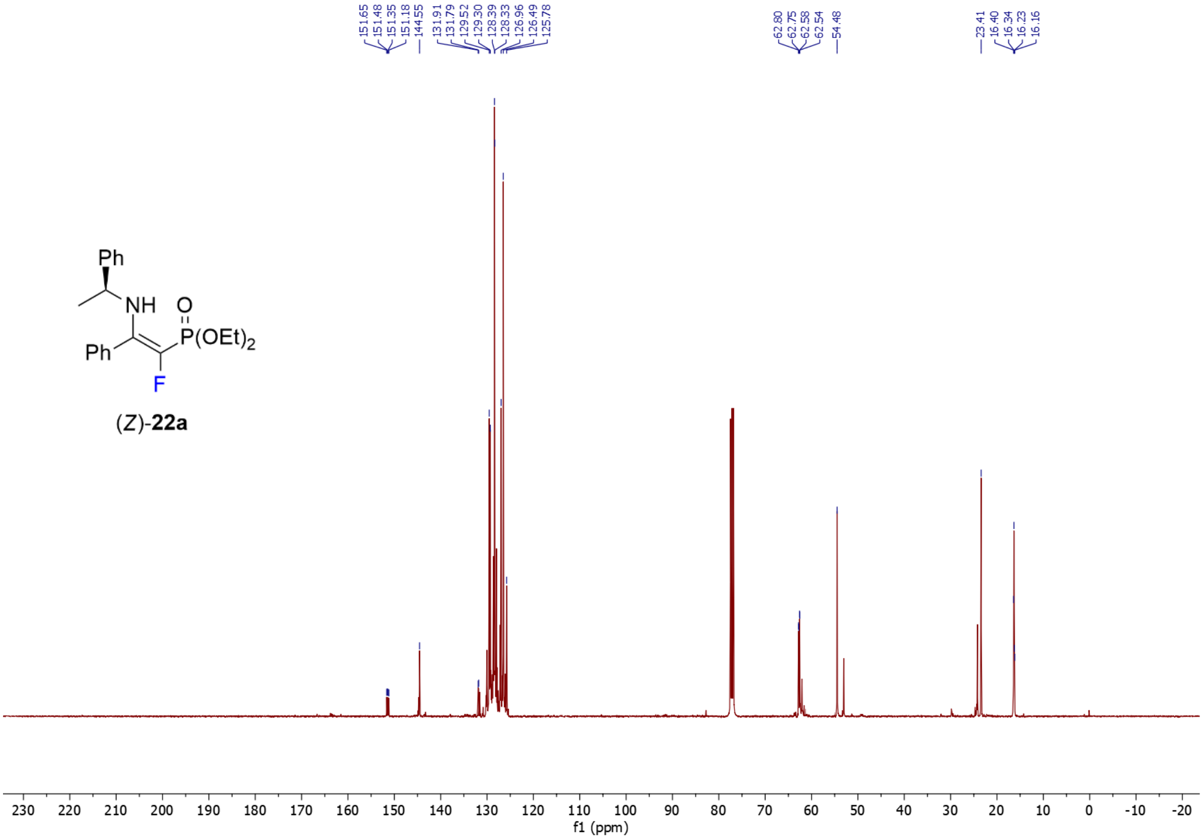

^{13}C NMR of enamine (**22a,b**)/imine (**23a,b**) ratio 1:0.05. (*Z*)-**22a**/(*E*)-**22b** ratio 1:0.31.

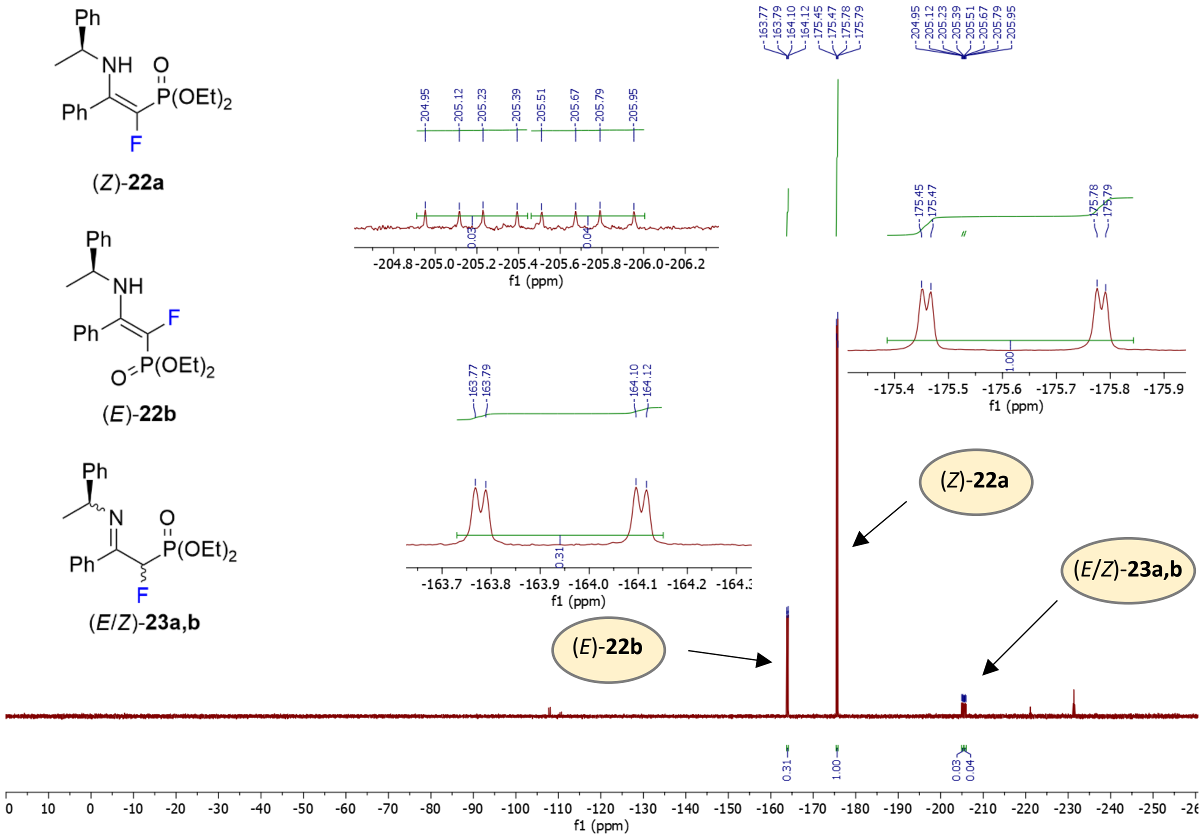

^{19}F NMR of enamine (**22a,b**)/imine (**23a,b**) ratio 1:0.05. (*Z*)**-22a**/(*E*)**-22b** ratio 1:0.31.

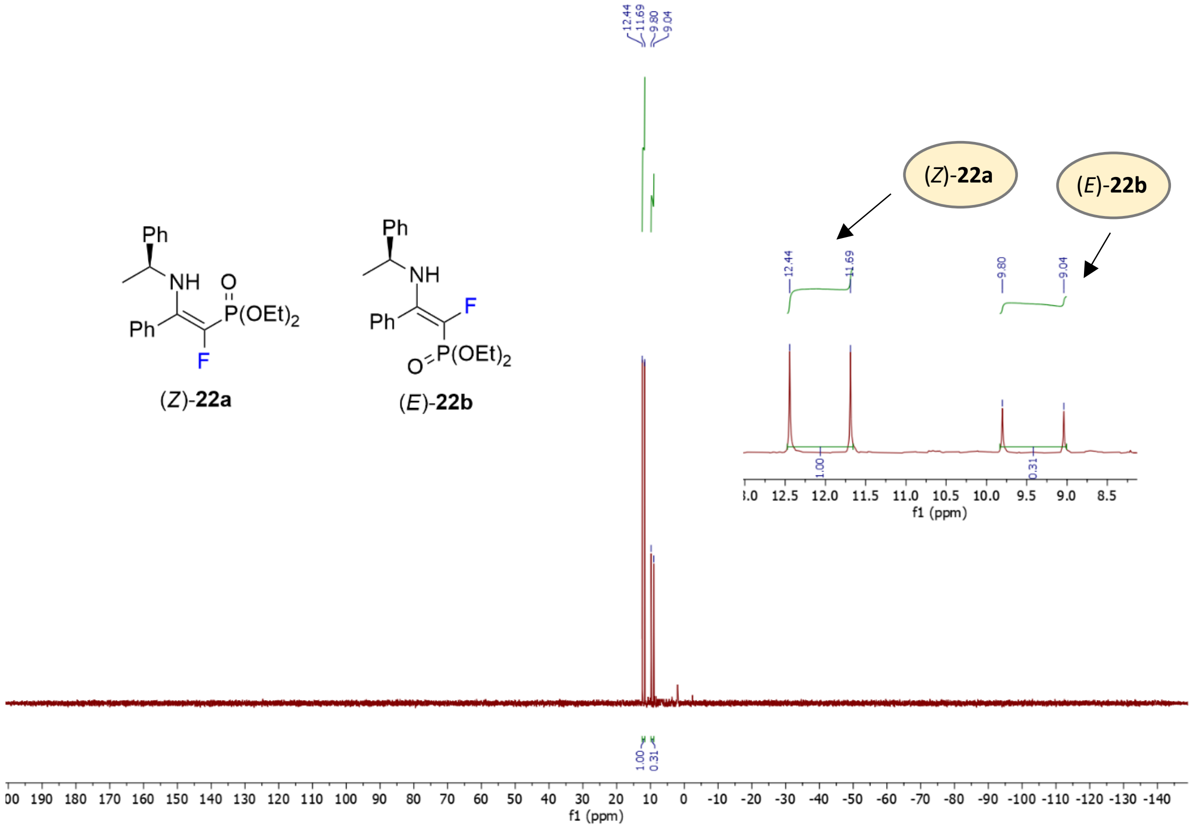

^{31}P NMR of enamine (**22a,b**)/imine (**23a,b**) ratio 1:0.05. (*Z*)**-22a**/(*E*)**-22b** ratio 1:0.31.

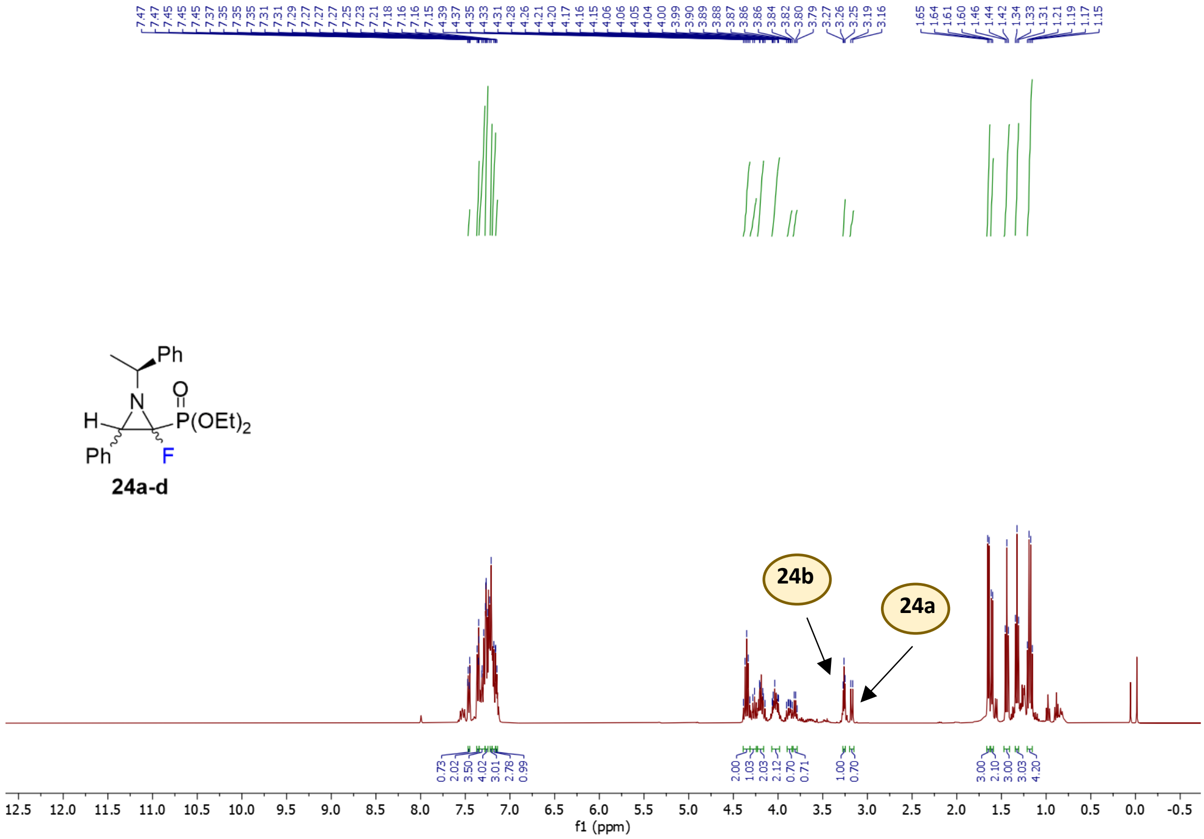

^{1}H NMR of **24a-d,** *dr* 0.70:1:0.17:0.04

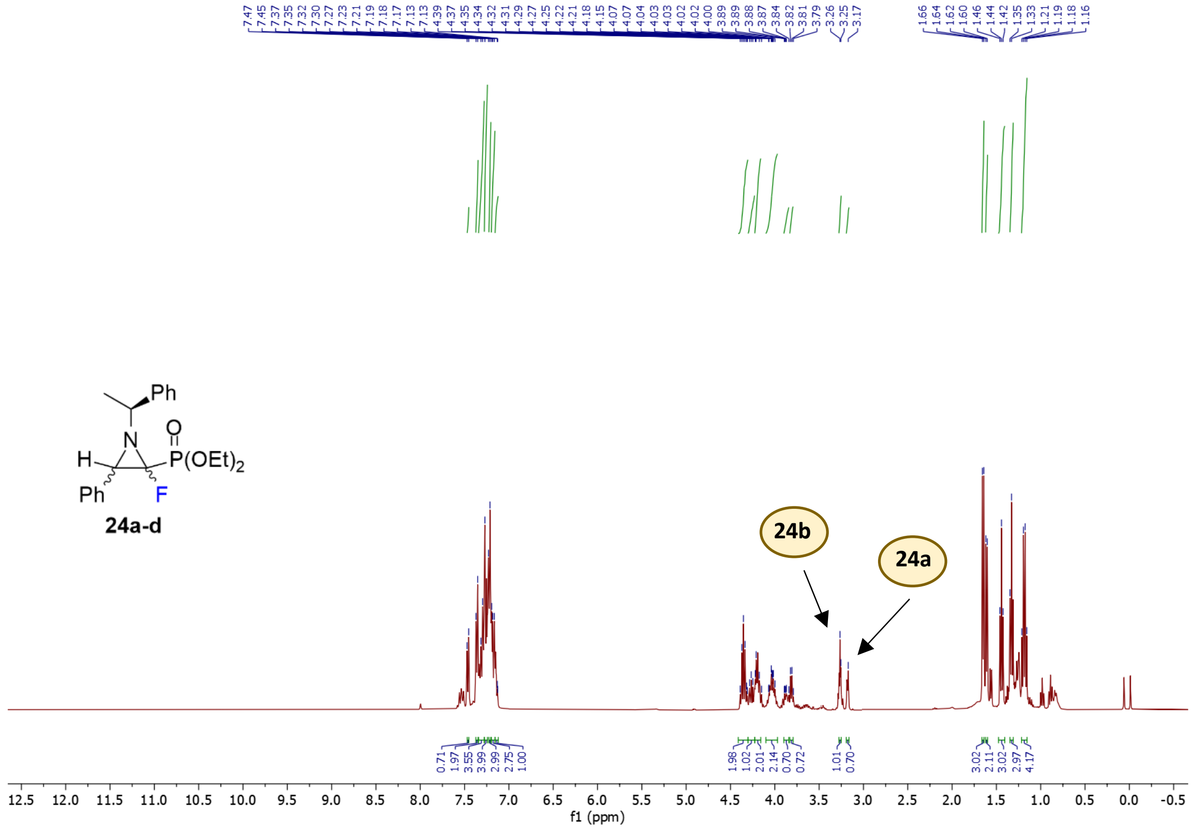

^{1}H{/^{19}F} NMR of **24a-d,** *dr* 0.70:1:0.17:0.04

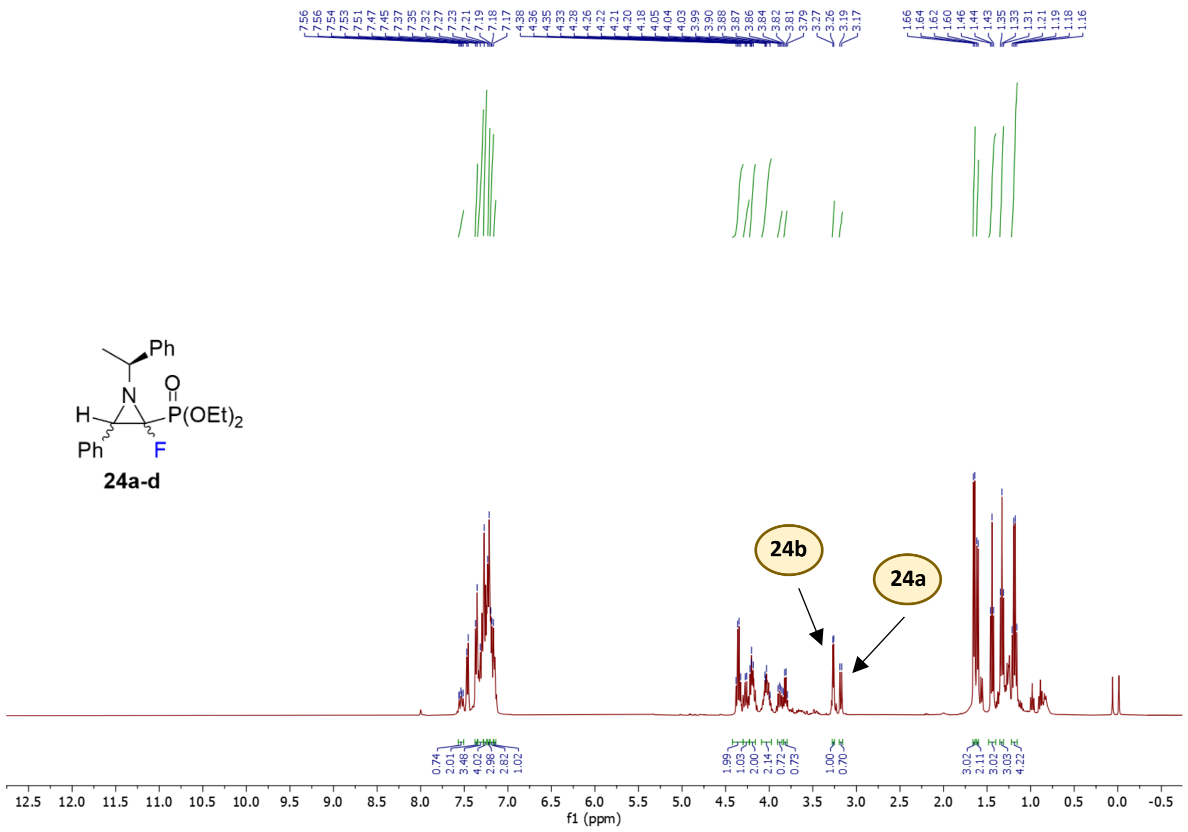

$^{1}H\{/^{31}P\}$ NMR of **24a-d,** *dr* 0.70:1:0.17:0.04

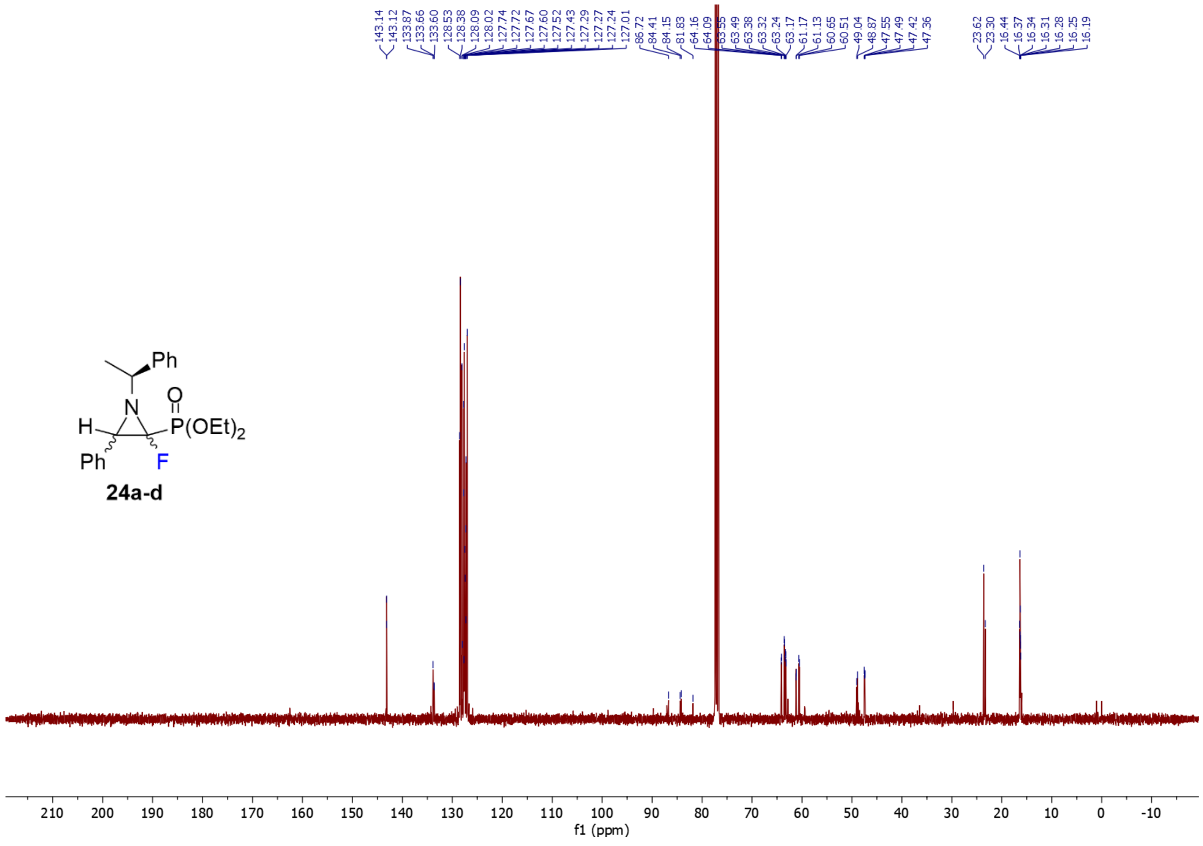

^{13}C NMR of **24a-d,** *dr* 0.70:1:0.17:0.04

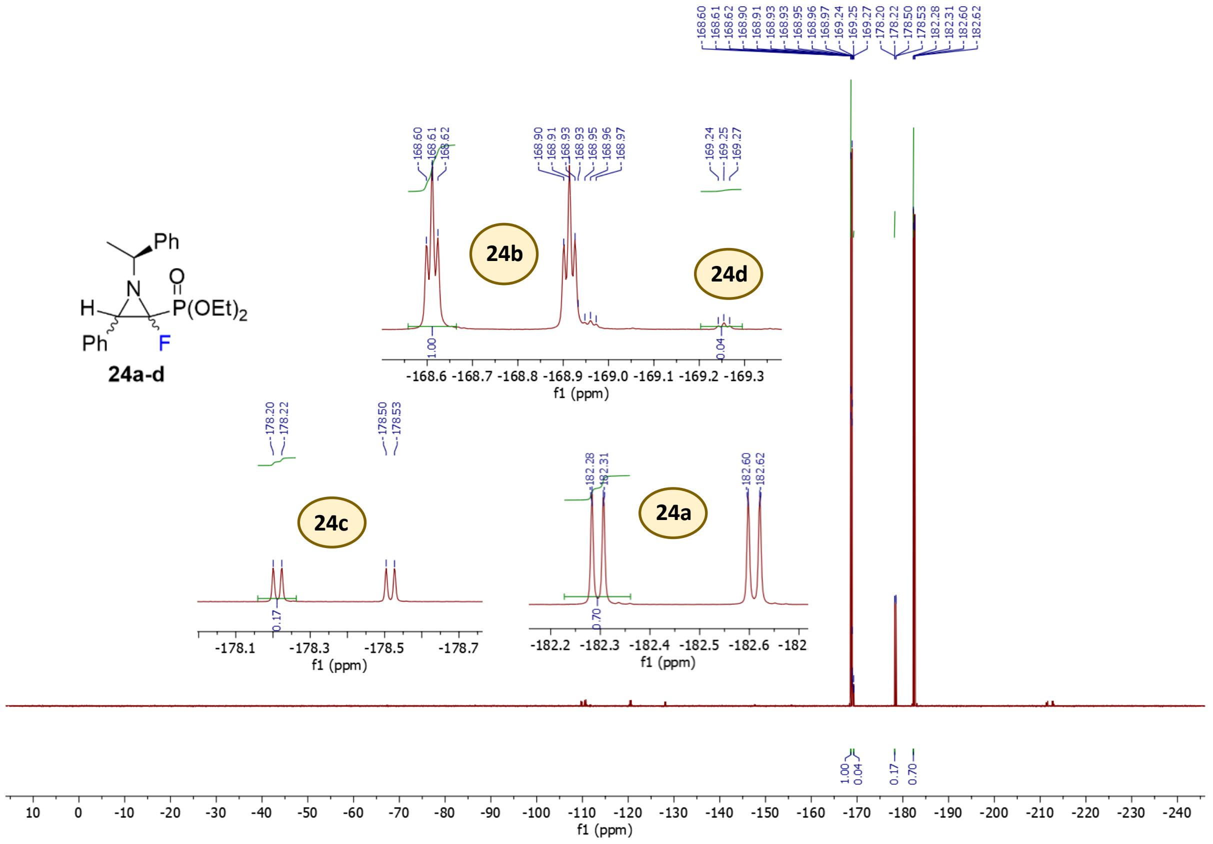

^{19}F NMR of **24a-d,** *dr* 0.70:1:0.17:0.04

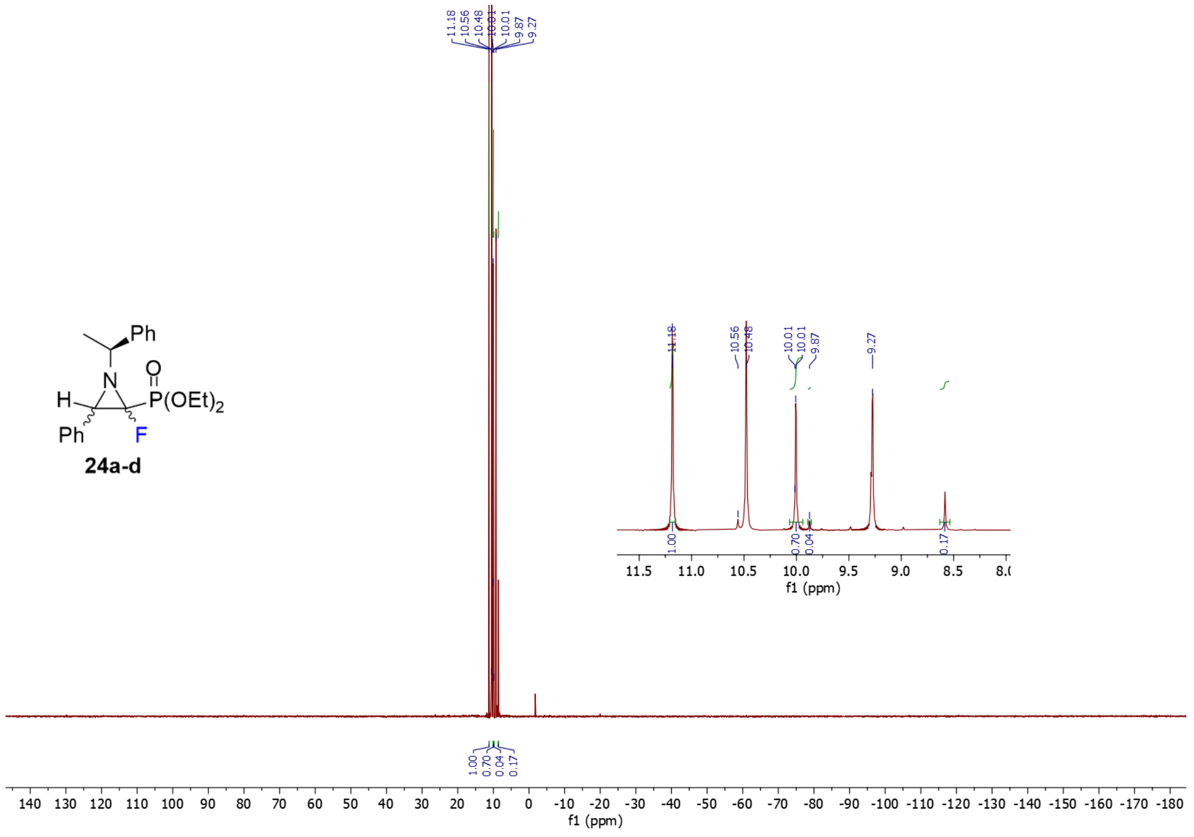

^{31}P{/^{1}H} NMR of **24a-d,** *dr* 0.70:1:0.17:0.04

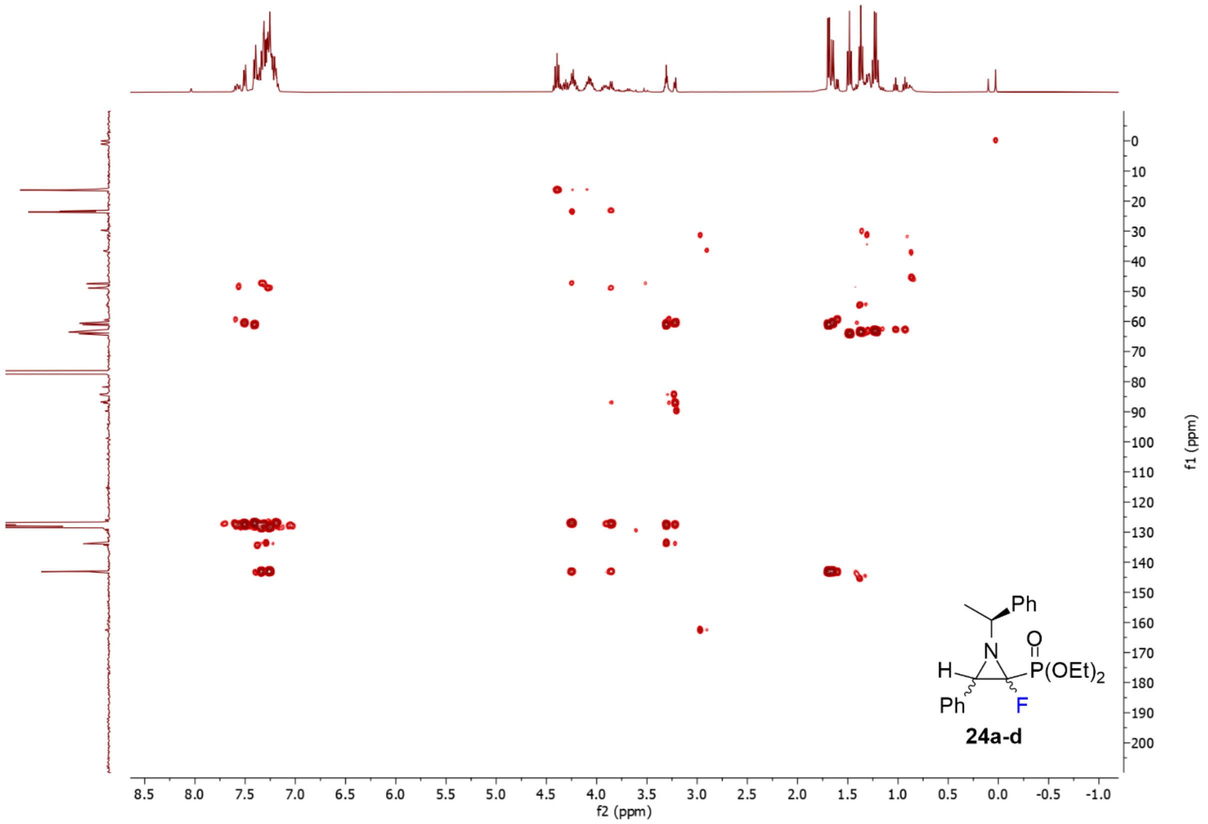

^{1}H-^{13}C HMBC NMR of **24a-d,** *dr* 1:0.70:0.04:0.17

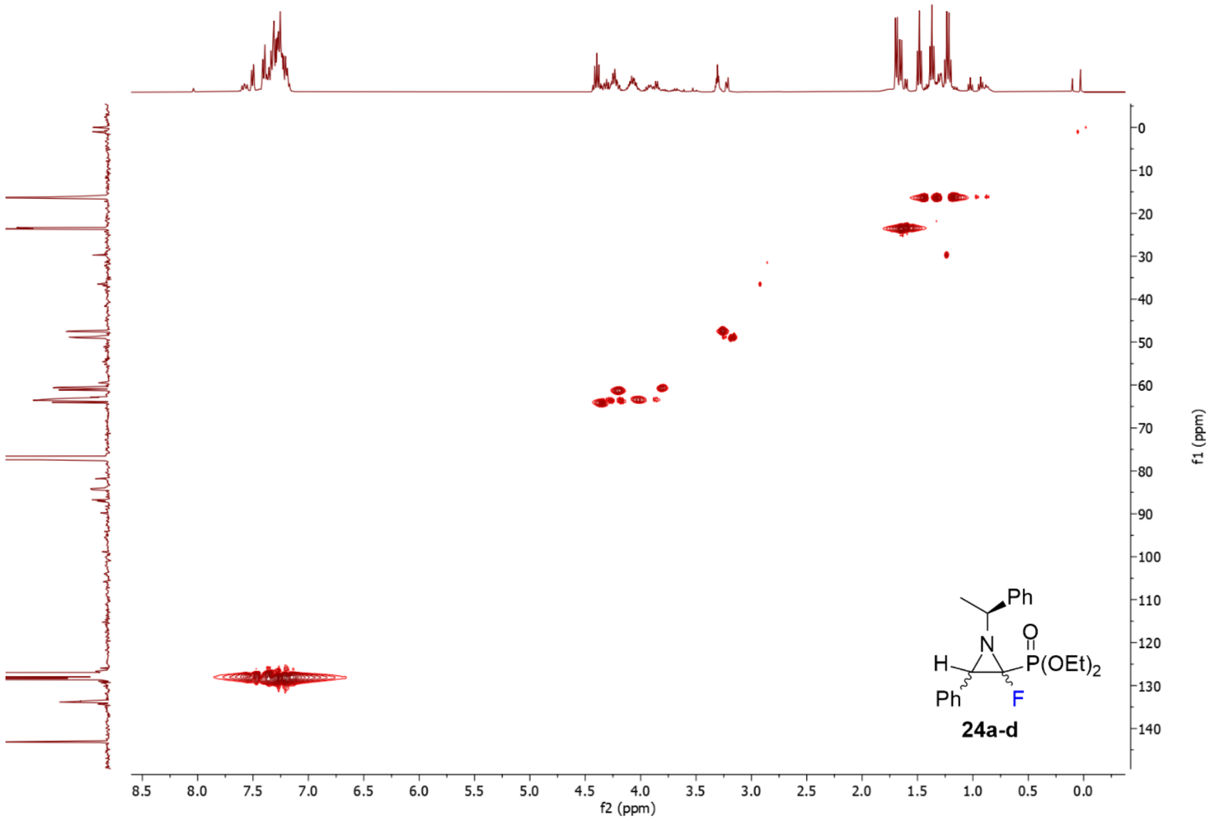

^{1}H-^{13}C HSQC NMR of **24a-d,** *dr* 1:0.70:0.04:0.17

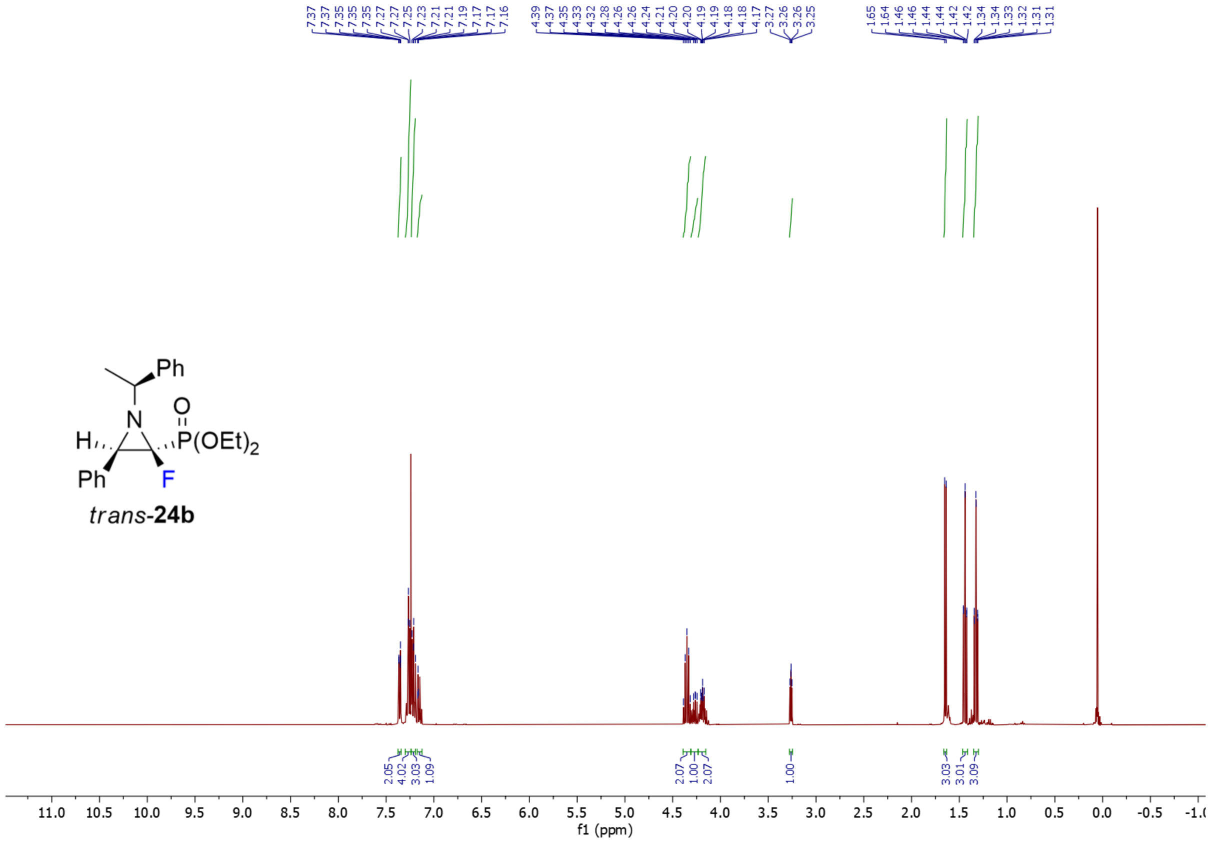

^{1}H NMR of *trans*-**24b**

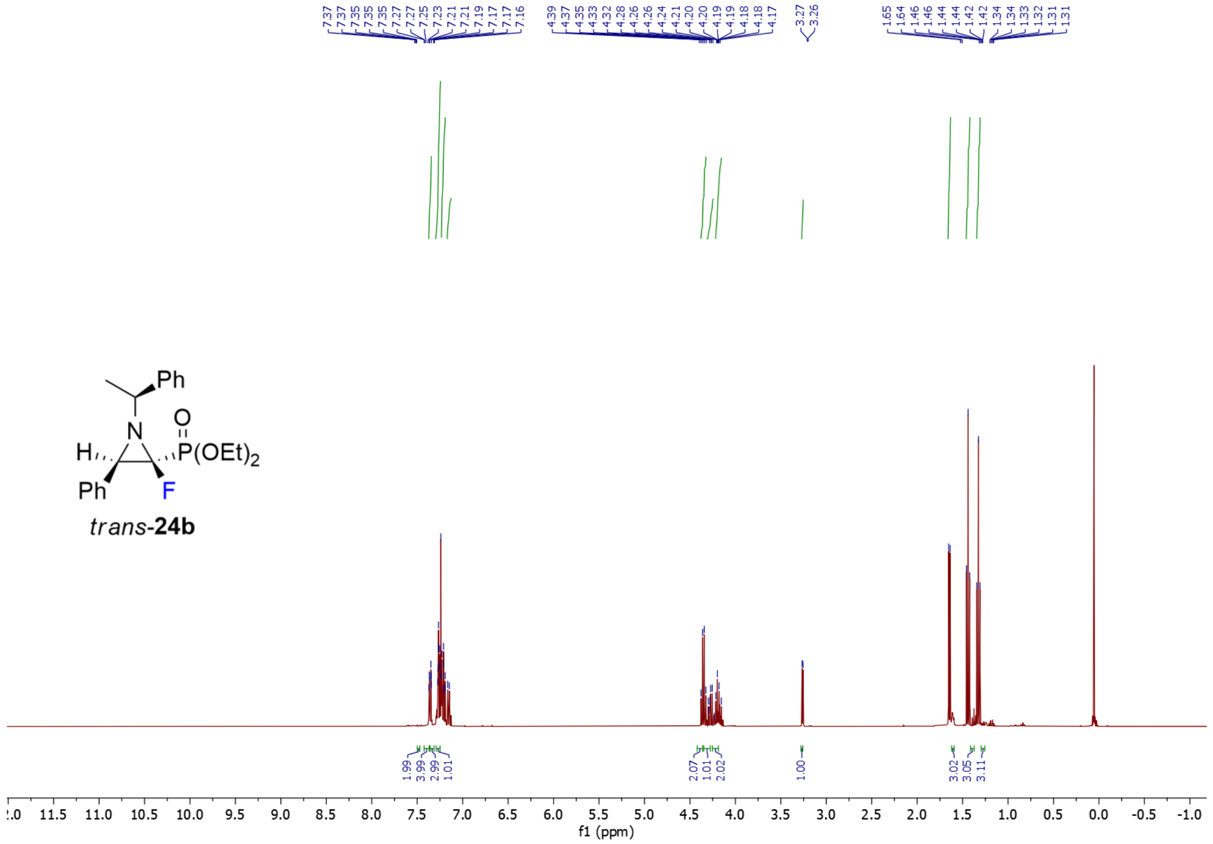

$^{1}H\{/^{19}F\}$ NMR of *trans*-**24b**

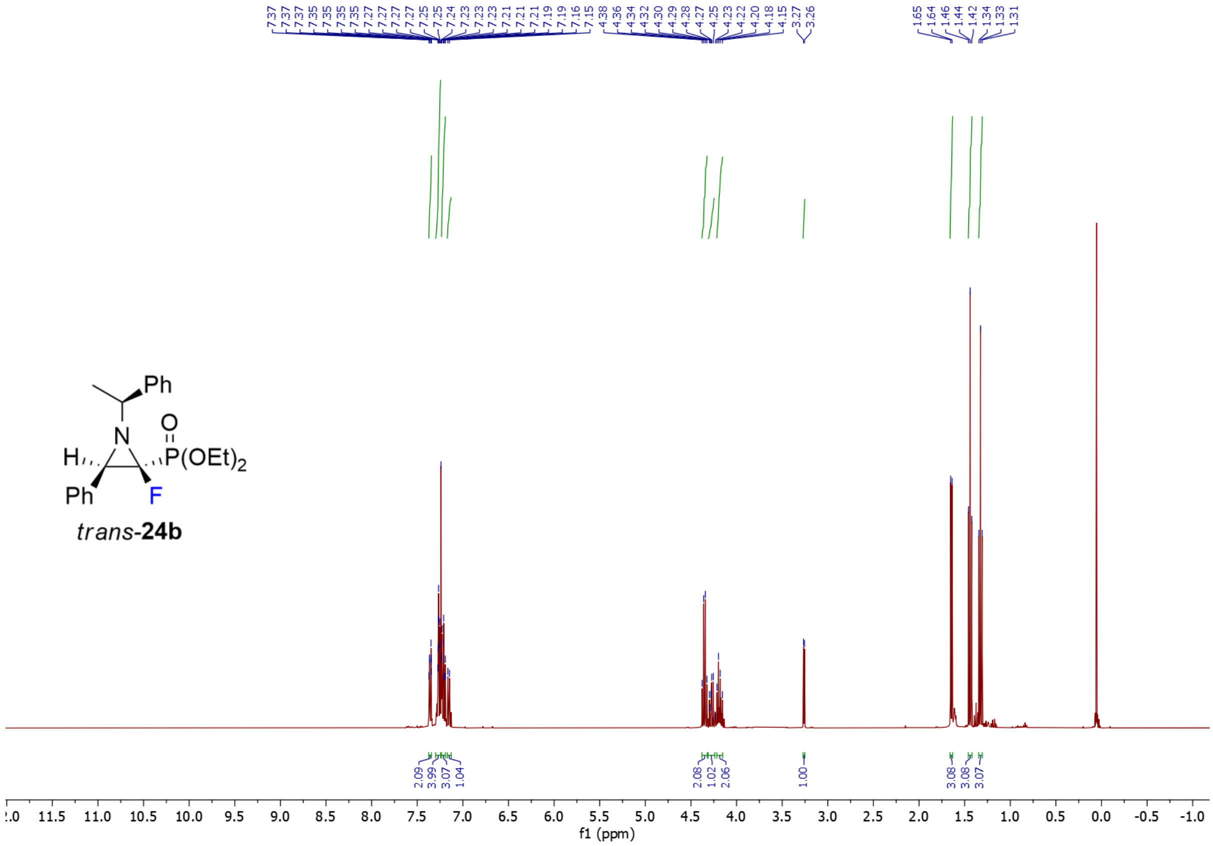

^{1}H{/^{31}P} NMR of *trans*-**24b**

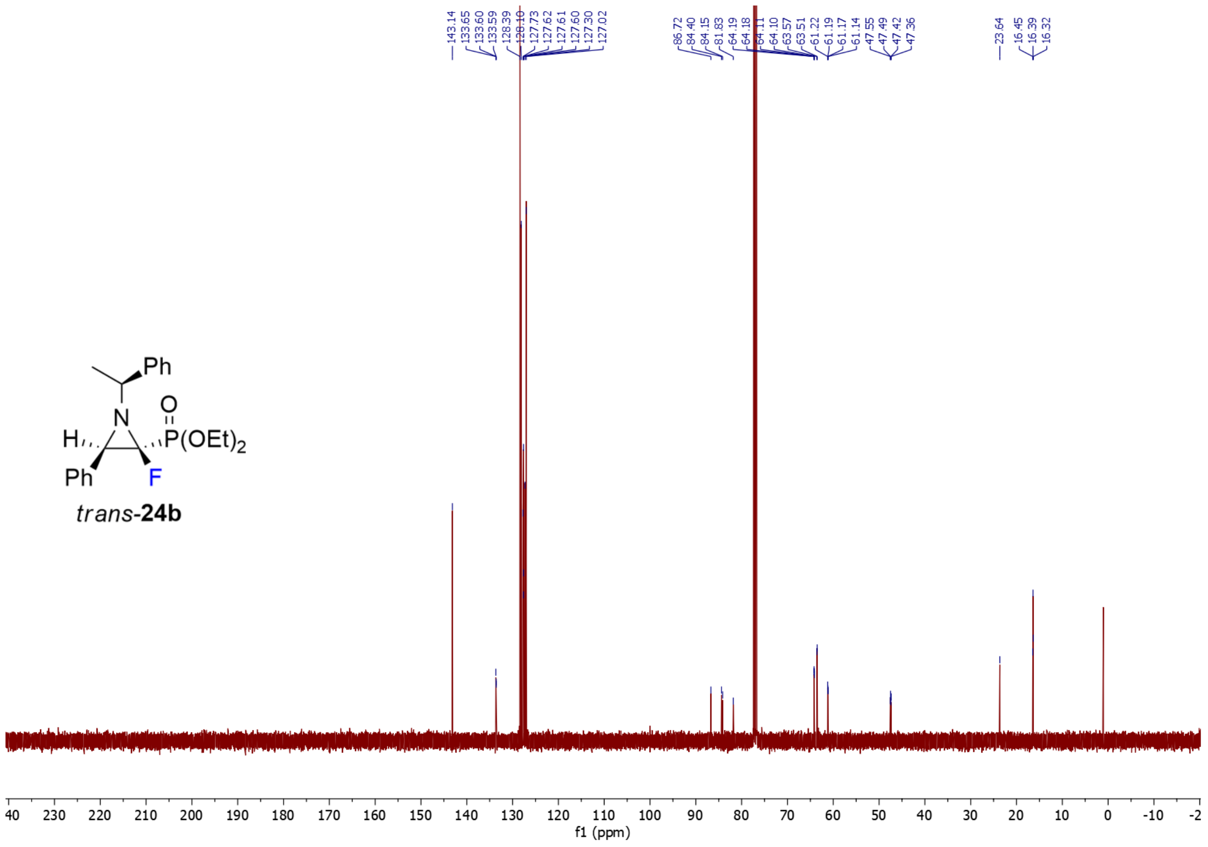

^{13}C NMR of *trans*-**24b**

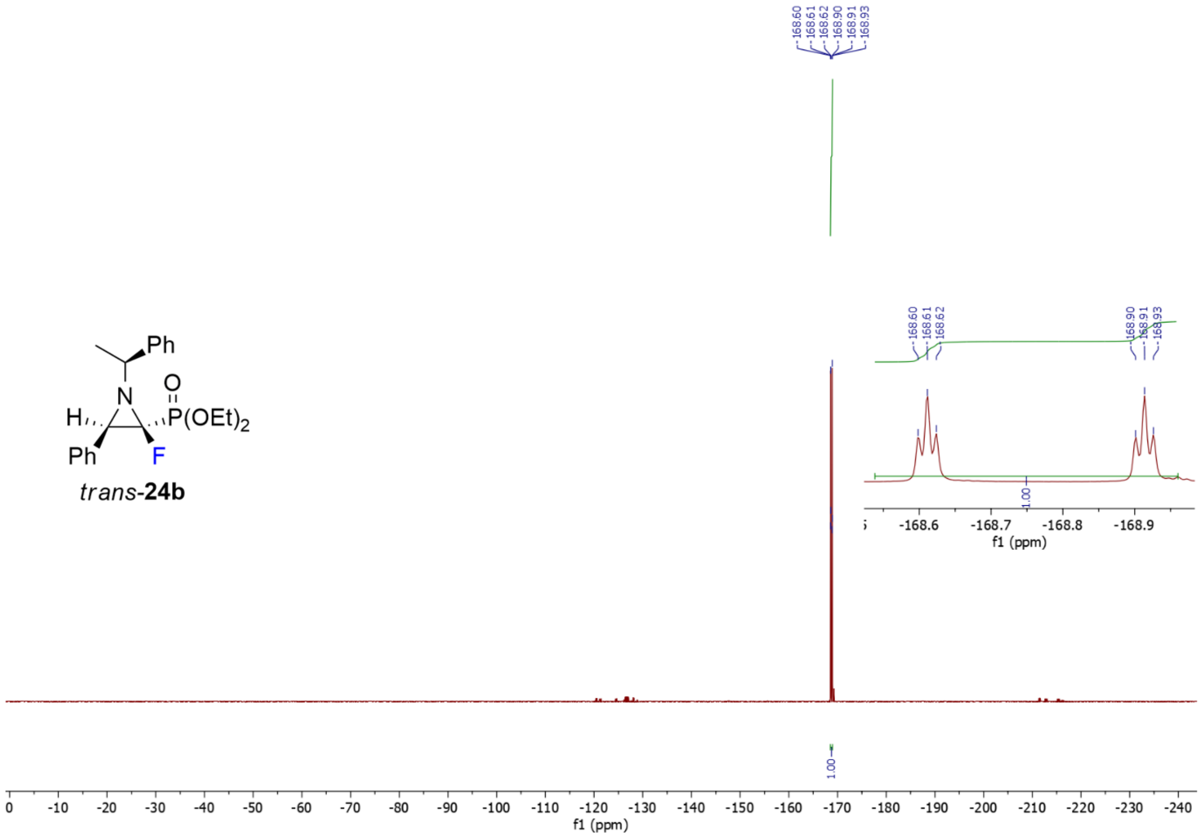

^{19}F NMR of *trans*-**24b**

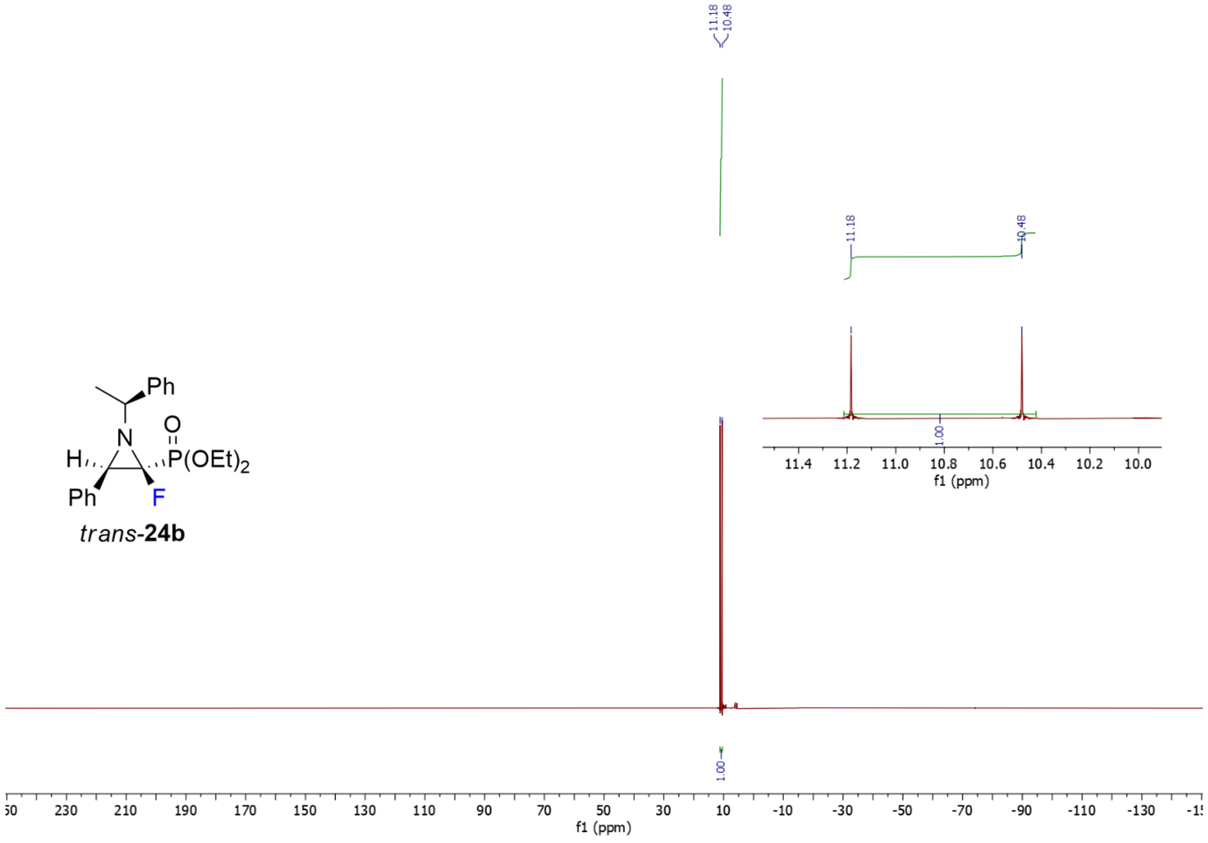

^{31}P{/^{1}H} NMR of *trans*-**24b**

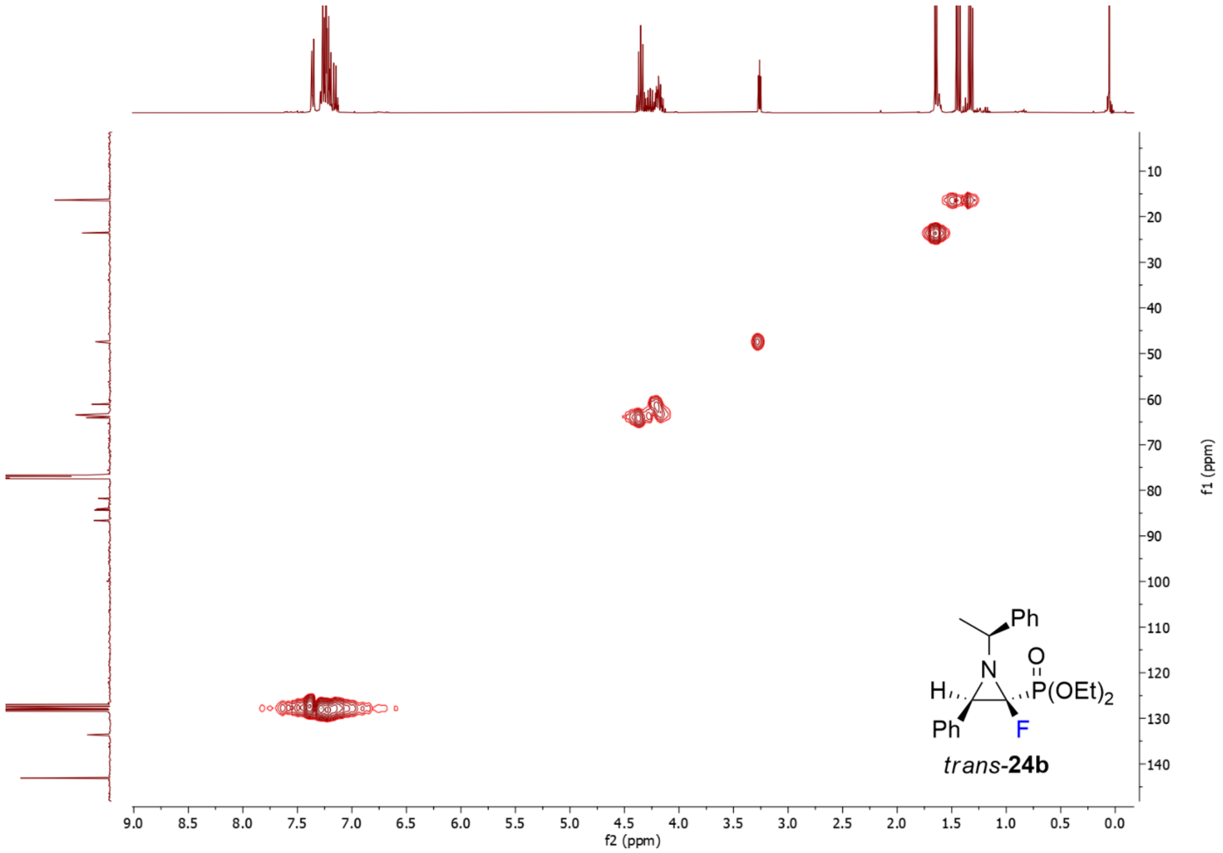

^{1}H-^{13}C HSQC NMR of *trans*-**24b**

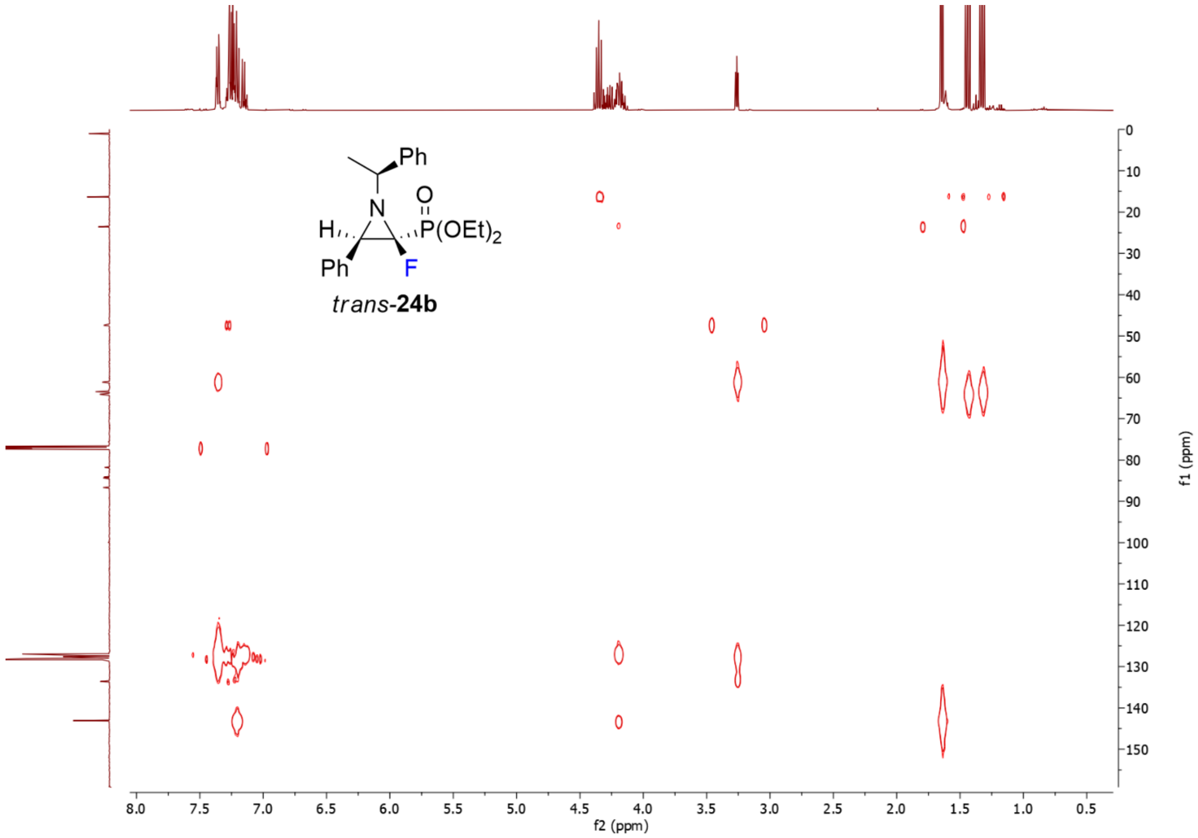

^{1}H-^{13}C HMBC NMR of of *trans*-**24b**

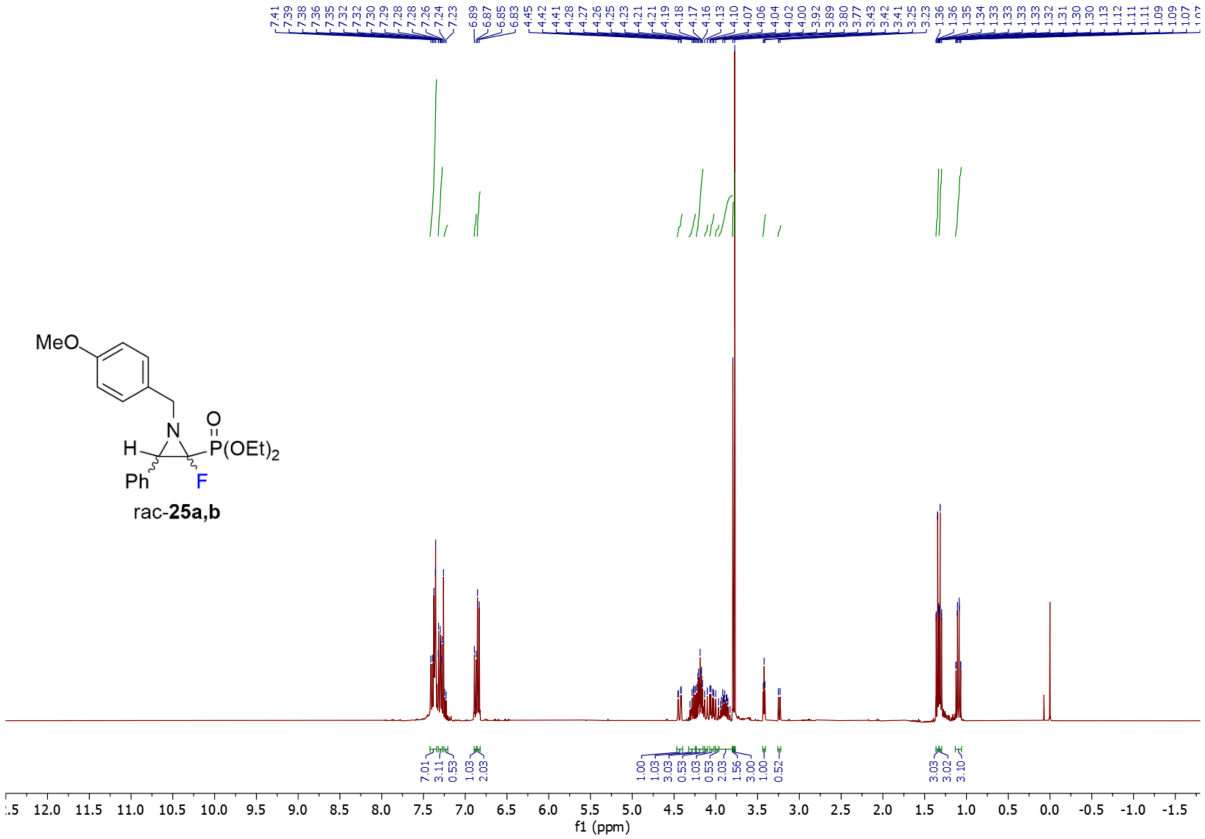

^{1}H NMR of rac-**25a,b** *dr* 0.52:1

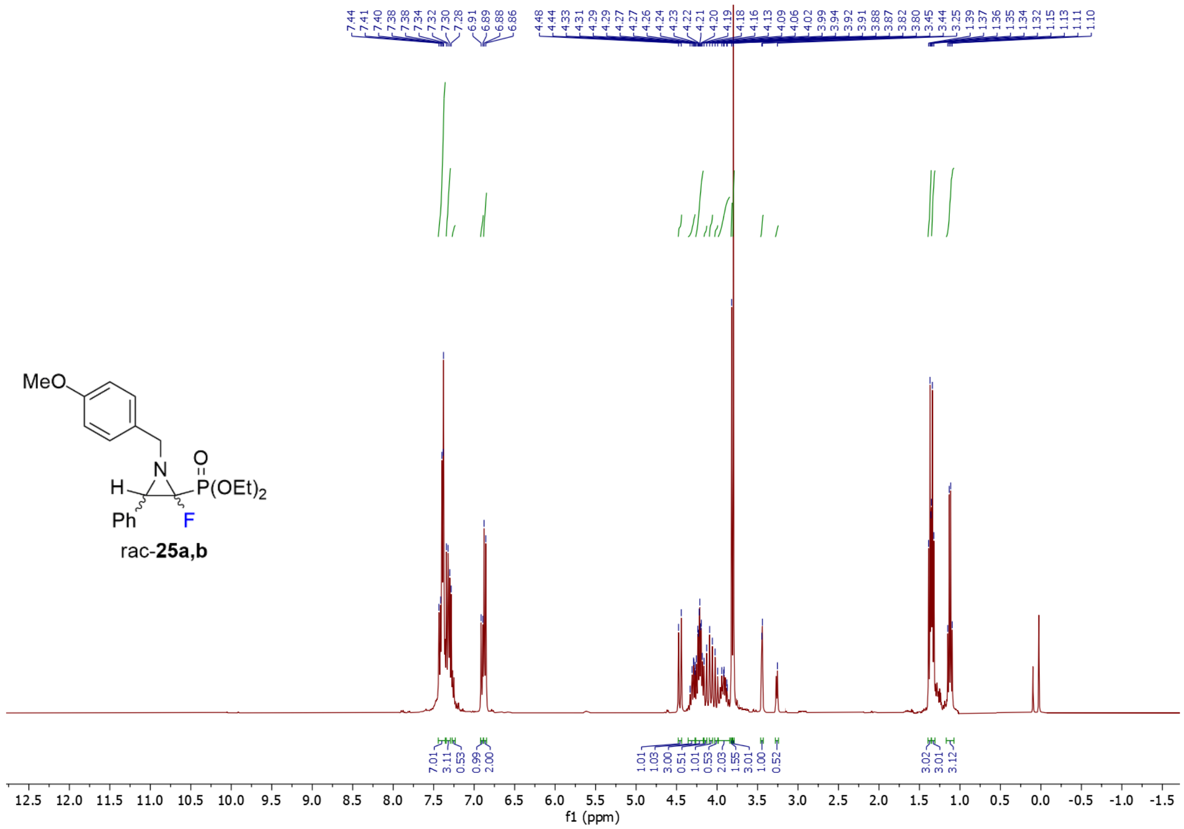

$^{1}H\{/^{19}F\}$ NMR of rac-**25a,b** *dr* 0.52:1

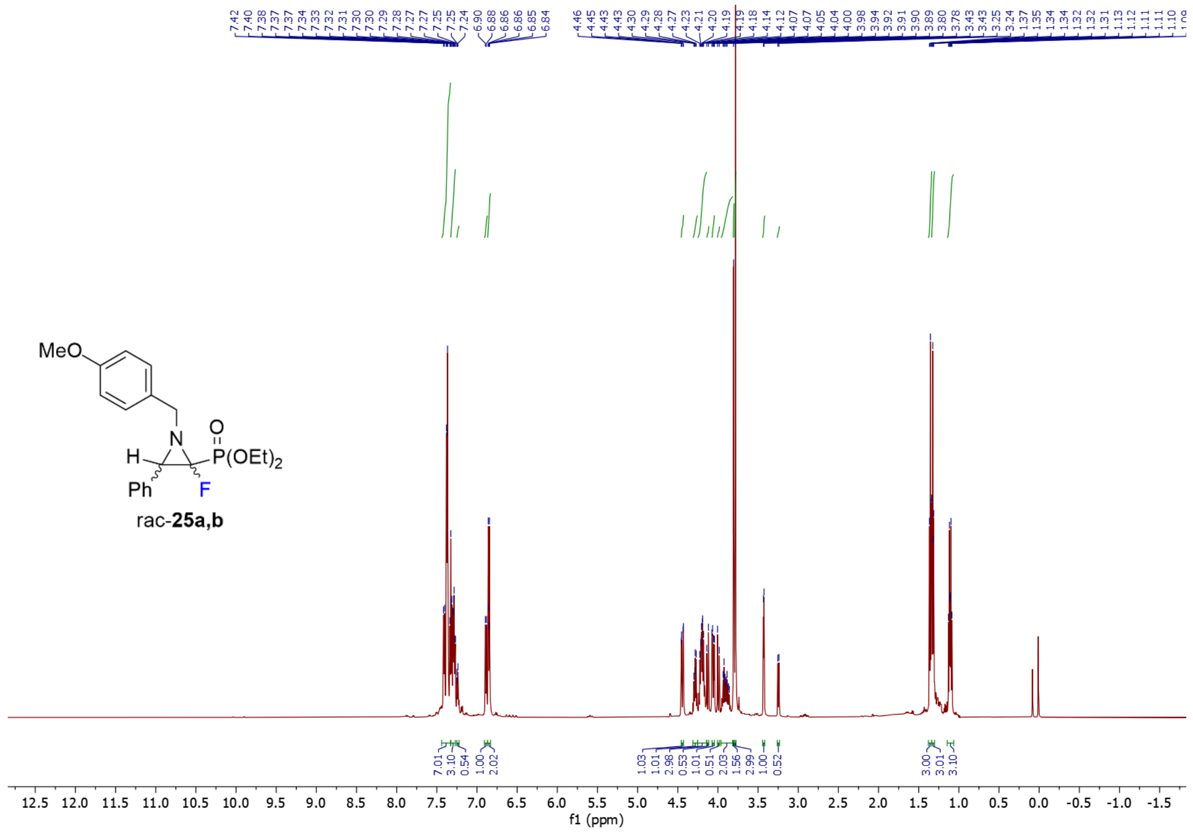

$^{1}H\{/^{31}P\}$ NMR of rac-**25a,b** *dr* 0.52:1

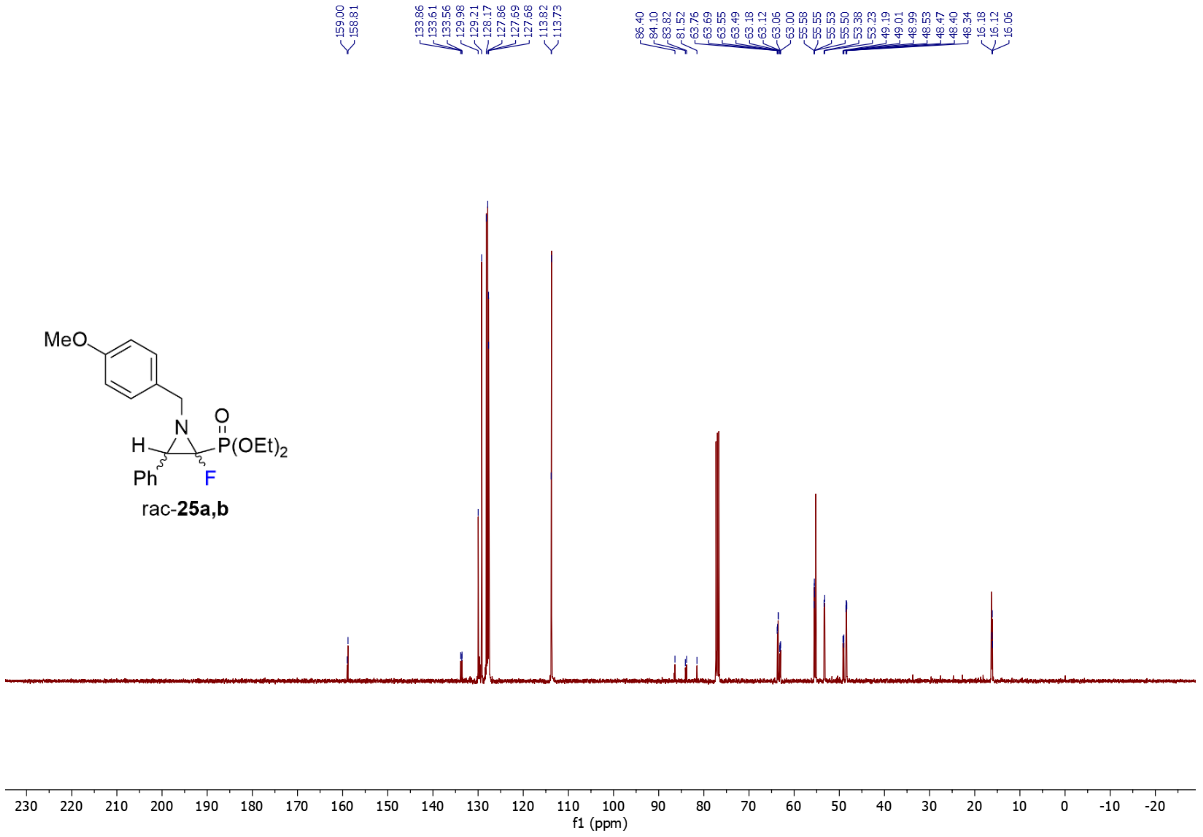

^{13}C NMR of rac-**25a,b** *dr* 0.52:1

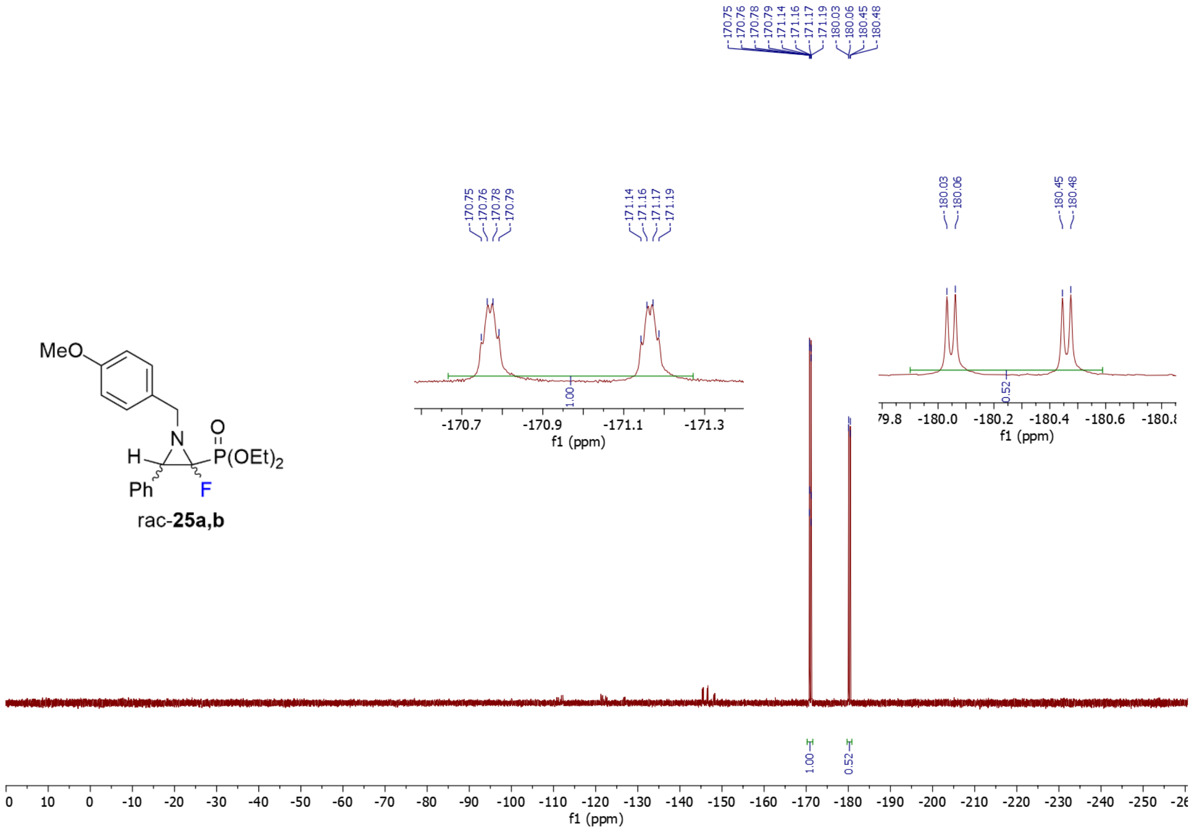

^{19}F NMR of rac-**25a,b** *dr* 0.52:1

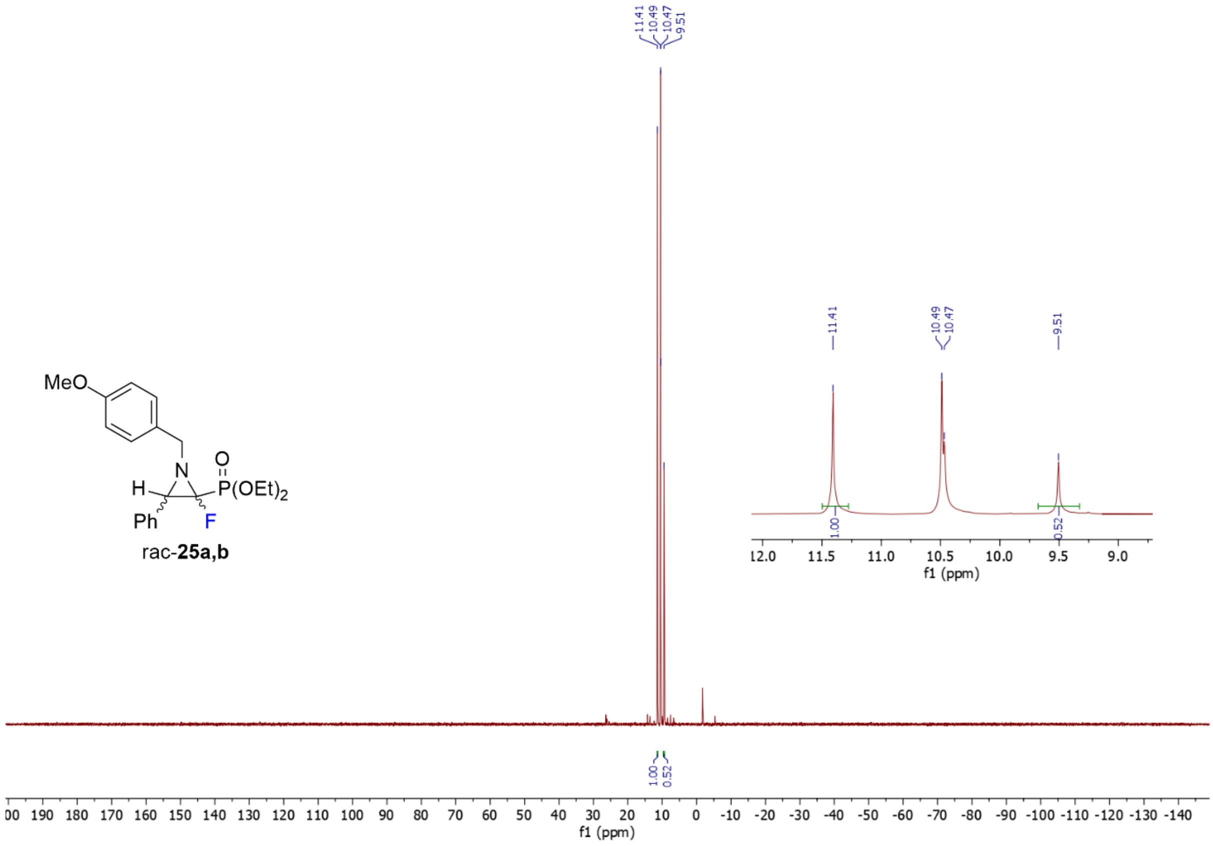

^{31}P{/^{1}H} NMR of rac-**25a,b** *dr* 0.52:1

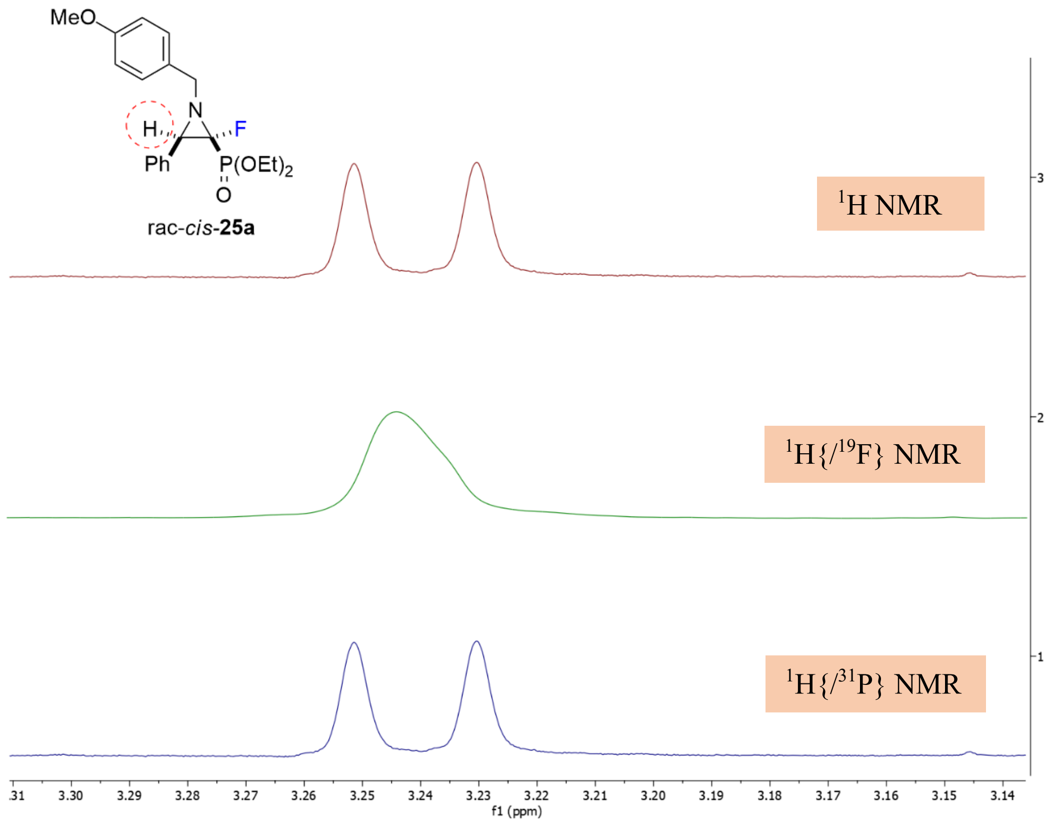

Aziridine proton decoupling of rac-*cis*-**25a**

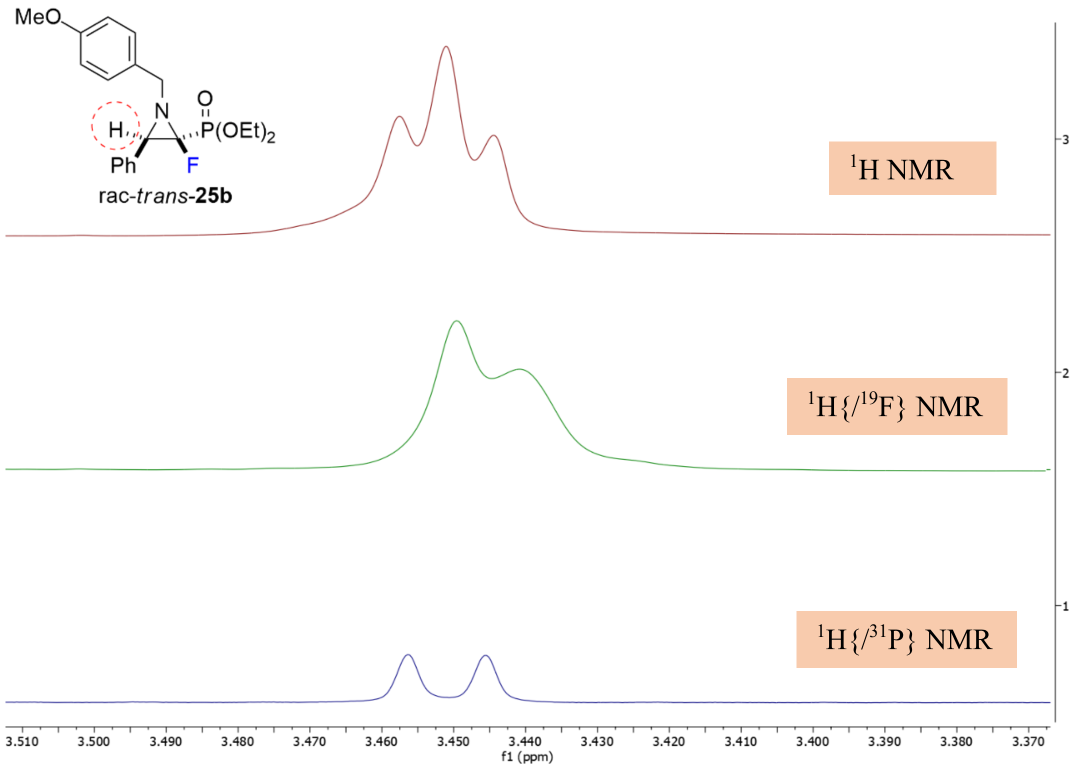

Aziridine proton decoupling of rac-*trans*-**25b**

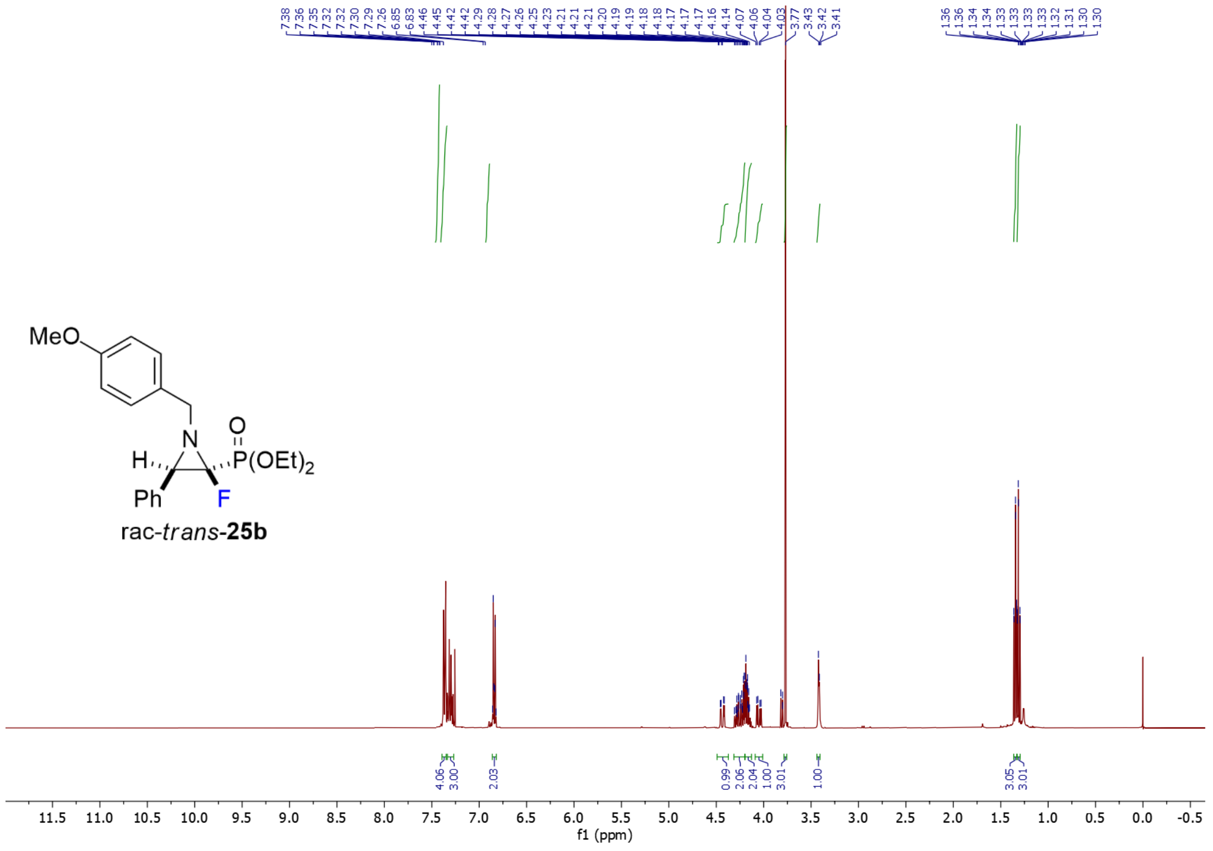

^{1}H NMR of rac-*trans*-**25b**

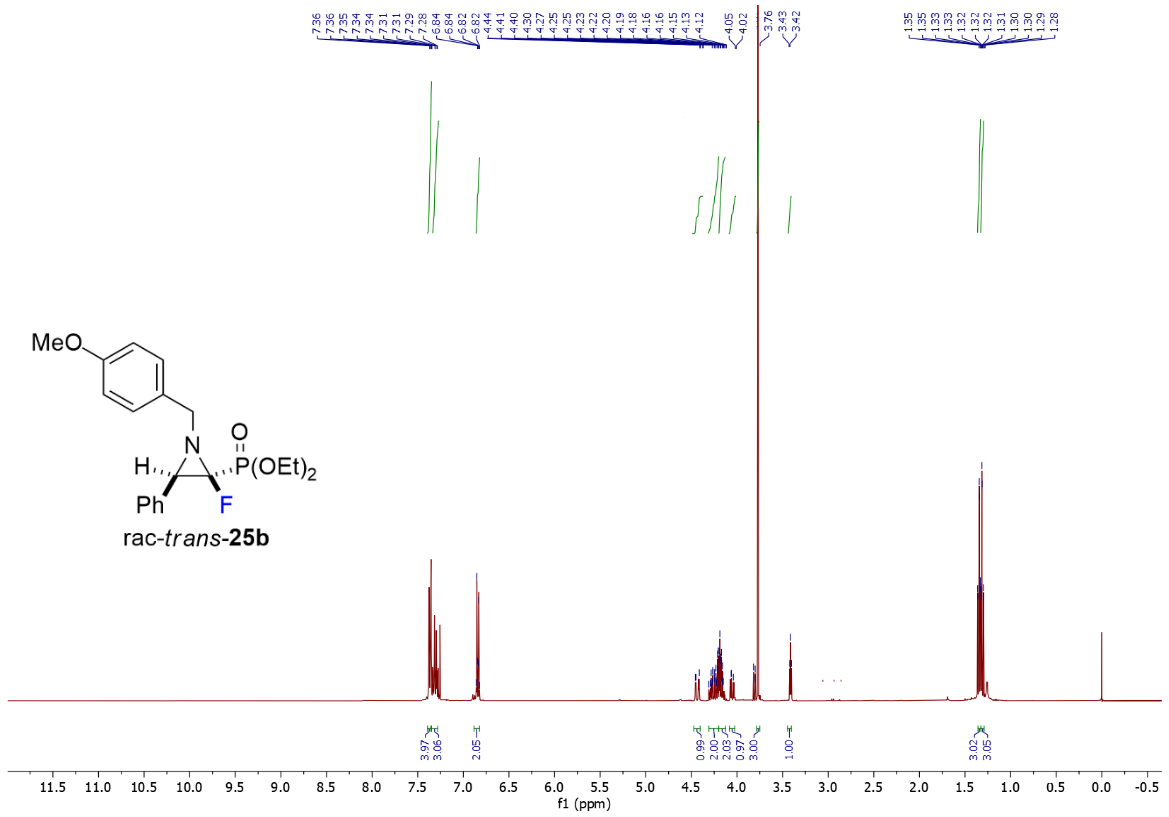

^{1}H{/^{19}F} NMR of rac-*trans*-**25b**

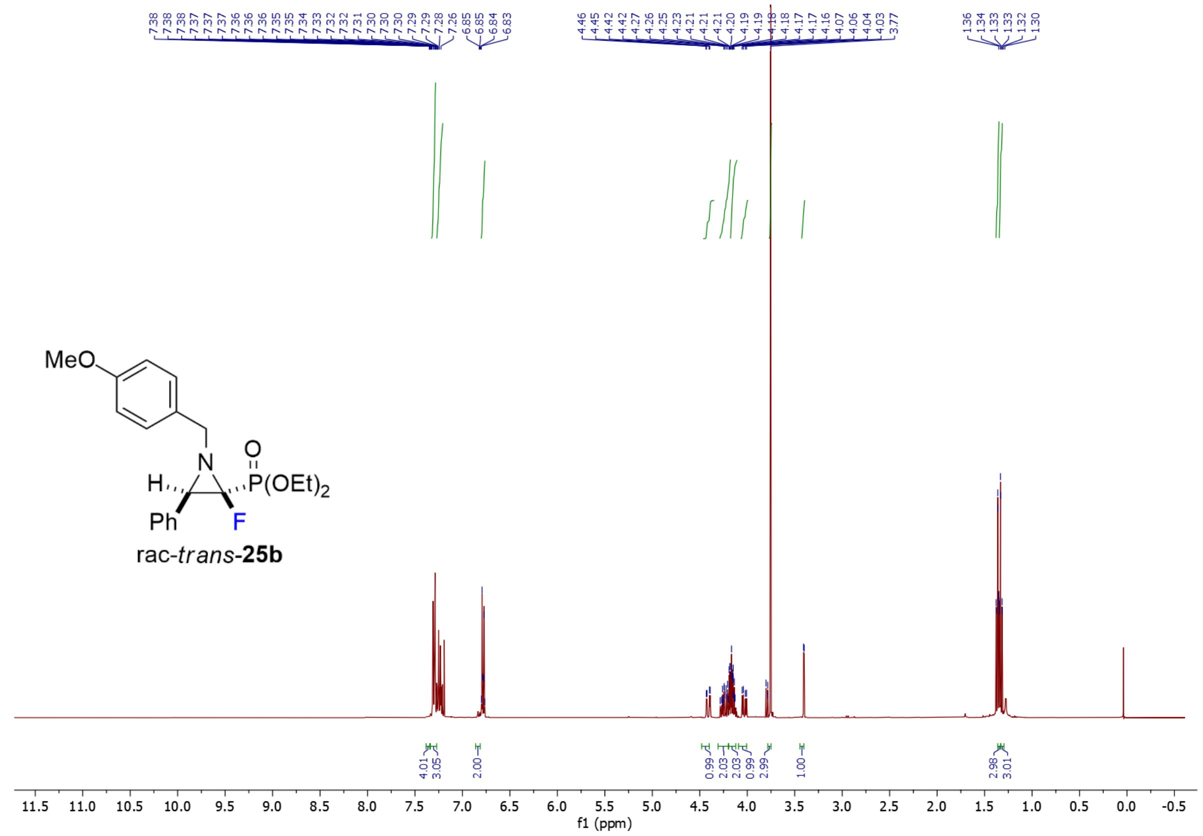

^{1}H{/^{31}P} NMR of rac-*trans*-**25b**

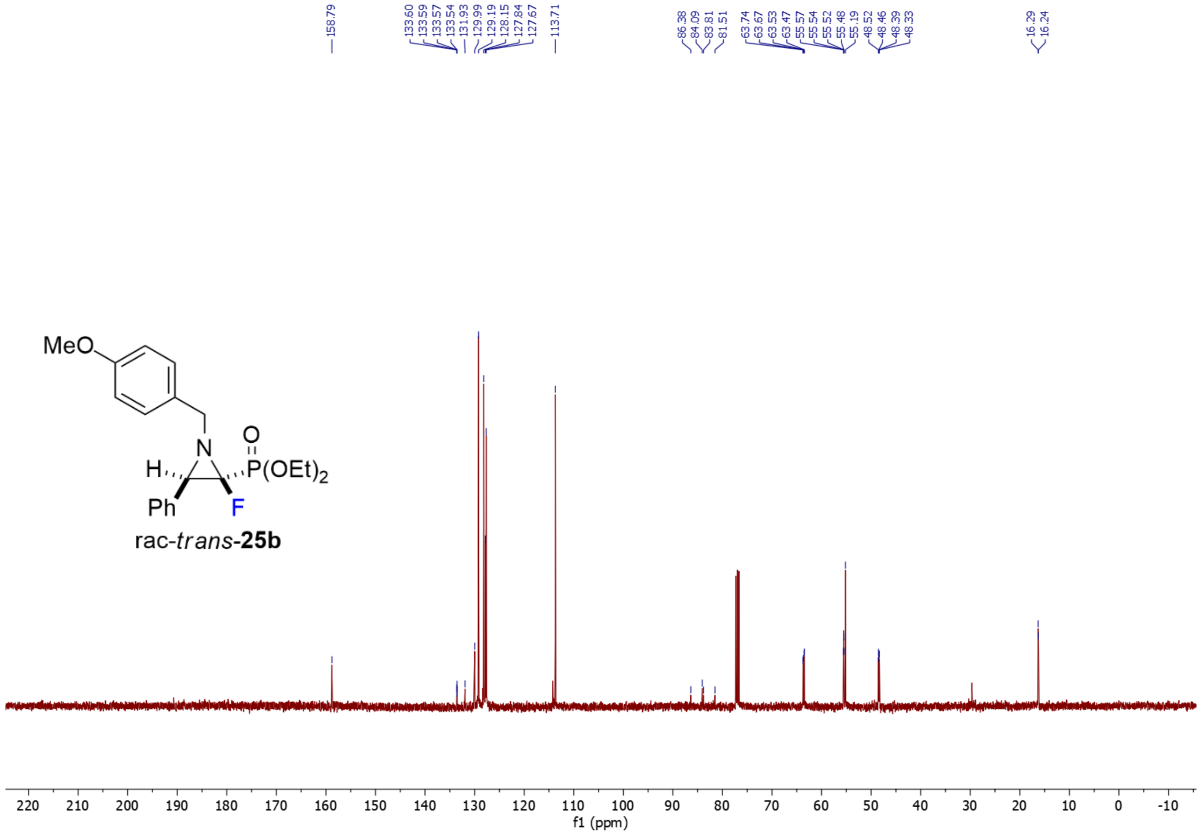

^{13}C NMR of rac-*trans*-**25b**

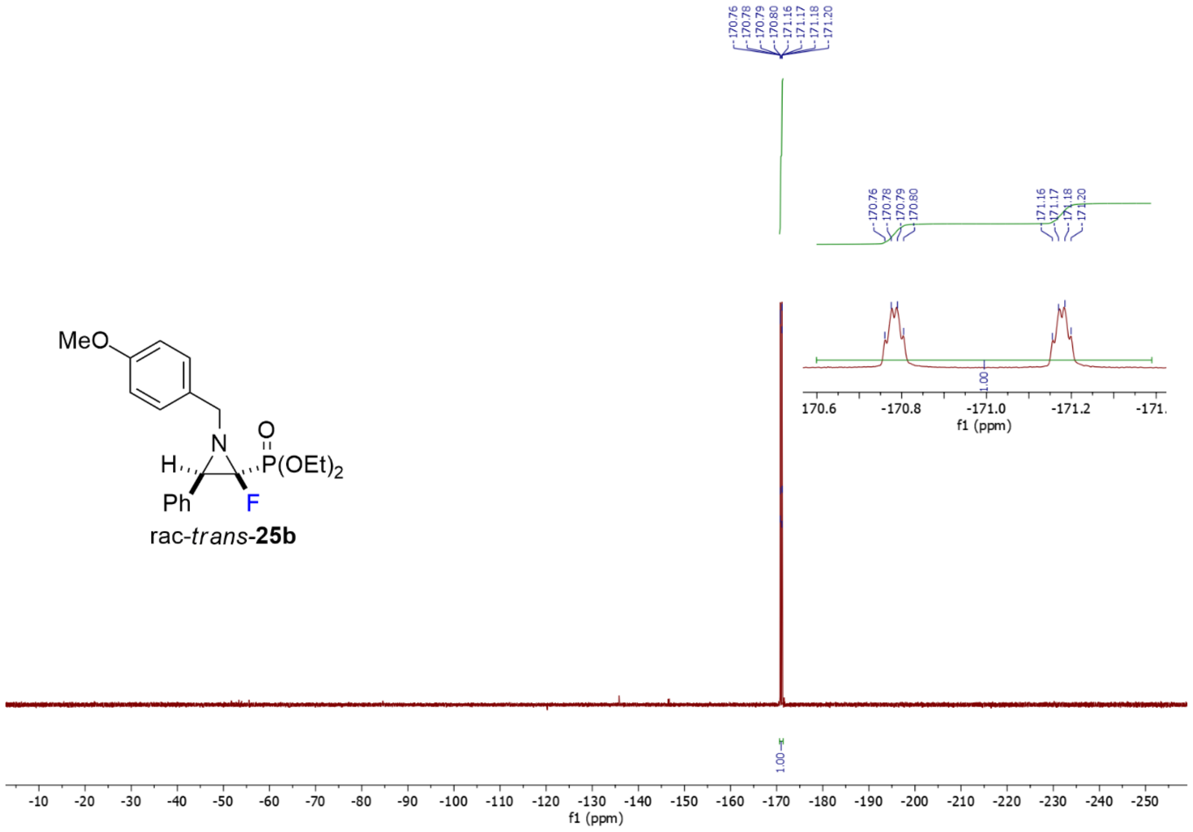

^{19}F NMR of rac-*trans*-**25b**

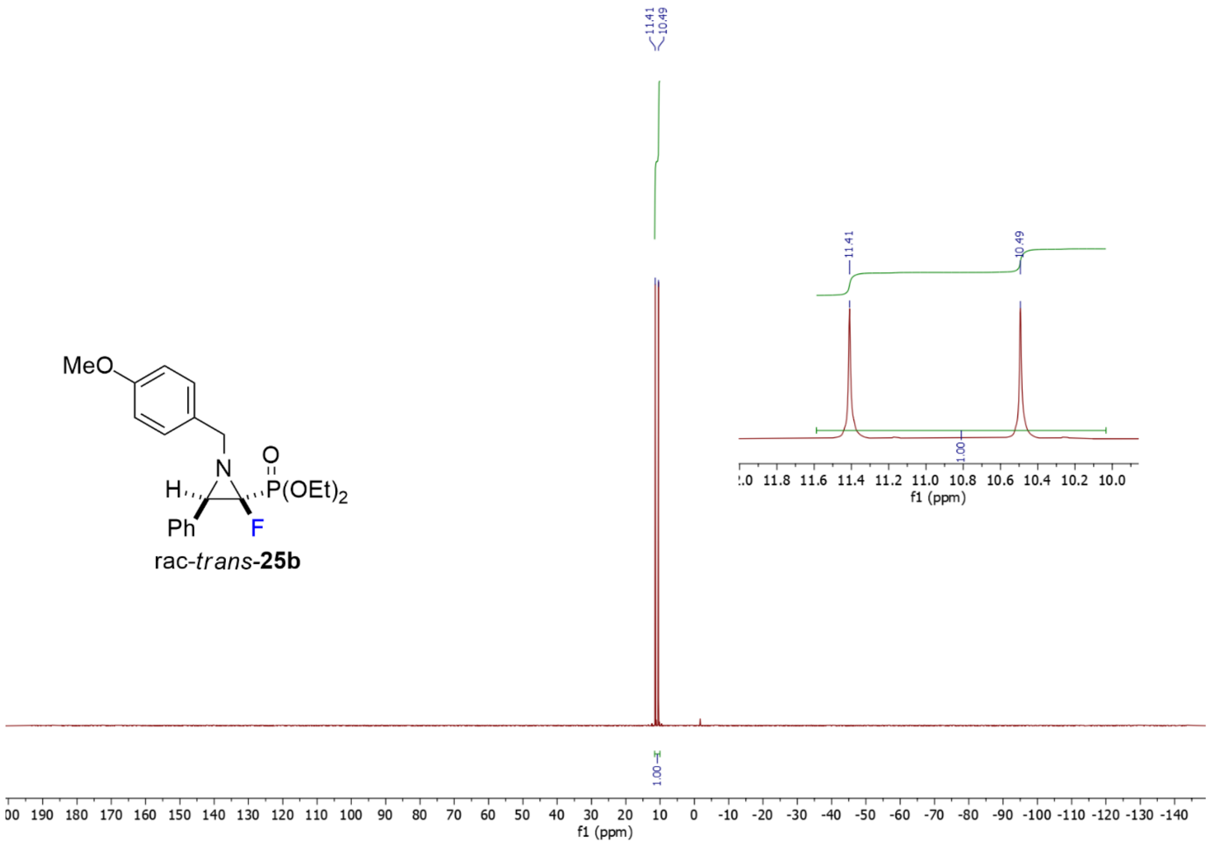

^{31}P{/^{1}H} NMR of rac-*trans*-**25b**

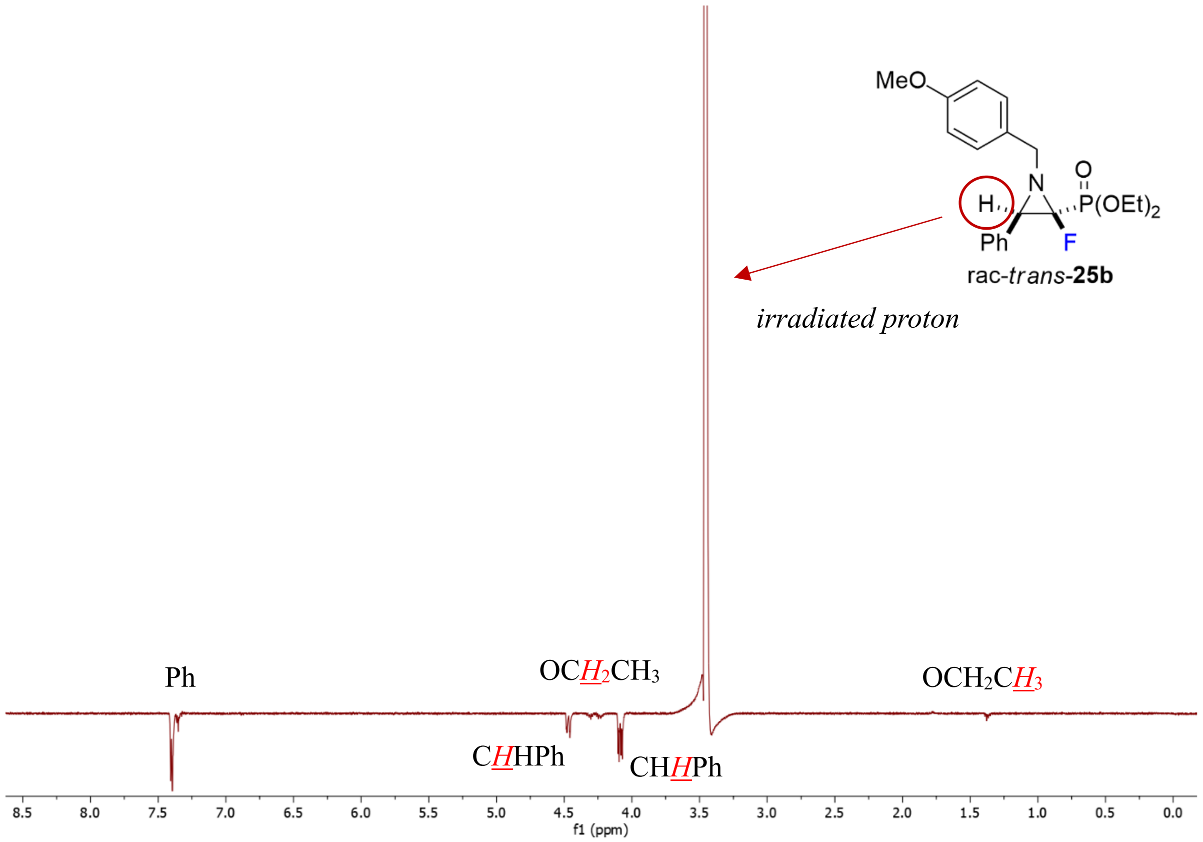

1D ^{1}H-^{1}H NOE NMR of rac-*trans*-**25b**

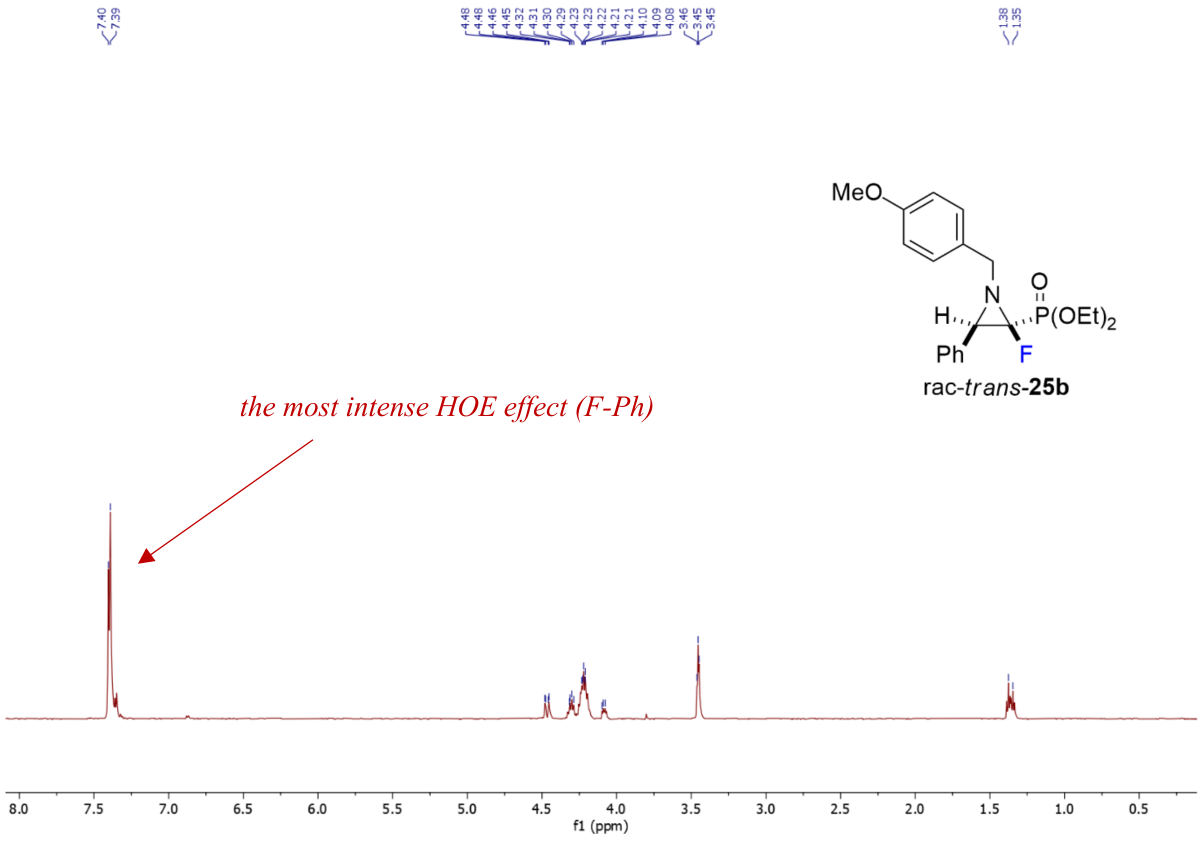

1D ^{1}H-^{19}F NOE NMR of rac-*trans*-**25b**

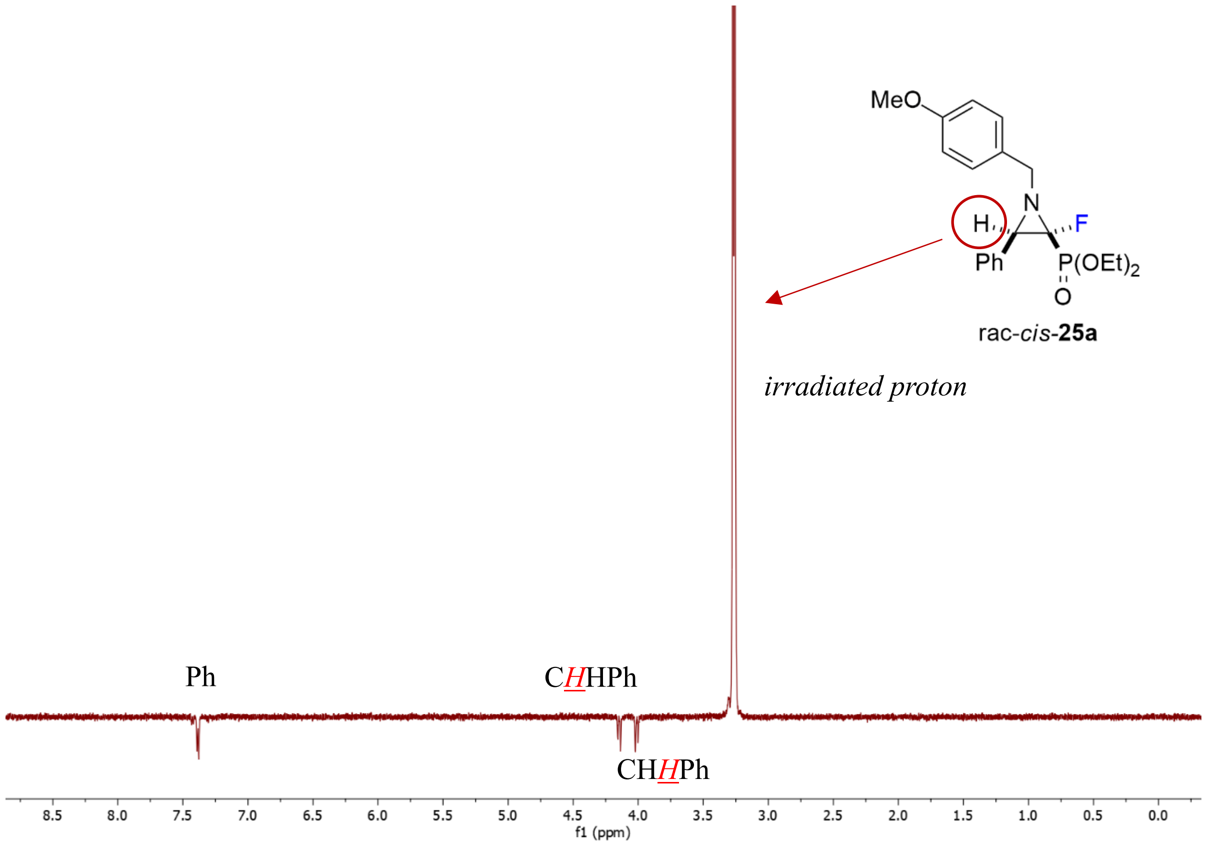

1D ^{1}H-^{1}H NOE NMR of rac-*cis*-**25a**

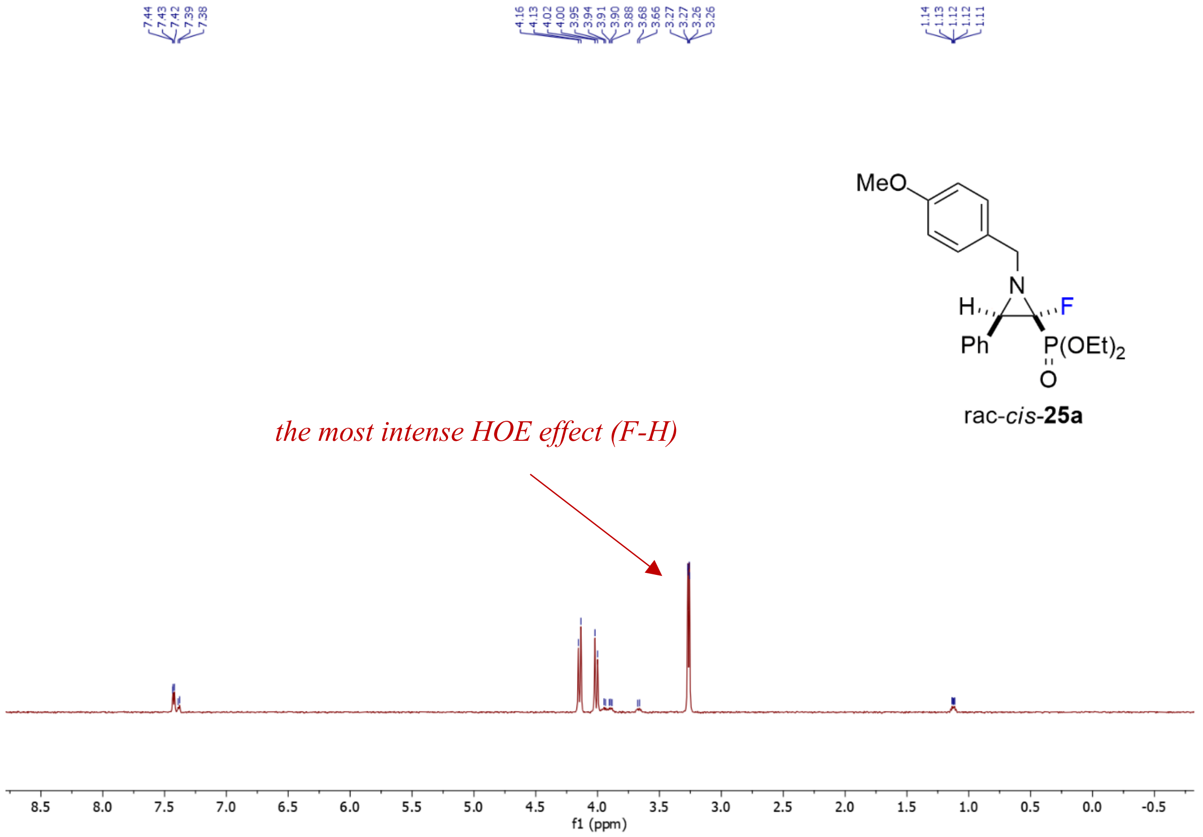

1D ^{1}H-^{19}F NOE NMR of rac-*cis*-**25a**

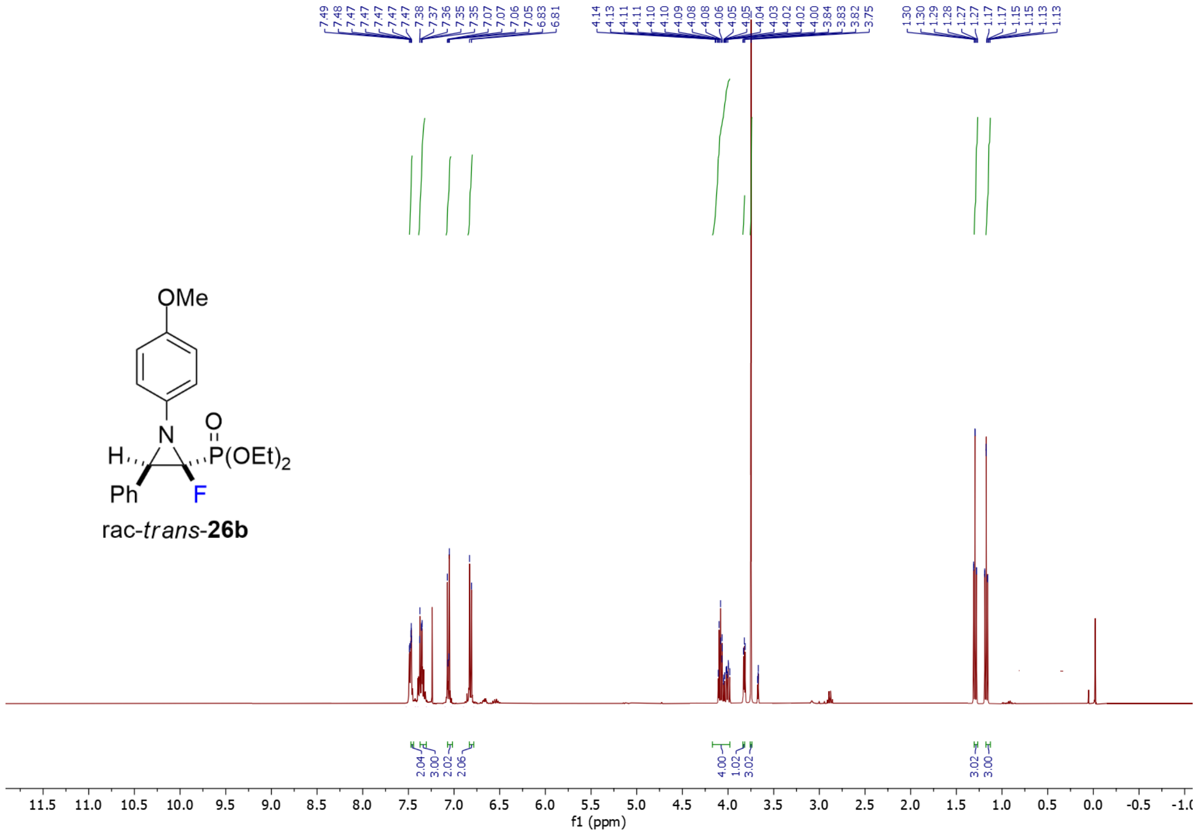

^{1}H NMR of rac-*trans*-**26b**

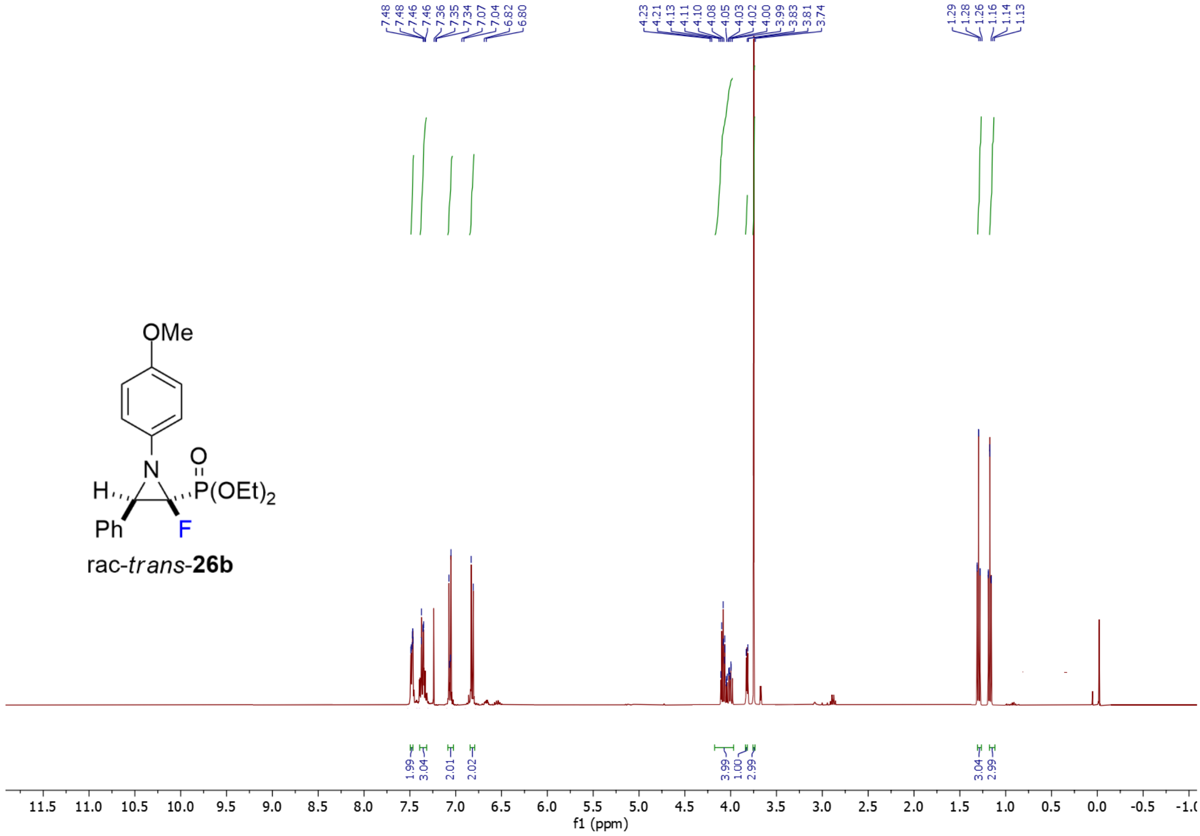

$^{1}H\{/^{31}P\}$ NMR of rac-*trans*-**26b**

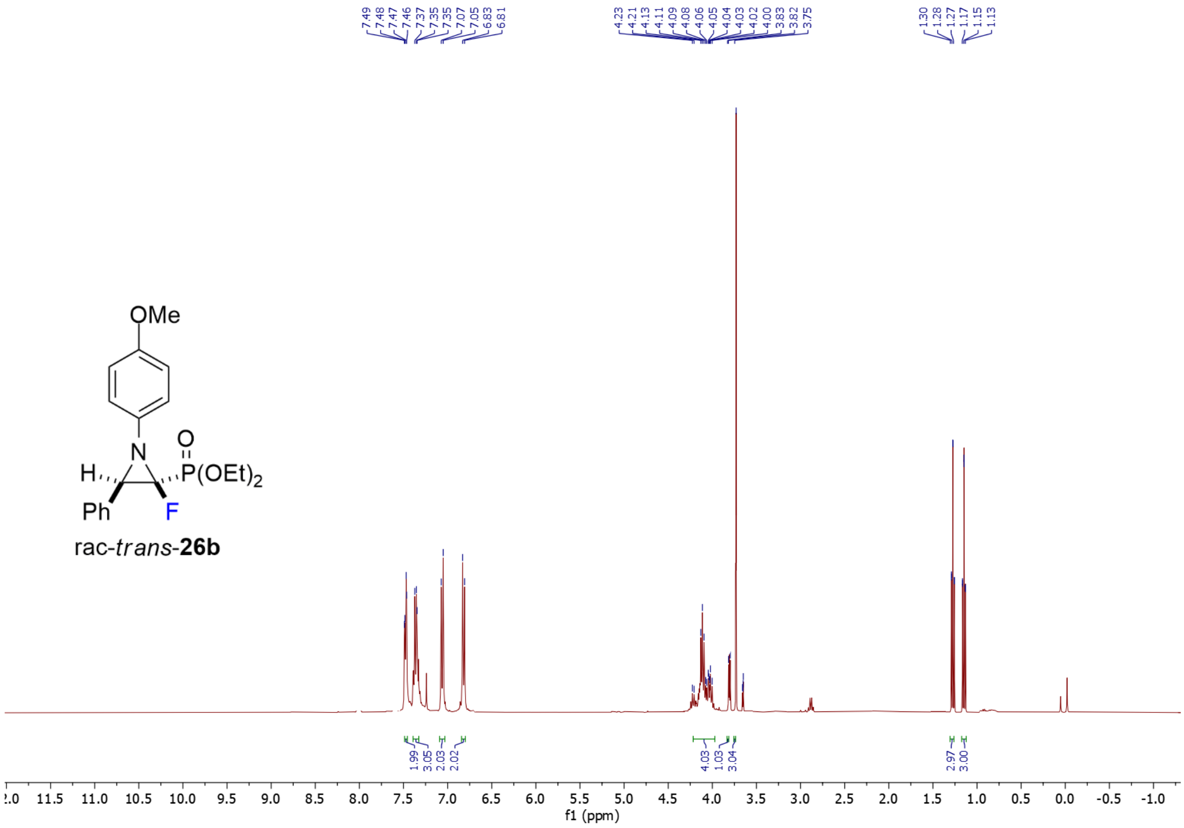

$^1H\{/^{19}F\}$ NMR of rac-*trans*-**26b**

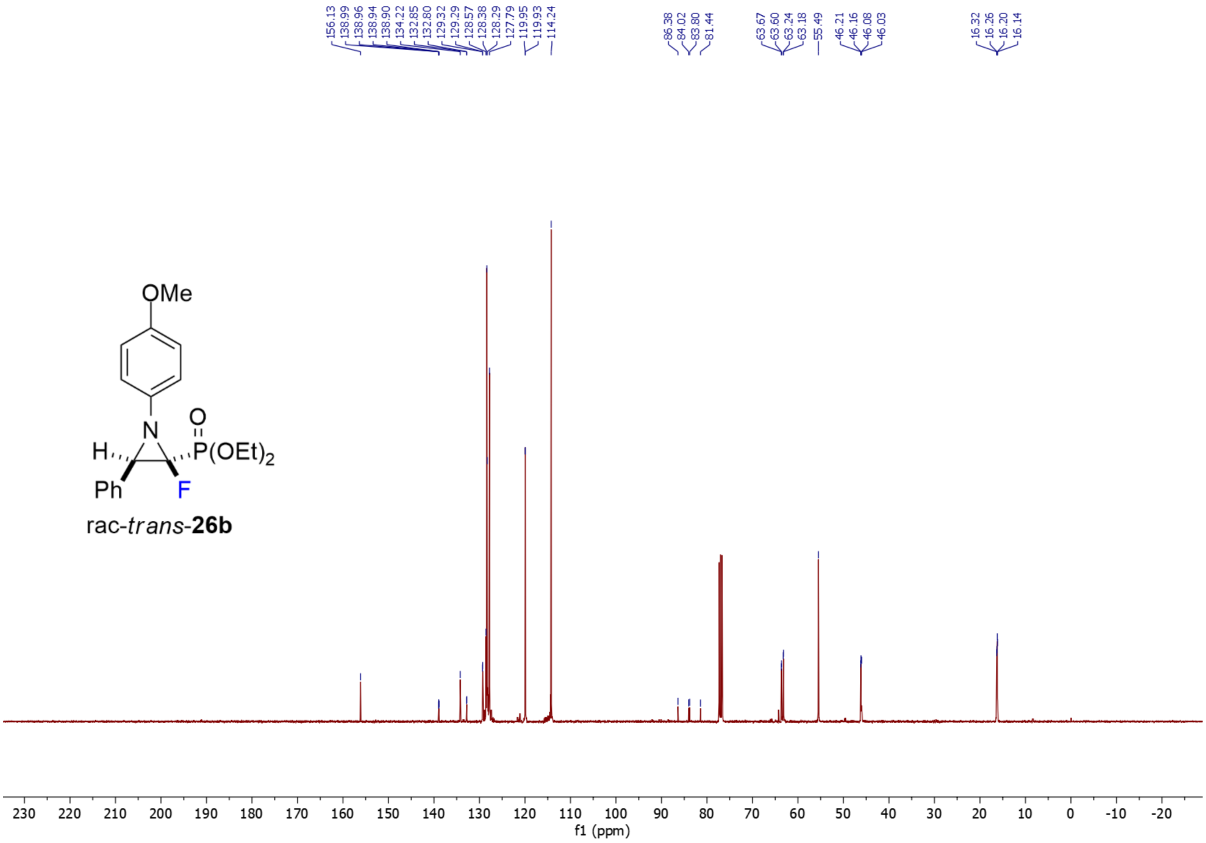

^{13}C NMR of rac-*trans*-**26b**

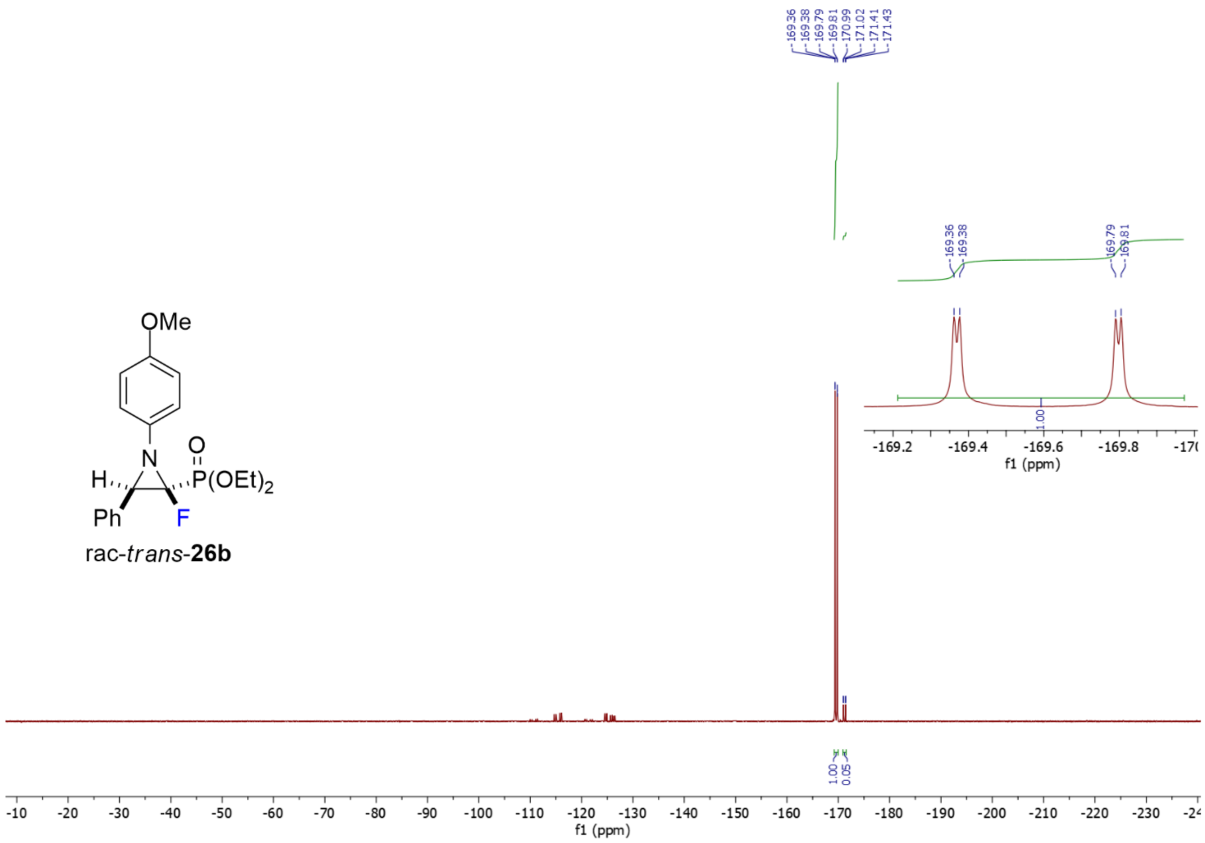

^{19}F NMR of rac-*trans*-**26b**

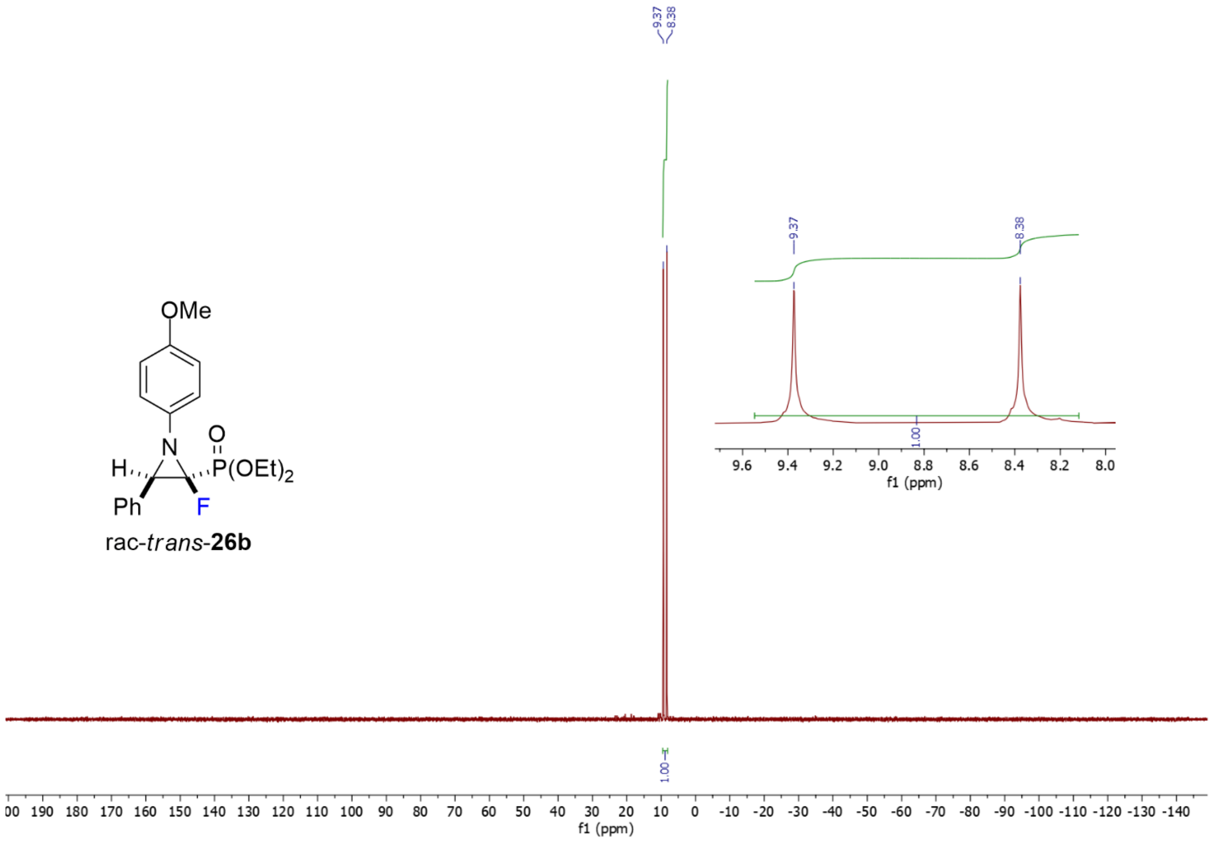

^{31}P{/^{1}H} NMR of rac-*trans*-**26b**

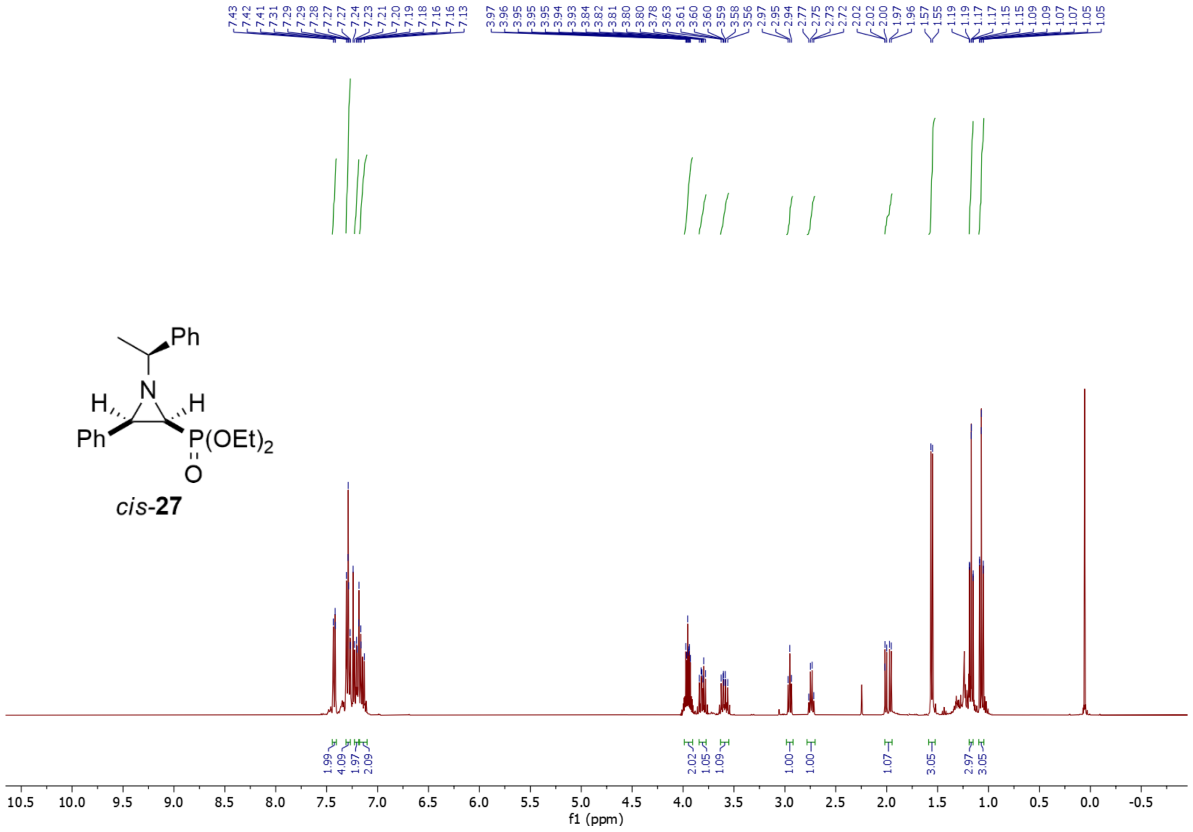

^{1}H NMR of *cis*-**27**

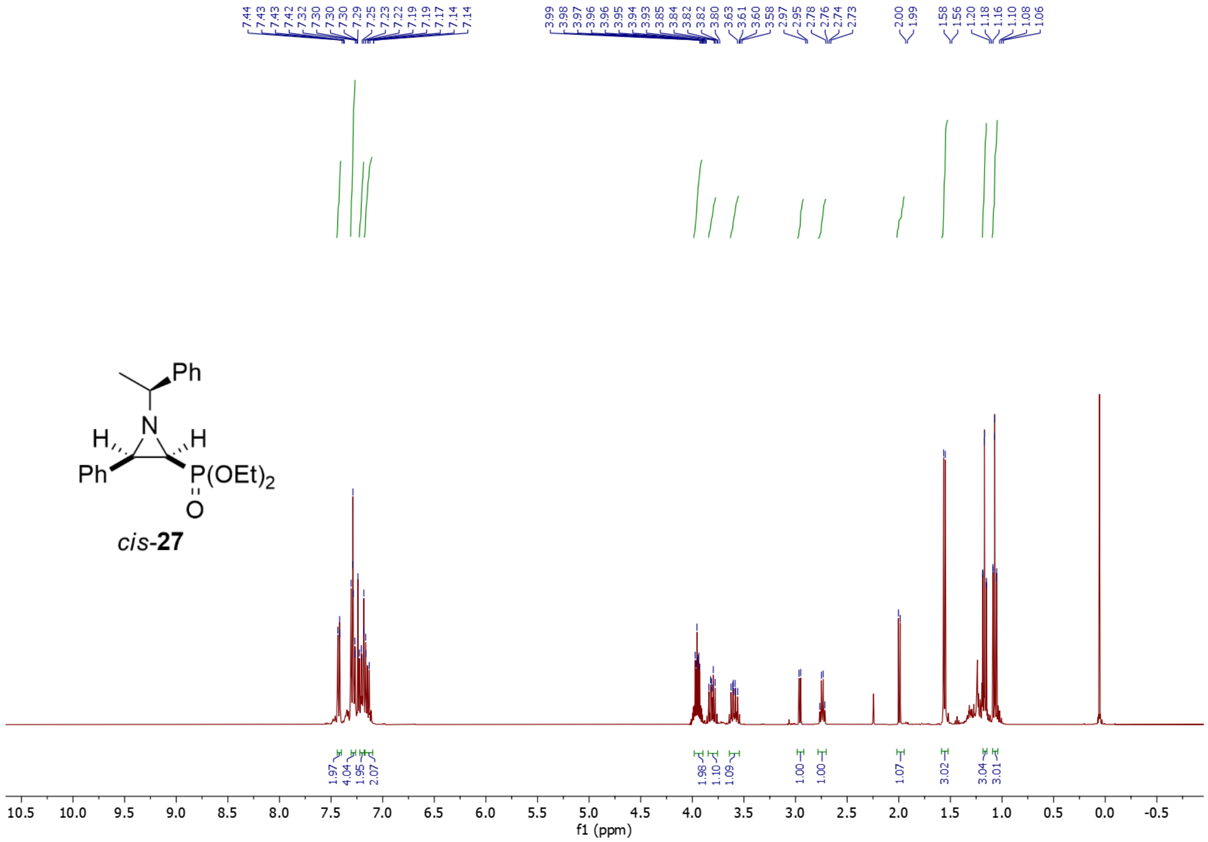

^{1}H{/^{31}P} NMR of *cis*-**27**

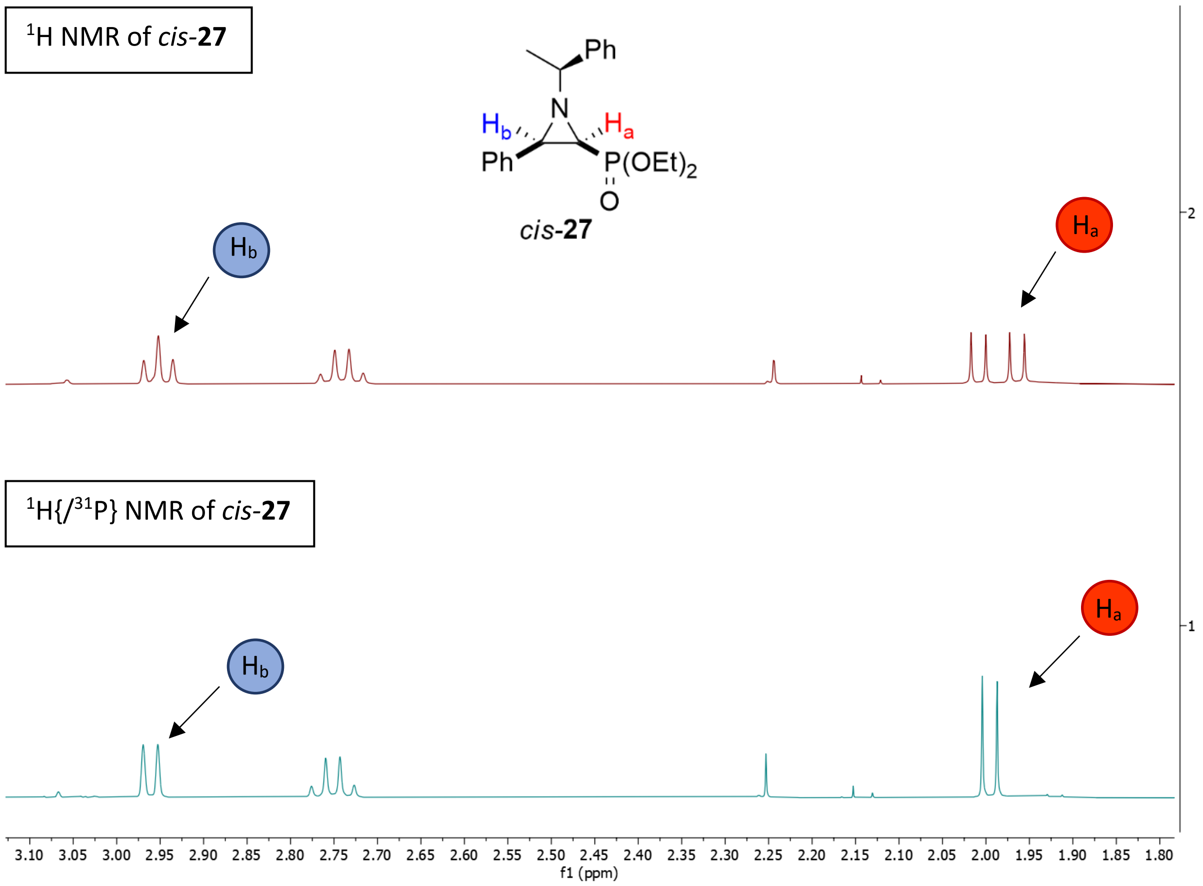

Aziridine protons decoupling of *cis*-**27**

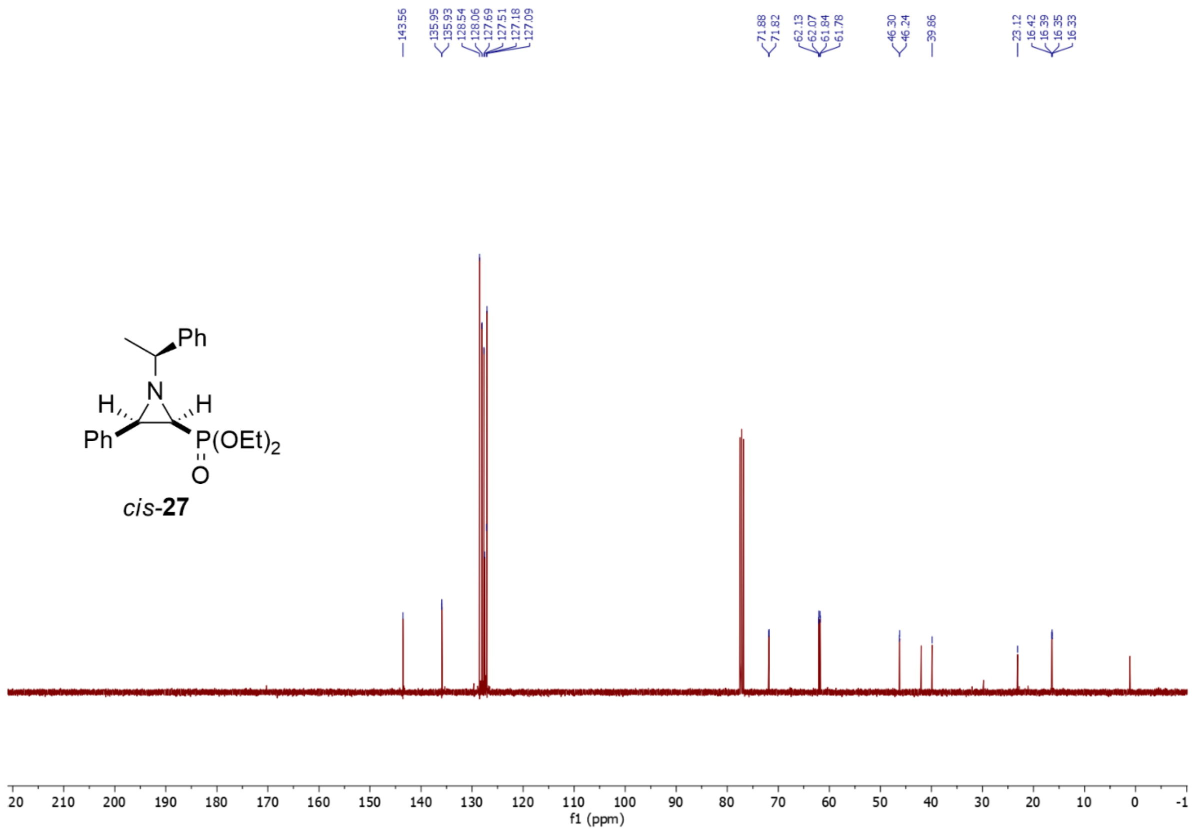

^{13}C NMR of *cis*-**27**

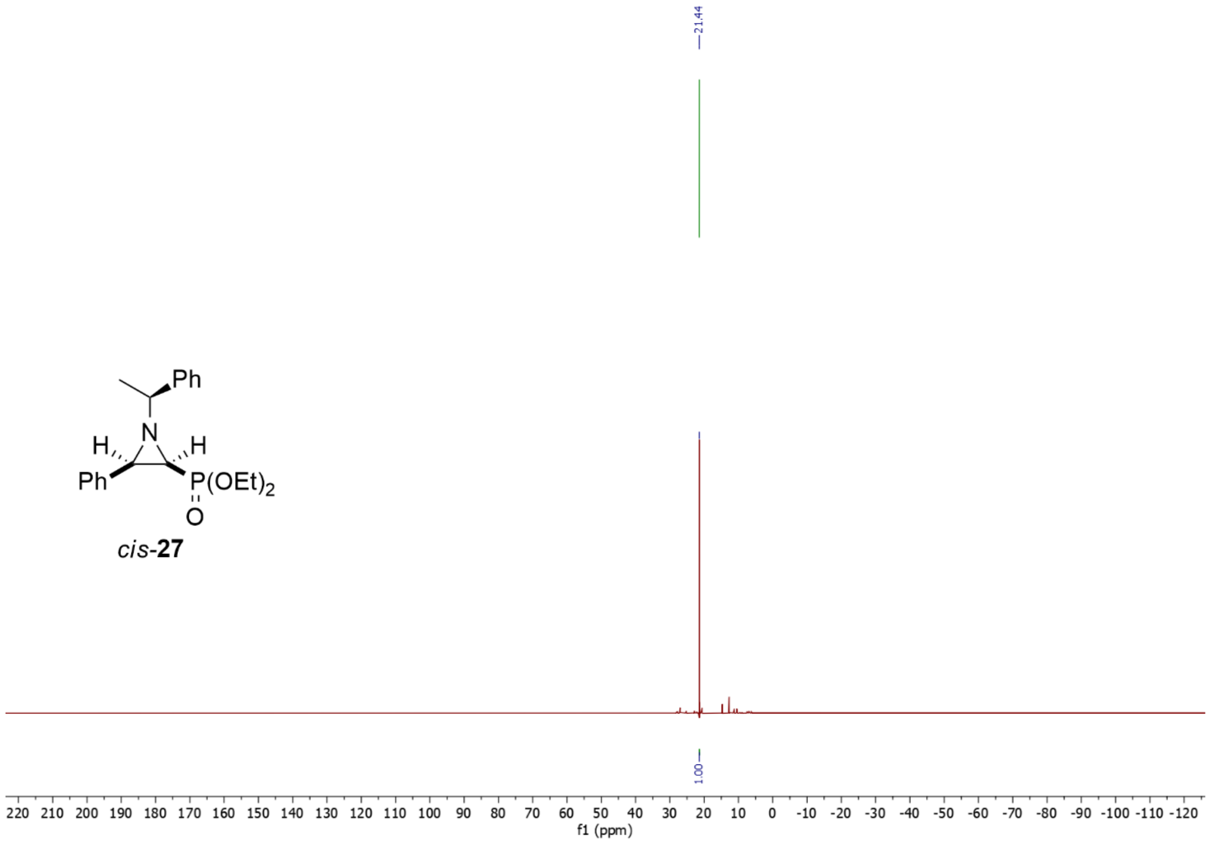

^{31}P{/^{1}H} NMR of *cis*-**27**

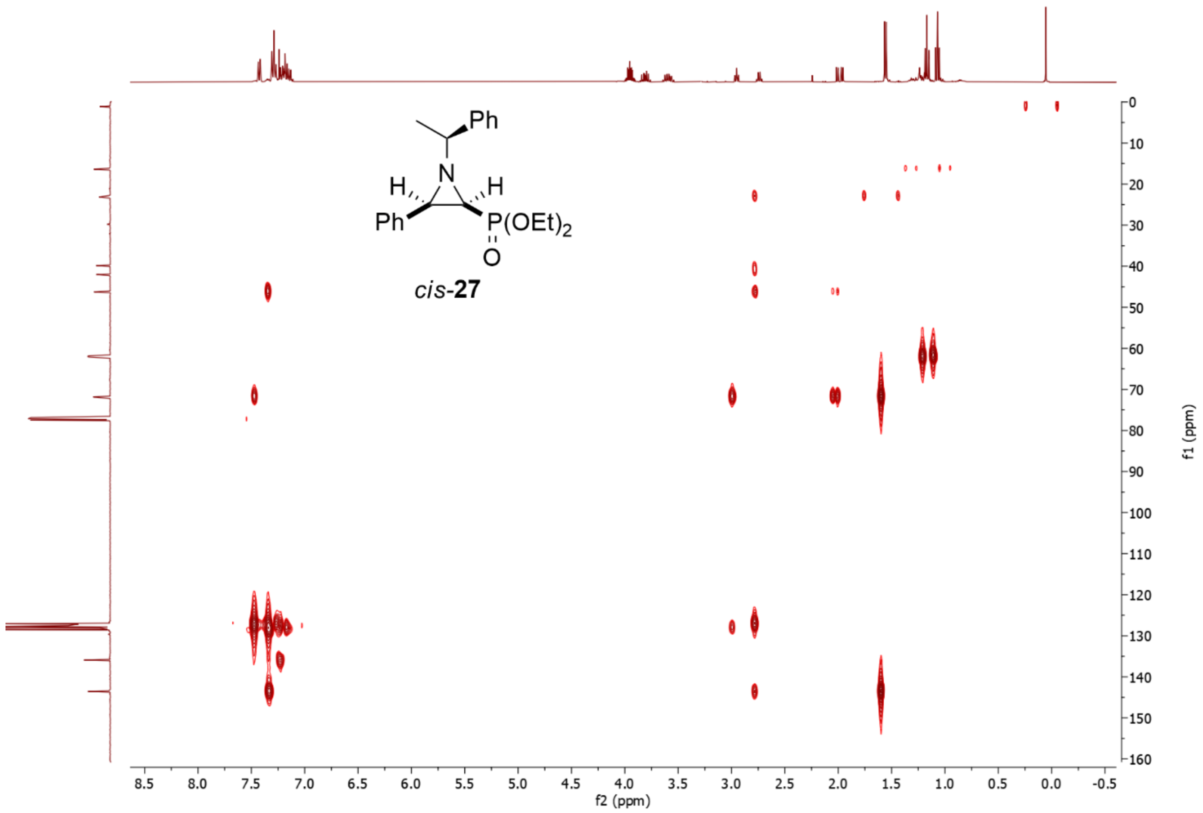

^{1}H-^{13}C HMBC NMR of *cis*-**27**

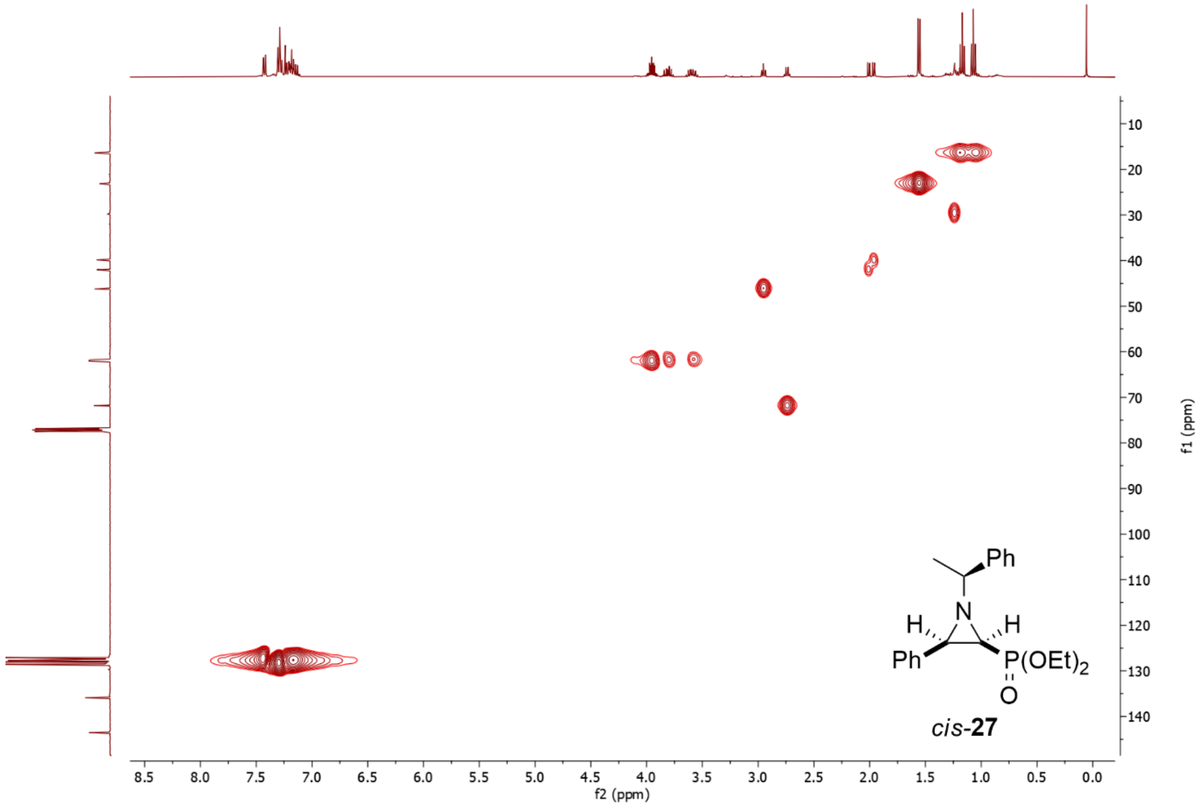

^{1}H-^{13}C HSQC NMR of *cis*-**27**

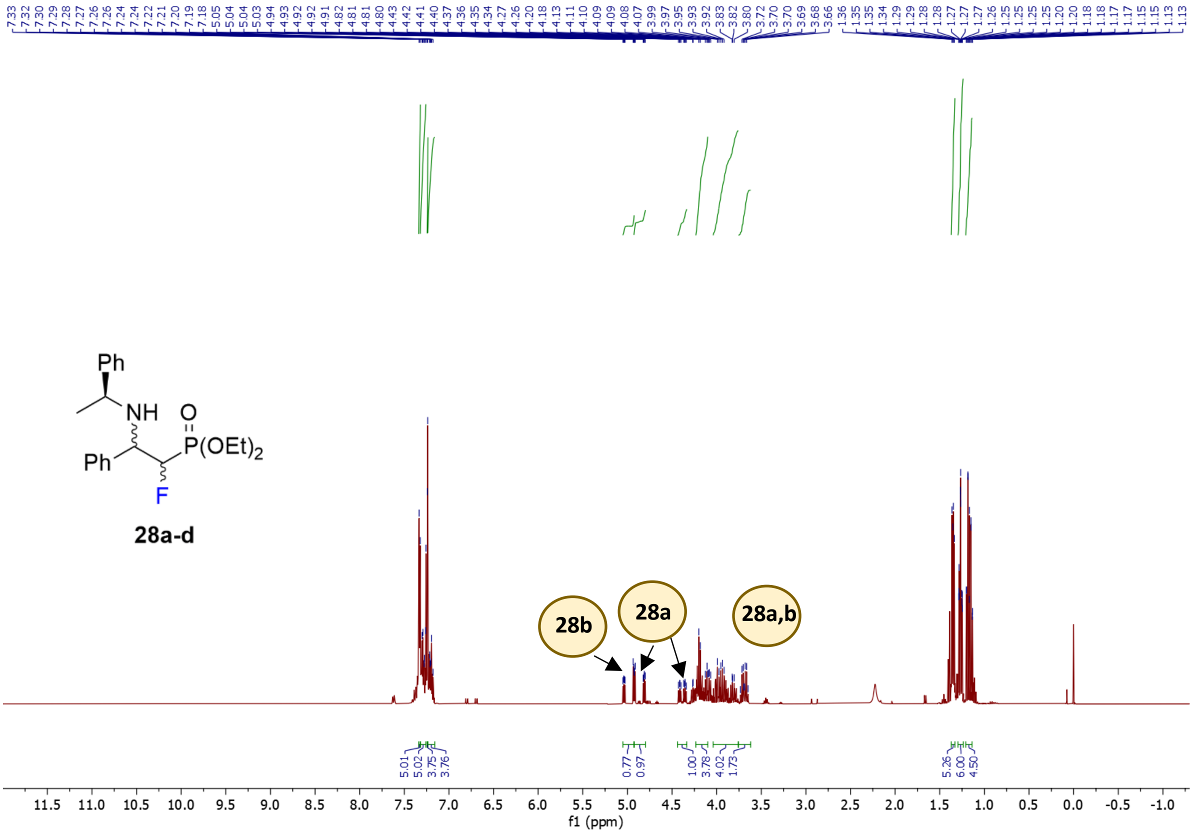

^{1}H NMR of **28a-d** *dr* 1:0.75:0.09:0.05

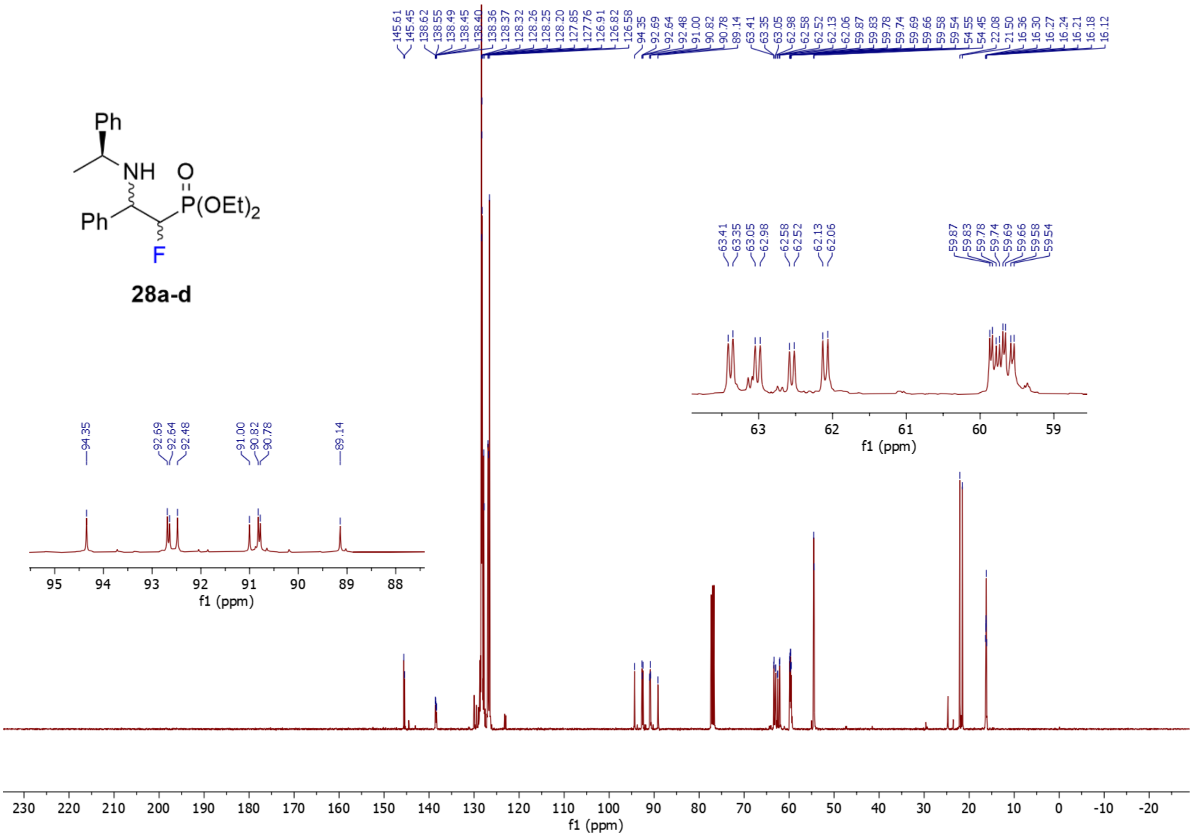

^{13}C NMR of **28a-d** *dr* 1:0.75:0.09:0.05

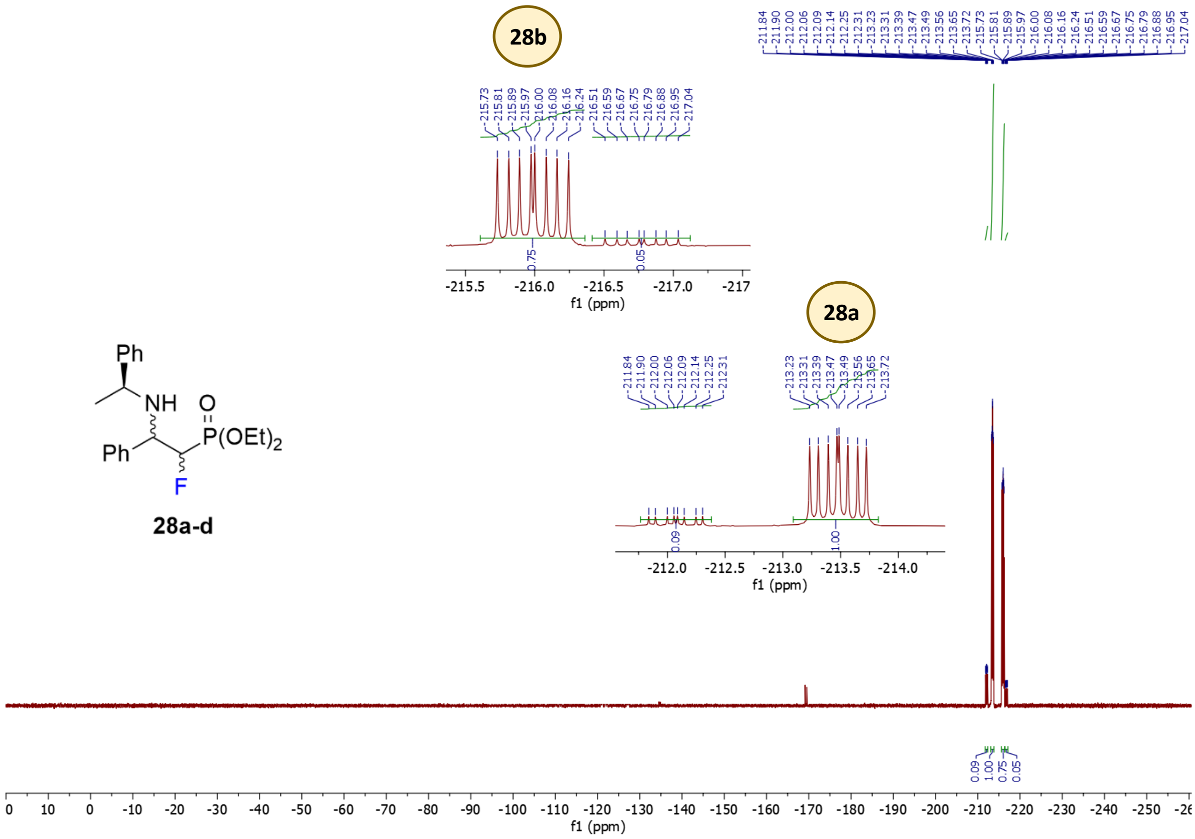

^{19}F NMR of **28a-d** *dr* 1:0.75:0.09:0.05

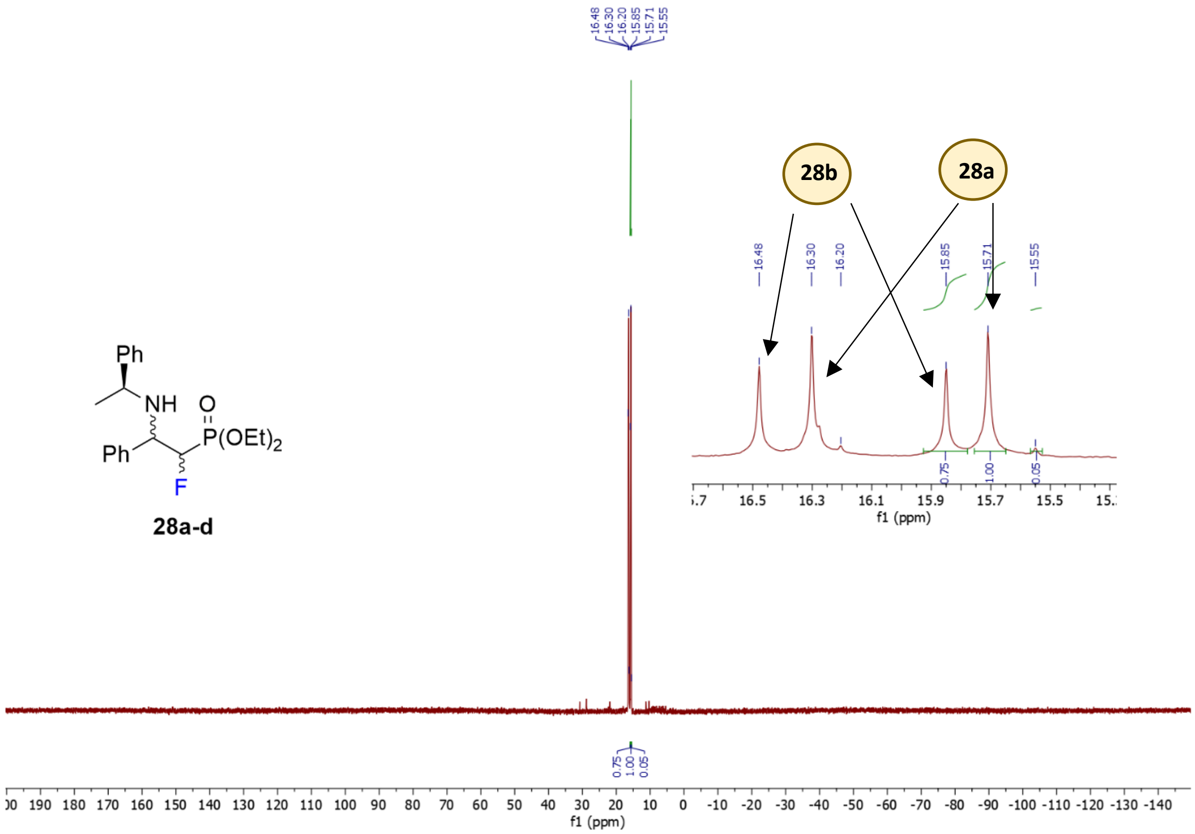

^{31}P NMR of **28a-d** *dr* 1:0.75:0.09:0.05

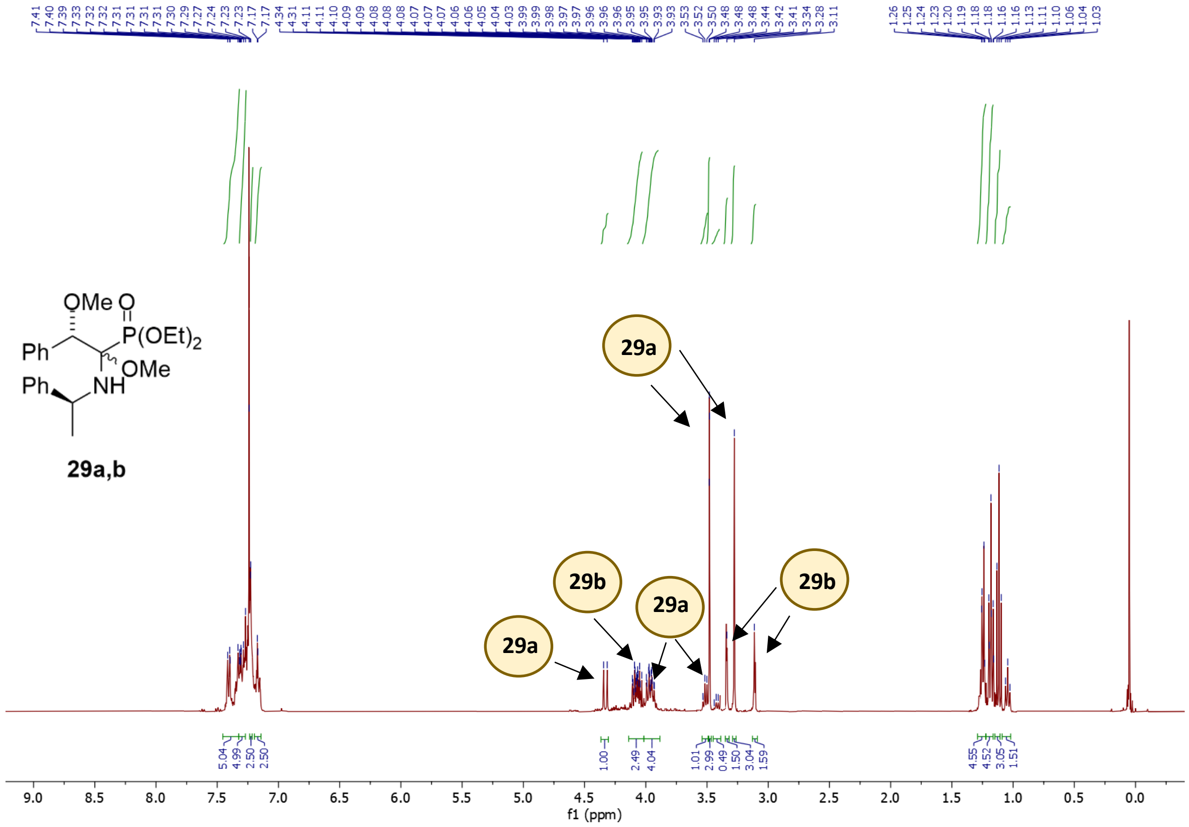

^{1}H NMR of **29a,b** *dr* 1:0.5

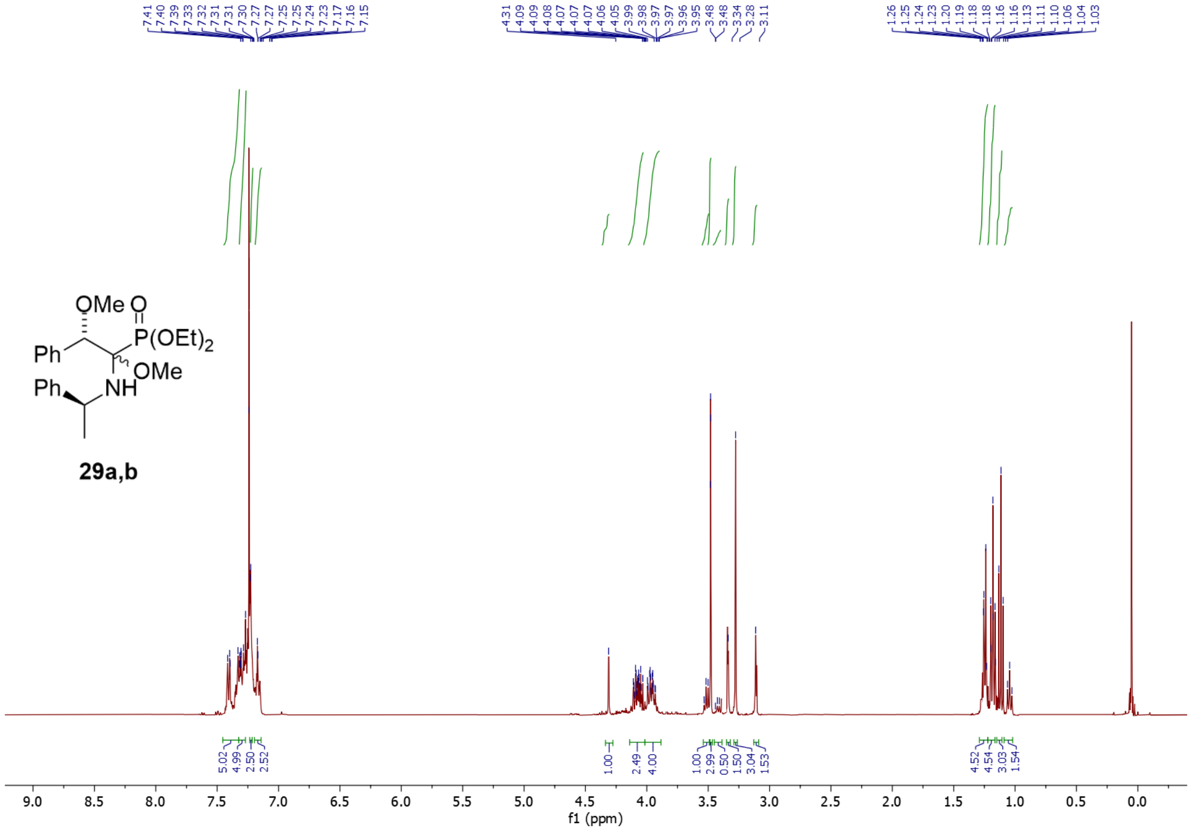

^{1}H {/^{31}P} NMR of **29a,b** *dr* 1:0.5

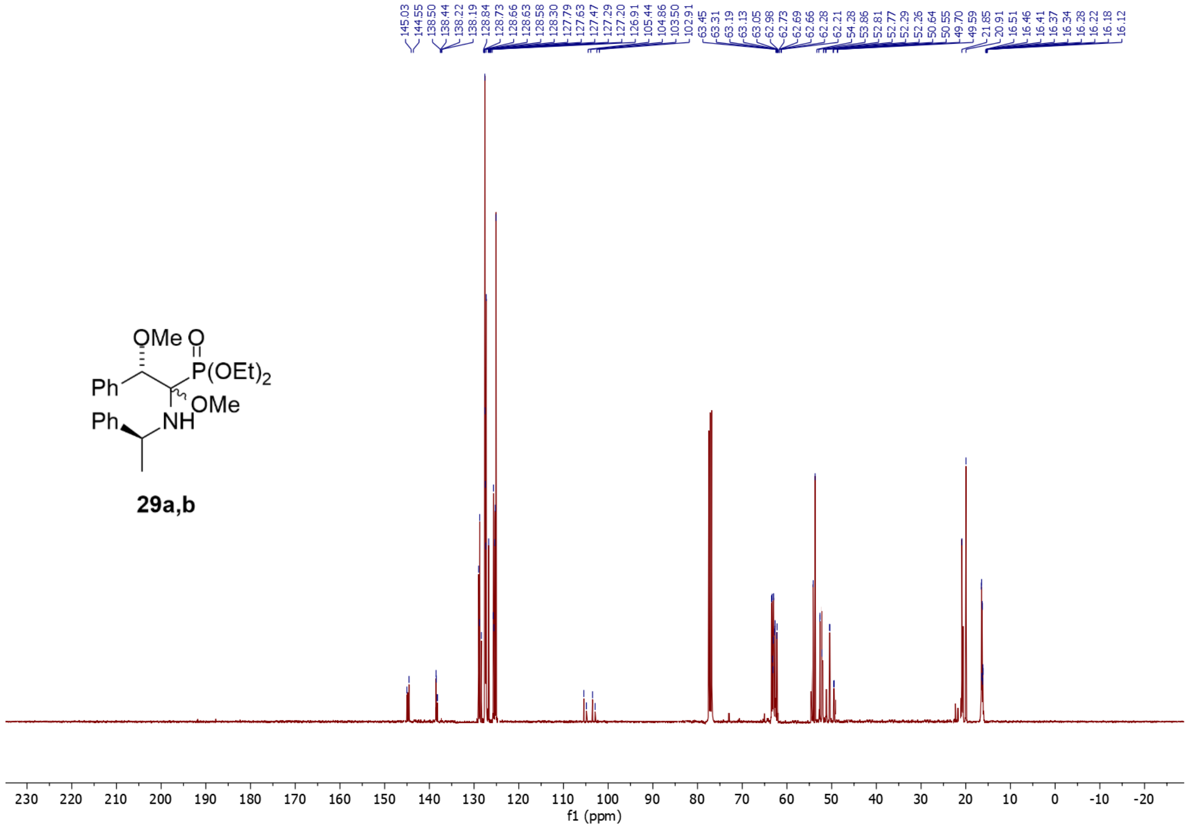

^{13}C NMR of **29a,b** *dr* 1:0.5

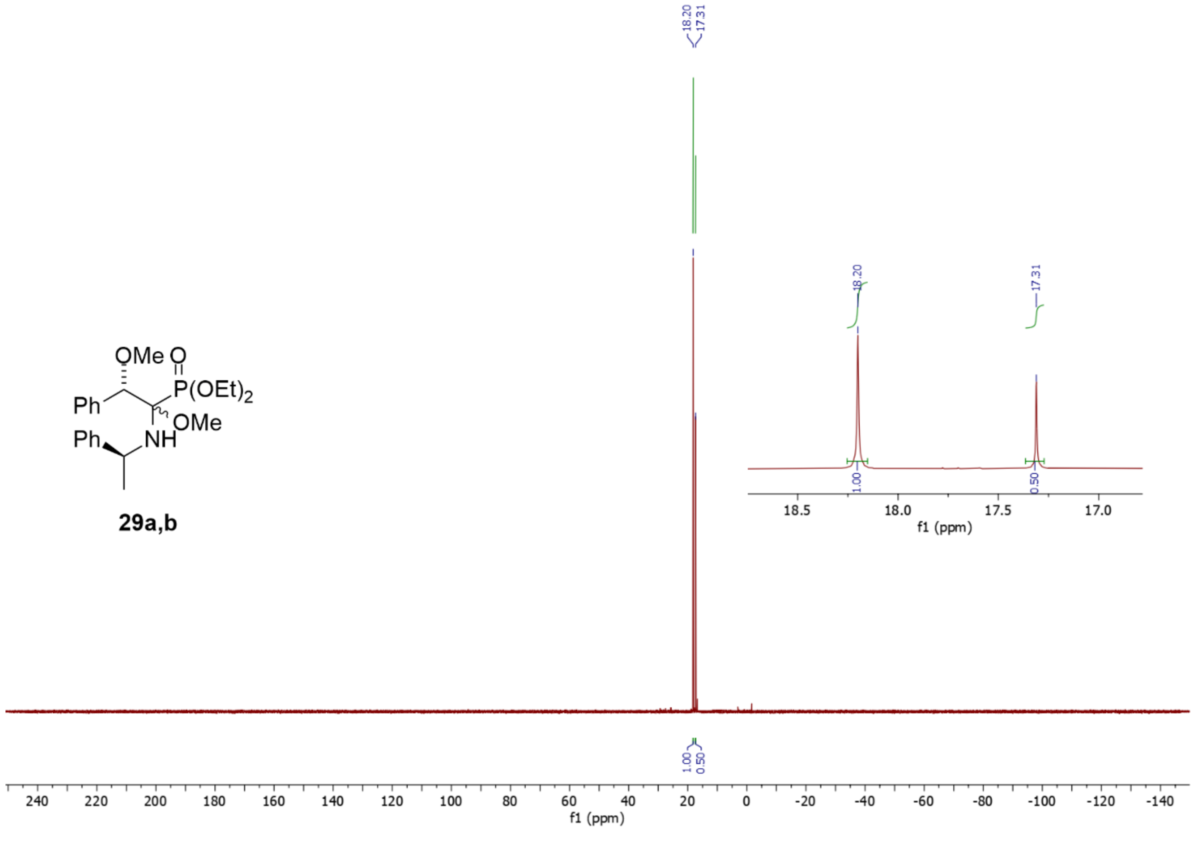

^{31}P{/^{1}H} NMR of **29a,b** *dr* 1:0.5

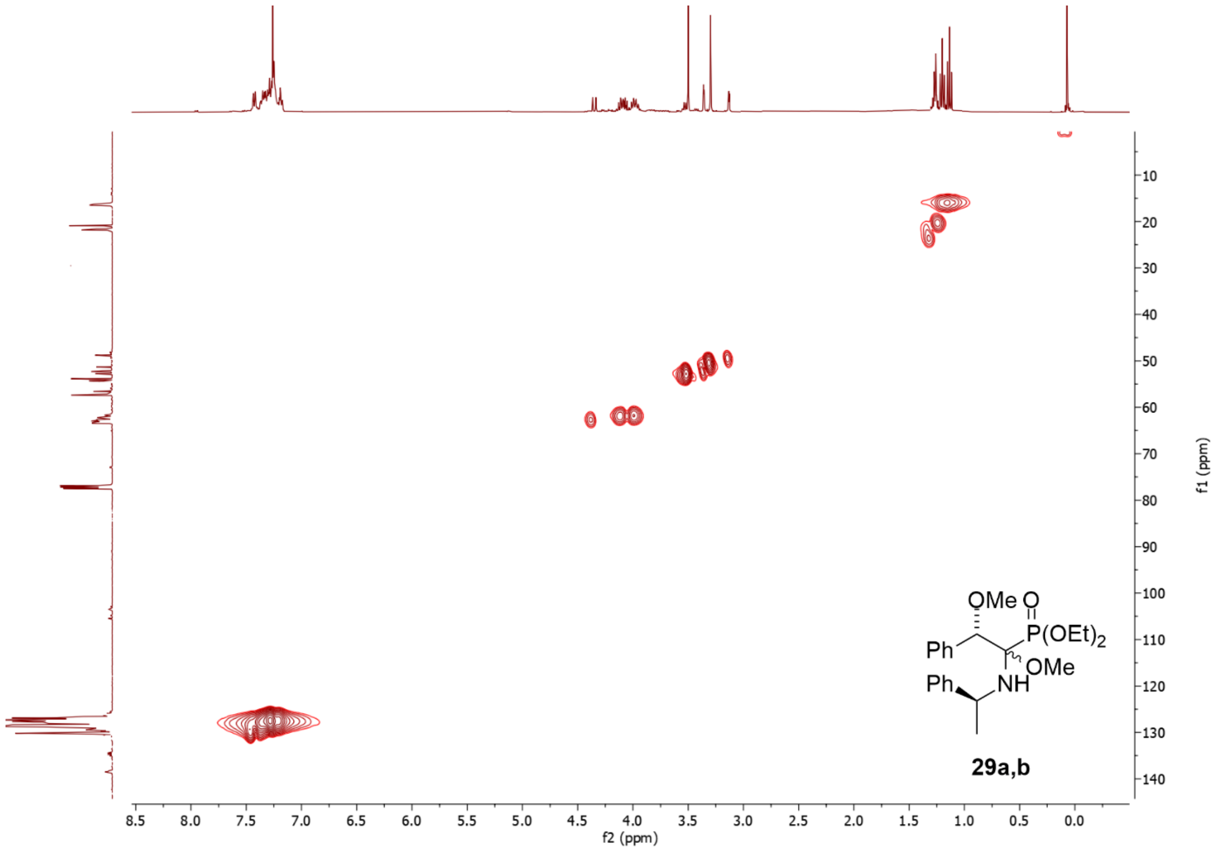

^{1}H-^{13}C HSQC NMR of **29a,b** *dr* 1:0.5

	Comp.	^{19}F NMR*	^{31}P NMR*	C=N/ CH(Ph)CF (^{13}C NMR)*	CFP (^{13}C NMR)*	CHPhCF (^{1}H NMR)*
Imines	**10a**	-129.08 (d, *J* 82.2)	7.37 (d, *J* 82.1)	164.44 (dd, *J* 27.0, 6.0)	100.05 (dd, *J* 269.8, 187.5)	-
	**10b**	-128.65 (d, *J* 82.3)	7.93 (d, *J* 82.3)	164.64 (dd, *J* 28.3, 5.9)	99.78 (dd, *J* 269.3, 187.2)	-
	**11a**	-128.52 (d, *J* 82.1)	7.38 (d, *J* 82.1)	164.45 (dd, *J* 27.1, 6.1)	100.99 (dd, *J* 269.7, 187.4)	-
	**11b**	-128.13 (d, *J* 82.3)	7.42 (d, *J* 82.4)	164.63 (dd, *J* 28.4, 5.9)	99.14 (dd, *J* 269.1, 187.0)	-
	**12**	-129.07 (d, *J* 81.2)	7.98 (d, *J* 81.4)	166.67 (dd, *J* 27.7, 6.1)	99.97 (dd, *J* 269.7, 187.6)	-
	**13**	-128.49 (d, *J* 81.7)	7.30 (d, *J* 81.5)	164.12 (dd, *J* 28.4, 5.9)	100.17 (dd, *J* 270.1, 187.2)	-
	**14a**	-125.59 (d, *J* 86.2)	8.02 (d, *J* 86.0)	163.55 (dd, *J* 28.1, 6.5)	105.89 (dd, *J* 260.7, 189.9)	-
	**14b**	-125.39 (d, *J* 87.0)	7.54 (d, *J* 87.0)	163.75 (dd, *J* 28.6, 6.8)	105.77 (dd, *J* 259.9, 193.0)	-
	**15**	-125.57 (d, *J* 85.6)	8.09 (d, *J* 86.0)	165.78 (dd, *J* 27.9, 6.7)	105.94 (dd, *J* 260.5, 193.5)	-
Amines	**16a**	-125.06 (dd, *J* 82.3, 10.2)	9.77 (d, *J* 82.0)	64.12 (dd, *J* 22.0, 7.5)	107.44 (dd, *J* 270.1, 184.3)	4.58 (dd, *J* 10.2, 4.7)
	**16b**	-135.38 (dd, *J* 84.6, 22.1)	9.25 (d, *J* 84.7)	66.47 (dd, *J* 18.2, 7.9)	107.62 (dd, *J* 274.2, 183.1)	4.37 (dd, *J* 22.0, 3.5)
	**16c**	-128.26 (dd, *J* 78.0, 10.2)	9.06 (d, *J* 77.9)	only diagnostic signals (^{19}F, ^{31}P NMR)		
	**16d**	-135.49 (dd, *J* 88.4, 20.9)	*masked*			
	**17a**	-125.04 (dd, *J* 82.1, 10.1)	9.25 (d, *J* 82.0)	64.05 (dd, *J* 21.9, 7.5)	107.39 (dd, *J* 270.1, 184.4)	4.58 (dd, *J* 10.2, 4.7)
	**17b**	-135.36 (dd, *J* 84.6, 22.0)	8.73 (d, *J* 84.6)	66.42 (dd, *J* 18.2, 8.0)	107.57 (dd, *J* 274.0, 182.8)	4.34 (dd, *J* 22.1, 3.2)
	**17c**	-128.25 (dd, *J* 77.9, 10.2)	8.56 (d, *J* 78.0)	only diagnostic signals (^{19}F, ^{31}P NMR)		
	**17d**	*masked*	8.40 (d, *J* 86.9)			
	**18a**	-126.81 (dd, *J* 79.5, 9.4)	9.52 (d, *J* 79.5)	65.24 (dd, *J* 21.5, 7.9)	106.46 (dd, *J* 270.2, 183.8)	*masked*
	**18b**	-135.34 (dd, *J* 85.7, 20.6)	8.93 (d, *J* 85.7)	67.72 (dd, *J* 18.4, 8.2)	106.64 (dd, *J* 272.9, 184.4)	*masked*
	**19a**	-126.36 (dd, *J* 78.1, 9.7)	9.22 (d, *J* 77.9)	63.30 (dd, *J* 23.4, 6.9)	105.61 (dd, *J* 271.6, 183.6)	*masked*
	**19b**	-135.34 (dd, *J* 82.1, 18.6)	8.17 (d, *J* 82.1)	66.49 (dd, *J* 19.7, 7.6)	105.37 (dd, *J* 274.0, 181.3)	*masked*
	**20a**	-123.06 (dd, *J* 87.4, 8.8)	9.61 (d, *J* 87.4)	63.80 (dd, *J* 23.3, 8.1)	110.45 (dd, *J* 260.4, 191.5)	4.71 (dd, *J* 8.9, 4.6)
	**20b**	-134.74 (dd, *J* 89.0, 21.5)	9.02 (d, *J* 89.2)	65.83 (dd, *J* 18.6, 8.7)	111.08 (dd, *J* 265.2, 190.1)	4.41 (dd, *J* 21.3, 3.3)
	**20c**	-125.91 (dd, *J* 83.2, 9.2)	8.89 (d, *J* 83.3)	only diagnostic signals (^{19}F, ^{31}P NMR)		
	**20d**	-134.88 (dd, *J* 91.3, 21.0)	8.66 (d, *J* 91.5)			
	**21a**	-124.54 (dd, *J* 84.8, 8.3)	9.37 (d, *J* 84.7)	65.07 (dd, *J* 22.1, 8.6)	109.70 (dd, *J* 260.8, 191.2)	4.45 (dd, *J* 8.4, 4.4)

	**21b**	-134.91 (dd, *J* 90.6, 20.5)	8.76 (d, *J* 90.8)	67.24 (dd, *J* 18.8, 8.9)	110.35 (dd, *J* 264.0, 191.1)	*masked*
Aziridines	*cis*-**24a**	-182.45 (dd, *J* 118.5, 8.7)	9.64 (d, *J* 118.8)	48.96 (dd, *J* 19.1, 1.6)	87.05 (dd, *J* 274.2, 272.1)	3.18 (d, *J* 8.7)
	trans-**24b**	-168.76 (dt, *J* 113.8, 4.8)	10.83 (d, *J* 114.0)	47.46 (dd, *J* 12.7, 5.8)	84.27 (dd, *J* 258.7, 233.2)	3.26 (t, *J* 4.2)
	cis-**24c**	-178.36 (dd, *J* 114.2, 8.5)	8.94 (d, *J* 114.1)	only diagnostic signals (^{19}F, ^{31}P NMR)		
	trans-**24d**	-169.11 (dt, *J* 110.8, 4.7)	10.22 (d, *J* 110.6)			
	cis-**25a**	-180.27 (dd, *J* 116.7, 8.4)	9.97 (d, *J* 116.9)	49.08 (dd, *J* 19.2, 1.3)	86.67 (dd, *J* 274.0, 272.5)	3.26 (d, *J* 8.5)
	trans-**25b**	-170.98 (dq, *J* 111.7, 4.3)	10.95 (d, *J* 111.6)	48.43 (dd, *J* 12.9, 6.0)	83.95 (dd, *J* 259.6, 231.6)	3.45 (t, *J* 4.1)
	cis-**26a**	-171.22 (dd, *J* 117.5, 7.9)	8.63 (d, *J* 117.4)	only diagnostic signals (^{19}F, ^{31}P NMR)		
	trans-**26b**	-169.58 (dd, *J* 121.4, 4.3)	8.82 (d, *J* 121.5)	46.12 (dd, *J* 13.1, 5.4)	83.91 (dd, *J* 260.8, 238.3)	3.84 (t, *J* 4.1)

*δ [ppm], *J* [Hz]

The geometries of reported structures optimized at the PCM/ωb97x-d/def-TZVP(D) level of theory.

TS$_{C1}$ (solvent=n,n-DiMethylFormamide, Figures 8 and 9 in the manuscript):

6	-2.019045000	0.410186000	-0.108645000
6	-3.200516000	1.121259000	-0.305764000
6	-4.327410000	0.838393000	0.447347000
6	-4.286102000	-0.166266000	1.404224000
6	-3.114435000	-0.880449000	1.598958000
6	-1.983956000	-0.594170000	0.847054000
1	-1.084814000	-1.169784000	1.004211000
6	-0.852160000	0.821076000	-0.965256000
1	-3.239899000	1.903080000	-1.055474000
1	-5.238572000	1.398903000	0.282929000
1	-5.165603000	-0.392683000	1.993312000
1	-3.074354000	-1.667540000	2.340952000
7	-0.265251000	2.112853000	-0.650123000
1	-1.115077000	0.774096000	-2.021452000
6	0.543996000	0.346973000	-0.805123000
15	1.477998000	0.430251000	0.807352000
9	1.286973000	0.507710000	-1.878011000
8	0.595307000	0.906832000	1.875833000
8	2.646351000	1.348511000	0.323330000
8	2.197990000	-0.925762000	1.089748000
6	3.807972000	1.596139000	1.135537000
6	1.687702000	-1.926997000	1.986421000
1	4.420149000	2.299227000	0.579379000
1	4.354157000	0.668119000	1.295456000
1	3.514019000	2.033062000	2.089109000
1	1.094585000	-1.463387000	2.772725000
1	2.552902000	-2.424967000	2.415008000
1	1.087115000	-2.632005000	1.415000000
6	-0.089769000	3.137713000	-1.657512000
1	-1.052592000	3.538931000	-1.982429000
1	0.425589000	2.720755000	-2.521883000
1	0.518121000	3.937982000	-1.242548000
35	0.286030000	-2.188826000	-1.317112000
1	-0.417580000	2.412209000	0.302577000

TS$_{C2}$ (solvent=n,n-DiMethylFormamide, Figures 8 and 9 in the manuscript):

6	2.108536000	-0.292335000	-0.257594000
6	2.017484000	0.091071000	1.073063000
6	3.166645000	0.200004000	1.842844000
6	4.410204000	-0.076623000	1.295591000
6	4.505666000	-0.461943000	-0.033849000
6	3.360061000	-0.564570000	-0.805405000
1	3.438826000	-0.859909000	-1.845402000

6	0.941491000	-0.485283000	-1.182803000
1	1.054301000	0.300494000	1.511844000
1	3.085729000	0.503883000	2.878692000
1	5.303001000	0.010392000	1.901658000
1	5.471380000	-0.676196000	-0.473025000
7	0.391727000	-1.834610000	-1.287573000
1	1.203184000	-0.110693000	-2.170626000
6	-0.480077000	-0.142698000	-0.948241000
15	-1.626322000	-0.543083000	0.464604000
9	-1.148816000	0.047608000	-2.069823000
8	-0.938127000	-0.664934000	1.749623000
8	-2.275754000	-1.838300000	-0.137244000
8	-2.798101000	0.484396000	0.384929000
6	-3.409275000	-2.456775000	0.495201000
6	-3.035272000	1.531881000	1.340391000
1	-3.636561000	-3.342057000	-0.090782000
1	-4.259724000	-1.776247000	0.488294000
1	-3.160783000	-2.742217000	1.516881000
1	-2.286067000	1.509604000	2.128886000
1	-4.025972000	1.366104000	1.757637000
1	-2.990892000	2.480148000	0.813200000
6	0.708322000	-2.866928000	-0.317292000
1	1.738697000	-3.208773000	-0.437533000
1	0.022085000	-3.697267000	-0.462894000
1	0.583316000	-2.482761000	0.693494000
35	-0.212821000	2.420999000	-0.465625000
1	0.233445000	-2.153522000	-2.230995000

TS_{T1} (solvent=n,n-DiMethylFormamide, Figures 8 and 9 in the manuscript):

6	-2.244579000	-0.591238000	0.190985000
6	-2.810732000	-0.991223000	-1.015207000
6	-4.154392000	-0.760388000	-1.266122000
6	-4.943452000	-0.130540000	-0.314973000
6	-4.384849000	0.265803000	0.891247000
6	-3.041739000	0.035263000	1.141943000
1	-2.606687000	0.349487000	2.082486000
6	-0.800363000	-0.833023000	0.513005000
1	-2.216643000	-1.481477000	-1.776368000
1	-4.584051000	-1.073751000	-2.208664000
1	-5.992481000	0.048337000	-0.512906000
1	-4.995028000	0.754128000	1.639949000
7	-0.211836000	-2.113374000	0.157757000
1	-0.575333000	-0.598705000	1.549479000
6	0.288274000	-0.330188000	-0.352227000
15	2.060135000	-0.267023000	0.191493000
9	0.082266000	-0.310125000	-1.644298000
8	2.137090000	-0.229879000	1.652925000
8	2.787977000	0.861777000	-0.601209000
8	2.569423000	-1.560485000	-0.538958000
6	3.281751000	2.084336000	-0.028585000

6	3.970675000	-1.871070000	-0.613970000
1	3.081981000	2.115946000	1.040240000
1	2.781722000	2.911204000	-0.523780000
1	4.353319000	2.112505000	-0.213303000
1	4.388964000	-1.969337000	0.387336000
1	4.497265000	-1.096157000	-1.169337000
1	4.043075000	-2.819103000	-1.138249000
6	0.290303000	-3.014244000	1.182346000
1	-0.538487000	-3.454218000	1.741608000
1	0.932507000	-2.463013000	1.866661000
1	0.873625000	-3.798712000	0.706674000
35	-0.160810000	2.253232000	0.054811000
1	-0.636280000	-2.562406000	-0.640915000

TS_{T2} (solvent=n,n-DiMethylFormamide, Figures 8 and 9 in the manuscript):

6	-2.224187000	-0.401913000	0.316358000
6	-2.750249000	-0.514107000	-0.968078000
6	-4.101004000	-0.297117000	-1.189691000
6	-4.942161000	0.028318000	-0.135772000
6	-4.426117000	0.138158000	1.146289000
6	-3.074872000	-0.075030000	1.368119000
1	-2.675487000	0.019435000	2.370537000
6	-0.784110000	-0.644999000	0.657759000
1	-2.117545000	-0.763397000	-1.806960000
1	-4.496451000	-0.382834000	-2.193486000
1	-5.996304000	0.197212000	-0.314397000
1	-5.072890000	0.394206000	1.975383000
7	-0.249315000	-2.002153000	0.561167000
1	-0.556061000	-0.244049000	1.640428000
6	0.341732000	-0.344963000	-0.247125000
15	2.090033000	-0.374705000	0.368165000
9	0.189391000	-0.533809000	-1.532179000
8	2.081048000	-0.316027000	1.831694000
8	2.935436000	0.696642000	-0.385821000
8	2.552060000	-1.704127000	-0.325684000
6	3.445020000	1.905520000	0.201733000
6	3.940011000	-2.055022000	-0.455475000
1	4.527043000	1.881057000	0.093902000
1	3.172934000	1.966039000	1.253380000
1	3.020433000	2.744703000	-0.340944000
1	4.411639000	-2.102921000	0.525522000
1	4.451379000	-1.329150000	-1.085372000
1	3.961050000	-3.035168000	-0.922120000
6	-0.839072000	-3.030827000	-0.269424000
1	-1.871636000	-3.232329000	0.022057000
1	-0.243785000	-3.933517000	-0.160587000
1	-0.824070000	-2.735800000	-1.318406000
35	0.020424000	2.275825000	-0.237192000
1	0.226810000	-2.317445000	1.392081000

16a’ (solvent=n,n-DiMethylFormamide, Figures 8 and 9 in the manuscript, **B1** from the Figure 5):

6	0.888300000	-2.783003000	0.955936000
6	1.528401000	-3.170313000	-0.217206000
6	1.967924000	-4.473964000	-0.388886000
6	1.780695000	-5.407789000	0.620107000
6	1.156318000	-5.027614000	1.798980000
6	0.713758000	-3.723485000	1.964691000
1	0.234858000	-3.445279000	2.895219000
6	0.411161000	-1.347571000	1.107386000
1	1.686817000	-2.442738000	-1.004362000
1	2.463434000	-4.758388000	-1.308524000
1	2.127255000	-6.425361000	0.491620000
1	1.014234000	-5.747365000	2.595141000
7	0.384260000	-0.785800000	2.434905000
1	1.082649000	-0.717707000	0.514705000
6	-0.962120000	-1.082773000	0.473534000
15	-1.355603000	0.740086000	0.523422000
9	-0.934498000	-1.456478000	-0.841805000
8	-0.188225000	1.542792000	0.135224000
8	-2.022228000	1.121698000	1.892266000
8	-2.580126000	0.744990000	-0.462602000
6	-1.330172000	1.767226000	2.968619000
6	-3.259693000	1.964535000	-0.790381000
1	-1.959218000	2.590252000	3.300536000
1	-0.364344000	2.142313000	2.636663000
1	-1.189177000	1.048754000	3.771955000
1	-2.562299000	2.682788000	-1.220939000
1	-3.729151000	2.384049000	0.099248000
1	-4.020068000	1.705874000	-1.521443000
6	1.691739000	-0.546680000	3.019739000
1	2.228581000	0.184633000	2.412793000
1	2.310475000	-1.449288000	3.098181000
1	1.562886000	-0.128276000	4.017264000
35	-2.387753000	-2.117235000	1.330923000
1	-0.196598000	-1.333610000	3.055214000

16a’ A1 (solvent=Chloroform, Figure 5 in the manuscript):

6	-1.682257000	0.614623000	-0.119361000
6	-2.278591000	0.519227000	1.134577000
6	-3.555863000	-0.000570000	1.263666000
6	-4.257273000	-0.421072000	0.140086000
6	-3.670302000	-0.324757000	-1.111113000
6	-2.386753000	0.189088000	-1.237583000
1	-1.928641000	0.261631000	-2.216508000
6	-0.306527000	1.239166000	-0.288157000
1	-1.743298000	0.870716000	2.006153000

1	-4.010848000	-0.070208000	2.243684000
1	-5.259009000	-0.818833000	0.242547000
1	-4.210149000	-0.646100000	-1.992719000
7	-0.269952000	2.541604000	0.352977000
1	-0.109924000	1.299103000	-1.368414000
6	0.820156000	0.346073000	0.263925000
15	0.848360000	-1.379933000	-0.443588000
9	0.766461000	0.261115000	1.626240000
8	0.639846000	-1.381737000	-1.890301000
8	-0.205253000	-2.126138000	0.448949000
8	2.227328000	-1.997923000	-0.023471000
6	-1.351842000	-2.825032000	-0.052948000
6	2.629187000	-2.300126000	1.317916000
1	-2.226475000	-2.429699000	0.457942000
1	-1.226067000	-3.882620000	0.170675000
1	-1.450788000	-2.677636000	-1.126418000
1	1.808082000	-2.753231000	1.871608000
1	2.962737000	-1.391119000	1.817459000
1	3.455500000	-3.001215000	1.239810000
6	-1.146767000	3.512685000	-0.283036000
1	-2.189938000	3.236289000	-0.124860000
1	-0.980063000	3.602370000	-1.366120000
1	-0.983723000	4.487735000	0.174349000
35	2.559288000	1.169344000	-0.186588000
1	0.678319000	2.893242000	0.341583000

16a' B1 (solvent=Chloroform, Figure 5 in the manuscript):

6	0.882131000	-2.783041000	0.960101000
6	1.520597000	-3.160027000	-0.216950000
6	1.967839000	-4.459490000	-0.397111000
6	1.789708000	-5.400093000	0.606752000
6	1.165664000	-5.030960000	1.788854000
6	0.715623000	-3.730832000	1.962891000
1	0.235237000	-3.461405000	2.895236000
6	0.398052000	-1.351197000	1.120849000
1	1.669393000	-2.427341000	-1.001199000
1	2.461302000	-4.735764000	-1.320317000
1	2.141811000	-6.414841000	0.471151000
1	1.028730000	-5.756700000	2.580502000
7	0.378598000	-0.796808000	2.452222000
1	1.061645000	-0.714073000	0.527133000
6	-0.980947000	-1.091286000	0.496846000
15	-1.364997000	0.733935000	0.530673000
9	-0.965841000	-1.479185000	-0.812408000
8	-0.194116000	1.528707000	0.141975000
8	-2.029735000	1.129524000	1.899531000
8	-2.591621000	0.737856000	-0.452372000
6	-1.325050000	1.776366000	2.964150000
6	-3.242331000	1.961462000	-0.811748000

1	-1.948142000	2.602034000	3.301834000
1	-0.362298000	2.148667000	2.619540000
1	-1.175691000	1.061042000	3.769289000
1	-2.525137000	2.660492000	-1.241922000
1	-3.720100000	2.404451000	0.062435000
1	-3.995118000	1.704252000	-1.551461000
6	1.689497000	-0.548403000	3.024386000
1	2.208150000	0.198315000	2.420533000
1	2.321515000	-1.443438000	3.083434000
1	1.568454000	-0.143768000	4.028897000
35	-2.398931000	-2.113586000	1.381839000
1	-0.191417000	-1.355592000	3.072561000

16a' C1 (solvent=Chloroform, Figure 5 in the manuscript):

6	-2.126615000	0.362048000	-0.088670000
6	-2.924416000	-0.446967000	0.713556000
6	-4.244805000	-0.696753000	0.376087000
6	-4.791919000	-0.129603000	-0.766008000
6	-4.011608000	0.696227000	-1.560540000
6	-2.690117000	0.942269000	-1.218533000
1	-2.088630000	1.594495000	-1.840522000
6	-0.676790000	0.659606000	0.285826000
1	-2.513525000	-0.889561000	1.613105000
1	-4.849531000	-1.332677000	1.010472000
1	-5.824499000	-0.321665000	-1.028696000
1	-4.432511000	1.155609000	-2.445956000
7	-0.437379000	0.918864000	1.690053000
1	-0.378840000	1.562396000	-0.256675000
6	0.319901000	-0.425938000	-0.193834000
15	0.086062000	-2.132319000	0.510572000
9	1.582254000	-0.054292000	0.190835000
8	-0.174451000	-2.082435000	1.955974000
8	1.369598000	-2.932121000	0.083634000
8	-1.033931000	-2.742142000	-0.392554000
6	2.574286000	-2.944502000	0.864228000
6	-1.970677000	-3.715721000	0.086347000
1	3.032100000	-3.918968000	0.715904000
1	2.347576000	-2.796650000	1.919094000
1	3.240141000	-2.160478000	0.509028000
1	-2.255344000	-3.498072000	1.114814000
1	-1.529769000	-4.709632000	0.021273000
1	-2.840034000	-3.647700000	-0.561446000
6	-1.145014000	2.076965000	2.201069000
1	-0.895798000	2.208476000	3.253601000
1	-2.237642000	2.011329000	2.109492000
1	-0.816447000	2.970305000	1.664535000
35	0.358414000	-0.505858000	-2.149719000
1	-0.611164000	0.093512000	2.248680000

16b' D1 (solvent=Chloroform, Figure 5 in the manuscript):

6	1.761021000	-0.363087000	-0.358211000
6	2.307342000	-0.715688000	0.871991000
6	3.501566000	-0.154788000	1.295093000
6	4.174034000	0.752488000	0.486616000
6	3.644183000	1.095393000	-0.747049000
6	2.443149000	0.540151000	-1.164338000
1	2.026524000	0.815527000	-2.125202000
6	0.476215000	-0.993747000	-0.862434000
1	1.802573000	-1.447578000	1.487369000
1	3.915053000	-0.435075000	2.255649000
1	5.111137000	1.183343000	0.815891000
1	4.164874000	1.794823000	-1.388633000
7	0.505735000	-2.437170000	-0.723577000
1	0.371137000	-0.692337000	-1.917117000
6	-0.808324000	-0.426779000	-0.237113000
15	-0.965650000	1.424944000	-0.349557000
9	-1.863951000	-0.920961000	-0.985435000
8	-0.577830000	1.893143000	-1.683604000
8	-0.179892000	1.978457000	0.880653000
8	-2.447219000	1.709908000	0.087806000
6	0.963412000	2.838973000	0.798227000
6	-3.551674000	1.650221000	-0.824855000
1	0.751506000	3.720731000	1.398804000
1	1.157279000	3.124286000	-0.233844000
1	1.818648000	2.300676000	1.201345000
1	-4.021663000	0.671105000	-0.755480000
1	-3.218247000	1.835990000	-1.845070000
1	-4.252548000	2.422941000	-0.519434000
6	1.486927000	-3.070036000	-1.591233000
1	1.375655000	-2.792200000	-2.649227000
1	1.390367000	-4.151606000	-1.504588000
1	2.494169000	-2.795312000	-1.275339000
35	-1.128519000	-1.021499000	1.596163000
1	-0.411089000	-2.810582000	-0.930181000

16b' E1 (solvent=Chloroform, Figure 5 in the manuscript):

6	0.874686000	-2.783289000	0.896772000
6	1.918059000	-3.124710000	0.045103000
6	2.382358000	-4.430058000	-0.022443000
6	1.804902000	-5.412355000	0.766784000
6	0.766321000	-5.080359000	1.625412000
6	0.307573000	-3.774546000	1.692523000
1	-0.503635000	-3.534131000	2.368283000
6	0.410468000	-1.339567000	0.980149000
1	2.371318000	-2.360426000	-0.575436000
1	3.196972000	-4.677822000	-0.691104000
1	2.164022000	-6.432430000	0.716804000

1	0.311129000	-5.841676000	2.246221000
7	0.442310000	-0.759098000	2.309262000
1	1.069525000	-0.733643000	0.354737000
6	-0.999807000	-1.087745000	0.427478000
15	-1.408292000	0.731112000	0.468493000
9	-1.933544000	-1.769238000	1.162086000
8	-0.299133000	1.554018000	-0.022258000
8	-1.945681000	1.085616000	1.906084000
8	-2.742731000	0.727674000	-0.364195000
6	-1.177126000	1.784632000	2.889280000
6	-3.496858000	1.930350000	-0.545407000
1	-1.776334000	2.630149000	3.222867000
1	-0.236260000	2.134634000	2.469676000
1	-0.979452000	1.111003000	3.719251000
1	-2.874382000	2.704928000	-0.993671000
1	-3.893067000	2.273804000	0.410358000
1	-4.314640000	1.682582000	-1.216258000
6	1.782065000	-0.537742000	2.824678000
1	2.293834000	0.188446000	2.190588000
1	2.393002000	-1.448505000	2.868350000
1	1.712734000	-0.118631000	3.828191000
35	-1.117122000	-1.725092000	-1.417650000
1	-0.094848000	-1.325005000	2.952761000

16b' F1 (solvent=Chloroform, Figure 5 in the manuscript):

6	-1.771870000	-0.710969000	-0.257079000
6	-2.380354000	-0.679075000	-1.506124000
6	-3.621842000	-0.085631000	-1.675592000
6	-4.277042000	0.475929000	-0.589777000
6	-3.684640000	0.433514000	0.663740000
6	-2.441807000	-0.157545000	0.827734000
1	-1.989315000	-0.178764000	1.811416000
6	-0.397079000	-1.361362000	-0.096187000
1	-1.876381000	-1.120399000	-2.357653000
1	-4.080145000	-0.067198000	-2.656341000
1	-5.248706000	0.935880000	-0.717933000
1	-4.192129000	0.861089000	1.519394000
7	-0.133887000	-1.987782000	1.172896000
1	-0.318655000	-2.136086000	-0.866487000
6	0.731065000	-0.363343000	-0.456877000
15	0.827219000	1.143372000	0.632359000
9	0.552572000	0.047626000	-1.752365000
8	0.657486000	0.816867000	2.049692000
8	-0.278448000	2.040097000	-0.035247000
8	2.198350000	1.854925000	0.353312000
6	-1.055337000	2.989246000	0.705353000
6	2.570134000	2.431529000	-0.904923000
1	-1.953475000	3.172208000	0.122177000
1	-0.490150000	3.912913000	0.827242000

1	-1.322542000	2.582978000	1.679631000
1	1.734719000	2.980601000	-1.337737000
1	2.897129000	1.647642000	-1.587465000
1	3.394223000	3.109260000	-0.700374000
6	-0.996952000	-3.110782000	1.479507000
1	-0.860027000	-3.890176000	0.726225000
1	-2.065951000	-2.860895000	1.516156000
1	-0.708588000	-3.524926000	2.445132000
35	2.461652000	-1.274966000	-0.396721000
1	-0.105423000	-1.308107000	1.921234000

16a' A2 (solvent=Chloroform, Figure 5 in the manuscript):

6	-1.692685000	0.582967000	-0.097770000
6	-2.284522000	0.433216000	1.153868000
6	-3.566579000	-0.077781000	1.270131000
6	-4.279978000	-0.440894000	0.134512000
6	-3.698806000	-0.295790000	-1.114481000
6	-2.411544000	0.212219000	-1.226867000
1	-1.959499000	0.321192000	-2.205343000
6	-0.319941000	1.229969000	-0.257065000
1	-1.744543000	0.717978000	2.048372000
1	-4.013099000	-0.189888000	2.250027000
1	-5.284576000	-0.833648000	0.226361000
1	-4.246468000	-0.574014000	-2.005895000
7	-0.224592000	2.548982000	0.335129000
1	-0.134595000	1.334678000	-1.328066000
6	0.827781000	0.347898000	0.264355000
15	0.841469000	-1.382572000	-0.427133000
9	0.782020000	0.281213000	1.637320000
8	0.624323000	-1.386902000	-1.873014000
8	-0.217228000	-2.121319000	0.468510000
8	2.216229000	-2.014622000	-0.013146000
6	-1.352874000	-2.832121000	-0.041513000
6	2.628453000	-2.292107000	1.329933000
1	-2.235774000	-2.442729000	0.459767000
1	-1.221158000	-3.888179000	0.186231000
1	-1.443256000	-2.688869000	-1.116353000
1	1.809304000	-2.727250000	1.901027000
1	2.974284000	-1.375594000	1.807088000
1	3.448314000	-3.001520000	1.259018000
6	-1.094559000	3.531235000	-0.288628000
1	-2.159084000	3.262579000	-0.265065000
1	-0.801183000	3.661390000	-1.332259000
1	-0.969057000	4.488004000	0.216980000
35	2.545249000	1.165687000	-0.213722000
1	-0.403262000	2.502444000	1.329286000

16a' B2 (solvent=Chloroform, Figure 5 in the manuscript):

6	-2.076732000	0.489053000	-0.169593000
6	-2.915972000	-0.202876000	0.695519000
6	-4.217397000	-0.499589000	0.321187000
6	-4.693896000	-0.106345000	-0.921301000
6	-3.864155000	0.592599000	-1.785515000
6	-2.564035000	0.891236000	-1.407356000
1	-1.920900000	1.443269000	-2.082731000
6	-0.646338000	0.814396000	0.222250000
1	-2.542051000	-0.494992000	1.667534000
1	-4.862746000	-1.038145000	1.003794000
1	-5.710940000	-0.337520000	-1.212082000
1	-4.230370000	0.910864000	-2.753379000
7	-0.505714000	1.045166000	1.641810000
1	-0.362848000	1.704812000	-0.366397000
6	0.348717000	-0.259579000	-0.272056000
15	2.115384000	0.257441000	-0.033275000
9	0.147714000	-0.443915000	-1.611760000
8	2.485241000	0.440305000	1.375290000
8	2.268231000	1.554490000	-0.922092000
8	2.860147000	-0.838703000	-0.860919000
6	2.247829000	2.879779000	-0.376723000
6	4.292866000	-0.865826000	-0.948459000
1	2.968084000	3.467266000	-0.940335000
1	2.520730000	2.865695000	0.677226000
1	1.252053000	3.305579000	-0.500895000
1	4.729412000	-0.929892000	0.047519000
1	4.652805000	0.024691000	-1.463541000
1	4.545598000	-1.752241000	-1.522355000
6	-1.071170000	2.310902000	2.073426000
1	-0.872437000	2.446144000	3.135668000
1	-2.152986000	2.306110000	1.930311000
1	-0.663102000	3.176191000	1.529968000
35	0.097654000	-1.985257000	0.604625000
1	0.462573000	0.974910000	1.928264000

16a' C2 (solvent=Chloroform, Figure 5 in the manuscript):

6	-2.229788000	0.468202000	-0.154567000
6	-3.049969000	-0.301380000	0.661732000
6	-4.399966000	-0.431041000	0.380314000
6	-4.949899000	0.214574000	-0.718094000
6	-4.142498000	0.998437000	-1.527713000
6	-2.791773000	1.127200000	-1.241213000
1	-2.167014000	1.748688000	-1.871572000
6	-0.754628000	0.646827000	0.150903000
1	-2.629706000	-0.798140000	1.524614000
1	-5.025491000	-1.038382000	1.022169000
1	-6.005473000	0.113024000	-0.936763000
1	-4.563767000	1.516348000	-2.379967000
7	-0.528166000	0.825642000	1.575130000

1	-0.409755000	1.523034000	-0.421471000
6	0.174223000	-0.485640000	-0.343270000
15	0.022394000	-2.181395000	0.419866000
9	1.463201000	-0.059687000	-0.104913000
8	-1.279930000	-2.821271000	0.230666000
8	0.490253000	-2.039510000	1.919228000
8	1.270028000	-2.852182000	-0.258253000
6	-0.421216000	-2.058974000	3.025985000
6	1.597856000	-4.221374000	0.006010000
1	0.151383000	-2.388392000	3.889324000
1	-1.237078000	-2.753892000	2.832273000
1	-0.807723000	-1.053133000	3.182570000
1	0.754043000	-4.866569000	-0.237489000
1	1.877133000	-4.346195000	1.052504000
1	2.443180000	-4.462878000	-0.631876000
6	-1.101886000	2.056325000	2.098210000
1	-0.790090000	2.178367000	3.134881000
1	-2.190432000	2.001784000	2.075612000
1	-0.794344000	2.947697000	1.533537000
35	-0.002079000	-0.694361000	-2.276929000
1	0.466748000	0.823023000	1.754748000

16b' D2 (solvent=Chloroform, Figure 5 in the manuscript):

6	1.751067000	-0.357853000	-0.340568000
6	2.274919000	-0.650429000	0.915481000
6	3.470019000	-0.085644000	1.331313000
6	4.167998000	0.771345000	0.490926000
6	3.660354000	1.060739000	-0.765429000
6	2.459553000	0.499170000	-1.174845000
1	2.062169000	0.734577000	-2.154455000
6	0.476769000	-1.017915000	-0.855331000
1	1.753230000	-1.327955000	1.579312000
1	3.861063000	-0.320660000	2.313088000
1	5.105021000	1.206456000	0.814401000
1	4.198747000	1.722915000	-1.431508000
7	0.459838000	-2.464091000	-0.789030000
1	0.393652000	-0.750624000	-1.913454000
6	-0.827761000	-0.448571000	-0.270539000
15	-0.956441000	1.406528000	-0.339242000
9	-1.865827000	-0.898841000	-1.049511000
8	-0.561104000	1.890446000	-1.665699000
8	-0.155737000	1.923807000	0.898168000
8	-2.432759000	1.710195000	0.104352000
6	0.962788000	2.817236000	0.818899000
6	-3.541477000	1.669440000	-0.804630000
1	0.718258000	3.704980000	1.398171000
1	1.167610000	3.086670000	-0.215267000
1	1.824026000	2.308938000	1.246956000
1	-4.018930000	0.693323000	-0.744804000
1	-3.210475000	1.864068000	-1.823959000

1	-4.234815000	2.444163000	-0.487344000
6	1.468073000	-3.093729000	-1.625190000
1	1.269013000	-2.857799000	-2.672568000
1	1.402032000	-4.174684000	-1.506471000
1	2.495478000	-2.781016000	-1.395752000
35	-1.195365000	-1.084557000	1.548189000
1	0.555697000	-2.773383000	0.169154000

16b' E2 (solvent=Chloroform, Figure 5 in the manuscript):

6	0.923274000	-2.588708000	0.968192000
6	1.937155000	-2.921099000	0.079771000
6	2.401560000	-4.225283000	-0.008155000
6	1.852996000	-5.210842000	0.797779000
6	0.846255000	-4.883165000	1.695907000
6	0.387658000	-3.578665000	1.785011000
1	-0.380984000	-3.317431000	2.499659000
6	0.429260000	-1.156874000	1.060345000
1	2.367333000	-2.152332000	-0.551758000
1	3.194151000	-4.469515000	-0.704117000
1	2.213049000	-6.229886000	0.732489000
1	0.419513000	-5.647166000	2.333661000
7	0.407088000	-0.718486000	2.445112000
1	1.096656000	-0.537522000	0.439514000
6	-0.973348000	-0.959346000	0.442057000
15	-1.533106000	0.814715000	0.400055000
9	-1.927120000	-1.617852000	1.172226000
8	-1.606038000	1.397423000	1.746368000
8	-2.884255000	0.844936000	-0.401325000
8	-0.491486000	1.432151000	-0.591870000
6	-4.159086000	0.664259000	0.233564000
6	-0.444750000	2.845600000	-0.840689000
1	-4.889802000	1.174816000	-0.387740000
1	-4.150792000	1.095201000	1.233503000
1	-4.389833000	-0.398837000	0.282841000
1	-0.361666000	3.391058000	0.098640000
1	-1.338273000	3.156800000	-1.380914000
1	0.436245000	3.018497000	-1.451453000
6	1.736896000	-0.559677000	3.006517000
1	2.382792000	0.104496000	2.412843000
1	2.231880000	-1.529477000	3.080611000
1	1.650295000	-0.147869000	4.011387000
35	-1.038463000	-1.685338000	-1.372318000
1	-0.115982000	0.143401000	2.536976000

16b' F2 (solvent=Chloroform, Figure 5 in the manuscript):

6	-1.890810000	-0.803881000	-0.385280000
6	-2.538873000	-0.821519000	-1.614261000
6	-3.822593000	-0.314160000	-1.744736000

6	-4.476961000	0.205622000	-0.638355000
6	-3.840319000	0.214072000	0.594543000
6	-2.554763000	-0.286231000	0.719524000
1	-2.058182000	-0.276961000	1.679885000
6	-0.487090000	-1.378886000	-0.277866000
1	-2.034820000	-1.235890000	-2.479356000
1	-4.313158000	-0.331728000	-2.709688000
1	-5.481427000	0.598241000	-0.734787000
1	-4.346294000	0.615423000	1.463557000
7	-0.233975000	-1.959276000	1.024586000
1	-0.392159000	-2.123996000	-1.083669000
6	0.599677000	-0.330875000	-0.608171000
15	0.854604000	1.068034000	0.601117000
9	0.297966000	0.241641000	-1.807602000
8	-0.355048000	1.852028000	0.855267000
8	2.028975000	1.806936000	-0.134596000
8	1.537074000	0.455377000	1.881112000
6	2.493968000	3.079221000	0.332675000
6	0.836081000	0.301101000	3.124544000
1	3.227513000	3.421954000	-0.391341000
1	2.963686000	2.970701000	1.310726000
1	1.667401000	3.786809000	0.389189000
1	0.187447000	-0.571390000	3.063817000
1	0.250481000	1.192842000	3.340397000
1	1.598584000	0.159674000	3.885969000
6	-1.015050000	-3.160018000	1.283363000
1	-0.910319000	-3.916609000	0.493560000
1	-2.071981000	-2.909171000	1.372705000
1	-0.689682000	-3.595663000	2.227254000
35	2.311685000	-1.281389000	-0.862167000
1	0.748688000	-2.188759000	1.098561000
